# Ruthenium Catalyzed *Ortho*‐Arylation Reaction of Benzoic Acids with Arylthianthrenium Salts

**DOI:** 10.1002/anie.202504888

**Published:** 2025-05-22

**Authors:** Kaiping Wang, Xiaowen Teng, Duo Zhang, Bingxin Xu, Pan Gao, Shuli Wang, Shuwei Zhang, Lukas J. Gooßen, Feng Chen, Guodong Zhang

**Affiliations:** ^1^ School of Chemistry and Chemical Engineering Yangzhou University Siwangting Road 180 Yangzhou 225002 China; ^2^ Medicine Center Guangxi University of Science and Technology Liushi Road 257 Liuzhou 545006 China; ^3^ Fakultät für Chemie und Biochemie Ruhr‐Universität Bochum Universitätsstr. 150 44801 Bochum Germany

**Keywords:** Aromatic carboxylates, Arylthianthrenium salts, C─H arylation, Decarboxylation, Ruthenium

## Abstract

Arylthianthrenium salts have become key intermediates for late‐stage functionalizations of drug‐like molecules. Ruthenium‐phosphine catalysts are now shown to enable their use as aryl sources in high‐yielding *ortho*‐C─H arylations of benzoic acids. The arylthianthrenium salts are converted chemo‐selectively, leaving aryl halides and boronates untouched. The carboxylate groups are uniquely effective as directing groups, ensuring exclusive *ortho*‐selectivity even in the presence of competing pyridine or amide groups. This makes the reaction orthogonal to cross‐couplings and conventional C─H arylations. The carboxylate group can be removed via decarboxylation or serve as an anchor for downstream transformations. Mechanistic studies identify C─H ruthenation as the rate‐limiting step and highlight the unique efficiency of P(Cy)₃ ligands.

## Graphical abstract

A catalytic *ortho‐*arylation method has been developed to convert arylthianthrenium salts into carboxylate‐functionalized biaryls, which can be removed in situ or used for further derivatization. This reaction is broadly applicable to (hetero)arenecarboxylic acids, with excellent functional group compatibility and orthogonality to cross‐couplings, enhancing the utility of thianthrenium salts in drug discovery.

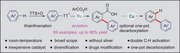

Several decades after the pioneering work by Calvin,^[^ ^1^ ^]^ Lucken,^[^ ^2^ ^]^ and Shine,^[^ ^3^, ^4^, ^5^ ^]^ aryl sulfonium salts have been rediscovered as versatile intermediates for the late‐stage C─H functionalization of complex organic molecules.^[^ ^6^, ^7^, ^8^ ^]^ In 2019, Ritter and co‐workers demonstrated that the thianthrenium (TT) tetrafluoroborate moiety can be regioselectively introduced into specific C─H groups of diversely functionalized arenes.^[^ ^9^ ^]^ It can then serve as a leaving group in various catalytic cross‐couplings or photo‐redox reactions, enabling the regioselective formation of C─C, C─N, C─O, C─S, or C─halogen bonds as demonstrated by Ritter,^[^ ^10^, ^11^, ^12^, ^13^, ^14^ ^]^ Alcarazo,^[^ ^15^, ^16^ ^]^ Procter,^[^ ^17^, ^18^, ^19^ ^]^ Ackermann,^[^ ^20^, ^21^, ^22^ ^]^ and others.^[^ ^23^, ^24^, ^25^, ^26^ ^]^

Whereas the cross‐coupling chemistry of sulfonium salts is approaching synthetic maturity, their application in directed C─H functionalization is still in its infancy. First examples include photocatalytic couplings of structurally constrained arenes,^[^ ^9^, ^19^, ^27^, ^28^ ^]^ Pd‐catalyzed arylations of azoles and indoles (Scheme 1a),^[^ ^20^, ^29^ ^]^ and an *ortho* C─H arylation of arenes with dibenzothiophenium (DBT) salts based on triazole and tetrazole directing groups by Ackermann et al (Scheme 1b).^[^ ^22^ ^]^ The synthetic value of the latter is somewhat limited by the necessity to install and remove strongly coordinating directing groups and the high reaction temperatures. The use of simple, abundantly available, and easily transformable functionalities such as carboxylate groups would be highly advantageous.^[^ ^30^ ^]^

**Scheme 1:**
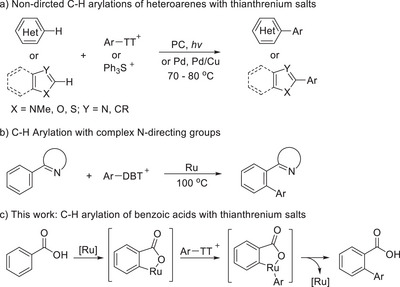
Direct C─H bond arylations with arylsulfonium salts.

Despite the structural simplicity of the carboxylate group, Yu,^[^ ^31^, ^32^, ^33^ ^]^ Daugulis,^[^ ^34^ ^]^ Li,^[^ ^35^ ^]^ Larrosa,^[^ ^36^, ^37^, ^38^ ^]^ Ackermann,^[^ ^39^ ^]^ Gooßen,^[^ ^40^, ^41^, ^42^ ^]^ and others have managed to utilize carboxylates as directing groups in C─H functionalizations with aryl halides,^[^ ^43^, ^44^, ^45^ ^]^ boronates,^[^ ^31^, ^46^ ^]^ diazonium salts,^[^ ^41^ ^]^ hypervalent iodine reagents,^[^ ^47^ ^]^ and (hetero)arenes.^[^ ^48^, ^49^, ^50^, ^51^ ^]^ After serving as directing groups, the carboxylate groups can be derivatized, cleaved tracelessly, or used as leaving group in various decarboxylative couplings with formation of C─C or C─heteroatom bonds.^[^ ^52^, ^53^, ^54^, ^55^, ^56^, ^57^, ^58^ ^]^ A catalytic C─H arylation of (hetero)arenecarboxylic acids that proceeds in high yields under mild conditions at room temperature would be of substantial synthetic value and markedly increase the scope of compounds accessible from arenes via C─H thianthrenations.

We reasoned that the desired conversion might be achieved with ruthenium catalysts (Scheme 1c). The ability of ruthenium complexes to insert into C─H groups *ortho* to carboxylates with formation of cyclometalated Ru(II) complexes via base assisted concerted metalation‐deprotonation (CMD) is amply documented.^[^ ^39^, ^40^, ^59^ ^]^ Electron‐donating ligands might make such complexes sufficiently electron‐rich to allow their insertion into the C─S bond of thianthrenium salts. Reductive elimination would then liberate the biarylcarboxylic acid product and regenerate the Ru‐catalyst.

To validate this hypothesis, we chose *ortho*‐toluic acid (**1**) and *p*‐methoxyphenylthianthrenium salt (**2**) as model substrates and investigated various ligands and solvents in combination with 2.5% the inexpensive ruthenium complex [Ru(*p*‐cymene)Cl₂]₂ and the mild base K₂CO₃ (Tables 1 and S1).

**Table 1:** Optimization of the reaction conditions.^a)^

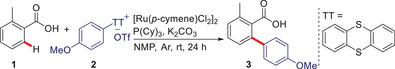		
Entry	Variation from standard conditions	Yield (%)^b)^
1	Without P(Cy)_3_	<1
2	None	98
3	P(*n*‐Bu)_3_ as the ligand	13
4	PPh_3_ as the ligand	72
5	P(C_6_F_5_)_3_, 1,10‐Phen, Boc‐L‐Valine as the ligand	<1
6	Without K_2_CO_3_	<1
7	K_3_PO_4_, Na_3_PO_4_, Na_2_CO_3_ as the base	50, 39, 76
8	Acetone, DMAc, dioxane as the solvent	33, 51, 22
9	under air	<1

^a)^ Conditions: 0.20 mmol **1**, 1.2 equiv. **2**, 2.5 mol% [Ru(*p*‐cymene)Cl_2_]_2_, 10 mol% P(Cy)_3_, 1.2 equiv. K_2_CO_3_, 4 mL NMP, under an argon, rt, 24 h.

^b)^ GC‐yields of the methyl esters following esterification with K_2_CO_3_ and MeI in MeCN using *n*‐tetradecane as the internal standard.

Whereas no conversion was observed at room temperature in the absence of ligand, the addition of electron‐rich, monodentate phosphines led to the formation of the desired product **3**, with tricyclohexylphosphine (PCy_3_) being most effective (entries 1, 2). Amino acids such as Boc‐L‐Valine,^[^ ^60^, ^61^ ^]^ or chelating amines such as 1,10‐phenanthroline were ineffective (entries 3–5). The addition of a base is crucial, with best results obtained with K₂CO₃ (entries 6–7). Polar aprotic solvents such as NMP facilitate the reaction (entry 8, details see Table S1). Further control experiments revealed that the reaction is sensitive to air but much less the presence of water (entry 9).

Systematic studies revealed the broad scope of the transformation and its orthogonality to traditional cross‐coupling and C─H functionalization reactions (Table 2). In all cases, the reaction proceeds selectively. Moderate yields are due to incomplete conversion, and no byproducts such as phenols or aryl esters were observed. Aromatic and heteroaromatic carboxylic acids were successfully coupled with *p*‐methoxyphenylthianthrenium salt (**3**–**26**), with the highest yields being obtained for electron‐rich substrates. Various functional groups are tolerated, including amines (**13**), ethers (**4**–**6**, **14**), amides (**15**), esters (**10**, **17**), and carbonyl groups (**9**, **18**). C─C bond formation takes place selectively in the less hindered C─H position for *meta*‐substituted carboxylates (**12**–**18**). Solely *para*‐ or unsubstituted benzoic acids partially undergo double arylation (**19**). This is a drawback which is commonly observed for Rh‐ or Ru‐catalyzed *ortho* arylations. Multi‐substituted benzoic acids and naphthylcarboxylic acids also gave satisfactory to good yields (**20**–**24**). The applicability of the reaction to heteroaromatic carboxylic acids is exemplified by the coupling of indole‐2‐carboxylic acid (**25**) and thiophene‐2‐carboxylic acid (**26**).

**Table 2:** Substrate scope.^a)^

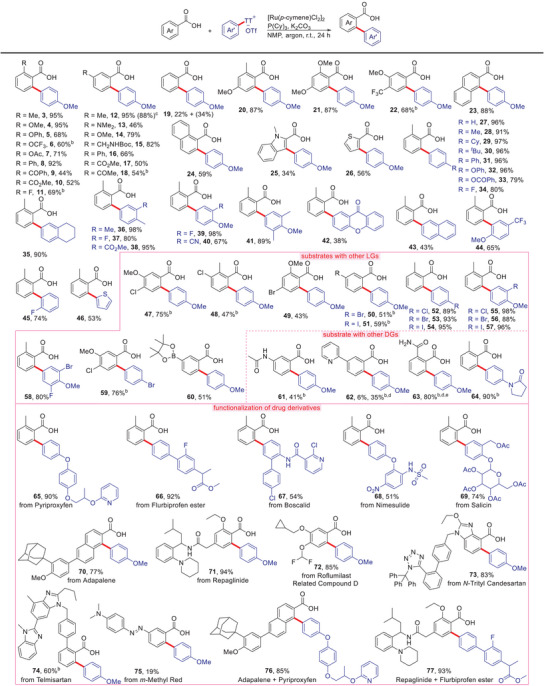

^a)^ Conditions: 0.2 mmol arenecarboxylic acids, 1.2 equiv. thianthrenium salts, 2.5 mol% Ru(*p*‐cymene)Cl_2_]_2_, 10 mol% P(Cy)_3_, 1.2 equiv. K_2_CO_3_, 4 mL NMP, under an argon, rt, 24 h. Isolated yields of the corresponding methyl esters.

^b)^ with 5 mol% [Ru(*p*‐cymene)Cl_2_]_2_ and 20 mol% P(Cy)_3_.

^c)^ 5 mmol scale [d] at 60 °C. [e] starting from 2‐((1‐phenylethyl)carbamoyl)benzoic acid after acidification.

The scope of arylthianthrenium salt substrates proved to be similarly broad (**27**–**46**). The method by Ritter was used to synthesize diversely functionalized derivatives in predictable geometries. The thiathrenium unit is usually positioned *para* to electron‐donating functionalities. Only if the *para*‐position is blocked, *ortho*‐thianthrenolysis is achieved (**44**). Even electron‐deficient thianthrenium salts, which are accessible from boronic acids,^[^ ^62^ ^]^ were successfully coupled (**45**).

Both in the carboxylate and the thianthrenium substrates, nucleophilic leaving groups such as chloride, bromide, and even highly reactive iodide substituents are left intact (**47**–**59**). A key advantage of our system is its orthogonality to the established catalysts that are based on aryl halide substrate. The preparative utility of this is demonstrated in two examples, in which we first coupled with one molecule of benzoic acid, and then reacted with another molecule of benzoic acid (SI, **100**–**101**). Electrophilic groups such as pinacol borates are also tolerated (**60**). This allows introducing functionalities that can later serve as leaving groups in Suzuki‐reactions, C─heteroatom bond formations, etc.^[^ ^63^, ^64^ ^]^

Moreover, the arylation takes place exclusively in the *ortho*‐position of the carboxylate groups even in the presence of strongly coordinating pyridyl and amide groups (**61**–**64**). These functionalities can subsequently be used as directing groups (DGs) for conventional C─H functionalization.^[^ ^65^, ^66^ ^]^ Competition experiments confirmed that carboxylates are uniquely effective as directing groups. Neither pyridine, acyl amino, amide, ester, keto, nor other directing groups, including Daugulis amides and Ackermann's tetrazoles, gave any conversion (see Table S2).^[^ ^22^ ^]^

The synthesis of the thianthrenium salts and their subsequent coupling with the benzoic acid can be carried out on gram scales as demonstrated for compound **12,** which was isolated in an overall 88% yield as the free acid starting from 5 mmol of *m*‐toluic acid. The other examples in Tables 2 were isolated as their methyl esters to simply their purification via column chromatography. The application of the method to the late‐stage functionalization of natural product and drug derivatives is also illustrated in Table 2. The reaction was also used to fuse two biologically meaningful substrates, that is, adapalene and pyriproxyfen (**76**).

The examples in Table 3 demonstrate that the carboxylate group can be tracelessly removed in situ by adding copper(I) to the reaction solution and heating it to 195 °C (**77**–**91**). The one‐pot arylation/decarboxylation protocol is compatible with substrates that are challenging to access via halide precursors (**93**). It proved to be tolerant of OAc groups (**83**) and halogens (**88**–**92**), but failed for NHAc and Bpin groups. Interestingly, for the substrate bearing an ester group in benzylic position, intramolecular transesterification was observed, and the decarboxylation took place in the benzylic position (**94**).

**Table 3:** One‐pot arylation/decarboxylation.^a)^

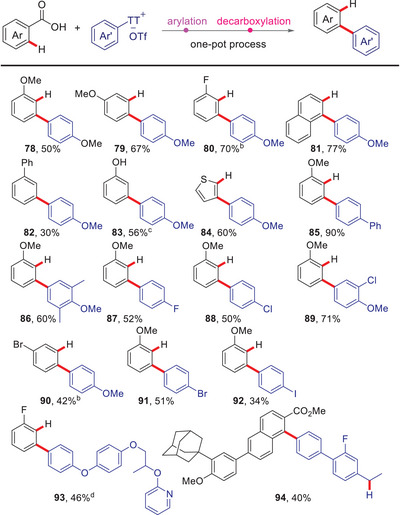

^a)^ Conditions: 0.20 mmol arenecarboxylic acids, 1.2 equiv. thianthrenium salts, 2.5 mol% [Ru(*p*‐cymene)Cl_2_]_2_, 10 mol% P(Cy)_3_, 1.2 equiv. K_2_CO_3_, 4 mL NMP, under an argon, rt, 24 h; then 1 equiv. Cu_2_O, 195 °C, 12 h. isolated yields.

^b)^ with 5 mol% [Ru(*p*‐cymene)Cl_2_]_2_, 20 mol% P(Cy)_3_.

^c)^ starting from acetylsalicylic acid.

^d)^ 0.5 equiv. Ag_2_CO_3_ instead of Cu_2_O, 160 °C.

The carboxylate groups can also be transformed into other functional groups such as lactones, alcohols, cyano groups, or imides as illustrated in Scheme 2.

**Scheme 2:**
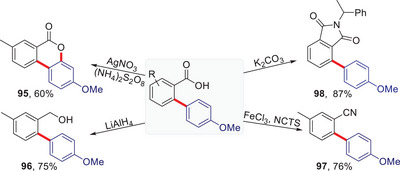
Derivatizations of *o*‐arylated benzoic acids. See SI for details.

Numerous control experiments were conducted to clarify the reaction pathway. When *ortho*‐toluic acid was treated with the Ru/PCy_3_ catalyst in the presence of D_2_O, 87% deuteration at the *ortho*‐position was observed (Scheme 3a). In combination with the profound kinetic isotope effects measured in parallel (3:1) and competition experiments (5:1), one can conclude that the initiating CMD step is slow and reversible (Scheme 3b). A one‐pot competition experiment revealed that electron‐rich benzoic acids react faster than electron‐deficient ones. This trend is also observed in parallel experiments, giving further evidence that the CMD step, which is known to be facilitated by electron‐rich arenes, is rate limiting (Scheme 3c).

**Scheme 3:**
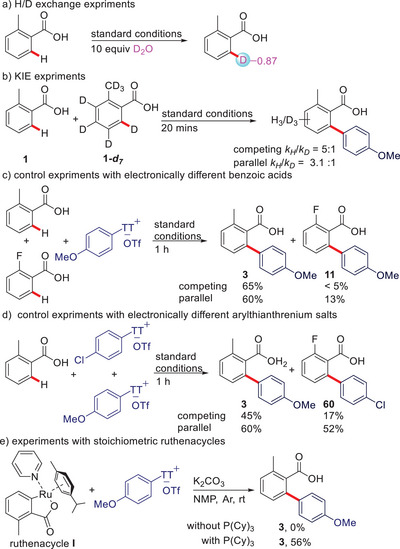
Mechanistic studies.

In a one‐pot competition experiment of an electron‐rich and an electron‐deficient thianthrenium salt, the electron‐rich one reacts faster. When performing the reactions in parallel, both substrates are converted at similar rates (Scheme 3d). This is expected for a reaction that is limited by the rate of the CMD step. Since the electron‐rich electrophile reacts faster than the electron‐deficient one, the reductive elimination, which is facilitated by electron‐donating substituents, seems to be slower than the oxidative addition, which is accelerated by electron‐deficient substituents. When adding an arylthianthrenium salt to a solution of [Ru(*p*‐cymene)Cl_2_]_2_ and PCy_3_, only the dichloro(*p*‐cymene)(tricyclohexylphosphine)ruthenium(II) was detected by ^31^P‐NMR after 2 h (Figure S4). In contrast, the formation of ruthenacycle takes place swiftly. This suggest that the C─H ruthenation is the initial step in the catalytic cycle. When starting from the cyclometallated intermediate, no conversion is observed without PCy_3_. An NMR study revealed that no oxidative addition of added thianthrenium salt takes place. As soon as PCy_3_ is added, rapid arylation of the coordinated benzoate took place (Schemes 3e and S9). This shows that the PCy_3_ ligand enables the oxidative addition of the thianthrenium salt. Interestingly, it accelerated this step so strongly, that it is not rate determining in the optimal protocol.

Overall, a catalytic *ortho*‐arylation method has been developed that allows converting arylthianthrenium salts into carboxylate‐functionalized biaryls. The reaction is broadly applicable, tolerant of common functional groups and was shown to be orthogonal to common cross‐coupling strategies. The carboxylate group can be removed in situ or used as an anchor for further derivatization. The new transformation greatly expands the utility of thianthrenium salts as synthetic lynch pins in drug discovery.

## Conflict of Interests

The authors declare no conflict of interest.

## Supporting information



Supporting Information

## Data Availability

The data that support the findings of this study are available in the Supporting information of this article.
