# Supplementary material for: Ruthenium Catalyzed Ortho‐Arylation Reaction of Benzoic Acids with Arylthianthrenium Salts
Source: Angew Chem Int Ed Engl. 2025 May 22;64(26):e202504888. doi: 10.1002/anie.202504888 (PMC12184312; doi:10.1002/anie.202504888)
Supplement: Supplementary file 1 — Supporting Information [file ANIE-64-e202504888-s001.pdf]

Supporting Information

©Wiley-VCH 2025
69451 Weinheim, Germany

Ruthenium Catalyzed *ortho*-Arylation Reaction of Benzoic Acids with Arylthianthrenium Salts

Kaiping Wang,+[a] Xiaowen Teng,+[a] Duo Zhang,+[b] Bingxin Xu,[b] Pan Gao,[a] Shuli Wang,[a] Shuwei Zhang,[a] Lukas J. Gooßen,*[c] Feng Chen,*[a] Guodong Zhang*[a]

a School of Chemistry and Chemical Engineering, Yangzhou University, Siwangting Road 180, 225002, Yangzhou (China).
E-mail: guodong.zhang@yzu.edu.cn; feng.chen@yzu.edu.cn

b Medicine Center, Guangxi University of Science and Technology, Liushi Road 257, 545006, Liuzhou, Guangxi (China).

c Fakultät für Chemie und Biochemie, Ruhr-Universität Bochum, Universitätsstr. 150, 44801 Bochum (Germany).
E-mail: lukas.goossen@rub.de

+These authors contributed equally.

Table of Contents

Table of Contents 1

General Methods 2

Preparation of the Starting Materials 2

Optimization of the Reaction Conditions 2

Screening of Other Directing Groups 3

Mechanistic Studies 4

Synthesis and Characterization of the Corresponding Products 15

Gram-scale Reaction 65

Further Derivatization 66

Further subsequent couplings with benzoic acids 69

Copies of ^{1}H and ^{13}C NMR Spectra 72

References 173

General Methods

All reagents were purchased from commercial suppliers without further purification, and solvents were degassed with argon prior to use. Reactions were performed in oven-dried glassware under an argon atmosphere containing a Teflon-coated stirring bar and dry septum. All reactions were monitored by GC using tetradecane as an internal standard. Analytical TLC was performed with silica gel GF254 plates, and the products were visualized by UV detection. Flash column chromatography as performed over silica gel (200-300 mesh). NMR spectra were recorded on an Agilent DD2 400 MHz spectrometers using $CDCl_3$ or DMSO-d_6 as solvent with proton (400) and carbon (101) resonances. Chemical shifts (δ values) were reported in ppm relative to internal TMS (^{1}H NMR) or $CDCl_3$ (^{13}C NMR), respectively, and spin-spin coupling constants (*J*) were given in Hz. Melting points were measured on a Yanaco Micro Melting Point Apparatus. The High-resolution mass spectral (HRMS) data were obtained on Bruker Daltonics maXis Q-TOF (APCI and ESI).

Preparation of the Starting Materials

All the carboxylic acids were commercially available with further purification.

All the arylthianthrenium salts were prepared according to known literatures from arenes or arylboronic acids with thianthrene S-oxide in DCM or MeCN.[1] The arylthianthrenium salts were precipitated and washed with diethyl ether without further purification for the direct use.

Optimization of the Reaction Conditions

Table S1: Optimization of the reaction conditions using *ortho*-toluic acid (**1**) and *p*-methoxyphenylthianthrenium salt (**2**).[a]

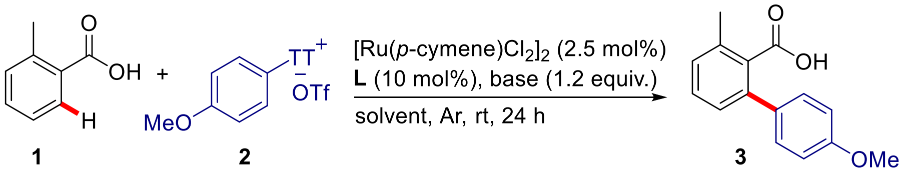

Entry	Ligand	Base	Solvent	Yield (%) [b]
1	-	K_2CO_3	NMP	<1
2	P(Cy)$_3$	’’	’’	98
3	PPh$_3$	’’	’’	72
4	P(*n*-Bu)$_3$	’’	’’	13
5	P(C_6F_5)$_3$	’’	’’	<1
7	Boc-L-Valine	’’	’’	<1
8	1,10-Phen	’’	’’	<1
9[c]	P(Cy)$_3$	’’	’’	65

10	$P(Cy)_3$	K_3PO_4	”	50
11	”	Na_3PO_4	”	39
12	”	Na_2CO_3	”	76
13	”	$KHCO_3$	”	46
14	”	-	”	<1
15	”	K_2CO_3	acetone	33
16	”	”	DMAc	51
17	”	”	dioxane	22
18	”	”	DCE	<1
19	”	”	MeCN	<1
20[d]	”	”	NMP	<1

[a] Reaction conditions: **1** (0.2 mmol), **2** (1.2 equiv.), $[Ru(p\text{-cymene})Cl_2]_2$ (2.5 mol%), Ligand (10 mol%), base (1.2 equiv.), solvent (4 mL), under an argon atmosphere, room temperature, 24 h. [b] Yields of corresponding methyl esters determined by GC after esterification with K_2CO_3 (3.0 equiv.) and MeI (5.0 equiv.) in MeCN using *n*-tetradecane as the internal standard. [c] Ligand (5 mol%) was used. [d] under air.

Screening of Other Directing Groups

Table S2: Screening of unsuccessful directing groups for Ru-catalyzed *ortho*-arylation with arylsulfonium salts [a]

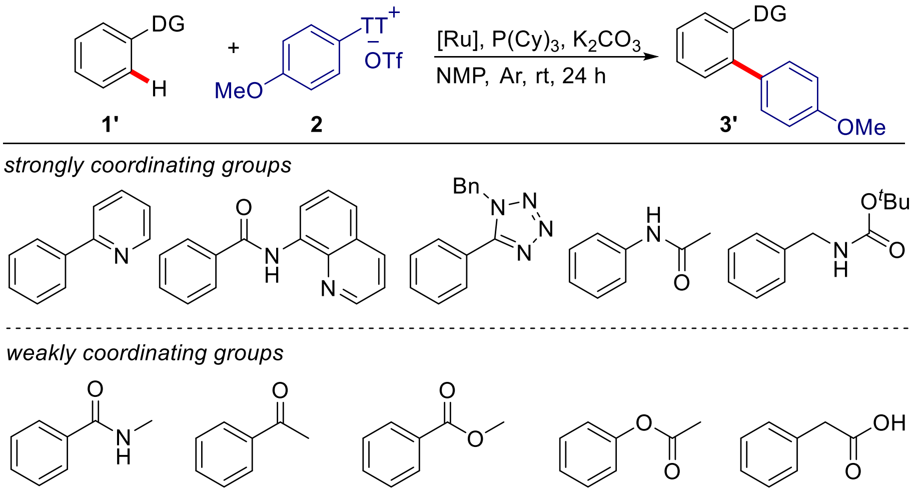

[a] Reaction conditions: **1’** (0.2 mmol), **2** (1.2 equiv.), $[Ru(p\text{-cymene})Cl_2]_2$ (2.5 mol%), $P(Cy)_3$ (10 mol%), K_2CO_3 (1.2 equiv.), NMP (4 mL), under an argon atmosphere, room temperature, 24 h.

Mechanistic Studies

Deuterium Exchange Experiment:

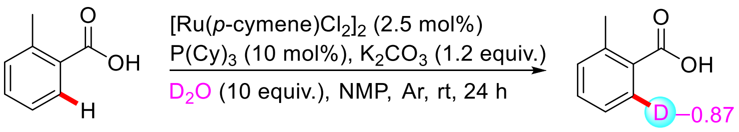

Scheme S1. H/D-exchange experiment of **1a**.

Deuterium Exchange Experiment: An oven-dried 10 mL vessel was charged with 2-methylbenzoic acid (28.2 mg, 0.20 mmol, 1 equiv.), [Ru(*p*-cymene)Cl_2]$_2$ (3.2 mg, 5.00 μmol, 0.025 equiv.), K_2CO_3 (33.6 mg, 0.24 mmol, 1.2 equiv.), P(Cy)$_3$ (5.6 mg, 0.02 mmol, 0.1 equiv.). Under exclusion of air, NMP (4 mL) and D_2O (40.1 mg, 2.0 mmol, 10 equiv.) were added via syringe. The reaction mixture was stirred at rt for 24 h. MeCN (2 mL), K_2CO_3 (83.0 mg, 0.60 mmol, 3 equiv.) and CH_3I (141.9 mg, 1.0 mmol, 5 equiv.) were added and the mixtures were stirred at 60 °C for 2.5 h. Brine (10 mL) was added and the resulting mixture was extracted with ethyl acetate (3×20 mL). The combined organic layers were dried over $MgSO_4$, filtered, and the volatiles were removed under reduced pressure. The residue was purified by column chromatography (SiO_2, Petroleum Ether: EtOAc = 40:1), was determined on the basis of ^{1}H NMR analysis.

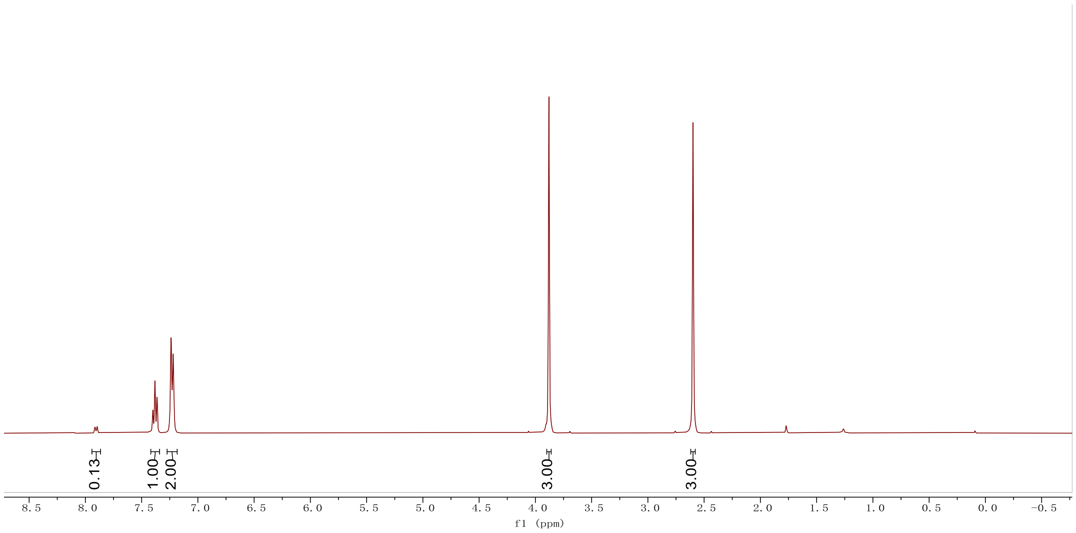

Figure S1. ^{1}H-NMR profile of (D)-**1a** after H/D-exchange experiment.

KIE measurements:

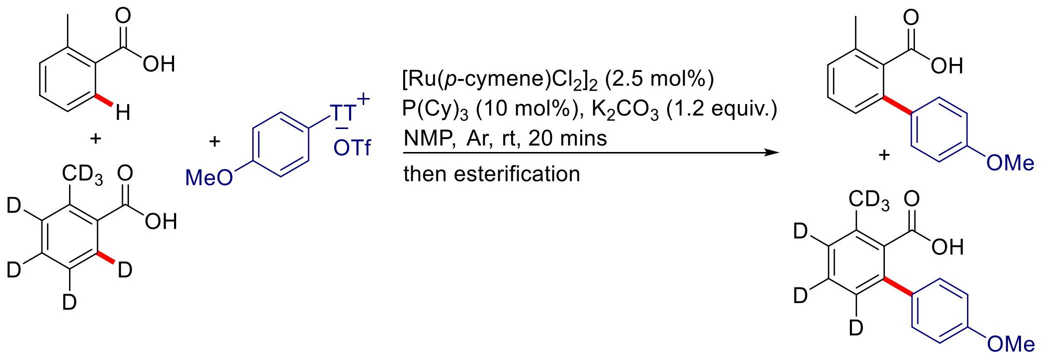

Scheme S2. Competition experiments for KIE measurement

Intermolecular KIE competition experiments: An oven-dried vessel was charged with [Ru(*p*-cymene)Cl_2]$_2$ (3.2 mg, 5.0 μmol, 0.05 equiv.), K_2CO_3 (33.6 mg, 0.24 mmol, 2.4 equiv.), P(Cy)$_3$ (5.6 mg, 0.02 mmol, 0.2 equiv.), and *p*-methoxyphenyl thianthrenium salt (113.4 mg, 0.24 mmol, 2.4 equiv.), an equimolar mixture of 2-methylbenzoic acid (0.1 mmol, 14.1 mg, 1 equiv.) and 2-methylbenzoic acid-d_7 (0.1 mmol, 14.8 mg, 1 equiv.). Under exclusion of air, NMP (4 mL) was added via syringe. The resulting reaction mixture was stirred at rt for 20 minutes. K_2CO3 (83.0 mg, 0.60 mmol, 6 equiv.) and CH3I (141.9 mg, 1.0 mmol, 10 equiv.) were added and the mixtures were stirred at 60 °C for 2.5 h. the two reaction mixtures were combined, and brine (10 mL) was added and the resulting mixture was extracted with ethyl acetate (3×20 mL). The combined organic layers were dried over $MgSO_4$, filtered, and the volatiles were removed under reduced pressure. The residue was purified by column chromatography (SiO_2, Petroleum Ether: EtOAc = 10:1), affording the mixed product. KIE value (K_H/K_D = 5:1) was determined on the basis of ^{1}H NMR analysis.

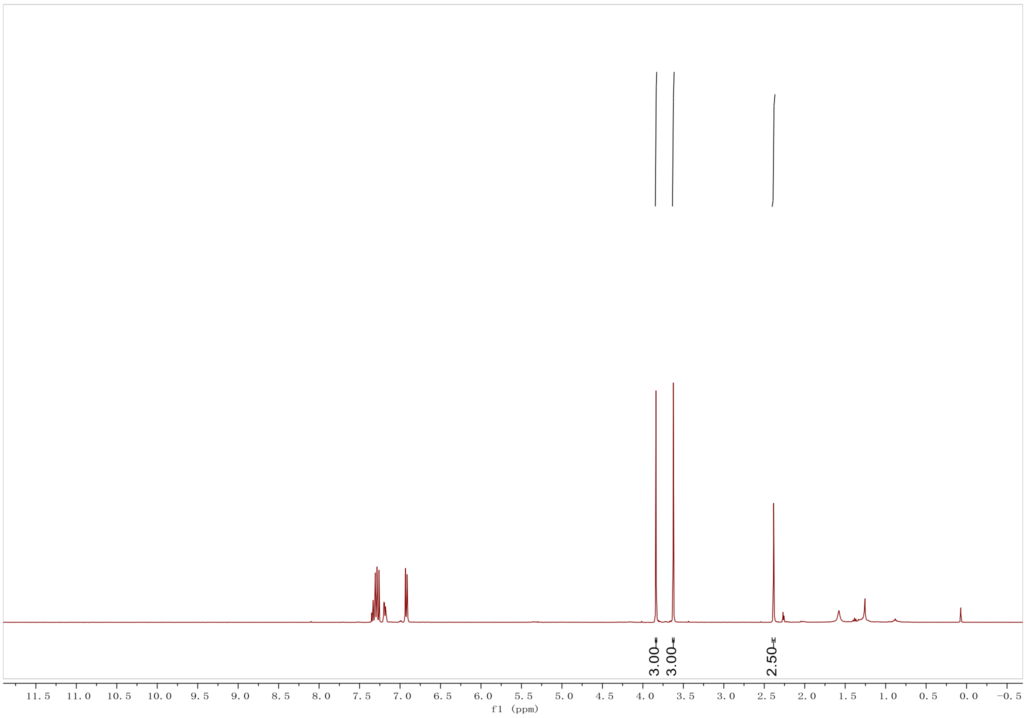

Figure S2. ^{1}H-NMR profile of competition experiments for KIE measurement.

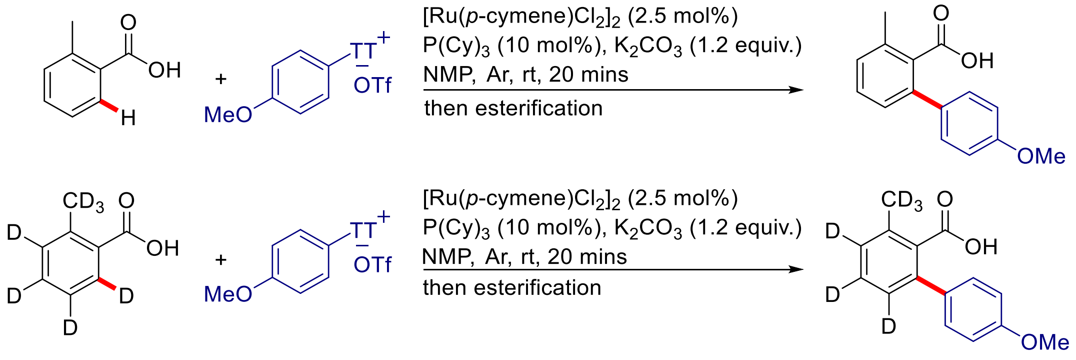

Scheme S3. Parallel experiments for KIE measurement

Parallel KIE experiments: Two oven-dried vessels were charged with $[Ru(p\text{-cymene})Cl_2]_2$ (1.6 mg, 2.50 μmol, 0.025 equiv.), K_2CO_3 (16.8 mg, 0.12 mmol, 1.2 equiv.), $P(Cy)_3$ (2.8 mg, 0.01 mmol, 0.1 equiv.), and *p*-methoxyphenyl thianthrenium salt (56.7 mg, 0.12 mmol, 1.2 equiv.). One vessel was charged with 2-methylbenzoic acid (0.10 mmol, 14.1 mg, 1 equiv.), the other one with 2-methylbenzoic acid-d_7 (0.10 mmol, 14.8 mg, 1.2 equiv). Under exclusion of air, NMP (2 mL) was added via syringe in both vessels. The two reaction mixtures were stirred side-by-side in the same stirrer at rt for 20 minutes. MeCN (2 mL), K_2CO_3 (41.5 mg, 0.30 mmol, 3 equiv.) and CH_3I (72.0 mg, 0.5 mmol, 5equiv.) were added and the mixtures were stirred at 60 °C for 2.5 h. These two mixtures were combined, brine (10 mL) was added and the resulting mixture was extracted with ethyl acetate (3×20 mL). The combined organic layers were dried over $MgSO_4$, filtered, and the volatiles

were removed under reduced pressure. The residue was purified by column chromatography (SiO_2, Petroleum Ether: EtOAc = 10:1), affording the mixed product. KIE value (K_H/K_D = 3.1:1) was determined on the basis of ^{1}H NMR analysis.

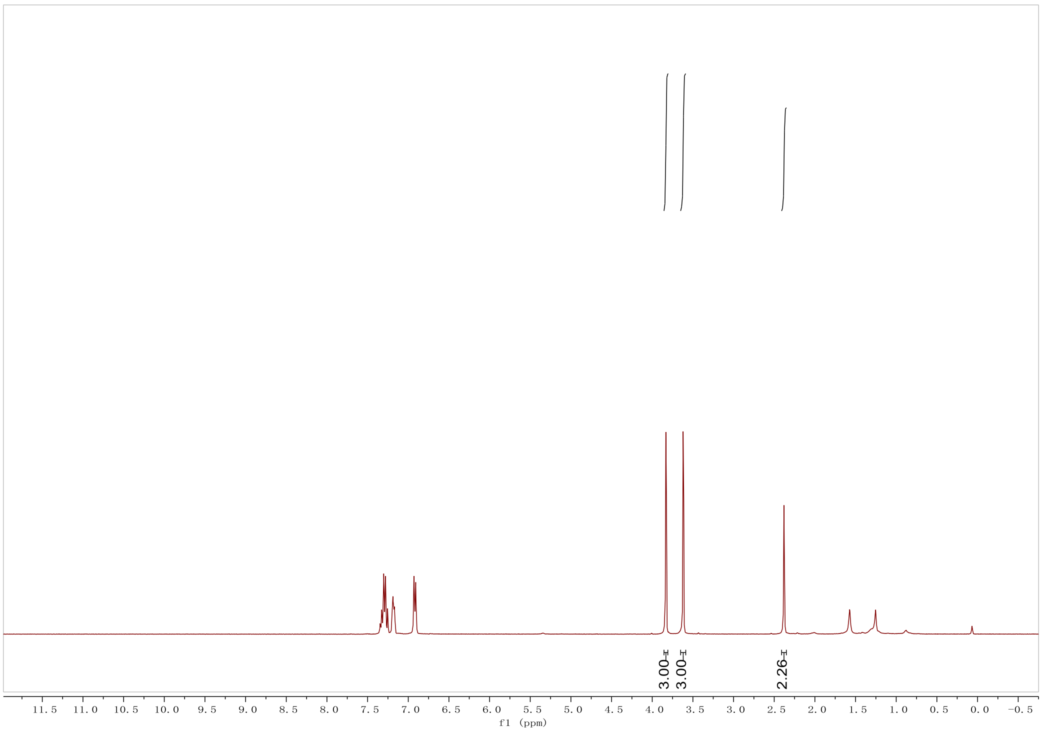

Figure S3. ^{1}H-NMR profile of parallel experiments for KIE measurement.

Competition experiments between electronically different benzoic acids:

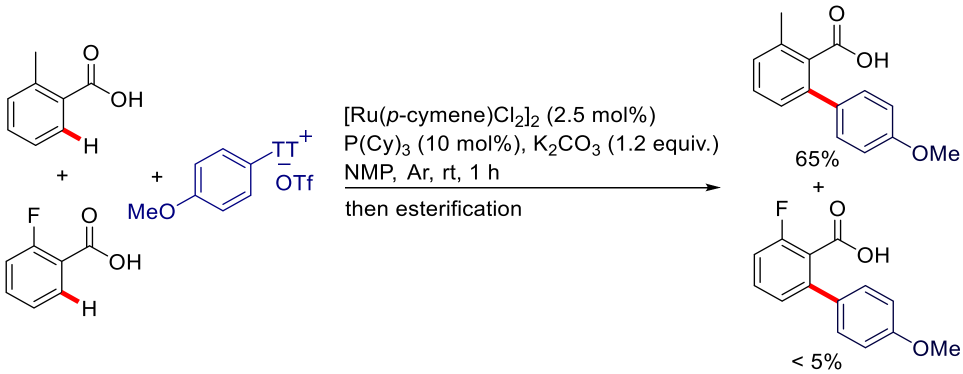

Scheme S4. Competition between electronically different benzoic acids

An oven-dried vessel was charged with [Ru(*p*-cymene)Cl_2]$_2$ (6.4 mg, 10.00 μmol, 0.05 equiv.), K_2CO_3 (67.2 mg, 0.48 mmol, 2.4 equiv.), P(Cy)$_3$ (11.2 mg, 0.04 mmol, 0.2 equiv.), and *p*-methoxyphenyl thianthrenium salt (226.8 mg, 0.48 mmol, 2.4 equiv.), an equimolar mixture of 2-methylbenzoic acid (28.2 mg, 0.2 mmol, 1 equiv.) and 2-

fluorobenzoic acid (28.0 mg, 0.2 mmol, 1 equiv.). Under exclusion of air, NMP (8 mL) was added via syringe. The resulting reaction mixture was stirred at rt for 1 h. MeCN (2 mL), K_2CO_3 (168 mg, 1.2 mmol, 6 equiv.) and CH_3I (273.6 mg, 2.0 mmol, 10 equiv.) were added and the mixtures were stirred at 60 °C for 2.5 h. Brine (20 mL) was added and the resulting mixture was extracted with ethyl acetate (3×20 mL). The combined organic layers were dried over $MgSO_4$, filtered, and the volatiles were removed under reduced pressure. The residue was purified by column chromatography (SiO_2, Petroleum Ether: EtOAc = 10:1), affording the corresponding products.

Parallel experiments between electronically different benzoic acids:

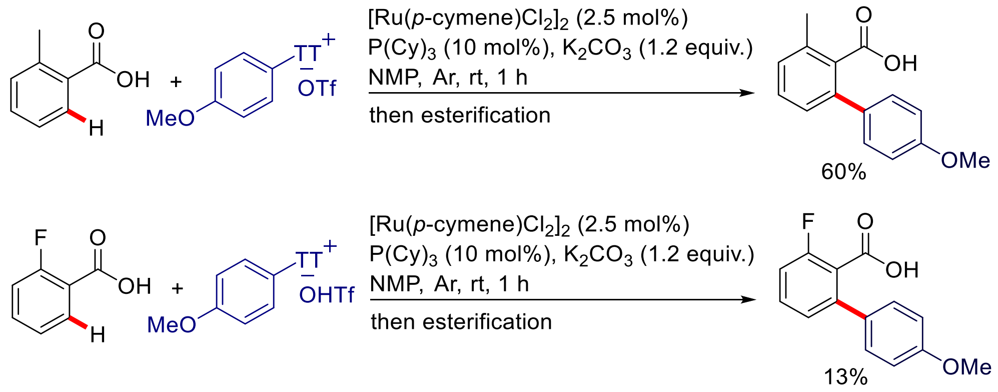

Scheme S5. Competition between electronically different benzoic acids

Two oven-dried vessels were charged with $[Ru(p\text{-cymene})Cl_2]_2$ (3.2 mg, 5.0 μmol, 0.025 equiv.), K_2CO_3 (33.6 mg, 0.24 mmol, 1.2 equiv.), $P(Cy)_3$ (5.6 mg, 0.02 mmol, 0.1 equiv.), and *p*-methoxyphenyl thianthrenium salt (113.4 mg, 0.24 mmol, 1.2 equiv.). One vessel was charged with 2-methylbenzoic acid (28.2 mg, 0.2 mmol, 1 equiv.), the other one with 2-fluorobenzoic acid (28.0 mg, 0.2 mmol, 1 equiv.). Under exclusion of air, NMP (4 mL) was added via syringe in both vessels. The two reaction mixtures were stirred side-by-side in the same stirrer at rt for 1 h. MeCN (2 mL), K_2CO_3 (83.0 mg, 0.6 mmol, 3 equiv.) and CH_3I (141.9 mg, 1.0 mmol, 5 equiv.) were added and the mixtures were stirred at 60 °C for 2.5 h. These two mixtures were combined, brine (20 mL) was added and the resulting mixture was extracted with ethyl acetate (3×20 mL). The combined organic layers were dried over $MgSO_4$, filtered, and the volatiles were removed under reduced pressure. The residue was purified by column chromatography (SiO_2, Petroleum Ether: EtOAc = 10:1), affording the corresponding products.

Competition experiments between electronically different arylthianthrenium salts:

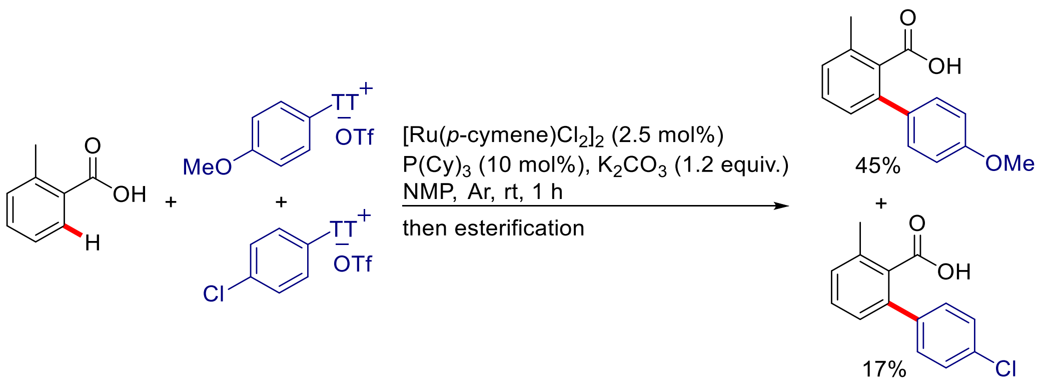

Scheme S6. Competition between electronically different benzoic acids

An oven-dried vessel was charged with [Ru(p-cymene)Cl_2]$_2$ (3.2 mg, 5.00 μmol, 0.025 equiv.), K_2CO_3 (33.6 mg, 0.24 mmol, 1.2 equiv.), P(Cy)$_3$ (5.6 mg, 0.02 mmol, 0.1 equiv.), 2-methylbenzoic acid (28.2 mg, 0.20 mmol, 1 equiv.), and an equimolar mixture of *p*-methoxyphenyl thianthrenium salt (113.4 mg, 0.24 mmol,1.2 equiv.) and *p*-chlorophenyl thianthrenium salt (114.4 mg, 0.24 mmol, 1.2 equiv.). Under exclusion of air, NMP (4 mL) was added via syringe. The resulting reaction mixture was stirred at rt for 1 h. MeCN (2 mL), K_2CO_3 (83.0 mg, 0.60 mmol, 3 equiv.) and CH_3I (141.9 mg, 1.0 mmol, 5 equiv.) were added and the mixtures were stirred at 60 °C for 2.5 h. Brine (10 mL) was added and the resulting mixture was extracted with ethyl acetate (3×20 mL). The combined organic layers were dried over MgSO4, filtered, and the volatiles were removed under reduced pressure. The residue was purified by column chromatography (SiO_2, Petroleum Ether: EtOAc = 10:1), affording the corresponding products.

Parallel experiments between electronically different arylthianthrenium salts:

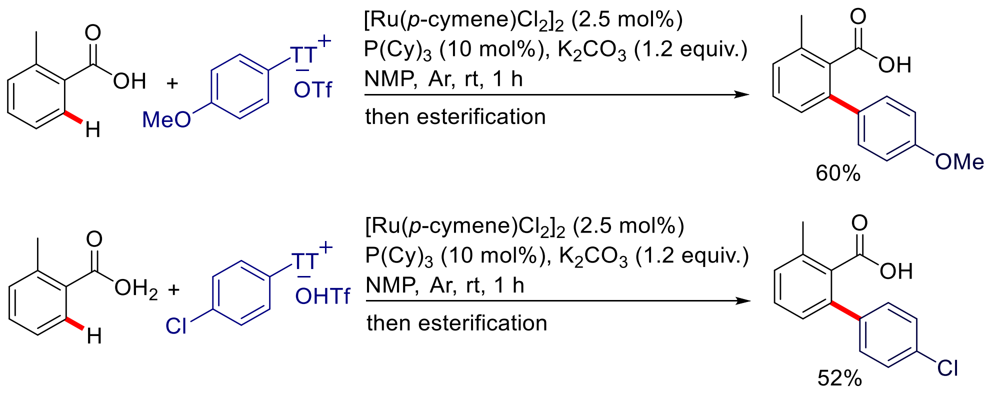

Scheme S7. Competition between electronically different benzoic acids

Two oven-dried vessels were charged with [Ru(*p*-cymene)Cl_2]$_2$ (3.2 mg, 5.0 μmol, 0.025 equiv.), K_2CO_3 (33.6 mg, 0.24 mmol, 1.2 equiv.), P(Cy)$_3$ (5.6 mg, 0.02 mmol, 0.1 equiv.), and 2-methylbenzoic acid (28.2 mg, 0.2 mmol, 1 equiv.), One vessel was charged with *p*-methoxyphenyl thianthrenium salt (113.4 mg, 0.24 mmol, 1.2 equiv.), the other one with 2 *p*-chlorophenyl thianthrenium salt (114.4 mg, 0.24 mmol, 1.2 equiv.). Under exclusion of air, NMP (4 mL) was added via syringe in both vessels. The two reaction mixtures were stirred side-by-side in the same stirrer at rt for 1 h. MeCN (2 mL), K_2CO_3 (83.0 mg, 0.60 mmol, 3 equiv.) and CH_3I (141.9 mg, 1.0 mmol, 5 equiv.) were added and the mixtures were stirred at 60 °C for 2.5 h. These two mixtures were combined, brine (20 mL) was added and the resulting mixture was extracted with ethyl acetate (3×20 mL). The combined organic layers were dried over $MgSO_4$, filtered, and the volatiles were removed under reduced pressure. The residue was purified by column chromatography (SiO_2, Petroleum Ether: EtOAc = 10:1), affording the corresponding products.

Control experiments of arylthianthrenium salt with [Ru(*p*-cymene)Cl_2]$_2$/PCy_3:

^{31}P-NMR Study: [Ru (*p*-cymene)Cl_2]$_2$ (0.075 mmol) and PCy_3/PCy_3 oxide (0.15 mmol) were dissolved in NMP (2 mL). A first ^{31}P-NMR was taken after 10 minutes using the phosphine oxide as internal standard. The spectrum corresponds to that of (*p*-cymene)bis(tricyclohexylphosphine)ruthenium(II), no changes were observed after 1 h. When adding a solution of the thianthrenium salt (0.15 mmol) in NMP (1.5 mL) to the reaction mixture, no new signals were observed after stirring for several hours (Figure S4).

^{31}P NMR was measured on a Spinsolve Benchtop NMR-80-ultra (MAGRITEK) spectrometers at 25 °C instrument.

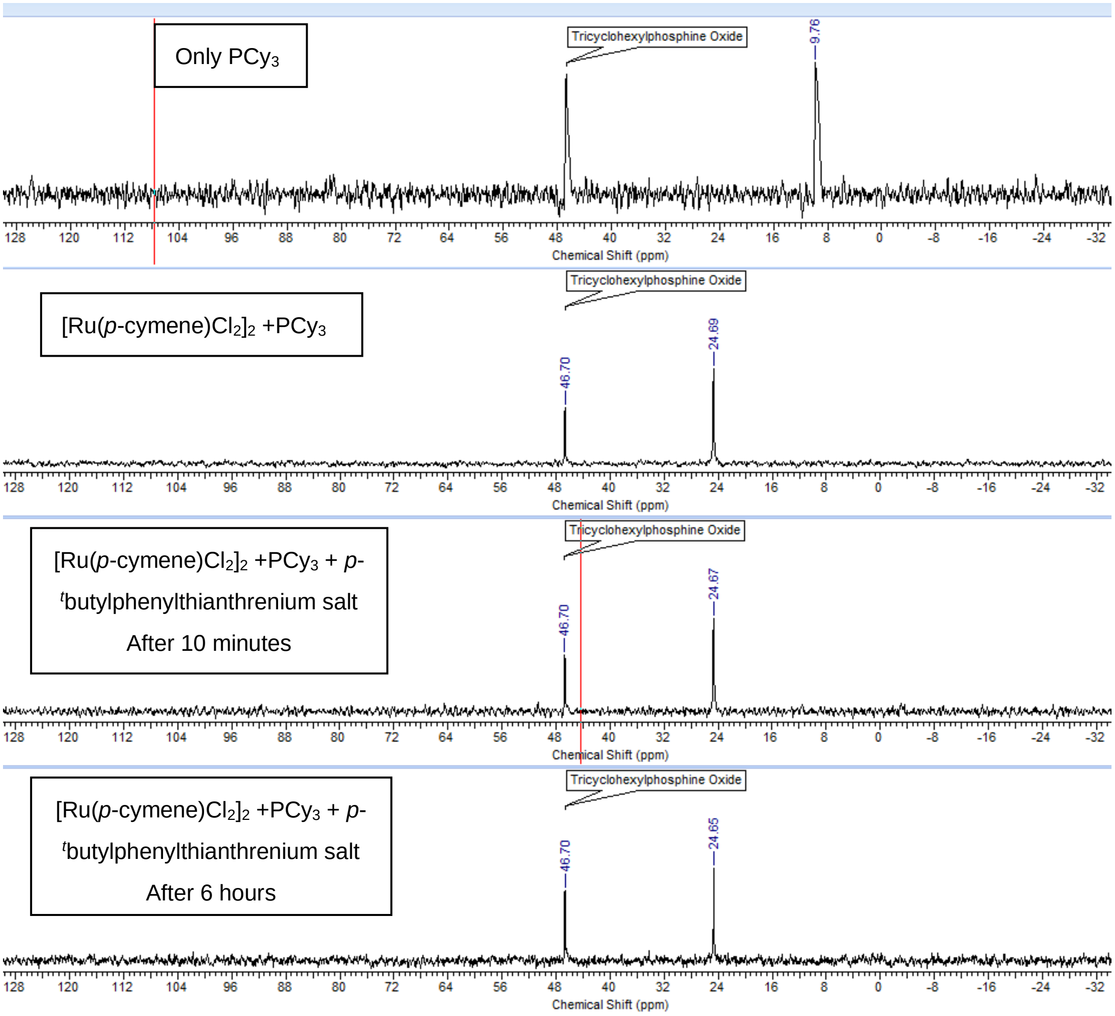

Figure S4. ^{31}P-NMR profile of the control experiments.

ESI-MS Study: The product mixture obtained this way was also analyzed by mass spectroscopy (ESI) using acetonitrile/water (0.1% formic acid) as additional solvent. Samples were prepared by diluting the mixture in acetonitrile (ca. 1 mg/mL) and filtration through a PTFE syringe filter. The evaluation and calculation of MS spectra were performed with the MassLynx software. The signals were consistent with the ones expected for (p-cymene)bis(tricyclohexylphosphine)ruthenium(II). No fragments could be assigned to an oxidative addition complex of the thianthrenium salt.

ESI MS were measured on a MS Waters Micromass ZQ instrument.

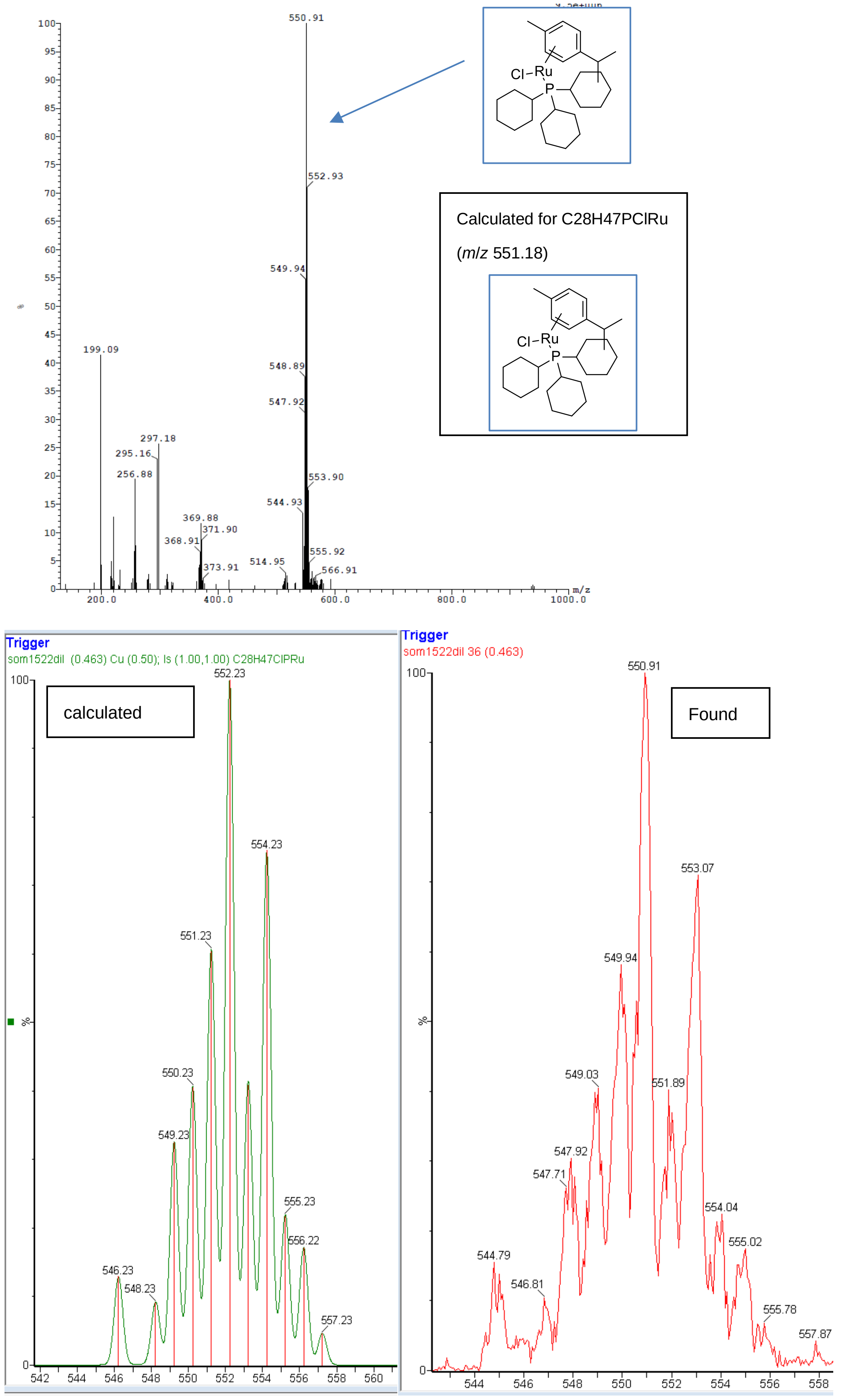

Figure S5. ESI-MS profile of the control experiments.

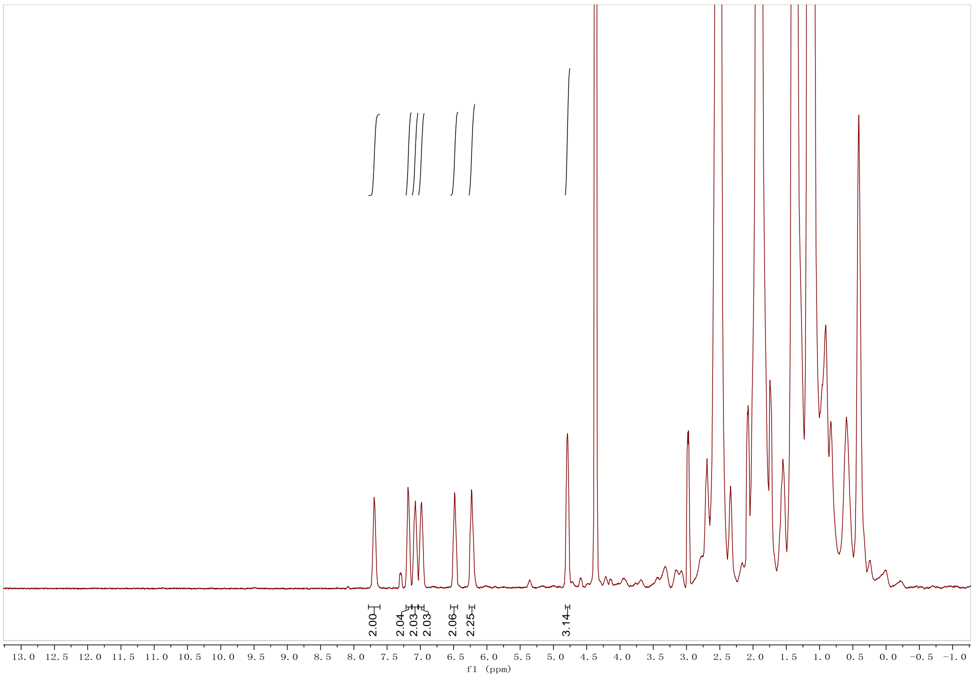

Figure S6. ^{1}H-NMR profile after adding the thianthrenium salt **2**.

Reaction from ruthenacycles:

Ruthenacycle **I** was prepared according to known literature procedure.[2]

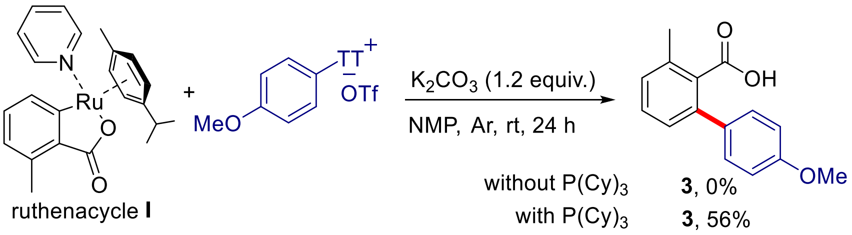

Scheme S8. Intermediate controlling experiments.

Experiments with stoichiometric ruthenacycle I: Two oven-dried vessel were charged with Ru ruthenacycle **I** (89.8 mg, 0.2 mmol, 1 equiv.), K_2CO_3 (33.6 mg, 0.24 mmol, 1.2 equiv.), and *p*-methoxyphenyl thianthrenium salt (113.4 mg, 0.24 mmol, 1.2 equiv.). One of the vessels was additionally charged with $P(Cy)_3$ (56 mg, 0.2 mmol, 1 equiv.), while the other was not. Under exclusion of air, NMP (4 mL) was added via syringe in both vessels. The resulting reaction mixture was stirred at rt for 24 h. MeCN (2 mL), K_2CO_3 (83.0 mg, 0.60 mmol, 3 equiv.) and CH_3I (141.9 mg, 1.0 mmol, 5 equiv.) were added and the mixtures were stirred at 60 °C for 2.5 h. Brine (10 mL) and tetradecane as an internal standard was added and the resulting mixture. The yield was determined by GC analysis.

Control experiments on oxidative addition without $P(Cy)_3$:

An oven-dried vessel was charged with ruthenacycle **I** (89.8 mg, 0.2 mmol, 1 equiv.), K_2CO_3 (33.6 mg, 0.24 mmol, 1.2 equiv.), and *p*-methoxyphenyl thianthrenium salt (113.4 mg, 0.24 mmol, 1.2 equiv.). Under exclusion of air, NMP (1 mL) was added via syringe in both vessels. The resulting reaction mixture was stirred at rt for 24 h. According to the ^{1}H NMR analysis, only unreacted ruthenacycle **I** is present (the number of hydrogen atoms corresponds exactly, though one H is shielded by the solvent NMP). Additionally, we almost quantitatively recovered both the arylthianthrenium salt and the ruthenacycle **I**, with no evidence of the oxidative addition intermediate. Figure S7 shows the unreacted ruthenacycle intermediate, where one hydrogen atom is overlapped by the solvent NMP.

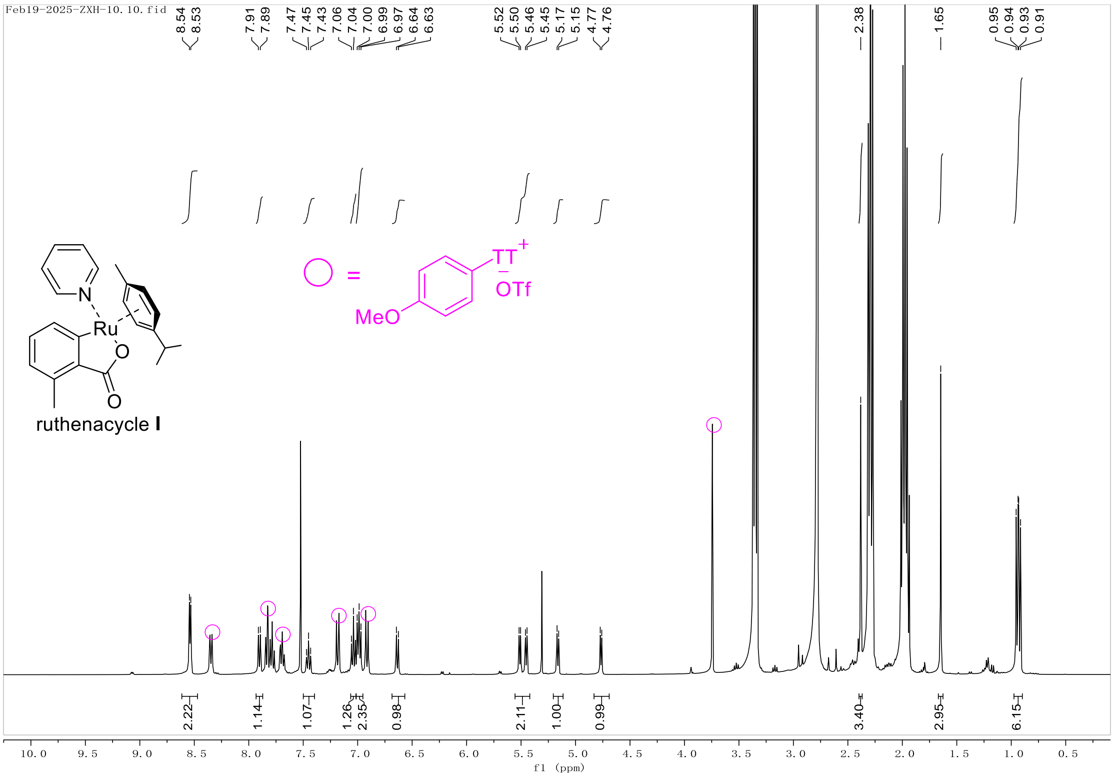

Figure S7. ^{1}H-NMR profiles after reaction of ruthenacycles with arylthianthrenium salts

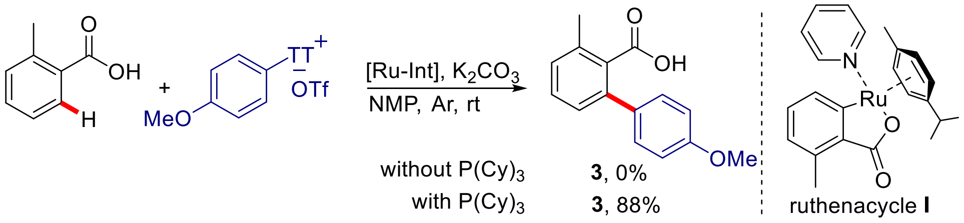

Scheme S9. Intermediate controlling experiments.

Experiments with catalytic ruthenacycle I: Two oven-dried vessel were charged with 2-methylbenzoic acid (0.20 mmol, 28.2 mg, 1 equiv.), ruthenacycle **I** (4.5 mg, 0.01 mmol, 0.05 equiv.), K_2CO_3 (33.6 mg, 0.24 mmol,

1.2 equiv.), and *p*-methoxyphenyl thianthrenium salt (113.4 mg, 0.24 mmol, 1.2 equiv.). One of the vessels was additionally charged with $P(Cy)_3$ (5.6 mg, 0.02 mmol), while the other was not. Under exclusion of air, NMP (4 mL) was added via syringe in both vessels. The resulting reaction mixture was stirred at rt for 24 h. MeCN (2 mL), K_2CO_3 (83.0 mg, 0.60 mmol, 3 equiv.) and CH_3I (141.9 mg, 1.0 mmol, 5 equiv.) were added and the mixtures were stirred at 60 °C for 2.5 h. Brine (10 mL) and tetradecane as an internal standard was added and the resulting mixture. The yield was determined by GC analysis.

Synthesis and Characterization of the Corresponding Products

General Procedure A: An oven-dried vessel was charged with arenecarboxylic acids (0.20 mmol, 1 equiv.), and arylthianthrenium salts (0.24 mmol, 1.2 equiv.), $[Ru(p\text{-cymene})Cl_2]_2$ (3.2 mg, 5.00 μmol, 0.025 equiv.), K_2CO_3 (33.6 mg, 0.24 mmol, 1.2 equiv.), $P(Cy)_3$ (5.6 mg, 0.02 mmol, 0.1 equiv.). Under exclusion of air, NMP (4 mL) was added via syringe. The resulting reaction mixture was stirred at rt (approximately 26-27 °C) for 24 h. MeCN (2 mL), K_2CO_3 (83.0 mg, 0.60 mmol, 3 equiv.) and CH_3I (141.9 mg, 1.0 mmol, 5 equiv.) were added and the mixtures were stirred at 60 °C for 2.5 h. Brine (10 mL) was added and the resulting mixture was extracted with ethyl acetate (3×20 mL). The combined organic layers were dried over $MgSO_4$, filtered, and the volatiles were removed under reduced pressure. The residue was purified by column chromatography (SiO_2, ethyl acetate/cyclohexane gradient), affording the corresponding products.

Methyl 4'-methoxy-3-methyl-[1,1'-biphenyl]-2-carboxylate (3)

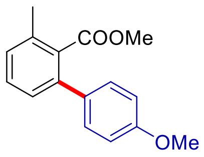

Compound **3** was prepared following the procedure **A**, starting from 2-methylbenzoic acid (28.2 mg, 0.20 mmol) and 5-(4-methoxyphenyl)-5*H*-thianthren-5-ium trifluoromethanesulfonate (113.4 mg, 0.24 mmol). After column chromatography on silica (Petroleum Ether: EtOAc = 10:1), **3** was afforded as colorless solid (49.2 mg, 95%).

M.P.: 69 – 70 °C.

^{1}H NMR (400 MHz, $CDCl_3$): δ 7.37 – 7.27 (m, 3H), 7.23 – 7.15 (m, 2H), 6.96 – 6.90 (m, 2H), 3.84 (s, 3H), 3.63 (s, 3H), 2.39 (s, 3H) ppm.

^{13}C NMR (101 MHz, $CDCl_3$): δ 170.5, 159.0, 139.6, 135.3, 133.3, 133.1, 129.33, 129.29, 128.7, 127.2, 113.7, 55.2, 51.9, 19.7 ppm.

Spectra data are consistent with the reported literature.[3]

Methyl 3,4'-dimethoxy-[1,1'-biphenyl]-2-carboxylate (4)

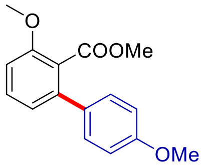

Compound **4** was prepared following the procedure **A**, starting from 2-methoxybenzoic acid (30.6 mg, 0.20 mmol) and 5-(4-methoxyphenyl)-5*H*-thianthren-5-ium trifluoromethanesulfonate (113.4 mg, 0.24 mmol). After column chromatography on silica (Petroleum Ether: EtOAc = 5:1), **4** was afforded as white solid (51.6 mg, 95%)

M.P.: 82 – 83 °C.

^{1}H NMR (400 MHz, $CDCl_3$): δ 7.41 – 7.36 (m, 1H), 7.35 – 7.30 (m, 2H), 6.98 – 6.95 (m, 1H), 6.94 – 6.89 (m, 3H), 3.87 (s, 3H), 3.83 (s, 3H), 3.67 (s, 3H) ppm.

^{13}C NMR (101 MHz, $CDCl_3$): δ 168.7, 159.1, 156.4, 140.7, 132.4, 130.4, 129.3, 122.9, 121.9, 113.7, 109.4, 56.0, 55.2, 52.1 ppm.

Spectra data are consistent with the reported literature.[3]

Methyl 4'-methoxy-3-phenoxy-[1,1'-biphenyl]-2-carboxylate (5)

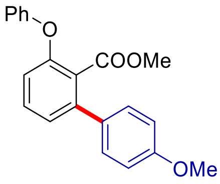

Compound **5** was prepared following the procedure **A**, starting from 2-phenoxybenzoic acid (43.8 mg, 0.20 mmol) and 5-(4-methoxyphenyl)-5*H*-thianthren-5-ium trifluoromethanesulfonate (113.4 mg, 0.24 mmol). After column chromatography on silica (Petroleum Ether: EtOAc = 10:1), **5** was afforded as yellow oil liquid (45.4 mg, 68%).

^{1}H NMR (400 MHz, $CDCl_3$): δ 7.40 – 7.32 (m, 5H), 7.15 – 7.06 (m, 4H), 6.98 – 6.93 (m, 2H), 6.89 – 6.85 (m, 1H), 3.85 (s, 3H), 3.64 (s, 3H) ppm.

^{13}C NMR (101 MHz, $CDCl_3$): δ 168.0, 159.3, 157.0, 154.4, 141.3, 132.1, 130.4, 129.7, 129.4, 125.6, 124.4, 123.6, 119.2, 116.9, 113.8, 55.0, 52.2 ppm.

Spectra data are consistent with the reported literature.[3]

Methyl 4'-methoxy-3-(trifluoromethoxy)-[1,1'-biphenyl]-2-carboxylate (6)

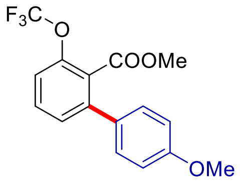

Compound **6** was prepared following the procedure **A**, starting from 2-(trifluoromethoxy)benzoic acid (42.0 mg, 0.20 mmol) and 5-(4-methoxyphenyl)-5*H*-thianthren-5-ium trifluoromethanesulfonate (113.4 mg, 0.24 mmol) with [Ru(p-cymene)Cl_2]$_2$ (6.2 mg, 0.01 mmol) and P(Cy)$_3$ (11.2 mg, 0.04 mmol). After column chromatography on silica (Petroleum Ether: EtOAc = 10:1), **6** was afforded as white oil liquid (39.2 mg, 60%).

^{1}H NMR (400 MHz, $CDCl_3$): δ 7.50 – 7.44 (m, 1H), 7.34 – 7.26 (m, 4H), 6.96 – 6.92 (m, 2H), 3.84 (s, 3H), 3.71 (s, 3H) ppm.

^{13}C NMR (101 MHz, $CDCl_3$) δ 166.6, 159.6, 146.0 (q, $J_{C\text{-}F}$ = 1.7 Hz), 141.9, 131.4, 130.5, 129.4, 128.0, 127.0, 120.4 (q, $J_{C\text{-}F}$ = 259.6 Hz), 118.7, 114.0, 55.3, 52.4 ppm.

HRMS (ESI-TOF) m/z: [M+H]$^+$ Calcd for $C_{16}H_{14}F_3O_4^+$ 327.0839; Found 327.0833.

Methyl 3-acetoxy-4'-methoxy-[1,1'-biphenyl]-2-carboxylate (7)

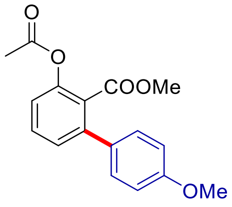

Compound **7** was prepared following the procedure **A**, starting from 2-acetoxybenzoic acid (36.2 mg, 0.20 mmol) and 5-(4-methoxyphenyl)-5*H*-thianthren-5-ium trifluoromethanesulfonate (113.4 mg, 0.24 mmol). After column chromatography on silica (Petroleum Ether: EtOAc = 5:1), **7** was afforded as yellow solid (42.6 mg, 71%).

M.P.: 95 – 96 °C.

^{1}H NMR (400 MHz, $CDCl_3$): δ 7.48 – 7.43 (m, 1H), 7.29 – 7.25 (m, 3H), 7.13 – 7.08 (m, 1H), 6.94 – 6.90 (m, 2H), 3.83 (s, 3H), 3.61 (s, 3H), 2.28 (s, 3H) ppm.

^{13}C NMR (101 MHz, $CDCl_3$): δ 169.1, 167.2, 159.3, 148.1, 141.8, 132.1, 130.5, 129.3, 127.4, 126.0, 121.3, 113.8, 55.2, 52.2, 20.8 ppm.

Spectra data are consistent with the reported literature.[3]

Methyl 4-methoxy-[1,1':3',1''-terphenyl]-2'-carboxylate (8)

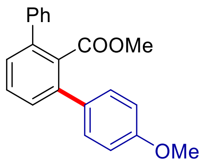

Compound **8** was prepared following the procedure **A**, starting from [1,1'-biphenyl]-2-carboxylic acid (39.8 mg, 0.20 mmol) and 5-(4-methoxyphenyl)-5*H*-thianthren-5-ium trifluoromethanesulfonate (113.4 mg, 0.24 mmol). After column chromatography on silica (Petroleum Ether: EtOAc = 10:1), **8** was afforded as yellow solid (60.4 mg, 92%).

M.P.: 87 – 88 °C.

^{1}H NMR (400 MHz, $CDCl_3$): δ 7.53 – 7.48 (m, 1H), 7.46 – 7.40 (m, 4H), 7.40 – 7.34 (m, 5H), 7.00 – 6.94 (m, 2H), 3.85 (s, 3H), 3.44 (s, 3H) ppm.

^{13}C NMR (101 MHz, $CDCl_3$): δ 170.0, 159.1, 140.5, 140.2, 139.8, 132.8, 132.7, 129.5, 129.3, 128.8, 128.4, 128.3, 128.2, 127.4, 113.7, 55.2, 51.7 ppm.

Spectra data are consistent with the reported literature.[3]

Methyl 3-benzoyl-4'-methoxy-[1,1'-biphenyl]-2-carboxylate (9)

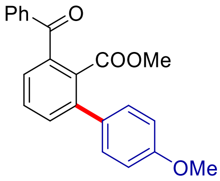

Compound **9** was prepared following the procedure **A**, starting from 2-benzoylbenzoic acid (46.2 mg, 0.20 mmol) and 5-(4-methoxyphenyl)-5*H*-thianthren-5-ium trifluoromethanesulfonate (113.4 mg, 0.24 mmol). After column chromatography on silica (Petroleum Ether: EtOAc = 5:1), **9** was afforded as yellow solid (30.4 mg, 44%).

M.P.: 117 – 118 °C.

^{1}H NMR (400 MHz, $CDCl_3$): δ 7.87 – 7.82 (m, 2H), 7.62 – 7.51 (m, 3H), 7.50 – 7.45 (m, 3H), 7.33 – 7.29 (m, 2H), 6.97 – 6.93 (m, 2H), 3.84 (s, 3H), 3.45 (s, 3H) ppm.

^{13}C NMR (101 MHz, $CDCl_3$): δ 196.4, 168.9, 159.3, 141.2, 138.4, 136.8, 133.1, 132.8, 132.7, 132.1, 130.0, 129.5, 129.2, 128.3, 127.8, 113.8, 55.2, 52.1 ppm.

Spectra data are consistent with the reported literature.[3]

Dimethyl 4'-methoxy-[1,1'-biphenyl]-2,3-dicarboxylate (10)

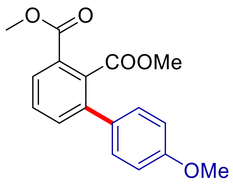

Compound **10** was prepared following the procedure **A**, starting from 2-(methoxycarbonyl)benzoic acid (38.0 mg, 0.20 mmol) and 5-(4-methoxyphenyl)-5*H*-thianthren-5-ium trifluoromethanesulfonate (113.4 mg, 0.24 mmol). After column chromatography on silica (Petroleum Ether: EtOAc = 10:1), **10** was afforded as yellow solid (31.2 mg, 52%).

M.P.: 118 – 119 °C.

^{1}H NMR (400 MHz, $CDCl_3$): δ 8.00 – 7.93 (m, 1H), 7.55 – 7.47 (m, 2H), 7.32 – 7.27 (m, 2H), 6.95 – 6.90 (m, 2H), 3.90 (s, 3H), 3.83 (s, 3H), 3.70 (s, 3H) ppm.

^{13}C NMR (101 MHz, $CDCl_3$): δ 169.4, 166.2, 159.3, 140.2, 134.7, 134.3, 131.5, 129.7, 129.0, 128.5, 128.0, 113.7, 55.2, 52.5, 52.3 ppm.

Spectra data are consistent with the reported literature.[3]

Methyl 3-fluoro-4'-methoxy-[1,1'-biphenyl]-2-carboxylate (11)

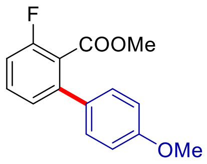

Compound **11** was prepared following the procedure **A**, starting from 2-fluorobenzoic acid (28.0 mg, 0.20 mmol) and 5-(4-methoxyphenyl)-5*H*-thianthren-5-ium trifluoromethanesulfonate (113.4 mg, 0.24 mmol) with [Ru(p-

cymene)Cl_2]$_2$ (6.2 mg, 0.01 mmol) and P(Cy)$_3$ (11.2 mg, 0.04 mmol). After column chromatography on silica (Petroleum Ether: EtOAc = 15:1), **11** was afforded as colorless oil liquid (35.8 mg, 69%).

^{1}H NMR (400 MHz, $CDCl_3$): δ 7.46 – 7.38 (m, 1H), 7.34 – 7.27 (m, 2H), 7.19 – 7.14 (m, 1H), 7.12 – 7.04 (m, 1H), 6.97 – 6.90 (m, 2H), 3.84 (s, 3H), 3.71 (s, 3H) ppm.

^{13}C NMR (101 MHz, $CDCl_3$): δ 166.4, 159.7 (d, J_{C-F} = 252.5 Hz), 159.5, 142.1 (d, J_{C-F} = 3.0 Hz), 131.6 (d, J_{C-F} = 3.0 Hz), 131.2 (d, J_{C-F} = 10.0 Hz), 129.3, 125.4 (d, J_{C-F} = 3.0 Hz),121.3 (d, J_{C-F} = 16.7 Hz), 114.1 (d, J_{C-F} = 21.6 Hz), 113.9 , 55.3, 52.4 ppm.

Spectra data are consistent with the reported literature.[3]

Methyl 4'-methoxy-4-methyl-[1,1'-biphenyl]-2-carboxylate (12)

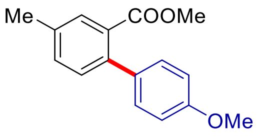

Compound **12** was prepared following the procedure **A**, starting from 3-methylbenzoic acid (27.6 mg, 0.20 mmol) and 5-(4-methoxyphenyl)-5*H*-thianthren-5-ium trifluoromethanesulfonate (113.4 mg, 0.24 mmol). After column chromatography on silica (Petroleum Ether: EtOAc = 10:1), **12** was afforded as white solid (48.7 mg, 95%). mmol). After column chromatography on silica (Petroleum Ether: EtOAc = 10:1), **12** was afforded as yellow oil liquid (31.2 mg, 52%).

^{1}H NMR (400 MHz, $CDCl_3$): δ 7.65 – 7.56 (m, 1H), 7.33 – 7.29 (m, 1H), 7.28 – 7.21 (m, 3H), 6.97 – 6.89 (m, 2H), 3.84 (s, 3H), 3.66 (s, 3H), 2.41 (s, 3H) ppm.

^{13}C NMR (101 MHz, $CDCl_3$): δ 169.5, 158.7, 139.1, 136.5, 133.5, 131.9, 130.5, 130.1, 129.4, 113.4, 55.1, 51.8, 20.8 ppm.

Spectra data are consistent with the reported literature.[3]

Methyl 4-(dimethylamino)-4'-methoxy-[1,1'-biphenyl]-2-carboxylate (13)

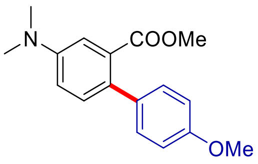

Compound **13** was prepared following the procedure **A**, starting from 3-(dimethylamino)benzoic acid (34.0 mg, 0.20 mmol) and 5-(4-methoxyphenyl)-5*H*-thianthren-5-ium trifluoromethanesulfonate (113.4 mg, 0.24 mmol). After column chromatography on silica (Petroleum Ether: EtOAc = 10:1), **13** was afforded as yellow solid (26.2 mg, 46%).

M.P.: 75 – 78 °C.

^{1}H NMR (400 MHz, $CDCl_3$): δ 7.26 – 7.20 (m, 3H), 7.13 – 7.08 (m, 1H), 6.94 – 6.89 (m, 2H), 6.89 – 6.84 (m, 1H), 3.84 (s, 3H), 3.66 (s, 3H), 3.01 (s, 6H) ppm.

^{13}C NMR (101 MHz, $CDCl_3$): δ 170.2, 158.3, 149.1, 133.9, 131.3, 131.3, 129.7, 129.3, 115.1, 113.4, 113.1, 55.2, 51.9, 40.5 ppm.

HRMS (ESI-TOF) m/z: $[M+H]^+$ Calcd for $C_{17}H_{20}NO_3^+$ 286.1438; Found 286.1428.

Methyl 4,4'-dimethoxy-[1,1'-biphenyl]-2-carboxylate (14)

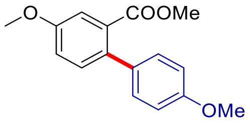

Compound **14** was prepared following the procedure **A**, starting from 3-methoxybenzoic acid (31.0 mg, 0.20 mmol) and 5-(4-methoxyphenyl)-5*H*-thianthren-5-ium trifluoromethanesulfonate (113.4 mg, 0.24 mmol). After column chromatography on silica (Petroleum Ether: EtOAc = 10:1), **14** was afforded as yellow oil liquid (43.0 mg, 79%).

^{1}H NMR (400 MHz, $CDCl_3$): δ 7.24 - 7.21 (m, 1H), 7.20 - 7.16 (m, 1H), 7.15 – 7.09 (m, 2H), 6.98 - 6.94 (m, 1H), 6.86 – 6.80 (m, 2H), 3.77 (s, 3H), 3.74 (s, 3H), 3.57 (s, 3H) ppm.

^{13}C NMR (101 MHz, $CDCl_3$): δ 169.1, 158.6, 158.2, 134.5, 133.3, 131.8, 131.5, 129.4, 117.4, 114.2, 113.4, 55.5, 55.2, 52.0 ppm.

Spectra data are consistent with the reported literature.[4]

Methyl 4-(((*tert*-butoxycarbonyl)amino)methyl)-4'-methoxy-[1,1'-biphenyl]-2-carboxylate (15)

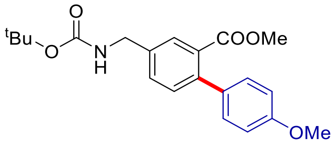

Compound **15** was prepared following the procedure **A**, starting from 3-(((*tert*-butoxycarbonyl)amino)methyl)benzoic acid (53.0 mg, 0.20 mmol) and 5-(4-methoxyphenyl)-5*H*-thianthren-5-ium trifluoromethanesulfonate (113.4 mg, 0.24 mmol). After column chromatography on silica (Petroleum Ether: EtOAc = 3:1), **15** was afforded as yellow oil liquid (60.8 mg, 82%).

^{1}H NMR (400 MHz, $CDCl_3$): δ 7.68 (s, 1H), 7.45 – 7.38 (m, 1H), 7.33 – 7.28 (m, 1H), 7.25 – 7.16 (m, 2H), 6.94 – 6.88 (m, 2H), 5.07 (s, 1H), 4.40 – 4.28 (m, 2H), 3.82 (s, 3H), 3.65 (s, 3H), 1.46 (s, 9H) ppm.

^{13}C NMR (101 MHz, $CDCl_3$): δ 169.2, 158.9, 155.9, 140.9, 137.7, 133.2, 131.0, 130.9, 130.2, 129.4, 128.5, 113.5, 79.6, 55.2, 51.9, 43.9, 28.3 ppm.

HRMS (ESI-TOF) m/z: $[M+H]^+$ Calcd for $C_{21}H_{26}NO_5^+$ 372.1805; Found 372.1800.

Methyl 4-methoxy-[1,1':4',1''-terphenyl]-2'-carboxylate (16)

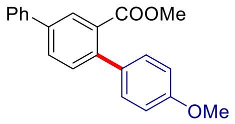

Compound **16** was prepared following the procedure **A**, starting from [1,1'-biphenyl]-3-carboxylic acid (39.6 mg, 0.20 mmol) and 5-(4-methoxyphenyl)-5*H*-thianthren-5-ium trifluoromethanesulfonate (113.4 mg, 0.24 mmol). After column chromatography on silica (Petroleum Ether: EtOAc = 10:1), **16** was afforded as white solid (42.0 mg, 66%).

M.P.: 102 – 104 °C.

^{1}H NMR (400 MHz, $CDCl_3$): δ 8.08 – 8.03 (m, 1H), 7.77 – 7.72 (m, 1H), 7.69 – 7.64 (m, 2H), 7.51 – 7.44 (m, 3H), 7.43 – 7.36 (m, 1H), 7.35 – 7.29 (m, 2H), 7.01 – 6.95 (m, 2H), 3.87 (s, 3H), 3.72 (s, 3H) ppm.

^{13}C NMR (101 MHz, $CDCl_3$): δ 169.4, 159.0, 140.8, 139.72, 139.71, 133.2, 131.20, 131.17, 129.6, 129.4, 128.9, 128.3, 127.7, 127.0, 113.6, 55.2, 52.0 ppm.

HRMS (ESI-TOF) m/z: $[M+H]^+$ Calcd for $C_{21}H_{19}O_3^+$ 319.1329; Found 319.1320.

Dimethyl 4'-methoxy-[1,1'-biphenyl]-2,4-dicarboxylate (17)

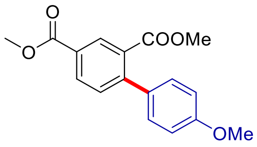

Compound **17** was prepared following the procedure **A**, starting from 3-(methoxycarbonyl)benzoic acid (36.8 mg, 0.20 mmol) and 5-(4-methoxyphenyl)-5*H*-thianthren-5-ium trifluoromethanesulfonate (113.4 mg, 0.24 mmol). After column chromatography on silica (Petroleum Ether: EtOAc = 5:1), **17** was afforded as yellow oil liquid (29.4 mg, 50%).

^{1}H NMR (400 MHz, $CDCl_3$): δ 8.46 – 8.40 (m, 1H), 8.17 – 8.11 (m, 1H), 7.46 – 7.41 (m, 1H), 7.27 – 7.24 (m, 2H), 6.96 – 6.92 (m, 2H), 3.94 (s, 3H), 3.84 (s, 3H), 3.70 (s, 3H) ppm.

^{13}C NMR (101 MHz, $CDCl_3$): δ 168.5, 166.1, 159.5, 146.3, 132.4, 131.9, 131.04, 131.00, 130.9, 129.4, 128.6, 113.7, 55.2, 52.3, 52.2 ppm.

Spectra data are consistent with the reported literature.[3]

Methyl 4-acetyl-4'-methoxy-[1,1'-biphenyl]-2-carboxylate (18)

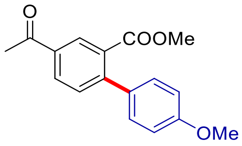

Compound **18** was prepared following the procedure **A**, starting from 3-acetylbenzoic acid (33.2 mg, 0.20 mmol) and 5-(4-methoxyphenyl)-5*H*-thianthren-5-ium trifluoromethanesulfonate (113.4 mg, 0.24 mmol) with [Ru(p-cymene)Cl_2]$_2$ (6.2 mg, 0.01 mmol) and P(Cy)$_3$ (11.2 mg, 0.04 mmol). After column chromatography on silica (Petroleum Ether: EtOAc = 10:1), **18** was afforded as yellow oil liquid (30.6 mg, 54%).

^{1}H NMR (400 MHz, $CDCl_3$): δ 8.31 – 8.22 (m, 1H), 8.03 – 7.97 (m, 1H), 7.41 – 7.37 (m, 1H), 7.21 – 7.16 (m, 2H), 6.91 – 6.85 (m, 2H), 3.77 (s, 3H), 3.63 (s, 3H), 2.57 (s, 3H) ppm.

^{13}C NMR (101 MHz, $CDCl_3$): δ 196.8, 168.7, 159.5, 146.4, 135.3, 132.3, 131.1, 131.0, 130.5, 130.0, 129.4, 113.7, 55.2, 52.2, 26.6 ppm.

Spectra data are consistent with the reported literature.[5]

Methyl 4'-methoxy-[1,1'-biphenyl]-2-carboxylate (19)

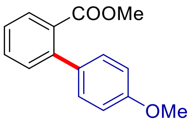

Compound **19** were prepared following the procedure **A**, starting from benzoic acid (24.6 mg, 0.20 mmol) and 5-(4-methoxyphenyl)-5*H*-thianthren-5-ium trifluoromethanesulfonate (113.4 mg, 0.24 mmol). After column chromatography on silica (Petroleum Ether: EtOAc = 10:1), **19** was afforded as light yellow oil liquid (10.6 mg, 22%).

^{1}H NMR (400 MHz, $CDCl_3$): δ 7.75 – 7.67 (m, 1H), 7.47 – 7.39 (m, 1H), 7.34 – 7.25 (m, 2H), 7.20 – 7.14 (m, 2H), 6.90 – 6.82 (m, 2H), 3.77 (s, 3H), 3.59 (s, 3H) ppm.

^{13}C NMR (101 MHz, $CDCl_3$): δ 169.4, 159.0, 142.0, 133.6, 131.2, 130.8, 130.7, 129.7, 129.4, 126.8, 113.5, 55.2, 52.0 ppm.

Spectra data are consistent with the reported literature.[5]

Methyl 4,4''-dimethoxy-[1,1':3',1''-terphenyl]-2'-carboxylate (19’)

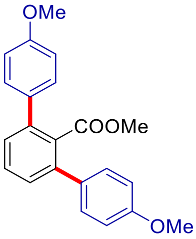

Compound **19’** were prepared following the procedure **A**, starting from benzoic acid (24.6 mg, 0.20 mmol) and 5-(4-methoxyphenyl)-5*H*-thianthren-5-ium trifluoromethanesulfonate (113.4 mg, 0.24 mmol). After column chromatography on silica (Petroleum Ether: EtOAc = 10:1), **19’** was afforded as white solid (23.6 mg, 34%).

M.P.: 112 – 113 °C.

^{1}H NMR (400 MHz, $CDCl_3$): δ 7.49 – 7.44 (m, 1H), 7.37 – 7.32 (m, 6H), 6.96 – 6.92 (m, 4H), 3.84 (s, 6H), 3.44 (s, 3H) ppm.

^{13}C NMR (101 MHz, $CDCl_3$): δ 170.2, 159.1, 139.8, 132.9, 132.7, 129.5, 129.2, 128.5, 113.7, 55.2, 51.8 ppm.

Spectra data are consistent with the reported literature.[5]

Methyl 4',5-dimethoxy-3-methyl-[1,1'-biphenyl]-2-carboxylate (20)

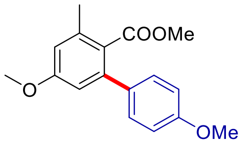

Compound **20** was prepared following the procedure **A**, starting from 4-methoxy-2-methylbenzoic acid (33.2 mg, 0.20 mmol) and 5-(4-methoxyphenyl)-5*H*-thianthren-5-ium trifluoromethanesulfonate (113.4 mg, 0.24 mmol). After column chromatography on silica (Petroleum Ether: EtOAc = 10:1), **20** was afforded as colorless oil liquid (49.8 mg, 87%).

^{1}H NMR (400 MHz, $CDCl_3$): δ 7.32 – 7.27 (m, 2H), 6.95 – 6.90 (m, 2H), 6.72 (s, 2H), 3.84 – 3.81 (m, 6H), 3.58 (s, 3H), 2.38 (s, 3H) ppm.

^{13}C NMR (101 MHz, $CDCl_3$): δ 170.4, 159.9, 159., 141.83, 137.7, 133.5, 129.1, 125.8, 114.3, 113.7, 112.5, 55.24, 55.20, 51.7, 20.1 ppm.

HRMS (ESI-TOF) m/z: $[M+H]^+$ Calcd for $C_{17}H_{19}O_4^+$ 287.1278; Found 287.1271.

Methyl 3,4',5-trimethoxy-[1,1'-biphenyl]-2-carboxylate (21)

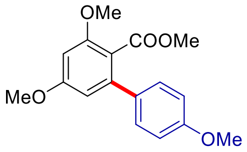

Compound **21** was prepared following the procedure **A**, starting from 2,4-dimethoxybenzoic acid (36.6 mg, 0.20 mmol) and 5-(4-methoxyphenyl)-5*H*-thianthren-5-ium trifluoromethanesulfonate (113.4 mg, 0.24 mmol). After column chromatography on silica (Petroleum Ether: EtOAc = 10:1), **21** was afforded as yellow oil liquid (52.6 mg, 87%).

^{1}H NMR (400 MHz, $CDCl_3$): δ 7.33 – 7.29 (m, 2H), 6.93 – 6.89 (m, 2H), 6.47 – 6.44 (m, 2H), 3.84 – 3.81 (m, 9H), 3.61 (s, 3H) ppm.

^{13}C NMR (101 MHz, $CDCl_3$): δ 168.6, 161.2, 159.1, 158.0, 142.2, 132.8, 129.1, 115.9, 113.7, 106.0, 97.1, 55.9, 55.4, 55.2, 52.0 ppm.

Spectra data are consistent with the reported literature.[3]

Methyl 4,4'-dimethoxy-5-(trifluoromethyl)-[1,1'-biphenyl]-2-carboxylate (22)

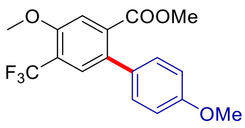

Compound **22** was prepared following the procedure **A**, starting from 3-methoxy-4-(trifluoromethyl)benzoic acid (44.6 mg, 0.20 mmol) and 5-(4-methoxyphenyl)-5*H*-thianthren-5-ium trifluoromethanesulfonate (113.4 mg, 0.24 mmol) with [Ru(p-cymene)Cl_2]$_2$ (6.2 mg, 0.01 mmol) and P(Cy)$_3$ (11.2 mg, 0.04 mmol). After column chromatography on silica (Petroleum Ether: EtOAc = 10:1), **22** was afforded as yellow oil liquid (46.2 mg, 68%).

^{1}H NMR (400 MHz, $CDCl_3$): δ 7.57 (s, 1H), 7.37 (s, 1H), 7.24 – 7.17 (m, 2H), 6.96 – 6.91 (m, 2H), 3.97 (s, 3H), 3.85 (s, 3H), 3.68 (s, 3H) ppm.

^{13}C NMR (101 MHz, $CDCl_3$): δ 168.4, 159.1, 156.0 (q, $J_{C\text{-}F}$ = 1.0 Hz), 135.0, 133.8, 132.0, 129.5 (q, $J_{C\text{-}F}$ = 5.1 Hz), 129.4, 123.0 (q, $J_{C\text{-}F}$ = 273.7 Hz), 120.9 (q, $J_{C\text{-}F}$ = 31.3 Hz), 113.7, 112.9, 56.3, 55.3, 52.3 ppm.

HRMS (ESI-TOF) m/z: $[M+H]^+$ Calcd for $C_{17}H_{16}FO_4^+$ 341.0995; Found 341.0992.

Methyl 1-(4-methoxyphenyl)-2-naphthoate (23)

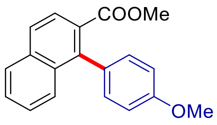

Compound **23** was prepared following the procedure **A**, starting from 2-naphthoic acid (34.8 mg, 0.20 mmol) and 5-(4-methoxyphenyl)-5*H*-thianthren-5-ium trifluoromethanesulfonate (113.4 mg, 0.24 mmol). After column chromatography on silica (Petroleum Ether: EtOAc = 10:1), **23** was afforded as white oil liquid (51.4 mg, 88%).

^{1}H NMR (400 MHz, $CDCl_3$): δ 8.38 (s, 1H), 7.95 – 7.91 (m, 1H), 7.87 – 7.83 (m, 1H), 7.81 (s, 1H), 7.60 – 7.51 (m, 2H), 7.38 – 7.33 (m, 2H), 7.01 – 6.96 (m, 2H), 3.87 (s, 3H), 3.75 (s, 3H) ppm.

^{13}C NMR (101 MHz, $CDCl_3$): δ 169.1, 158.8, 138.3, 134.4, 133.7, 131.3, 130.8, 129.6, 129.5, 129.2, 128.5, 128.1, 127.7, 126.5, 113.5, 55.2, 52.1 ppm.

Spectra data are consistent with the reported literature.[4]

Methyl 2-(4-methoxyphenyl)-1-naphthoate (24)

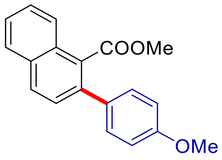

Compound **24** was prepared following the procedure **A**, starting from 1-naphthoic acid (35.0 mg, 0.20 mmol) and 5-(4-methoxyphenyl)-5*H*-thianthren-5-ium trifluoromethanesulfonate (113.4 mg, 0.24 mmol). After column chromatography on silica (Petroleum Ether: EtOAc = 15:1), **24** was afforded as white solid (34.4 mg, 59%).

M.P.: 117 – 118 °C.

^{1}H NMR (400 MHz, $CDCl_3$): δ 7.97 – 7.92 (m, 2H), 7.91 – 7.86 (m, 1H), 7.60 – 7.55 (m, 1H), 7.55 – 7.50 (m, 2H), 7.46 – 7.40 (m, 2H), 7.02 – 6.97 (m, 2H), 3.87 (s, 3H), 3.76 (s, 3H) ppm.

^{13}C NMR (101 MHz, $CDCl_3$): δ 170.2, 159.2, 137.5, 133.2, 132.0, 130.0, 129.8, 129.6, 128.1, 127.5, 127.4, 126.1, 124.9, 113.9, 55.2, 52.2 ppm.

Spectra data are consistent with the reported literature.[3]

Methyl 3-(4-methoxyphenyl)-1-methyl-1H-indole-2-carboxylate (25)

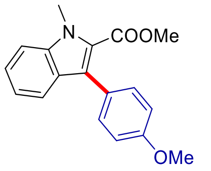

Compound **25** was prepared following the procedure **A**, starting from 1-methyl-1*H*-indole-2-carboxylic acid (35.8 mg, 0.20 mmol) and 5-(4-methoxyphenyl)-5*H*-thianthren-5-ium trifluoromethanesulfonate (113.4 mg, 0.24 mmol). After column chromatography on silica (Petroleum Ether: EtOAc = 10:1), **25** was afforded as yellow solid (20.6 mg, 34%).

M.P.: 106 – 109 °C.

^{1}H NMR (400 MHz, $CDCl_3$): δ 7.62 – 7.57 (m, 1H), 7.44 – 7.37 (m, 4H), 7.18 – 7.13 (m, 1H), 7.03 – 6.98 (m, 2H), 4.07 (s, 3H), 3.89 (s, 3H), 3.74 (s, 3H) ppm.

^{13}C NMR (101 MHz, $CDCl_3$): δ 163.2, 158.6, 138.5, 131.4, 126.8, 126.7, 125.3, 124.5, 124.4, 121.6, 120.5, 113.3, 110.0, 55.2, 51.3, 32.0 ppm.

HRMS (ESI-TOF) m/z: $[M+H]^+$ Calcd for $C_{18}H_{18}NO_3^+$ 296.1281; Found 296.1273.

Methyl 3-(4-methoxyphenyl)thiophene-2-carboxylate (26)

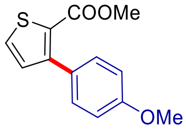

Compound **26** was prepared following the procedure **A**, starting from thiophene-2-carboxylic acid (25.6 mg, 0.20 mmol) and 5-(4-methoxyphenyl)-5*H*-thianthren-5-ium trifluoromethanesulfonate (113.4 mg, 0.24 mmol). After column chromatography on silica (Petroleum Ether: EtOAc = 10:1), **26** was afforded as colorless oil liquid (27.8 mg, 56%).

^{1}H NMR (400 MHz, $CDCl_3$): δ 7.50 – 7.47 (m, 1H), 7.45 – 7.40 (m, 2H), 7.10 – 7.05 (m, 1H), 6.97 – 6.92 (m, 2H), 3.85 (s, 3H), 3.79 (s, 3H) ppm.

^{13}C NMR (101 MHz, $CDCl_3$): δ 162.5, 159.4, 148.5, 131.5, 130.5, 130.1, 127.9, 126.0, 113.2, 55.2, 51.8 ppm.

Spectra data are consistent with the reported literature.[4]

Methyl 3-methyl-[1,1'-biphenyl]-2-carboxylate (27)

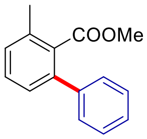

Compound **27** was prepared following the procedure **A**, starting from 2-methylbenzoic acid (28.2 mg, 0.20 mmol) and 5-phenyl-5*H*-thianthren-5-ium trifluoromethanesulfonate (106.2 mg, 0.24 mmol). After column chromatography on silica (Petroleum Ether: EtOAc = 40:1), **27** was afforded as colorless oil liquid (43.4 mg, 96%).

^{1}H NMR (400 MHz, $CDCl_3$): δ 7.43 – 7.40 (m, 1H), 7.40 – 7.38 (m, 3H), 7.37 – 7.32 (m, 2H), 7.25 – 7.21 (m, 2H), 3.59 (s, 3H), 2.42 (s, 3H) ppm.

^{13}C NMR (101 MHz, $CDCl_3$): δ 170.2, 140.9, 140.1, 135.4, 133.1, 129.4, 129.1, 128.2, 128.1, 127.3, 127.2, 51.8, 19.6 ppm.

Spectra data are consistent with the reported literature.[3]

Methyl 3,4'-dimethyl-[1,1'-biphenyl]-2-carboxylate (28)

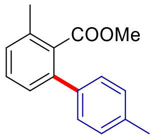

Compound **28** was prepared following the procedure **A**, starting from 2-methylbenzoic acid (28.2 mg, 0.20 mmol) and 5-(*p*-tolyl)-5*H*-thianthren-5-ium trifluoromethanesulfonate (109.6 mg, 0.24 mmol). After column chromatography on silica (Petroleum Ether: EtOAc = 40:1), **28** was afforded as colorless oil liquid (43.8 mg, 91%).

^{1}H NMR (400 MHz, $CDCl_3$): δ 7.37 – 7.33 (m, 1H), 7.29 – 7.27 (m, 2H), 7.23 – 7.20 (m, 4H), 3.63 (s, 3H), 2.41 (s, 3H), 2.40 (s, 3H) ppm.

^{13}C NMR (101 MHz, $CDCl_3$): δ 170.4, 140.0, 137.9, 137.0, 135.3, 133.1, 129.3, 129.0, 128.8, 128.0, 127.2, 51.8, 21.1, 19.6 ppm.

Spectra data are consistent with the reported literature.[4]

Methyl 4'-cyclohexyl-3-methyl-[1,1'-biphenyl]-2-carboxylate (29)

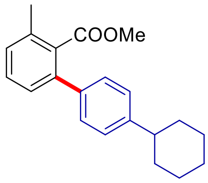

Compound **29** was prepared following the procedure **A**, starting from 2-methylbenzoic acid (28.2 mg, 0.20 mmol) and 5-(4-cyclohexylphenyl)-5*H*-thianthren-5-ium trifluoromethanesulfonate (120.6 mg, 0.24 mmol). After column chromatography on silica (Petroleum Ether: EtOAc = 40:1), **29** was afforded as white solid (59.8 mg, 97%).

M.P.: 54 – 56 °C.

^{1}H NMR (400 MHz, $CDCl_3$): δ 7.37 – 7.32 (m, 1H), 7.32 – 7.29 (m, 2H), 7.26 – 7.24 (m, 2H), 7.24 – 7.22 (m, 1H), 7.22 – 7.19 (m, 1H), 3.60 (s, 3H), 2.59 – 2.51 (m, 1H), 2.41 (s, 3H), 1.96 – 1.85 (m, 4H), 1.81 – 1.76 (m, 1H), 1.49 – 1.39 (m, 4H), 1.32 – 1.26 (m, 1H) ppm.

^{13}C NMR (101 MHz, $CDCl_3$): δ 170.4, 147.1, 140.1, 138.2, 135.2, 133.1, 129.3, 128.8, 128.0, 127.2, 126.7, 51.7, 44.1, 34.4, 26.8, 26.1, 19.6 ppm.

HRMS (ESI-TOF) m/z: $[M+H]^+$ Calcd for $C_{21}H_{25}O_2^+$ 309.1849; Found 309.1841.

Methyl 4'-(*tert*-butyl)-3-methyl-[1,1'-biphenyl]-2-carboxylate (30)

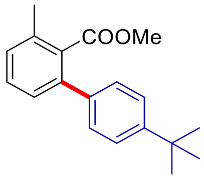

Compound **30** was prepared following the procedure **A**, starting from 2-methylbenzoic acid (28.2 mg, 0.20 mmol) and 5-(4-(*tert*-butyl)phenyl)-5*H*-thianthren-5-ium trifluoromethanesulfonate (119.6 mg, 0.24 mmol). After column chromatography on silica (Petroleum Ether: EtOAc = 40:1), **30** was afforded as light yellow oil liquid (54.2 mg, 96%).

^{1}H NMR (400 MHz, $CDCl_3$): δ 7.45 – 7.40 (m, 2H), 7.38 – 7.30 (m, 3H), 7.26 – 7.19 (m, 2H), 3.61 (s, 3H), 2.41 (s, 3H), 1.37 (s, 9H) ppm.

^{13}C NMR (101 MHz, $CDCl_3$): δ 170.4, 150.2, 140.0, 137.8, 135.3, 133.1, 129.3, 128.8, 127.8, 127.2, 125.2, 51.7, 34.5, 31.3, 19.6 ppm.

Spectra data are consistent with the reported literature.[3]

Methyl 3-methyl-[1,1':4',1''-terphenyl]-2-carboxylate (31)

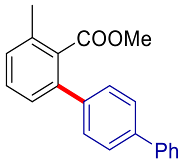

Compound **31** was prepared following the procedure **A**, starting from 2-methylbenzoic acid (28.2 mg, 0.20 mmol) and 5-([1,1'-biphenyl]-4-yl)-5*H*-thianthren-5-ium trifluoromethanesulfonate (124.4 mg, 0.24 mmol). After column chromatography on silica (Petroleum Ether: EtOAc = 10:1), **31** was afforded as white solid (58.0 mg, 96%).

M.P.: 79 – 80 °C.

^{1}H NMR (400 MHz, $CDCl_3$): δ 7.69 – 7.60 (m, 4H), 7.51 – 7.42 (m, 4H), 7.41 – 7.33 (m, 2H), 7.29 – 7.22 (m, 2H), 3.64 (s, 3H), 2.43 (s, 3H) ppm.

^{13}C NMR (101 MHz, $CDCl_3$): δ 170.3, 140.6, 140.1, 139.8, 139.6, 135.5, 133.1, 129.5, 129.2, 128.8, 128.6, 127.4, 127.2, 127.01, 126.97, 51.9, 19.7 ppm.

Spectra data are consistent with the reported literature.[3]

Methyl 3-methyl-4'-phenoxy-[1,1'-biphenyl]-2-carboxylate (32)

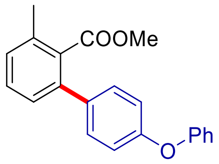

Compound **32** was prepared following the procedure **A**, starting from 2-methylbenzoic acid (28.2 mg, 0.20 mmol) and 5-(4-phenoxyphenyl)-5*H*-thianthren-5-ium trifluoromethanesulfonate (128.2 mg, 0.24 mmol). After column chromatography on silica (Petroleum Ether: EtOAc = 40:1), **32** was afforded as yellow oil liquid (58.6 mg, 92%).

^{1}H NMR (400 MHz, $CDCl_3$): δ 7.41 – 7.31 (m, 5H), 7.26 – 7.20 (m, 2H), 7.17 – 7.11 (m, 1H), 7.11 – 7.01 (m, 4H), 3.66 (s, 3H), 2.42 (s, 3H) ppm.

^{13}C NMR (101 MHz, $CDCl_3$): δ 170.3, 156.9, 156.8, 139.3, 135.8, 135.4, 133.1, 129.7, 129.5, 129.4, 128.9, 127.1, 123.4, 119.0, 118.4, 51.8, 19.6 ppm.

Spectra data are consistent with the reported literature.[3]

Methyl 4'-(benzoyloxy)-3-methyl-[1,1'-biphenyl]-2-carboxylate (33)

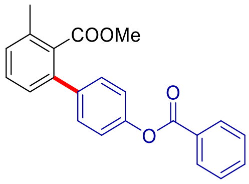

Compound **33** was prepared following the procedure **A**, starting from 2-methylbenzoic acid (28.2 mg, 0.20 mmol) and 5-(4-(benzoyloxy)phenyl)-5*H*-thianthren-5-ium trifluoromethanesulfonate (134.9 mg, 0.24 mmol). After column chromatography on silica (Petroleum Ether: EtOAc = 10:1), **33** was afforded as white solid (54.7 mg, 79%).

M.P.: 93 – 96 °C.

^{1}H NMR (400 MHz, $CDCl_3$): δ 8.16 – 8.11 (m, 2H), 7.59 – 7.53 (m, 1H), 7.46 – 7.41 (m, 2H), 7.36 – 7.32 (m, 2H), 7.30 – 7.26 (m, 1H), 7.18 – 7.13 (m, 4H), 3.54 (s, 3H), 2.33 (s, 3H) ppm.

^{13}C NMR (101 MHz, $CDCl_3$): δ 170.2, 165.1, 150.3, 139.2, 138.6, 135.5, 133.6, 133.1, 130.1, 129.5, 129.4, 129.30, 129.26, 128.6, 127.2, 121.5, 51.9, 19.7 ppm.

HRMS (ESI-TOF) m/z: $[M+H]^+$ Calcd for $C_{22}H_{19}O_4^+$ 347.1278; Found 347.1270.

Methyl 4'-fluoro-3-methyl-[1,1'-biphenyl]-2-carboxylate (34)

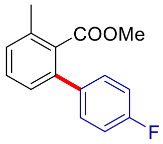

Compound **34** was prepared following the procedure **A**, starting from 2-methylbenzoic acid (28.2 mg, 0.20 mmol) and 5-(4-fluorophenyl)-5*H*-thianthren-5-ium trifluoromethanesulfonate (110.6 mg, 0.24 mmol). After column chromatography on silica (Petroleum Ether: EtOAc = 20:1), **34** was afforded as white solid (39.0 mg, 80%).

M.P.: 62 - 63 °C.

^{1}H NMR (400 MHz, $CDCl_3$): δ 7.37 – 7.30 (m, 3H), 7.24 – 7.20 (m, 1H), 7.20 – 7.16 (m, 1H), 7.11 – 7.04 (m, 2H), 3.61 (s, 3H), 2.40 (s, 3H) ppm.

^{13}C NMR (101 MHz, $CDCl_3$): δ 170.2, 162.3 (d, $J_{C\text{-}F}$ = 247.5 Hz), 139.0, 136.9 (d, $J_{C\text{-}F}$ = 4.0 Hz), 135.5, 133.2, 129.8 (d, $J_{C\text{-}F}$ = 8.1 Hz), 129.4, 129.2, 127.1, 115.2 (d, $J_{C\text{-}F}$ = 22.2 Hz), 51.9, 19.7 ppm.

Spectra data are consistent with the reported literature.[3]

Methyl 2-methyl-6-(5,6,7,8-tetrahydronaphthalen-2-yl)benzoate (35)

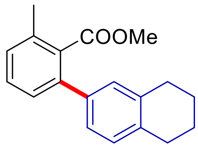

Compound **35** was prepared following the procedure **A**, starting from 2-methylbenzoic acid (28.2 mg, 0.20 mmol) and 5-(5,6,7,8-tetrahydronaphthalen-2-yl)-5*H*-thianthren-5-ium trifluoromethanesulfonate (119.2 mg, 0.24 mmol). After column chromatography on silica (Petroleum Ether: EtOAc = 40:1), **35** was afforded as colorless oil liquid (50.4 mg, 90%).

^{1}H NMR (400 MHz, $CDCl_3$): δ 7.37 – 7.32 (m, 1H), 7.26 – 7.18 (m, 2H), 7.14 – 7.07 (m, 3H), 3.68 (s, 3H), 2.84 – 2.79 (m, 4H), 2.41 (s, 3H), 1.84 (p, *J* = 3.3 Hz, 4H) ppm.

^{13}C NMR (101 MHz, $CDCl_3$): δ 170.5, 140.1, 137.9, 137.0, 136.3, 135.2, 133.0, 129.3, 129.0, 128.8, 128.7, 127.2, 125.2, 51.7, 29.4, 29.1, 23.1, 19.6 ppm.

HRMS (ESI-TOF) m/z: $[M+H]^+$ Calcd for $C_{19}H_{21}O_2^+$ 281.1536; Found 281.1528.

Methyl 3,3',4'-trimethyl-[1,1'-biphenyl]-2-carboxylate (36)

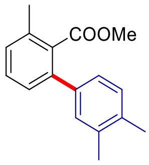

Compound **36** was prepared following the procedure **A**, starting from 2-methylbenzoic acid (28.2 mg, 0.20 mmol) and 5-(3,4-dimethylphenyl)-5*H*-thianthren-5-ium trifluoromethanesulfonate (113.0 mg, 0.24 mmol). After column chromatography on silica (Petroleum Ether: EtOAc = 40:1), **36** was afforded as colorless oil liquid (49.8 mg, 98%).

^{1}H NMR (400 MHz, $CDCl_3$): δ 7.37 – 7.33 (m, 1H), 7.25 – 7.19 (m, 2H), 7.19 – 7.16 (m, 2H), 7.15 – 7.11 (m, 1H), 3.66 (s, 3H), 2.42 (s, 3H), 2.31 (s, 6H) ppm.

^{13}C NMR (101 MHz, $CDCl_3$): δ 170.5, 140.0, 138.3, 136.4, 135.6, 135.2, 133.1, 129.5, 129.4, 129.3, 128.7, 127.2, 125.5, 51.7, 19.7, 19.6, 19.4 ppm.

HRMS (ESI-TOF) m/z: $[M+H]^+$ Calcd for $C_{17}H_{19}O_2^+$ 255.1380; Found 255.1371.

Methyl 3'-fluoro-3,4'-dimethyl-[1,1'-biphenyl]-2-carboxylate (37)

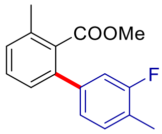

Compound **37** was prepared following the procedure **A**, starting from 2-methylbenzoic acid (28.2 mg, 0.20 mmol) and 5-(3-fluoro-4-methylphenyl)-5*H*-thianthren-5-ium trifluoromethanesulfonate (113.8 mg, 0.24 mmol). After column chromatography on silica (Petroleum Ether: EtOAc = 10:1), **40** was afforded as colorless oil liquid (41.3 mg, 80%).

^{1}H NMR (400 MHz, $CDCl_3$): δ 7.37 – 7.31 (m, 1H), 7.23 – 7.16 (m, 3H), 7.16 – 7.11 (m, 1H), 7.04 – 6.96 (m, 1H), 3.63 (s, 3H), 2.39 (s, 3H), 2.31 (s, 3H) ppm.

^{13}C NMR (101 MHz, $CDCl_3$): δ 170.3, 160.9 (d, $J_{C\text{-}F}$ = 246.4 Hz), 139.2, 136.6 (d, $J_{C\text{-}F}$ = 4.0 Hz), 135.4, 133.2, 131.3 (d, $J_{C\text{-}F}$ = 5.1 Hz), 129.4, 129.1, 127.1, 127.0 (d, $J_{C\text{-}F}$ = 9.1 Hz), 124.7 (d, $J_{C\text{-}F}$ = 18.2 Hz), 114.8 (d, $J_{C\text{-}F}$ = 22.2 Hz), 51.8, 19.7, 14.5 (d, $J_{C\text{-}F}$ = 4.0 Hz) ppm.

HRMS (ESI-TOF) m/z: $[M+H]^+$ Calcd for $C_{16}H_{16}FO_2^+$ 259.1129; Found 259.1114.

Dimethyl 3,4'-dimethyl-[1,1'-biphenyl]-2,3'-dicarboxylate (38)

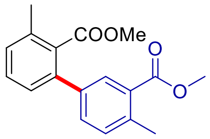

Compound **38** was prepared following the procedure **A**, starting from 2-methylbenzoic acid (28.2 mg, 0.20 mmol) and 5-(3-(methoxycarbonyl)-4-methylphenyl)-5*H*-thianthren-5-ium trifluoromethanesulfonate (123.4 mg, 0.24 mmol). After column chromatography on silica (Petroleum Ether: EtOAc = 10:1), **38** was afforded as colorless oil liquid (56.6 mg, 95%).

^{1}H NMR (400 MHz, $CDCl_3$): δ 7.89 – 7.84 (m, 1H), 7.33 – 7.29 (m, 1H), 7.28 – 7.23 (m, 1H), 7.19 – 7.15 (m, 1H), 7.14 – 7.09 (m, 2H), 3.79 (s, 3H), 3.55 (s, 3H), 2.53 (s, 3H), 2.30 (s, 3H) ppm.

^{13}C NMR (101 MHz, $CDCl_3$): δ 170.1, 167.7, 139.3, 138.8, 138.3, 135.4, 133.0, 131.7, 131.5, 130.3, 129.4, 129.4, 129.2, 127.1, 51.77, 51.75, 21.3, 19.6 ppm.

Spectra data are consistent with the reported literature.[4]

Methyl 3'-fluoro-4'-methoxy-3-methyl-[1,1'-biphenyl]-2-carboxylate (39)

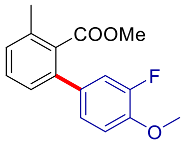

Compound **39**was prepared following the procedure **A**, starting from 2-methylbenzoic acid (28.2 mg, 0.20 mmol) and 5-(3-fluoro-4-methoxyphenyl)-5*H*-thianthren-5-ium trifluoromethanesulfonate (117.8 mg, 0.24 mmol). After column chromatography on silica (Petroleum Ether: EtOAc = 20:1), **39** was afforded as white solid (53.8 mg, 98%).

M.P.: 109 – 110 °C.

^{1}H NMR (400 MHz, $CDCl_3$): δ 7.36 – 7.30 (m, 1H), 7.23 – 7.18 (m, 1H), 7.18 – 7.15 (m, 1H), 7.15 – 7.09 (m, 1H), 7.09 – 7.04 (m, 1H), 7.01 – 6.94 (m, 1H), 3.91 (s, 3H), 3.66 (s, 3H), 2.38 (s, 3H) ppm.

^{13}C NMR (101 MHz, $CDCl_3$): δ 170.1, 151.9 (d, $J_{C\text{-}F}$ = 246.4 Hz), 147.0 (d, $J_{C\text{-}F}$ = 11.1 Hz), 138.4 (d, $J_{C\text{-}F}$ = 2.0 Hz), 135.4, 133.8 (d, $J_{C\text{-}F}$ = 7.1 Hz), 133.1, 129.4, 129.1, 127.0, 124.0 (d, $J_{C\text{-}F}$ = 4.0 Hz), 116.0 (d, $J_{C\text{-}F}$ = 19.2 Hz), 113.0 (d, $J_{C\text{-}F}$ = 2.0 Hz), 56.1, 51.8, 19.6 ppm.

Spectra data are consistent with the reported literature.[3]

Methyl 3'-cyano-4'-methoxy-3-methyl-[1,1'-biphenyl]-2-carboxylate (40)

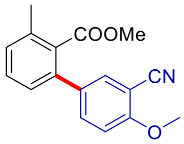

Compound **40** was prepared following the procedure **A**, starting from 2-methylbenzoic acid (28.2 mg, 0.20 mmol) and 5-(3-cyano-4-methoxyphenyl)-5*H*-thianthren-5-ium trifluoromethanesulfonate (119.4 mg, 0.24 mmol). After column chromatography on silica (Petroleum Ether: EtOAc = 3:1), **40** was afforded as yellow solid (37.6 mg, 67%).

M.P.: 130 – 133 °C.

^{1}H NMR (400 MHz, $CDCl_3$): δ 7.57 – 7.48 (m, 2H), 7.37 – 7.31 (m, 1H), 7.25 – 7.19 (m, 1H), 7.14 – 7.09 (m, 1H), 7.01 – 6.97 (m, 1H), 3.95 (s, 3H), 3.65 (s, 3H), 2.37 (s, 3H) ppm.

^{13}C NMR (101 MHz, $CDCl_3$): δ 169.8, 160.5, 137.3, 135.7, 134.2, 133.6, 133.2, 133.0, 129.6, 129.5, 126.9, 116.1, 111.2, 101.7, 56.1, 51.9, 19.6 ppm.

HRMS (ESI-TOF) m/z: $[M+H]^+$ Calcd for $C_{17}H_{16}NO_3^+$ 282.1125; Found 282.1119.

Methyl 4'-methoxy-3,3',5'-trimethyl-[1,1'-biphenyl]-2-carboxylate (41)

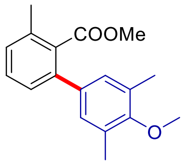

Compound **41** was prepared following the procedure **A**, starting from 2-methylbenzoic acid (28.2 mg, 0.20 mmol) and 5-(4-methoxy-3,5-dimethylphenyl)-5*H*-thianthren-5-ium trifluoromethanesulfonate (120.01 mg, 0.24 mmol). After column chromatography on silica (Petroleum Ether: EtOAc = 20:1), **41** was afforded as yellow oil liquid (50.6 mg, 89%).

^{1}H NMR (400 MHz, $CDCl_3$): δ 7.35 – 7.29 (m, 1H), 7.21 – 7.16 (m, 2H), 7.02 (s, 2H), 3.75 (s, 3H), 3.63 (s, 3H), 2.38 (s, 3H), 2.30 (s, 6H) ppm.

^{13}C NMR (101 MHz, $CDCl_3$): δ 170.5, 156.4, 139.7, 136.3, 135.2, 133.0, 130.7, 129.3, 128.8, 128.6, 127.1, 59.7, 51.7, 19.7, 16.1 ppm.

HRMS (ESI-TOF) m/z: $[M+H]^+$ Calcd for $C_{18}H_{21}O_3^+$ 285.1485; Found 285.1476.

Methyl 2-methyl-6-(9-oxo-9*H*-xanthen-2-yl)benzoate (42)

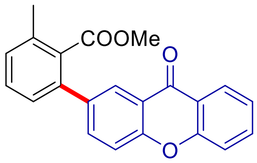

Compound **42** was prepared following the procedure **A**, starting from 2-methylbenzoic acid (28.2 mg, 0.20 mmol) and 5-(9-oxo-9*H*-xanthen-3-yl)-5*H*-thianthren-5-ium trifluoromethanesulfonate (134.4 mg, 0.24 mmol). After column chromatography on silica (Petroleum Ether: EtOAc = 5:1), **42** was afforded as white solid (26.2 mg, 38%).

M.P.: 150 – 153 °C.

^{1}H NMR (400 MHz, $CDCl_3$): δ 8.37 – 8.31 (m, 2H), 7.74 – 7.70 (m, 2H), 7.53 – 7.48 (m, 2H), 7.41 – 7.35 (m, 2H), 7.30 – 7.23 (m, 2H), 3.67 – 3.64 (m, 3H), 2.42 – 2.39 (m, 3H) ppm.

^{13}C NMR (101 MHz, $CDCl_3$): δ 177.0, 170.1, 156.1, 155.5, 138.3, 136.7, 135.7, 134.9, 134.8, 133.2, 129.6, 127.3, 126.7, 126.0, 124.0, 121.8, 121.6, 118.0, 51.9, 19.7 ppm.

HRMS (ESI-TOF) m/z: $[M+H]^+$ Calcd for $C_{22}H_{17}O_4^+$ 345.1121; Found 345.1121.

Methyl 2-methyl-6-(naphthalen-2-yl)benzoate (43)

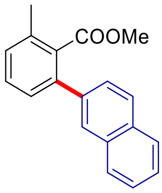

Compound **43** was prepared following the procedure **A**, starting from 2-methylbenzoic acid (28.2 mg, 0.20 mmol) and 5-(naphthalen-2-yl)-5*H*-thianthren-5-ium trifluoromethanesulfonate (118.2 mg, 0.24 mmol). After column chromatography on silica (Petroleum Ether: EtOAc = 40:1), **43** was afforded as yellow oil liquid (23.8 mg, 43%).

^{1}H NMR (400 MHz, $CDCl_3$): δ 7.91 – 7.84 (m, 2H), 7.72 – 7.65 (m, 1H), 7.52 – 7.47 (m, 2H), 7.45 – 7.40 (m, 2H), 7.39 – 7.36 (m, 1H), 7.35 – 7.30 (m, 1H), 7.28 – 7.24 (m, 1H), 3.28 (s, 3H), 2.49 (s, 3H) ppm.

^{13}C NMR (101 MHz, $CDCl_3$): δ 169.6, 138.7, 138.4, 135.6, 134.4, 133.4, 131.9, 129.4, 129.0, 128.4, 128.0, 127.8, 126.6, 126.2, 125.9, 125.7, 124.9, 51.4, 19.9 ppm.

Spectra data are consistent with the reported literature.[6]

Methyl 2'-methoxy-3-methyl-5'-(trifluoromethyl)-[1,1'-biphenyl]-2-carboxylate (44)

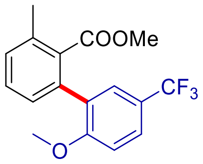

Compound **44** was prepared following the procedure **A**, starting from 2-methylbenzoic acid (28.2 mg, 0.20 mmol) and 5-(2-methoxy-5-(trifluoromethyl)phenyl)-5*H*-thianthren-5-ium trifluoromethanesulfonate (129.8 mg, 0.24 mmol). After column chromatography on silica (Petroleum Ether: EtOAc = 10:1), **44** was afforded as colorless oil liquid (42.2 mg, 65%).

^{1}H NMR (400 MHz, $CDCl_3$): δ 7.61 – 7.55 (m, 1H), 7.47 (s, 1H), 7.40 – 7.34 (m, 1H), 7.27 – 7.23 (m, 1H), 7.20 – 7.14 (m, 1H), 7.00 – 6.94 (m, 1H), 3.79 (s, 3H), 3.57 (s, 3H), 2.44 (s, 3H) ppm.

^{13}C NMR (101 MHz, $CDCl_3$): δ 169.3, 158.7, 136.1, 135.9, 133.3, 130.5, 130.0, 129.6, 128.3, 127.6 (q, $J_{C\text{-}F}$ = 3.0 Hz), 126.2 (q, $J_{C\text{-}F}$ = 4.0 Hz), 124.4 (q, $J_{C\text{-}F}$ = 272.7 Hz), 122.7 (q, $J_{C\text{-}F}$ = 32.3 Hz), 110.2, 55.7, 51.4, 20.3 ppm.

HRMS (ESI-TOF) m/z: $[M+H]^+$ Calcd for $C_{17}H_{16}F_3O_3^+$ 325.1046; Found 325.1040.

Methyl 2'-fluoro-3-methyl-[1,1'-biphenyl]-2-carboxylate (45)

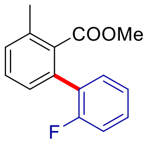

Compound **45** was prepared following the procedure **A**, starting from 2-methylbenzoic acid (28.2 mg, 0.20 mmol) and 5-(2-fluorophenyl)-5*H*-thianthren-5-ium trifluoromethanesulfonate (110.6 mg, 0.24 mmol). After column chromatography on silica (Petroleum Ether: EtOAc = 10:1), **45** was afforded as colorless oil liquid (36.2 mg, 74%).

^{1}H NMR (400 MHz, $CDCl_3$): δ 7.39 – 7.34 (m, 1H), 7.33 – 7.28 (m, 1H), 7.27 – 7.23 (m, 2H), 7.23 – 7.19 (m, 1H), 7.18 – 7.08 (m, 2H), 3.57 (s, 3H), 2.43 (s, 3H) ppm.

^{13}C NMR (101 MHz, $CDCl_3$): δ 169.5, 159.4 (d, J_{C-F} = 247.5 Hz), 136.1, 134.3, 133.4, 130.9 (d, J_{C-F} = 3.0 Hz), 130.0, 129.4, 129.3 (d, J_{C-F} = 8.1 Hz), 128.4 (d, J_{C-F} = 16.2 Hz), 128.1 (d, J_{C-F} = 1.0 Hz), 123.8 (d, J_{C-F} = 4.0 Hz), 115.4 (d, J_{C-F} = 22.2 Hz), 51.7, 20.1 ppm.

HRMS (ESI-TOF) m/z: $[M+H]^+$ Calcd for $C_{15}H_{14}FO_2^+$ 245.0972; Found 245.0972.

Methyl 2-methyl-6-(thiophen-2-yl)benzoate (46)

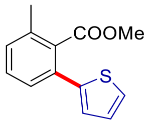

Compound **46** was prepared following the procedure **A**, starting from 2-methylbenzoic acid (28.2 mg, 0.20 mmol) and 5-(thiophen-2-yl)-5*H*-thianthren-5-ium trifluoromethanesulfonate (107.6 mg, 0.24 mmol). After column chromatography on silica (Petroleum Ether: EtOAc = 20:1), **46** was afforded as white oil liquid (24.6 mg, 53%).

^{1}H NMR (400 MHz, $CDCl_3$): δ 7.38 – 7.27 (m, 3H), 7.23 – 7.17 (m, 1H), 7.10 – 7.03 (m, 2H), 3.75 (s, 3H), 2.38 (s, 3H) ppm.

^{13}C NMR (101 MHz, $CDCl_3$): δ 170.2, 141.9, 135.3, 133.3, 132.0, 129.5, 129.3, 127.5, 127.4, 125.88, 125.87, 52.1, 19.5 ppm.

Spectra data are consistent with the reported literature.[2]

Methyl 5-chloro-4,4'-dimethoxy-[1,1'-biphenyl]-2-carboxylate (47)

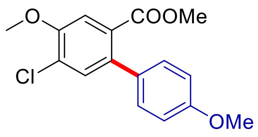

Compound **47** was prepared following the procedure **A**, starting from 4-chloro-3-methoxybenzoic acid (39.2 mg, 0.20 mmol) and 5-(4-methoxyphenyl)-5*H*-thianthren-5-ium trifluoromethanesulfonate (113.4 mg, 0.24 mmol) with $[Ru(p\text{-cymene})Cl_2]_2$ (6.2 mg, 0.01 mmol) and $P(Cy)_3$ (11.2 mg, 0.04 mmol). After column chromatography on silica (Petroleum Ether: EtOAc = 10:1), **47** was afforded as green oil liquid (46.0 mg, 75%).

^{1}H NMR (400 MHz, $CDCl_3$): δ 7.40 – 7.34 (m, 2H), 7.22 – 7.16 (m, 2H), 6.94 – 6.89 (m, 2H), 3.97 (s, 3H), 3.84 (s, 3H), 3.66 (s, 3H) ppm.

^{13}C NMR (101 MHz, $CDCl_3$): δ 168.4, 159.0, 153.7, 135.7, 132.4, 132.2, 129.6, 129.4, 125.6, 113.5, 113.1, 56.4, 55.2, 52.1 ppm.

HRMS (ESI-TOF) m/z: $[M+H]^+$ Calcd for $C_{16}H_{16}ClO_4^+$ 307.0732; Found 307.0731.

Methyl 4-chloro-4'-methoxy-3-methyl-[1,1'-biphenyl]-2-carboxylate (48)

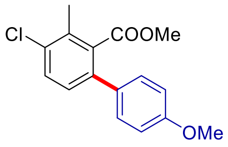

Compound **48** was prepared following the procedure **A**, starting from 3-chloro-2-methylbenzoic acid (34.2 mg, 0.20 mmol) and 5-(4-methoxyphenyl)-5*H*-thianthren-5-ium trifluoromethanesulfonate (113.4 mg, 0.24 mmol) with $[Ru(p\text{-cymene})Cl_2]_2$ (6.2 mg, 0.01 mmol) and $P(Cy)_3$ (11.2 mg, 0.04 mmol). After column chromatography on silica (Petroleum Ether: EtOAc = 10:1), **48** was afforded as yellow oil liquid (27.2 mg, 47%).

^{1}H NMR (400 MHz, $CDCl_3$): δ 7.37 – 7.32 (m, 1H), 7.21 – 7.16 (m, 2H), 7.07 – 7.04 (m, 1H), 6.86 – 6.82 (m, 2H), 3.76 (s, 3H), 3.56 (s, 3H), 2.31 (s, 3H) ppm.

^{13}C NMR (101 MHz, $CDCl_3$): δ 169.7, 159.2, 138.1, 135.0, 133.7, 133.0, 132.2, 130.0, 129.3, 128.3, 113.8, 55.2, 52.2, 17.3 ppm.

HRMS (ESI-TOF) m/z: $[M+H]^+$ Calcd for $C_{16}H_{16}ClO_3^+$ 291.0782; Found 291.0775.

Methyl 5-bromo-3,4'-dimethoxy-[1,1'-biphenyl]-2-carboxylate (49)

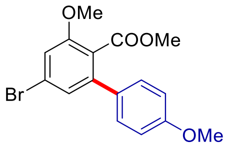

Compound **49** was prepared following the procedure **A**, starting from 4-bromo-2-methoxybenzoic acid (47.8 mg, 0.20 mmol) and 5-(4-methoxyphenyl)-5*H*-thianthren-5-ium trifluoromethanesulfonate (113.4 mg, 0.24 mmol). After column chromatography on silica (Petroleum Ether: EtOAc = 10:1), **49** was afforded as yellow oil liquid (30.2 mg, 43%).

^{1}H NMR (400 MHz, $CDCl_3$): δ 7.31 – 7.27 (m, 2H), 7.16 – 7.11 (m, 1H), 7.06 – 7.02 (m, 1H), 6.93 – 6.89 (m, 2H), 3.86 (s, 3H), 3.82 (s, 3H), 3.65 (s, 3H) ppm.

^{13}C NMR (101 MHz, $CDCl_3$): δ 167.9, 159.5, 157.0, 142.2, 131.0, 129.2, 124.8, 123.9, 122.0, 113.9, 112.9, 56.3, 55.2, 52.2 ppm.

HRMS (ESI-TOF) m/z: $[M+H]^+$ Calcd for $C_{16}H_{16}BrO_4^+$ 351.0226; Found 351.0222.

Methyl 4-bromo-4'-methoxy-[1,1'-biphenyl]-2-carboxylate (50)

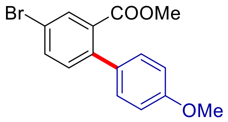

Compound **50** was prepared following the procedure **A**, starting from 3-bromobenzoic acid (40.2 mg, 0.20 mmol) and 5-(4-methoxyphenyl)-5*H*-thianthren-5-ium trifluoromethanesulfonate (113.4 mg, 0.24 mmol) with [Ru(p-cymene)Cl_2]$_2$ (6.2 mg, 0.01 mmol) and P(Cy)$_3$ (11.2 mg, 0.04 mmol). After column chromatography on silica (Petroleum Ether: EtOAc = 15:1), **50** was afforded as white solid (32.6 mg, 51%).

M.P.: 76 – 77 °C.

^{1}H NMR (400 MHz, $CDCl_3$): δ 7.94 – 7.91 (m, 1H), 7.65 – 7.59 (m, 1H), 7.25 – 7.19 (m, 3H), 6.96 – 6.91 (m, 2H), 3.84 (s, 3H), 3.68 (s, 3H) ppm.

^{13}C NMR (101 MHz, $CDCl_3$): δ 167.9, 159.2, 140.9, 134.1, 132.5, 132.34, 132.32, 132.27, 129.3, 120.6, 113.7, 55.3, 52.2 ppm.

Spectra data are consistent with the reported literature.[3]

Methyl 4-iodo-4'-methoxy-[1,1'-biphenyl]-2-carboxylate (51)

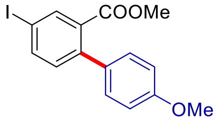

Compound **51** was prepared following the procedure **A**, starting from 3-iodobenzoic acid (49.8 mg, 0.20 mmol) and 5-(4-methoxyphenyl)-5*H*-thianthren-5-ium trifluoromethanesulfonate (113.4 mg, 0.24 mmol) with [Ru(p-cymene)Cl_2]$_2$ (6.2 mg, 0.01 mmol) and P(Cy)$_3$ (11.2 mg, 0.04 mmol). After column chromatography on silica (Petroleum Ether: EtOAc = 10:1), **51** was afforded as white solid (43.4 mg, 59%).

M.P.: 91 – 93 °C.

^{1}H NMR (400 MHz, $CDCl_3$): δ 8.14 – 8.07 (m, 1H), 7.86 – 7.77 (m, 1H), 7.24 – 7.18 (m, 2H), 7.12 – 7.07 (m, 1H), 6.96 – 6.91 (m, 2H), 3.84 (s, 3H), 3.67 (s, 3H) ppm.

^{13}C NMR (101 MHz, $CDCl_3$): δ 167.7, 159.2, 141.4, 140.0, 138.3, 132.5, 132.4, 129.3, 113.6, 91.6, 55.2, 52.2 ppm.

HRMS (ESI-TOF) m/z: $[M+H]^+$ Calcd for $C_{15}H_{14}IO_3^+$ 368.9982; Found 368.9981.

Methyl 4'-chloro-3-methyl-[1,1'-biphenyl]-2-carboxylate (52)

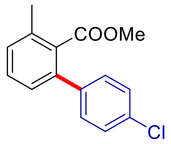

Compound **52** was prepared following the procedure **A**, starting from 2-methylbenzoic acid (28.2 mg, 0.20 mmol) and 5-(4-chlorophenyl)-5*H*-thianthren-5-ium trifluoromethanesulfonate (114.2 mg, 0.24 mmol). After column chromatography on silica (Petroleum Ether: EtOAc = 40:1), **52** was afforded as yellow oil liquid (46.3 mg, 89%).

^{1}H NMR (400 MHz, $CDCl_3$): δ 7.40 – 7.33 (m, 3H), 7.33 – 7.27 (m, 2H), 7.25 – 7.21 (m, 1H), 7.20 – 7.15 (m, 1H), 3.62 (s, 3H), 2.40 (s, 3H) ppm.

^{13}C NMR (101 MHz, $CDCl_3$): δ 170.0, 139.3, 138.8, 135.6, 133.5, 133.0, 129.51, 129.48, 129.4, 128.5, 127.1, 51.9, 19.7 ppm.

Spectra data are consistent with the reported literature.[3]

Methyl 4'-bromo-3-methyl-[1,1'-biphenyl]-2-carboxylate (53)

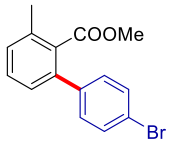

Compound **53** was prepared following the procedure **A**, starting from 2-methylbenzoic acid (28.2 mg, 0.20 mmol) and 5-(4-bromophenyl)-5*H*-thianthren-5-ium trifluoromethanesulfonate (124.8 mg, 0.24 mmol). After column chromatography on silica (Petroleum Ether: EtOAc = 40:1), **53** was afforded as yellow oil liquid (56.5 mg, 93%).

^{1}H NMR (400 MHz, $CDCl_3$): δ 7.53 – 7.48 (m, 2H), 7.37 – 7.32 (m, 1H), 7.25 – 7.20 (m, 3H), 7.18 – 7.14 (m, 1H), 3.61 (s, 3H), 2.39 (s, 3H) ppm.

^{13}C NMR (101 MHz, $CDCl_3$): δ 170.0, 139.8, 138.8, 135.6, 133.0, 131.4, 129.8, 129.5, 129.5, 127.0, 121.7, 51.9, 19.7 ppm.

Spectra data are consistent with the reported literature.[3]

Methyl 4'-iodo-3-methyl-[1,1'-biphenyl]-2-carboxylate (54)

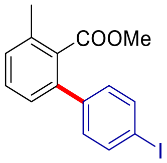

Compound **54** was prepared following the procedure **A**, starting from 2-methylbenzoic acid (28.2 mg, 0.20 mmol) and 5-(4-iodophenyl)-5*H*-thianthren-5-ium trifluoromethanesulfonate (136.3 mg, 0.24 mmol). After column chromatography on silica (Petroleum Ether: EtOAc = 40:1), **54** was afforded as white solid (66.9 mg, 95%).

M.P.: 73 – 74 °C.

^{1}H NMR (400 MHz, $CDCl_3$): δ 7.72 (d, *J* = 8.1 Hz, 2H), 7.38 – 7.33 (m, 1H), 7.25 – 7.21 (m, 1H), 7.19 – 7.15 (m, 1H), 7.14 – 7.08 (m, 2H), 3.63 (s, 3H), 2.40 (s, 3H) ppm.

^{13}C NMR (101 MHz, $CDCl_3$): δ 169.9, 140.3, 138.8, 137.3, 135.6, 132.9, 130.0, 129.5, 129.4, 126.9, 93.3, 51.9, 19.6 ppm.

Spectra data are consistent with the reported literature.[3]

Methyl 3'-chloro-4'-methoxy-3-methyl-[1,1'-biphenyl]-2-carboxylate (55)

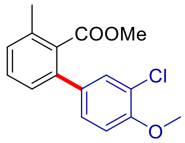

Compound **55** was prepared following the procedure **A**, starting from 2-methylbenzoic acid (28.2 mg, 0.20 mmol) and 5-(3-chloro-4-methoxyphenyl)-5*H*-thianthren-5-ium trifluoromethanesulfonate (121.6 mg, 0.24 mmol). After column chromatography on silica (Petroleum Ether: EtOAc = 20:1), **55** was afforded as white solid (56.8 mg, 98%).

M.P.: 109 – 110 °C.

^{1}H NMR (400 MHz, $CDCl_3$): δ 7.43 – 7.39 (m, 1H), 7.36 – 7.30 (m, 1H), 7.25 – 7.14 (m, 3H), 6.96 – 6.91 (m, 1H), 3.92 (s, 3H), 3.66 (s, 3H), 2.38 (s, 3H) ppm.

^{13}C NMR (101 MHz, $CDCl_3$): δ 170.2, 154.3, 138.2, 135.4, 134.1, 133.1, 130.0, 129.4, 129.2, 127.5, 127.0, 122.3, 111.7, 56.1, 51.9, 19.6 ppm.

Spectra data are consistent with the reported literature.[3]

Methyl 3'-bromo-4'-methoxy-3-methyl-[1,1'-biphenyl]-2-carboxylate (56)

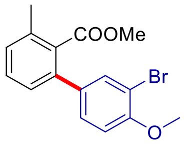

Compound **56** was prepared following the procedure **A**, starting from 2-methylbenzoic acid (28.2 mg, 0.20 mmol) and 5-(3-bromo-4-methoxyphenyl)-5*H*-thianthren-5-ium trifluoromethanesulfonate (129.8 mg, 0.24 mmol). After column chromatography on silica (Petroleum Ether: EtOAc = 10:1), **56** was afforded as yellow solid (58.8 mg, 88%).

M.P.: 84 – 87 °C.

^{1}H NMR (400 MHz, $CDCl_3$): δ 7.52 – 7.47 (m, 1H), 7.26 – 7.21 (m, 1H), 7.20 – 7.16 (m, 1H), 7.13 – 7.06 (m, 2H), 6.84 – 6.80 (m, 1H), 3.82 (s, 3H), 3.58 (s, 3H), 2.29 (s, 3H) ppm.

^{13}C NMR (101 MHz, $CDCl_3$): δ 170.1, 155.2, 138.1, 135.4, 134.5, 133.1, 133.0, 129.4, 129.1, 128.2, 127.0, 111.5, 111.4, 56.2, 51.9, 19.6 ppm.

Spectra data are consistent with the reported literature.[7]

Methyl 3'-iodo-4'-methoxy-3-methyl-[1,1'-biphenyl]-2-carboxylate (57)

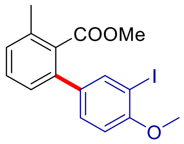

Compound **57** was prepared following the procedure **A**, starting from 2-methylbenzoic acid (28.2 mg, 0.20 mmol) and 5-(3-iodo-4-methoxyphenyl)-5*H*-thianthren-5-ium trifluoromethanesulfonate (143.5 mg, 0.24 mmol). After column chromatography on silica (Petroleum Ether: EtOAc = 10:1), **57** was afforded as yellow solid (73.3 mg, 96%).

M.P.: 86 – 88 °C.

^{1}H NMR (400 MHz, $CDCl_3$): δ 7.82 (s, 1H), 7.32 (d, *J* = 7.8 Hz, 2H), 7.22 – 7.15 (m, 2H), 6.83 (d, *J* = 8.4 Hz, 1H), 3.90 (s, 3H), 3.68 (s, 3H), 2.39 (s, 3H) ppm.

^{13}C NMR (101 MHz, $CDCl_3$): δ 170.1, 157.5, 139.1, 138.0, 135.4, 135.1, 133.1, 129.4, 129.3, 129.1, 127.0, 110.4, 85.7, 56.3, 51.9, 19.6 ppm.

HRMS (ESI-TOF) m/z: $[M+H]^+$ Calcd for $C_{16}H_{16}IO_3^+$ 383.0139; Found 383.0136.

Methyl 3'-bromo-5'-fluoro-4'-methoxy-3-methyl-[1,1'-biphenyl]-2-carboxylate (58)

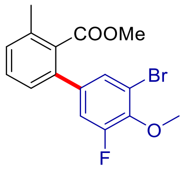

Compound **58** was prepared following the procedure **A**, starting from 2-methylbenzoic acid (28.2 mg, 0.20 mmol) and 5-(3-bromo-5-fluoro-4-methoxyphenyl)-5*H*-thianthren-5-ium trifluoromethanesulfonate (136.5 mg, 0.24 mmol). After column chromatography on silica (Petroleum Ether: EtOAc = 10:1), **58** was afforded as yellow solid (56.3 mg, 80%).

M.P.: 78 – 80°C.

^{1}H NMR (400 MHz, $CDCl_3$): δ 7.38 – 7.31 (m, 2H), 7.24 – 7.22 (m, 1H), 7.15 (d, *J* = 7.7 Hz, 1H), 7.08 (dd, *J* = 11.5, 1.7 Hz, 1H), 3.99 (s, 3H), 3.70 (s, 3H), 2.39 (s, 3H) ppm.

^{13}C NMR (101 MHz, $CDCl_3$): δ 184.2, 169.7, 155.2 (d, *J* = 252.0 Hz), 144.53 (d, *J* = 12.9 Hz), 137.6 (d, *J* = 8.0 Hz), 137.1 (d, *J* = 1.7 Hz), 135.7, 133.0, 129.8, 129.5, 128.1 (d, *J* = 3.1 Hz), 126.9, 117.2 (d, *J* = 3.7 Hz), 116.2 (d, *J* = 20.7 Hz), 61.4 (d, *J* = 5.2 Hz), 52.0, 19.6 ppm.

HRMS (ESI-TOF) m/z: $[M+H]^+$ Calcd for $C_{16}H_{15}BrFO_3^+$ 353.0183; Found 353.0188.

methyl 4'-bromo-5-chloro-4-methoxy-[1,1'-biphenyl]-2-carboxylate (59)

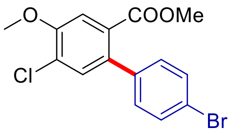

Compound **47** was prepared following the procedure **A**, starting from 4-chloro-3-methoxybenzoic acid (39.2 mg, 0.20 mmol) and 5-(4-bromophenyl)-5*H*-thianthren-5-ium trifluoromethanesulfonate (124.8 mg, 0.24 mmol) with $[Ru(p\text{-cymene})Cl_2]_2$ (6.2 mg, 0.01 mmol) and $P(Cy)_3$ (11.2 mg, 0.04 mmol). After column chromatography on silica (Petroleum Ether: EtOAc = 10:1), **59** was afforded as yellow oil liquid (53.9 mg, 76%).

^{1}H NMR (400 MHz, $CDCl_3$): δ 7.51 (d, *J* = 8.3 Hz, 2H), 7.42 (s, 1H), 7.34 (s, 1H), 7.13 (d, *J* = 8.2 Hz, 2H), 3.98 (s, 3H), 3.66 (s, 3H) ppm.

^{13}C NMR (101 MHz, $CDCl_3$): δ 167.7, 154.2, 138.9, 134.9, 132.3, 131.2, 130.0, 129.4, 125.9, 121.6, 113.3, 56.4, 52.2 ppm.

HRMS (ESI-TOF) m/z: $[M+H]^+$ Calcd for $C_{15}H_{13}BrClO_3^+$ 354.9731; Found 354.9718.

Methyl 4'-methoxy-4-(4,4,5,5-tetramethyl-1,3,2-dioxaborolan-2-yl)-[1,1'-biphenyl]-2-carboxylate (60)

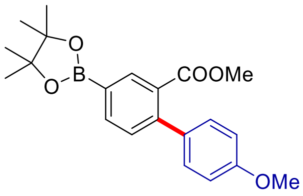

Compound **60** was prepared following the procedure **A**, starting from 3-(4,4,5,5-tetramethyl-1,3,2-dioxaborolan-2-yl)benzoic acid (50.6 mg, 0.20 mmol) and 5-(4-methoxyphenyl)-5*H*-thianthren-5-ium trifluoromethanesulfonate (113.4 mg, 0.24 mmol). After column chromatography on silica (Petroleum Ether: EtOAc = 10:1), **60** was afforded as yellow oil liquid (37.5 mg, 51%).

^{1}H NMR (400 MHz, $CDCl_3$): δ 8.20 (s, 1H), 7.95 – 7.87 (m, 1H), 7.39 – 7.34 (m, 1H), 7.27 – 7.23 (m, 2H), 6.95 – 6.90 (m, 2H), 3.83 (s, 3H), 3.67 (s, 3H), 1.35 (s, 12H) ppm.

^{13}C NMR (101 MHz, $CDCl_3$): δ 169.4, 159.1, 144.5, 137.3, 136.0, 133.4, 130.3, 130.0, 129.4, 113.6, 84.1, 55.2, 52.0, 24.8 ppm.

HRMS (ESI-TOF) m/z: $[M+H]^+$ Calcd for $C_{21}H_{26}BO_5^+$ 369.1868; Found 369.1863.

Methyl 4-acetamido-4'-methoxy-[1,1'-biphenyl]-2-carboxylate (61)

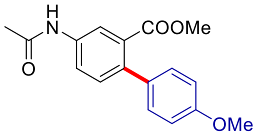

Compound **61** was prepared following the procedure **A**, starting from 3-acetamidobenzoic acid (36.2 mg, 0.20 mmol) and 5-(4-methoxyphenyl)-5*H*-thianthren-5-ium trifluoromethanesulfonate (113.4 mg, 0.24 mmol) with $[Ru(p\text{-}cymene)Cl_2]_2$ (6.2 mg, 0.01 mmol) and $P(Cy)_3$ (11.2 mg, 0.04 mmol). After column chromatography on silica (Petroleum Ether: EtOAc = 1:1), **61** was afforded as white solid (24.5 mg, 41%).

M.P.: 175 – 178 °C.

^{1}H NMR (400 MHz, $CDCl_3$): δ 7.84 – 7.79 (m, 1H), 7.78 – 7.74 (m, 1H), 7.62 (s, 1H), 7.31 – 7.27 (m, 1H), 7.21 – 7.17 (m, 2H), 6.92 – 6.88 (m, 2H), 3.84 – 3.80 (m, 3H), 3.67 – 3.63 (m, 3H), 2.17 (s, 3H) ppm.

^{13}C NMR (101 MHz, $CDCl_3$): δ 168.9, 168.6, 158.9, 137.8, 136.7, 133.0, 131.4, 131.0, 129.4, 122.6, 120.8, 113.5, 55.2, 52.1, 24.5 ppm.

HRMS (ESI-TOF) m/z: $[M+H]^+$ Calcd for $C_{17}H_{18}NO_4^+$ 300.1230; Found 300.1225.

Methyl 4'-methoxy-4-(pyridin-2-yl)-[1,1'-biphenyl]-2-carboxylate (62)

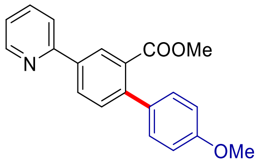

Compound **62** was prepared following the procedure **A**, starting from 3-(pyridin-2-yl)benzoic acid (40.6 mg, 0.20 mmol) and 5-(4-methoxyphenyl)-5*H*-thianthren-5-ium trifluoromethanesulfonate (113.4 mg, 0.24 mmol) with $[Ru(p\text{-cymene})Cl_2]_2$ (6.2 mg, 0.01 mmol) and $P(Cy)_3$ (11.2 mg, 0.04 mmol) at 60 °C. After column chromatography on silica (Petroleum Ether: EtOAc = 5:1), **62** was afforded as white solid (22.3 mg, 35%).

M.P.: 91 – 94 °C.

^{1}H NMR (400 MHz, $CDCl_3$): δ 8.72 – 8.71(m, 1H), 8.43 – 8.41 (m, 1H), 8.17 – 8.14 (m, 1H), 7.78 – 7.76 (m, 2H), 7.48 – 7.45 (m, 1H), 7.30 – 7.24 (m, 3H), 6.96 – 6.93 (m, 2H), 3.85 (s, 3H), 3.71 (s, 3H) ppm.

^{13}C NMR (101 MHz, $CDCl_3$): δ 169.2, 159.1, 156.0, 149.8, 142.4, 137.7, 136.9, 133.1, 131.23, 131.15, 129.5, 129.4, 128.2, 122.5, 120.4, 113.6, 55.2, 52.1 ppm.

HRMS (ESI-TOF) m/z: $[M+H]^+$ Calcd for $C_{20}H_{18}NO_3^+$ 320.1281; Found 320.1281.

3-carbamoyl-4'-methoxy-[1,1'-biphenyl]-2-carboxylic acid (63)

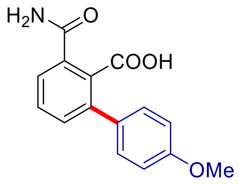

Compound **63** was prepared following the procedure **A**, starting from 2-((1-phenylethyl)carbamoyl)benzoic acid (55.6 mg, 0.20 mmol) and 5-(4-methoxyphenyl)-5H-thianthren-5-ium trifluoromethanesulfonate (113.4 mg, 0.24 mmol). After the reaction was comleted, 2.0 mL of Hydrochloric acid solution (1.0 mol/L) was added, and then the mixture was stirred at rt for 12 h. After column chromatography on silica (Petroleum Ether: EtOAc = 1:2, 2.5% formic acid and 2.5% methanol in EtOAc), **63** was afforded as yellow oil liquid containing 23% NMP (56.3 mg, 80%).

^{1}H NMR (400 MHz, DMSO-d_6): δ 7.86 (dd, J = 7.0, 2.0 Hz, 1H), 7.58 – 7.48 (m, 2H), 7.36 – 7.29 (m, 2H), 7.03 – 6.95 (m, 2H), 3.78 (s, 3H), 3.41 (br, 2H) ppm.

^{13}C NMR (101 MHz, DMSO-d_6): δ 169.6, 167.3, 158.9, 138.9, 135.4, 133.8, 131.7, 129.8, 129.1, 128.8, 128.2, 113.7, 55.2 ppm.

HRMS (ESI-TOF) m/z: $[M+H]^+$ Calcd for $C_{15}H_{14}NO_4^+$ 272.0917; Found 272.0927.

Methyl 3-methyl-4'-(2-oxopyrrolidin-1-yl)-[1,1'-biphenyl]-2-carboxylate (64)

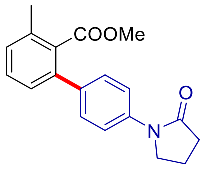

Compound **64** was prepared following the procedure **A**, starting from 2-methylbenzoic acid (28.2 mg, 0.20 mmol) and 5-(4-(2-oxopyrrolidin-1-yl)phenyl)-5*H*-thianthren-5-ium trifluoromethanesulfonate (126.0 mg, 0.24 mmol) with $[Ru(p\text{-cymene})Cl_2]_2$ (6.2 mg, 0.01 mmol) and $P(Cy)_3$ (11.2 mg, 0.04 mmol). After column chromatography on silica (Petroleum Ether: EtOAc = 3:1), **64** was afforded as white solid (55.6 mg, 90%).

M.P.: 136 – 139 °C.

^{1}H NMR (400 MHz, $CDCl_3$): δ 7.68 – 7.63 (m, 2H), 7.38 – 7.34 (m, 2H), 7.34 – 7.30 (m, 1H), 7.21 – 7.16 (m, 2H), 3.87 (t, *J* = 7.0 Hz, 2H), 3.62 (s, 3H), 2.64 – 2.58 (m, 2H), 2.38 (s, 3H), 2.19 – 2.11 (m, 2H) ppm.

^{13}C NMR (101 MHz, $CDCl_3$): δ 174.2, 170.2, 139.3, 138.7, 136.7, 135.3, 133.0, 129.3, 129.0, 128.5, 127.1, 119.3, 51.9, 48.6, 32.7, 19.6, 17.9 ppm.

HRMS (ESI-TOF) m/z: $[M+H]^+$ Calcd for $C_{19}H_{20}NO_3^+$ 310.1438; Found 310.1429.

Methyl 3-methyl-4'-(4-(2-(pyridin-2-yloxy)propoxy)phenoxy)-[1,1'-biphenyl]-2-carboxylate (65)

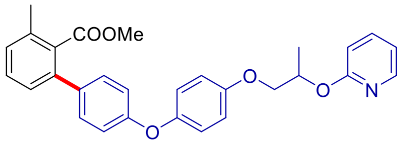

Compound **65** was prepared following the procedure **A**, starting from 2-methylbenzoic acid (28.2 mg, 0.20 mmol) and 5-(4-(4-(2-(pyridin-2-yloxy)propoxy)phenoxy)phenyl)-5*H*-thianthren-5-ium trifluoromethanesulfonate (164.6 mg, 0.24 mmol). After column chromatography on silica (Petroleum Ether: EtOAc = 5:1), **65** was afforded as colorless oil liquid (84.4 mg, 90%).

^{1}H NMR (400 MHz, $CDCl_3$): δ 8.20 – 8.12 (m, 1H), 7.64 – 7.50 (m, 1H), 7.36 – 7.27 (m, 3H), 7.23 – 7.17 (m, 2H), 7.03 – 6.93 (m, 6H), 6.90 – 6.82 (m, 1H), 6.81 – 6.69 (m, 1H), 5.65 – 5.56 (m, 1H), 4.24 – 4.18 (m, 1H), 4.12 – 4.07 (m, 1H), 3.64 (s, 3H), 2.39 (s, 3H), 1.50 (d, *J* = 6.4 Hz, 3H) ppm.

^{13}C NMR (101 MHz, $CDCl_3$): δ 170.3, 163.1, 158.0, 155.3, 149.9, 146.7, 139.4, 138.7, 135.3, 135.0, 133.1, 129.41, 129.35, 128.9, 127.1, 120.9, 117.2, 116.7, 115.8, 111.6, 71.0, 69.2, 51.8, 19.6, 16.9 ppm.

HRMS (ESI-TOF) m/z: $[M+H]^+$ Calcd for $C_{29}H_{28}NO_5^+$ 470.1962; Found 470.1967.

Methyl 2''-fluoro-4''-(1-methoxy-1-oxopropan-2-yl)-3-methyl-[1,1':4',1''-terphenyl]-2-carboxylate (66)

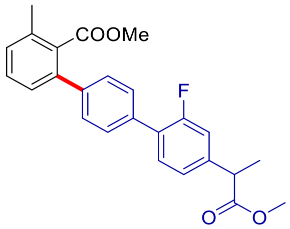

Compound **66** was prepared following the procedure **A**, starting from 2-methylbenzoic acid (28.2 mg, 0.20 mmol) and 5-(2-fluoro-4'-(1-methoxy-1-oxopropan-2-yl)-[1,1'-biphenyl]-4-yl)-5*H*-thianthren-5-ium trifluoromethanesulfonate (149.3 mg, 0.24 mmol). After column chromatography on silica (Petroleum Ether: EtOAc = 10:1), **66** was afforded as yellow oil liquid (74.7 mg, 92%).

^{1}H NMR (400 MHz, $CDCl_3$): δ 7.51 – 7.45 (m, 2H), 7.38 – 7.31 (m, 3H), 7.30 – 7.25 (m, 1H), 7.18 – 7.12 (m, 2H), 7.10 – 7.02 (m, 2H), 3.68 (q, *J* = 7.2 Hz, 1H), 3.61 (s, 3H), 3.53 (s, 3H), 2.32 (s, 3H), 1.45 (d, *J* = 7.1 Hz, 3H) ppm.

^{13}C NMR (101 MHz, $CDCl_3$): δ 174.3, 170.2, 159.7 (d, $J_{C\text{-}F}$ = 249.5 Hz), 141.9 (d, $J_{C\text{-}F}$ = 8.1 Hz), 140.2, 139.5, 135.5, 134.4 (d, $J_{C\text{-}F}$ = 1.0 Hz), 133.0, 130.6 (d, $J_{C\text{-}F}$ = 4.0 Hz), 129.4, 129.2, 128.8 (d, $J_{C\text{-}F}$ = 3.0 Hz), 128.2, 127.2 (d, $J_{C\text{-}F}$ = 15.2 Hz), 127.1, 123.5 (d, $J_{C\text{-}F}$ = 3.0 Hz), 115.2 (d, $J_{C\text{-}F}$ = 24.2 Hz), 52.11, 51.79, 44.8 (d, $J_{C\text{-}F}$ = 1.0 Hz), 19.62, 18.32 ppm.

HRMS (ESI-TOF) m/z: $[M+H]^+$ Calcd for $C_{25}H_{24}FO_4^+$ 407.1653; Found 407.1653.

4''-chloro-4'-(2-chloronicotinamido)-3-methyl-[1,1':3',1''-terphenyl]-2-carboxylic acid (67)

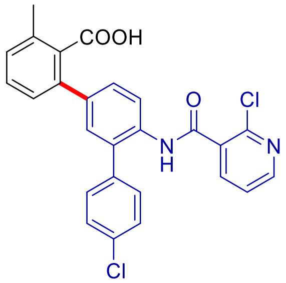

Compound **67** was prepared following the procedure **A**, starting from 2-methylbenzoic acid (28.2 mg, 0.20 mmol) and 5-(4'-chloro-6-(2-chloronicotinamido)-[1,1'-biphenyl]-3-yl)-5*H*-thianthren-5-ium trifluoromethanesulfonate (169.4 mg, 0.24 mmol). After the reaction completed, acidification was uesd with formic acid (2.0 mmol, 10 equiv.). After column chromatography on silica (Petroleum Ether: EtOAc = 1:1, 2.5% formic acid and 2.5% methanol in petroleum ether), **67** was afforded as brown solid (51.4 mg, 54%).

M.P.: 129 – 132 °C.

^{1}H NMR (400 MHz, DMSO-d_6): δ 13.14 (br, 1H), 10. 2 (s, 1H), 8.50 (s, 1H), 7.94 – 7.91 (m, 1H), 7.70 – 7.67 (m, 1H), 7.56 – 7.49 (m, 6H), 7.45 – 7.39 (m, 2H), 7.35 – 7.28 (m, 2H), 2.37 (s, 3H) ppm.

^{13}C NMR (101 MHz, DMSO): δ 170.7, 164.3, 150.4, 146.5, 138.87, 137.89, 137.5, 137.3, 135.9, 134.8, 134.1, 133.4, 133.0, 132.4, 130.8, 130.0, 129.3, 129.0, 128.5, 128.1, 127.4, 127.1, 123.1, 19.3 ppm.

HRMS (ESI-TOF) m/z: $[M+Na]^+$ Calcd for $C_{26}H_{18}Cl_2N_2O_3Na^+$ 499.0587; Found 499.0582.

Methyl 3-methyl-4'-(2-(*N*-methylmethylsulfonamido)-5-nitrophenoxy)-[1,1'-biphenyl]-2-carboxylate (68)

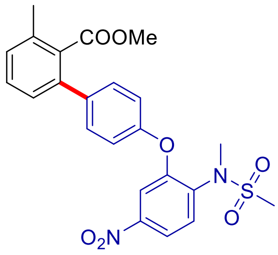

Compound **68** was prepared following the procedure **A**, starting from 2-methylbenzoic acid (28.2 mg, 0.20 mmol) and 5-(4-(2-(methylsulfonamido)-6-nitrophenoxy)phenyl)-5*H*-thianthren-5-ium trifluoromethanesulfonate (161.2 mg, 0.24 mmol). After column chromatography on silica (Petroleum Ether: EtOAc = 3:1), **68** was afforded as yellow oil liquid (48.0 mg, 51%).

^{1}H NMR (400 MHz, $CDCl_3$): δ 7.94 – 7.83 (m, 2H), 7.59 – 7.54 (m, 1H), 7.45 – 7.40 (m, 2H), 7.38 – 7.32 (m, 3H), 7.25 – 7.21 (m, 1H), 7.19 – 7.14 (m, 1H), 4.02 (s, 3H), 3.57 (s, 3H), 3.22 (s, 3H), 2.38 (s, 3H) ppm.

^{13}C NMR (101 MHz, $CDCl_3$): δ 170.0, 156.7, 148.3, 140.0, 139.5, 138.7, 135.7, 135.3, 133.0, 132.7, 129.5, 129.4, 127.0, 125.9, 116.7, 107.7, 56.4, 51.8, 40.3, 19.7 ppm.

HRMS (ESI-TOF) m/z: $[M+H]^+$ Calcd for $C_{23}H_{23}N_2O_7S^+$ 471.1220; Found 471.1219.

Methyl 2-(acetoxymethyl)-6-((3-(acetoxymethyl)-2'-(methoxycarbonyl)-3'-methyl-[1,1'-biphenyl]-4-yl)oxy)tetrahydro-2*H*-pyran-3,4,5-triyl triacetate (69)

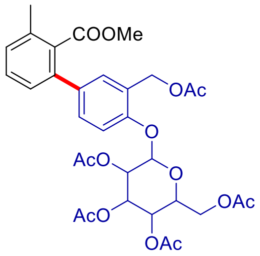

Compound **69** was prepared following the procedure **A**, starting from 2-methylbenzoic acid (28.2 mg, 0.20 mmol) and 5-(3-(acetoxymethyl)-4-(((2*S*,3*R*,4*S*,5*R*,6*R*)-3,4,5-triacetoxy-6-(acetoxymethyl)tetrahydro-2H-pyran-2-yl)oxy)phenyl)-5*H*-thianthren-5-ium trifluoromethanesulfonate (206.4 mg, 0.24 mmol). After column chromatography on silica (Petroleum Ether: EtOAc = 3:1), **69** was afforded as yellow oil liquid (95.3 mg, 74%).

^{1}H NMR (400 MHz, $CDCl_3$): δ 7.30 – 7.24 (m, 2H), 7.22 – 7.18 (m, 1H), 7.16 – 7.11 (m, 1H), 7.11 - 7.09 (m, 1H), 7.04 – 6.99 (m, 1H), 5.30 – 5.20 (m, 2H), 5.14 – 4.96 (m, 4H), 4.25 – 4.19 (m, 1H), 4.16 – 4.09 (m, 1H), 3.85 – 3.79 (m, 1H), 3.56 (s, 3H), 2.31 (s, 3H), 2.06 – 1.96 (m, 15H) ppm.

^{13}C NMR (101 MHz, $CDCl_3$): δ 170.6, 170.5, 170.20, 170.15, 169.3, 169.2, 153.7, 138.8, 136.1, 135.4, 133.1, 129.4, 129.2, 128.9, 128.7, 127.1, 126.2, 115.4, 99.2, 72.5, 72.0, 70.9, 68.2, 61.8, 60.7, 51.9, 20.9, 20.6, 20.5, 20.5, 19.6 ppm.

HRMS (ESI-TOF) m/z: $[M+Na]^+$ Calcd for $C_{32}H_{36}O_{14}Na^+$ 667.1997; Found 667.1991.

Methyl 6-(3-(adamantan-1-yl)-4-methoxyphenyl)-1-(4-methoxyphenyl)-2-naphthoate (70)

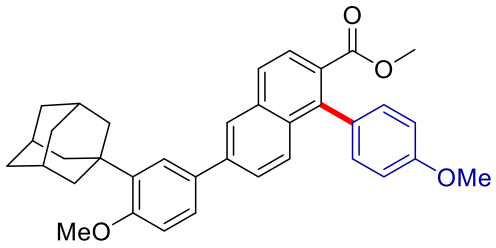

Compound **70** was prepared following the procedure **A**, starting from 6-(3-(adamantan-2-yl)-4-methoxyphenyl)-2-naphthoic acid (82.5 mg, 0.20 mmol) and 5-(4-methoxyphenyl)-5*H*-thianthren-5-ium trifluoromethanesulfonate (113.4 mg, 0.24 mmol). After column chromatography on silica (Petroleum Ether: EtOAc = 10:1), **70** was afforded as white solid (82.0 mg, 77%).

M.P.: 146 – 148 °C.

^{1}H NMR (400 MHz, $CDCl_3$): δ 8.40 (s, 1H), 8.01 (s, 1H), 7.99 – 7.95 (m, 1H), 7.86 (s, 1H), 7.82 – 7.78 (m, 1H), 7.65 – 7.62 (m, 1H), 7.57 – 7.53 (m, 1H), 7.41 – 7.35 (m, 2H), 7.03 – 6.97 (m, 3H), 3.92 – 3.87 (m, 6H), 3.77 (s, 3H), 2.22 (s, 6H), 2.13 (s, 3H), 1.86 – 1.80 (m, 6H) ppm.

^{13}C NMR (101 MHz, $CDCl_3$): δ 169.1, 158.8, 141.2, 138.9, 138.7, 134.8, 133.9, 132.5, 130.7, 130.1, 129.7, 129.6, 128.8, 128.5, 126.3, 125.9, 125.6, 124.6, 113.5, 112.0, 55.2, 55.1, 52.0, 40.5, 37.1, 37.1, 29.1 ppm.

HRMS (ESI-TOF) m/z: $[M+H]^+$ Calcd for $C_{36}H_{37}O_4^+$ 533.2686; Found 533.2686.

Methyl 3-ethoxy-4'-methoxy-5-(2-((3-methyl-1-(2-(piperidin-1-yl)phenyl)butyl)amino)-2-oxoethyl)-[1,1'-biphenyl]-2-carboxylate (71)

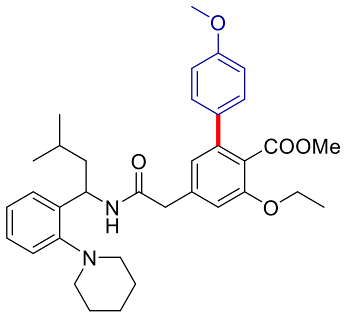

Compound **71** was prepared following the procedure **A**, starting from 2-ethoxy-4-(2-((3-methyl-1-(2-(piperidin-1-yl)phenyl)butyl)amino)-2-oxoethyl)benzoic acid (91.4 mg, 0.20 mmol) and 5-(4-methoxyphenyl)-5*H*-thianthren-5-ium trifluoromethanesulfonate (113.4 mg, 0.24 mmol). After column chromatography on silica (Petroleum Ether: EtOAc = 3:1), **71** was afforded as yellow oil liquid (107.6 mg, 94%).

^{1}H NMR (400 MHz, $CDCl_3$): δ 7.20 – 7.15 (m, 2H), 7.14 – 7.08 (m, 2H), 7.04 – 7.00 (m, 1H), 6.98 – 6.93 (m, 1H), 6.84 – 6.77 (m, 2H), 6.74 – 6.69 (m, 2H), 6.69 – 6.64 (m, 1H), 5.35 – 5.28 (m, 1H), 3.97 – 3.89 (m, 2H), 3.74 (s, 3H), 3.58 (s, 3H), 3.50 – 3.43 (m, 2H), 2.92 – 2.78 (m, 2H), 2.59 – 2.46 (m, 2H), 1.64 – 1.57 (m, 2H), 1.55 – 1.39 (m, 6H), 1.37 – 1.33 (m, 1H), 1.30 – 1.25 (m, 3H), 0.85 – 0.81 (m, 6H) ppm.

^{13}C NMR (101 MHz, $CDCl_3$): δ 169.0, 168.5, 159.2, 156.2, 152.5, 141.0, 138.7, 137.8, 132.1, 129.2, 127.8, 127.7, 125.0, 122.7, 122.6, 122.2, 113.7, 111.2, 64.5, 55.2, 52.0, 49.7, 46.6, 44.2, 26.6, 25.3, 24.1, 22.7, 22.4, 14.6 ppm.

HRMS (ESI-TOF) m/z: $[M+H]^+$ Calcd for $C_{35}H_{45}N_2O_5^+$ 573.3323; Found 573.3323.

Methyl 4-(cyclopropylmethoxy)-5-(difluoromethoxy)-4'-methoxy-[1,1'-biphenyl]-2-carboxylate (72)

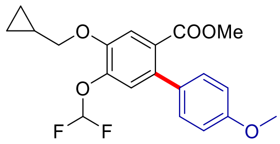

Compound **72** was prepared following the procedure **A**, starting from 3-(cyclopropylmethoxy)-4-(difluoromethoxy)benzoic acid (52.6 mg, 0.20 mmol) and 5-(4-methoxyphenyl)-5*H*-thianthren-5-ium trifluoromethanesulfonate (113.4 mg, 0.24 mmol). After column chromatography on silica (Petroleum Ether: EtOAc = 10:1), **72** was afforded as colorless oil liquid (64.3 mg, 85%).

^{1}H NMR (400 MHz, $CDCl_3$): δ 7.39 (s, 1H), 7.22 – 7.16 (m, 2H), 7.15 (s, 1H), 6.93 – 6.89 (m, 2H), 6.72 (t, *J* = 76.0 Hz, 1H), 3.94 (d, *J* = 6.9 Hz, 2H), 3.84 (s, 3H), 3.65 (s, 3H), 1.36 – 1.28 (m, 1H), 0.71 – 0.64 (m, 2H), 0.41 – 0.35 (m, 2H) ppm.

^{13}C NMR (101 MHz, $CDCl_3$): δ 168.3, 159.0, 148.8, 142.1 (t, $J_{C\text{-}F}$ = 3.0 Hz), 135.9, 132.4, 129.4, 128.1, 124.3, 115.8 (t, $J_{C\text{-}F}$ = 262.6 Hz), 115.7, 113.5, 74.2, 55.2, 52.1, 10.0, 3.2 ppm.

HRMS (ESI-TOF) m/z: $[M+H]^+$ Calcd for $C_{20}H_{21}F_2O_5^+$ 379.1352; Found 379.1350.

Methyl 2-ethoxy-5-(4-methoxyphenyl)-1-((2'-(1-trityl-1*H*-tetrazol-5-yl)-[1,1'-biphenyl]-4-yl)methyl)-1*H*-benzo[d]imidazole-4-carboxylate (73)

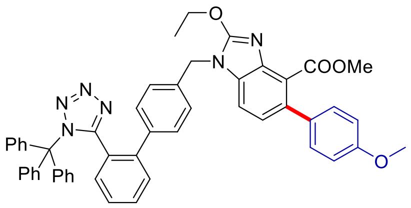

Compound **73** was prepared following the procedure **A**, starting from 2-ethoxy-1-((2'-(1-trityl-1*H*-tetrazol-5-yl)-[1,1'-biphenyl]-4-yl)methyl)-1*H*-benzo[d]imidazole-4-carboxylic acid (139.4 mg, 0.20 mmol) and 5-(4-methoxyphenyl)-5*H*-thianthren-5-ium trifluoromethanesulfonate (113.4 mg, 0.24 mmol). After column chromatography on silica (Petroleum Ether: EtOAc = 3:1), **73** was afforded as white solid (133.2 mg, 83%).

M.P.: 171 – 173 °C.

^{1}H NMR (400 MHz, $CDCl_3$): δ 7.78 – 7.70 (m, 1H), 7.63 – 7.55 (m, 1H), 7.37 – 7.29 (m, 2H), 7.24 – 7.18 (m, 4H), 7.16 – 7.07 (m, 9H), 6.95 – 6.91 (m, 2H), 6.88 – 6.82 (m, 6H), 6.81 – 6.76 (m, 2H), 6.74 – 6.68 (m, 2H), 5.21 (s, 2H), 4.55 (q, *J* = 7.1 Hz, 2H), 3.72 (s, 3H), 2.93 (s, 3H), 1.32 (t, *J* = 7.1 Hz, 3H) ppm.

^{13}C NMR (101 MHz, $CDCl_3$): δ 168.5, 164.0, 158.6, 158.3, 141.6, 141.2, 140.1, 140.0, 135.2, 133.9, 133.7, 130.5, 130.4, 130.2, 129.9, 129.5, 129.4, 128.2, 127.6, 127.6, 127.4, 126.3, 125.5, 123.6, 119.1, 116.2, 113.5, 82.8, 66.7, 55.2, 51.9, 45.7, 14.6 ppm.

HRMS (ESI-TOF) m/z: $[M+H]^+$ Calcd for $C_{51}H_{43}N_6O_4^+$ 803.3340; Found 803.3342.

Methyl 4-((1,7'-dimethyl-2'-propyl-1*H*,3'*H*-[2,5'-bibenzo[d]imidazol]-3'-yl)methyl)-4''-methoxy-[1,1':3',1''-terphenyl]-2'-carboxylate (74)

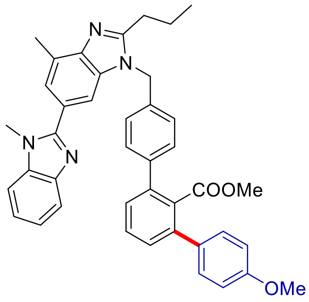

Compound **74** was prepared following the procedure **A**, starting from 4'-((1,7'-dimethyl-2'-propyl-1*H*,3'*H*-[2,5'-bibenzo[d]imidazol]-3'-yl)methyl)-[1,1'-biphenyl]-2-carboxylic acid (105.0 mg, 0.20 mmol) and 5-(4-methoxyphenyl)-5*H*-thianthren-5-ium trifluoromethanesulfonate (113.4 mg, 0.24 mmol) with [Ru(p-cymene)Cl_2]$_2$ (6.2 mg, 0.01 mmol) and P(Cy)$_3$ (11.2 mg, 0.04 mmol). After column chromatography on silica (Petroleum Ether: EtOAc = 1:10, 5% Et_3N in petroleum ether), **74** was afforded as yellow oil liquid (76.1 mg, 60%).

^{1}H NMR (400 MHz, $CDCl_3$): δ 7.80 – 7.75 (m, 1H), 7.51 – 7.48 (m, 1H), 7.47 – 7.43 (m, 1H), 7.42 – 7.39 (m, 1H), 7.37 – 7.31 (m, 4H), 7.30 – 7.25 (m, 5H), 7.11 – 7.07 (m, 2H), 6.93 – 6.88 (m, 2H), 5.43 (s, 2H), 3.82 – 3.78 (m, 6H), 3.29 (s, 3H), 2.94 – 2.89 (m, 2H), 2.76 (s, 3H), 1.90 – 1.80 (m, 3H), 1.06 – 1.01 (m, 3H) ppm.

^{13}C NMR (101 MHz, $CDCl_3$): δ 169.8, 159.1, 156.5, 154.6, 143.1, 142.7, 140.3, 140.0, 139.3, 136.6, 135.1, 135.0, 132.7, 132.6, 129.4, 129.4, 129.1, 129.0, 128.3, 126.2, 123.8, 122.5, 122.4, 119.5, 113.8, 109.5, 109.0, 55.2, 51.7, 47., 31.8, 29.8, 21.8, 16.9, 14.1 ppm.

HRMS (ESI-TOF) m/z: $[M+H]^+$ Calcd for $C_{41}H_{39}N_4O_3^+$ 635.3017; Found 635.3017.

Methyl (*E*)-4-((4-(dimethylamino)phenyl)diazenyl)-4'-methoxy-[1,1'-biphenyl]-2-carboxylate (75)

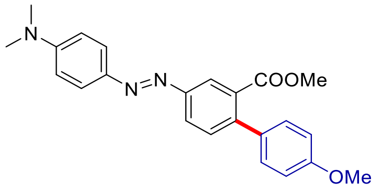

Compound **75** was prepared following the procedure **A**, starting from (*E*)-3-((4-(dimethylamino)phenyl)diazenyl)benzoic acid (53.8 mg, 0.20 mmol) and 5-(4-methoxyphenyl)-5*H*-thianthren-5-ium trifluoromethanesulfonate (113.4 mg, 0.24 mmol) with [Ru(p-cymene)Cl_2]$_2$ (6.2 mg, 0.01 mmol) and P(Cy)$_3$

(11.2 mg, 0.04 mmol). After column chromatography on silica (Petroleum Ether: EtOAc = 5:1), **75** was afforded as yellow solid (14.8 mg, 19%).

M.P.: 134 – 137 °C.

^{1}H NMR (400 MHz, $CDCl_3$): δ 8.31 – 8.24 (m, 1H), 8.01 – 7.96 (m, 1H), 7.91 (d, *J* = 8.7 Hz, 2H), 7.47 (d, *J* = 8.2 Hz, 1H), 7.34 – 7.28 (m, 2H), 6.99 – 6.93 (m, 2H), 6.77 (d, *J* = 8.7 Hz, 2H), 3.86 (s, 3H), 3.73 (s, 3H), 3.10 (s, 6H) ppm.

^{13}C NMR (101 MHz, $CDCl_3$): δ 169.1, 159.1, 152.6, 151.6, 143.6, 142.5, 133.1, 131.4, 131.4, 129.5, 125.2, 124.6, 123.7, 113.6, 111.5, 55.2, 52.1, 40.3 ppm.

HRMS (ESI-TOF) m/z: $[M+H]^+$ Calcd for $C_{23}H_{24}N_3O_3^+$ 390.1812; Found 390.1811.

Methyl 6-(3-(adamantan-1-yl)-4-methoxyphenyl)-3-(4-(4-(2-(pyridin-2-yloxy)propoxy)phenoxy)phenyl)-2-naphthoate (76)

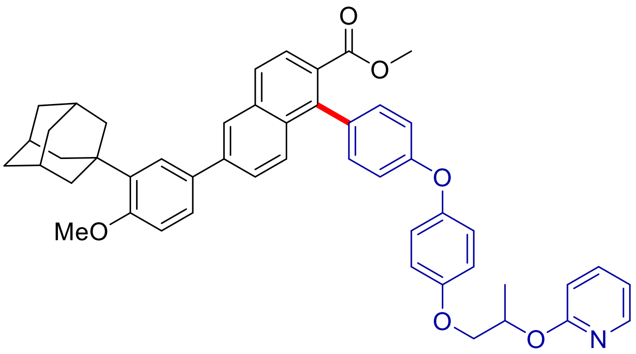

Compound **76** was prepared following the procedure **A**, starting from 6-(3-(adamantan-2-yl)-4-methoxyphenyl)-2-naphthoic acid (82.5 mg, 0.20 mmol) and 5-(4-(4-(2-(pyridin-2-yloxy)propoxy)phenoxy)phenyl)-5H-thianthren-5-ium trifluoromethanesulfonate (164.6 mg, 0.24 mmol). After column chromatography on silica (Petroleum Ether: EtOAc = 5:1), **76** was afforded as white solid (126.7 mg, 85%).

M.P.: 130 – 132 °C.

^{1}H NMR (400 MHz, $CDCl_3$): δ 8.41 (s, 1H), 8.20 – 8.16 (m, 1H), 8.02 – 8.00 (m, 1H), 7.98 – 7.95 (m, 1H), 7.85 (s, 1H), 7.82 – 7.78 (m, 1H), 7.64 – 7.61 (m, 1H), 7.60 – 7.53 (m, 2H), 7.39 – 7.35 (m, 2H), 7.07 – 6.96 (m, 7H), 6.80 – 6.85 (m, 1H), 6.80 – 6.74 (m, 1H), 5.66 – 5.59 (m, 1H), 4.25 – 4.20 (m, 1H), 4.13 – 4.09 (m, 1H), 3.91 (s, 3H), 3.77 (s, 3H), 2.22 – 2.19 (m, 6H), 2.14 – 2.10 (m, 3H), 1.84 – 1.79 (m, 6H), 1.51 (d, *J* = 6.4 Hz, 3H) ppm.

^{13}C NMR (101 MHz, $CDCl_3$): δ 169.0, 163.1, 158.9, 157.8, 155.3, 150.1, 146.7, 141.3, 138.9, 138.7, 138.5, 135.7, 134.8, 132.5, 130.9, 130.2, 129.8, 129.7, 128.9, 128.4, 126.4, 125.9, 125.6, 124.6, 120.8, 117.0, 116.7, 115.8, 112.1, 111.6, 71.0, 69.2, 55.1, 52.0, 40.6, 37.1, 37.1, 29.1, 17.0 ppm.

HRMS (ESI-TOF) m/z: $[M+H]^+$ Calcd for $C_{49}H_{48}NO_6^+$ 746.3476; Found 746.3453.

Methyl 3-ethoxy-2''-fluoro-4''-(1-methoxy-1-oxopropan-2-yl)-5-(2-((3-methyl-1-(2-(piperidin-1-yl)phenyl)butyl)amino)-2-oxoethyl)-[1,1':4',1''-terphenyl]-2-carboxylate (77)

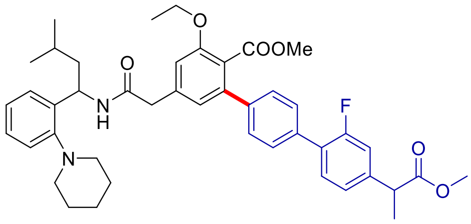

Compound **77** was prepared following the procedure **A**, starting from 2-ethoxy-4-(2-((3-methyl-1-(2-(piperidin-1-yl)phenyl)butyl)amino)-2-oxoethyl)benzoic acid (91.4 mg, 0.20 mmol) and 5-(2-fluoro-4'-(1-methoxy-1-oxopropan-2-yl)-[1,1'-biphenyl]-4-yl)-5H-thianthren-5-ium trifluoromethanesulfonate (149.3 mg, 0.24 mmol). After column chromatography on silica (Petroleum Ether: EtOAc = 3:1), **77** was afforded as colorless oil liquid (134.4 mg, 93%).

^{1}H NMR (400 MHz, $CDCl_3$): δ 7.56 – 7.52 (m, 2H), 7.44 – 7.37 (m, 3H), 7.21 – 7.10 (m, 5H), 7.07 – 7.03 (m, 1H), 6.88 – 6.82 (m, 2H), 6.80 – 6.71 (m, 1H), 5.42 – 5.35 (m, 1H), 4.10 – 3.97 (m, 2H), 3.80 – 3.74 (m, 1H), 3.71 (s, 3H), 3.65 (s, 3H), 3.57 (s, 2H), 2.93 (s, 2H), 2.69 – 2.52 (m, 2H), 1.67 - 1.52 (m, 11H), 1.40 – 1.43 (m, 1H), 1.38 (t, J =1.8 Hz), 0.94 – 0.90 (m, 6H) ppm.

^{13}C NMR (101 MHz, $CDCl_3$): δ 174.4, 169.0, 168.3, 159.7 (d, $J_{C\text{-}F}$ = 249.5 Hz), 156.3, 152.5, 141.9 (d, $J_{C\text{-}F}$ = 9.1 Hz), 141.0, 139.1, 138.7, 138.0, 134.8, 130.7 (d, $J_{C\text{-}F}$ = 4.0 Hz), 128.9 (d, $J_{C\text{-}F}$ = 3.0 Hz), 128.2, 127.9, 127.8, 127.2 (d, $J_{C\text{-}F}$ = 13.1 Hz), 125.1, 123.6 (d, $J_{C\text{-}F}$ = 3.0 Hz), 122.9, 122.6, 122.2, 115.3 (d, $J_{C\text{-}F}$ = 24.2 Hz), 111.8, 64.6, 52.2, 52.1, 49.9, 46.6, 44.9, 44.2, 26.7, 25.3, 24.1, 22.8, 22.5, 18.4, 14.6 ppm.

HRMS (ESI-TOF) m/z: $[M+H]^+$ Calcd for $C_{44}H_{52}FN_2O_6^+$ 723.3804; Found 723.3800.

General Procedure B: An oven-dried 10 mL vessel was charged with arenecarboxylic acids (0.20 mmol, 1 equiv.), and arylthianthrenium salts (0.24 mmol, 1.2 equiv.), $[Ru(p\text{-cymene})Cl_2]_2$ (3.2 mg, 5.00 μmol, 0.025 equiv.), K_2CO_3 (33.6 mg, 0.24 mmol, 1.2 equiv.), $P(Cy)_3$ (5.6 mg, 0.02 mmol, 0.1 equiv.). Under exclusion of air,

NMP (4 mL) was added via syringe. The resulting reaction mixture was stirred at rt for 24 h. And then Cu_2O (28.6 mg, 0.2 mmol, 1 equiv.) were added at argon atmosphere and the mixtures were stirred at 195 °C for 12 h. Brine (10 mL) was added and the resulting mixture was extracted with ethyl acetate (3×20 mL). The combined organic layers were dried over MgSO4, filtered, and the volatiles were removed under reduced pressure. The residue was purified by column chromatography (SiO_2, ethyl acetate/cyclohexane gradient), affording the corresponding products.

3,4'-dimethoxy-1,1'-biphenyl (78)

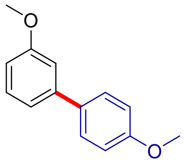

Compound **78** was prepared following the procedure **B**, starting from 2-methoxybenzoic acid (30.6 mg, 0.20 mmol) and 5-(4-methoxyphenyl)-5*H*-thianthren-5-ium trifluoromethanesulfonate (13.4 mg, 0.24 mmol). After column chromatography on silica (Petroleum Ether: EtOAc = 40:1), **78** was afforded as white solid (21.4 mg, 50%).

M.P.: 56 – 58 °C.

^{1}H NMR (400 MHz, $CDCl_3$): δ 7.57 – 7.51 (m, 2H), 7.37 – 7.32 (m, 1H), 7.18 – 7.14 (m, 1H), 7.13 – 7.08 (m, 1H), 7.01 – 6.96 (m, 2H), 6.90 – 6.84 (m, 1H), 3.87 (s, 3H), 3.86 (s, 3H) ppm.

^{13}C NMR (101 MHz, $CDCl_3$): δ 159.9, 159.2, 142.3, 133.6, 129.7, 128.2, 119.3, 114.1, 112.5, 112.0, 55.3, 55.2 ppm.

Spectra data are consistent with the reported literature.[8]

4,4'-dimethoxy-1,1'-biphenyl (79)

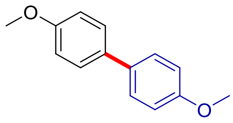

Compound **79** was prepared following the procedure **B**, starting from 3-methoxybenzoic acid (31.0 mg, 0.20 mmol) and 5-(4-methoxyphenyl)-5*H*-thianthren-5-ium trifluoromethanesulfonate (113.4 mg, 0.24 mmol). After

column chromatography on silica (Petroleum Ether: EtOAc = 10:1), **79** was afforded as white solid (28.7 mg, 67%).

M.P.: 171 – 174 °C.

^{1}H NMR (400 MHz, $CDCl_3$): δ 7.53 – 7.44 (m, 4H), 7.01 – 6.92 (m, 4H), 3.85 (s, 6H) ppm.

^{13}C NMR (101 MHz, $CDCl_3$): δ 158.7, 133.5, 127.7, 114.1, 55.3 ppm.

Spectra data are consistent with the reported literature.[9]

3-fluoro-4'-methoxy-1,1'-biphenyl (80)

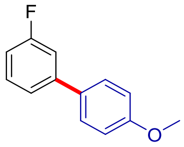

Compound **80** was prepared following the procedure **B**, starting from 2-fluorobenzoic acid (28.0 mg, 0.20 mmol) and 5-(4-methoxyphenyl)-5*H*-thianthren-5-ium trifluoromethanesulfonate (113.4 mg, 0.24 mmol) with [Ru(p-cymene)Cl_2]$_2$ (6.2 mg, 0.01 mmol) and P(Cy)$_3$ (11.2 mg, 0.04 mmol). After column chromatography on silica (Petroleum Ether: EtOAc = 20:1), **80** was afforded as white solid (28.3 mg, 70%)

M.P.: 65 – 67 °C.

^{1}H NMR (400 MHz, $CDCl_3$): δ 7.54 – 7.49 (m, 2H), 7.40 – 7.31 (m, 2H), 7.28 – 7.23 (m, 1H), 7.03 – 6.95 (m, 3H), 3.86 (s, 3H) ppm.

^{13}C NMR (101 MHz, $CDCl_3$): δ 163.2 (d, $J_{C\text{-}F}$ = 224.2 Hz), 159.5, 143.1 (d, $J_{C\text{-}F}$ = 8.1 Hz), 132.4 (d, $J_{C\text{-}F}$ = 2.0 Hz), 130.1 (d, $J_{C\text{-}F}$ = 9.1 Hz), 128.1, 122.2 (d, $J_{C\text{-}F}$ = 3.0 Hz), 114.3, 113.5 (d, $J_{C\text{-}F}$ = 12.1 Hz), 113.3 (d, $J_{C\text{-}F}$ = 11.1 Hz), 55.3 ppm.

Spectra data are consistent with the reported literature.[8]

1-(4-methoxyphenyl)naphthalene (81)

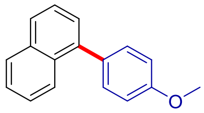

Compound **81** was prepared following the procedure **B**, starting from 2-naphthoic acid (34.8 mg, 0.20 mmol) and 5-(4-methoxyphenyl)-5*H*-thianthren-5-ium trifluoromethanesulfonate (113.4 mg, 0.24 mmol). After column chromatography on silica (Petroleum Ether: EtOAc = 20:1), **81** was afforded as yellow solid (36.1 mg, 77%).

M.P.: 113 – 116 °C.

^{1}H NMR (400 MHz, $CDCl_3$): δ 8.01 (s, 1H), 7.93 – 7.85 (m, 3H), 7.76 – 7.72 (m, 1H), 7.70 – 7.65 (m, 2H), 7.53 – 7.45 (m, 2H), 7.07 – 7.01 (m, 2H), 3.89 (s, 3H) ppm.

^{13}C NMR (101 MHz, $CDCl_3$): δ 159.2, 138.1, 133.7, 133.6, 132.3, 128.4, 128.3, 128.0, 127.6, 126.2, 125.6, 125.4, 125.0, 114.3, 55.4 ppm.

Spectra data are consistent with the reported literature.[9]

4-methoxy-1,1':3',1''-terphenyl (82)

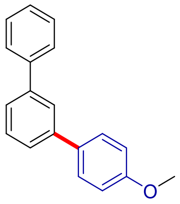

Compound **82** was prepared following the procedure **B**, starting from [1,1'-biphenyl]-2-carboxylic acid (39.8 mg, 0.20 mmol) and 5-(4-methoxyphenyl)-5*H*-thianthren-5-ium trifluoromethanesulfonate (113.4 mg, 0.24 mmol). After column chromatography on silica (Petroleum Ether: EtOAc = 40:1), **82** was afforded as white solid (15.6 mg, 30%).

M.P.: 134 – 136 °C.

^{1}H NMR (400 MHz, $CDCl_3$): δ 7.80 – 7.76 (m, 1H), 7.68 – 7.64 (m, 2H), 7.62 – 7.58 (m, 2H), 7.57 – 7.53 (m, 2H), 7.52 – 7.45 (m, 3H), 7.40 – 7.35 (m, 1H), 7.05 – 6.98 (m, 2H), 3.87 (s, 3H) ppm.

^{13}C NMR (101 MHz, $CDCl_3$): δ 159.2, 141.7, 141.4, 141.3, 133.7, 129.1, 128.8, 128.2, 127.3, 127.3, 125.7, 125.7, 125.5, 114.2, 55.4 ppm.

Spectra data are consistent with the reported literature.[8]

4'-methoxy-[1,1'-biphenyl]-3-ol (83)

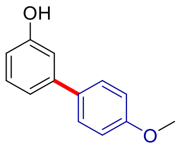

Compound **83** was prepared following the procedure **B**, starting from acetylsalicylic acid (36.1 mg, 0.20 mmol) and 5-(4-methoxyphenyl)-5*H*-thianthren-5-ium trifluoromethanesulfonate (113.4 mg, 0.24 mmol) with [Ru(p-

cymene)Cl_2]$_2$ (6.2 mg, 0.01 mmol) and P(Cy)$_3$ (11.2 mg, 0.04 mmol). After column chromatography on silica (Petroleum Ether: EtOAc = 10:1), **83** was afforded as white solid (22.4 mg, 56%).

M.P.: 116 – 118 °C.

^{1}H NMR (400 MHz, $CDCl_3$): δ 7.51 – 7.48 (m, 2H), 7.31 – 7.27 (m, 1H), 7.14 – 7.12 (m, 1H), 7.03 (s, 1H), 6.98 – 6.95 (m, 1H), 6.80 – 6.78 (m, 1H), 5.22 (s, 1H), 3.85 (s, 3H) ppm.

^{13}C NMR (101 MHz, $CDCl_3$): δ 159.2, 155.8, 142.5, 133.3, 129.9, 128.1, 119.3, 114.2, 113.63, 113.60, 55.4 ppm.

HRMS (ESI-TOF) m/z: $[M+H]^+$ Calcd for $C_{13}H_{13}O_2^+$ 201.0910; Found 201.0988.

3-(4-methoxyphenyl)thiophene (84)

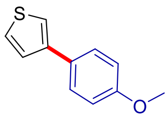

Compound **84** was prepared following the procedure **B**, starting from thiophene-2-carboxylic acid (25.6 mg, 0.20 mmol) and 5-(4-methoxyphenyl)-5*H*-thianthren-5-ium trifluoromethanesulfonate (113.4 mg, 0.24 mmol). After column chromatography on silica (Petroleum Ether: EtOAc = 40:1), **84** was afforded as white solid (22.8 mg, 60%).

M.P.: 126 – 128 °C.

^{1}H NMR (400 MHz, $CDCl_3$): δ 7.58 – 7.51 (m, 2H), 7.41 – 7.32 (m, 3H), 7.00 – 6.91 (m, 2H), 3.85 (s, 3H) ppm.

^{13}C NMR (101 MHz, $CDCl_3$): δ 158.8, 142.0, 128.7, 127.5, 126.2, 126.0, 118.9, 114.1, 55.3 ppm.

Spectra data are consistent with the reported literature.[8]

3-methoxy-1,1':4',1''-terphenyl (85)

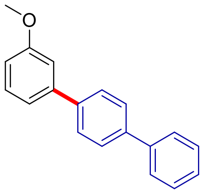

Compound **85** was prepared following the procedure **B**, starting from 2-methoxybenzoic acid (30.6 mg, 0.20 mmol) and 5-([1,1'-biphenyl]-4-yl)-5*H*-thianthren-5-ium trifluoromethanesulfonate (124.4 mg, 0.24 mmol). After column chromatography on silica (Petroleum Ether), **85** was afforded as white solid (46.8 mg, 90%).

M.P.: 104 – 105 °C.

^{1}H NMR (400 MHz, $CDCl_3$): δ 7.69 (s, 4H), 7.68 – 7.65 (m, 2H), 7.50 – 7.45 (m, 2H), 7.42 – 7.35 (m, 2H), 7.27 – 7.24 (m, 1H), 7.22 – 7.19 (m, 1H), 6.96 – 6.90 (m, 1H), 3.89 (s, 3H) ppm.

^{13}C NMR (101 MHz, $CDCl_3$): δ 160.0, 142.2, 140.7, 140.3, 140.0, 129.8, 128.8, 127.5, 127.4, 127.3, 127.0, 119.6, 112.8, 112.7, 55.3 ppm.

Spectra data are consistent with the reported literature.[8]

3',4-dimethoxy-3,5-dimethyl-1,1'-biphenyl (86)

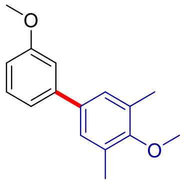

Compound **86** was prepared following the procedure **B**, starting from 2-methoxybenzoic acid (30.6 mg, 0.20 mmol) and 5-(4-methoxy-3,5-dimethylphenyl)-5*H*-thianthren-5-ium trifluoromethanesulfonate (120.2 mg, 0.24 mmol). After column chromatography on silica (Petroleum Ether: EtOAc = 100:1), **86** was afforded as light green oil liquid (29.1 mg, 60%).

^{1}H NMR (400 MHz, $CDCl_3$): δ 7.36 – 7.30 (m, 1H), 7.26 – 7.24 (m, 2H), 7.17 – 7.13 (m, 1H), 7.11 – 7.08 (m, 1H), 6.90 – 6.85 (m, 1H), 3.87 (s, 3H), 3.78 (s, 3H), 2.37 (s, 6H) ppm.

^{13}C NMR (101 MHz, $CDCl_3$): δ 159.8, 156.6, 142.5, 136.6, 131.1, 129.6, 127.6, 119.5, 112.6, 112.3, 59.7, 55.3, 16.2 ppm.

HRMS (ESI-TOF) m/z: $[M+H]^+$ Calcd for $C_{16}H_{19}O_2^+$ 243.1380; Found 243.1375.

4'-fluoro-3-methoxy-1,1'-biphenyl (87)

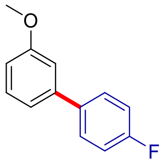

Compound **87** was prepared following the procedure **B**, starting from 2-methoxybenzoic acid (30.6 mg, 0.20 mmol) and 5-(4-fluorophenyl)-5*H*-thianthren-5-ium trifluoromethanesulfonate (110.6 mg, 0.24 mmol). After column chromatography on silica (Petroleum Ether), **87** was afforded as white solid (21.0 mg, 52%).

M.P.: 76 – 78 °C.

^{1}**H NMR** (400 MHz, $CDCl_3$): δ 7.58 – 7.52 (m, 2H), 7.38 – 7.33 (m, 1H), 7.16 – 7.06 (m, 4H), 6.93 – 6.87 (m, 1H), 3.87 (s, 3H) ppm.

^{13}**C NMR** (101 MHz, $CDCl_3$): δ 162.5 (d, $J_{C\text{-}F}$ = 247.5 Hz), 159.9, 141.7, 137.2 (d, $J_{C\text{-}F}$ = 3.0 Hz), 129.8, 128.7 (d, $J_{C\text{-}F}$ = 8.1 Hz), 119.5, 115.6 (d, $J_{C\text{-}F}$ = 21.2 Hz), 112.8, 112.5, 55.3 ppm.

Spectra data are consistent with the reported literature.[10]

4'-chloro-3-methoxy-1,1'-biphenyl (88)

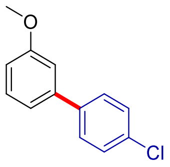

Compound **88** was prepared following the procedure **B**, starting from 2-methoxybenzoic acid (30.6 mg, 0.20 mmol) and 5-(4-chlorophenyl)-5*H*-thianthren-5-ium trifluoromethanesulfonate (114.2 mg, 0.24 mmol). After column chromatography on silica (Petroleum Ether), **88** was afforded as yellow oil liquid (21.8 mg, 50%).

^{1}**H NMR** (400 MHz, $CDCl_3$): δ 7.55 – 7.48 (m, 2H), 7.43 – 7.33 (m, 3H), 7.16 – 7.12 (m, 1H), 7.10 – 7.05 (m, 1H), 6.96 – 6.87 (m, 1H), 3.87 (s, 3H) ppm.

^{13}**C NMR** (101 MHz, $CDCl_3$): δ 160.0, 141.5, 139.5, 133.5, 129.9, 128.9, 128.4, 119.5, 112.9, 112.8, 55.3 ppm.

Spectra data are consistent with the reported literature.[10]

3-chloro-3',4-dimethoxy-1,1'-biphenyl (89)

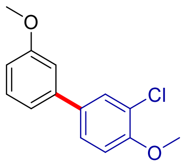

Compound **89** was prepared following the procedure **B**, starting from 2-methoxybenzoic acid (30.6 mg, 0.20 mmol) and 5-(3-chloro-4-methoxyphenyl)-5*H*-thianthren-5-ium trifluoromethanesulfonate (121.6 mg, 0.24 mmol). After column chromatography on silica (Petroleum Ether: EtOAc = 20:1), **89** was afforded as colorless oil liquid (35.2 mg, 71%).

^{1}**H NMR** (400 MHz, $CDCl_3$): δ 7.64 – 7.59 (m, 1H), 7.47 – 7.42 (m, 1H), 7.36 – 7.31 (m, 1H), 7.14 – 7.09 (m, 1H), 7.08 – 7.03 (m, 1H), 7.01 – 6.96 (m, 1H), 6.91 – 6.85 (m, 1H), 3.94 (s, 3H), 3.86 (s, 3H) ppm.

^{13}C NMR (101 MHz, $CDCl_3$): δ 160.0, 154.5, 141.0, 134.5, 129.8, 128.9, 126.3, 122.7, 119.2, 112.6, 112.5, 112.2, 56.2, 55.3 ppm.

Spectra data are consistent with the reported literature.[10]

4-bromo-4'-methoxy-1,1'-biphenyl (90)

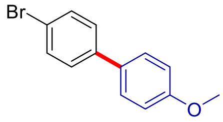

Compound **90** was prepared following the procedure **B**, starting from 2-bromobenzoic acid (39.9 mg, 0.20 mmol) and 5-(4-methoxyphenyl)-5*H*-thianthren-5-ium trifluoromethanesulfonate (113.4 mg, 0.24 mmol) with [Ru(p-cymene)Cl_2]$_2$ (6.2 mg, 0.01 mmol) and P(Cy)$_3$ (11.2 mg, 0.04 mmol). After column chromatography on silica (Petroleum Ether: EtOAc = 50:1), **90** was afforded as white solid (21.9 mg, 42%).

M.P.: 143 – 145 °C.

^{1}H NMR (400 MHz, $CDCl_3$): δ 7.57 – 7.46 (m, 4H), 7.44 – 7.39 (m, 2H), 7.02 – 6.94 (m, 2H), 3.85 (s, 3H) ppm.

^{13}C NMR (101 MHz, $CDCl_3$): δ 159.4, 139.7, 132.5, 131.8, 128.3, 128.0, 120.8, 114.3, 55.4 ppm.

HRMS (ESI-TOF) m/z: $[M+H]^+$ Calcd for $C_{13}H_{12}BrO^+$ 263.0066; Found 263.0066.

4'-bromo-3-methoxy-1,1'-biphenyl (91)

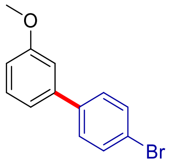

Compound **91** was prepared following the procedure **B**, starting from 2-bromobenzoic acid (30.5 mg, 0.20 mmol) and 5-(4-bromophenyl)-5*H*-thianthren-5-ium trifluoromethanesulfonate (124.8 mg, 0.24 mmol). After column chromatography on silica (Petroleum Ether: EtOAc = 50:1), **91** was afforded as colorless oil liquid (26.6 mg, 51%).

^{1}H NMR (400 MHz, $CDCl_3$): δ 7.60 – 7.53 (m, 2H), 7.45 (d, *J* = 8.3 Hz, 2H), 7.36 (t, *J* = 7.9 Hz, 1H), 7.14 (d, *J* = 7.6 Hz, 1H), 7.08 (s, 1H), 6.95 – 6.88 (m, 1H), 3.87 (s, 3H) ppm.

^{13}C NMR (101 MHz, $CDCl_3$): δ 160.0, 141.5, 140.0, 131.8, 129.9, 128.8, 121.6, 119.4, 112.9, 112.8, 55.3 ppm.

HRMS (ESI-TOF) m/z: $[M+H]^+$ Calcd for $C_{13}H_{12}BrO^+$ 263.0066; Found 263.0068.

4'-iodo-3-methoxy-1,1'-biphenyl (92)

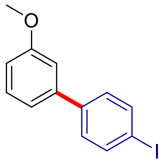

Compound **92** was prepared following the procedure **B**, starting from 2-methoxybenzoic acid (30.6 mg, 0.20 mmol) and 5-(4-iodophenyl)-5H-thianthren-5-ium trifluoromethanesulfonate (136.4 mg, 0.24 mmol). After column chromatography on silica (Petroleum Ether), **92** was afforded as colorless oil liquid (21.1 mg, 34%).

^{1}H NMR (400 MHz, $CDCl_3$): δ 7.66 – 7.56 (m, 2H), 7.50 – 7.42 (m, 2H), 7.39 – 7.37 (m, 1H), 7.24 – 7.18 (m, 1H), 7.18 – 7.13 (m, 1H), 6.96 – 6.88 (m, 1H), 3.89 (s, 3H) ppm.

^{13}C NMR (101 MHz, $CDCl_3$): δ 159.9, 142.8, 141.1, 129.7, 128.7, 127.4, 127.2, 119.7, 112.9, 112.6, 55.3 ppm.

Spectra data are consistent with the reported literature.[8]

2-((1-(4-((3'-fluoro-[1,1'-biphenyl]-4-yl)oxy)phenoxy)propan-2-yl)oxy)pyridine (93)

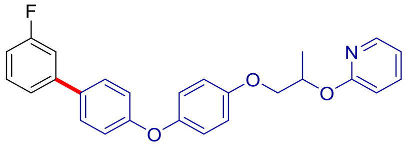

Compound **93** was prepared following the procedure **B**, starting from 2-fluorobenzoic acid (27.8 mg, 0.20 mmol) and 5-(4-(4-(2-(pyridin-2-yloxy)propoxy)phenoxy)phenyl)-5*H*-thianthren-5-ium trifluoromethanesulfonate (164.6 mg, 0.24 mmol) with [Ru(p-cymene)Cl_2]$_2$ (6.2 mg, 0.01 mmol) and P(Cy)$_3$ (11.2 mg, 0.04 mmol). And then with Ag_2CO_3 (27.6 mg, 0.1 mmol) at 160 °C for decarboxylation. After column chromatography on silica (Petroleum Ether: EtOAc = 10:1), **93** was afforded as colorless oil liquid (38.0 mg, 46%).

^{1}H NMR (400 MHz, $CDCl_3$): δ 8.16 (d, *J* = 4.0 Hz, 1H), 7.57 (t, *J* = 7.5 Hz, 1H), 7.50 (d, *J* = 8.3 Hz, 2H), 7.40 – 7.32 (m, 2H), 7.26 – 7.23 (m, 1H), 7.02 – 6.95 (m, 7H), 6.87 (t, *J* = 5.8 Hz, 1H), 6.76 (d, *J* = 8.3 Hz, 1H), 5.64 – 5.57 (m, 1H), 4.21 (dd, *J* = 9.6, 5.3 Hz, 1H), 4.09 (dd, *J* = 9.7, 4.7 Hz, 1H), 1.50 (d, *J* = 6.3 Hz, 3H) ppm.

^{13}C NMR (101 MHz, $CDCl_3$): δ 163.2 (d, J_{C-F} = 246.4 Hz), 163.1, 158.6, 155.4, 149.9, 146.8, 142.9 (d, J_{C-F} = 8.1 Hz), 138.7, 134.1 (d, J_{C-F} = 2.3 Hz), 130.2 (d, J_{C-F} = 8.5 Hz), 128.3, 122.4 (d, J_{C-F} = 2.7 Hz), 120.9, 117.7, 116.8, 115.9, 113.7, 113.5, 111.7, 71.1, 69.2, 17.0 ppm.

HRMS (ESI-TOF) m/z: $[M+H]^+$ Calcd for $C_{26}H_{23}FNO_3^+$ 416.1656; Found 416.1666.

methyl 6-(3-((3*r*,5*r*,7*r*)-adamantan-1-yl)-4-methoxyphenyl)-1-(4'-ethyl-2'-fluoro-[1,1'-biphenyl]-4-yl)-2-naphthoate (94)

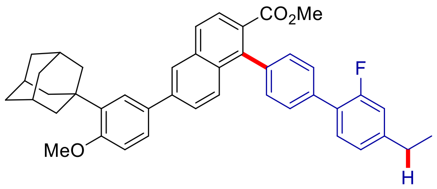

Compound **94** was prepared following the procedure **B**, starting from 6-(3-(adamantan-2-yl)-4-methoxyphenyl)-2-naphthoic acid (83.4 mg, 0.20 mmol) and 5-(2-fluoro-4'-(1-methoxy-1-oxopropan-2-yl)-[1,1'-biphenyl]-4-yl)-5*H*-thianthren-5-ium trifluoromethanesulfonate (149.2 mg, 0.24 mmol). After column chromatography on silica (Petroleum Ether: EtOAc = 10:1), **94** was afforded as white solid (49.9 mg, 40%).

M.P.: 98 – 101 °C.

^{1}H NMR (400 MHz, $CDCl_3$): δ 8.43 (s, 1H), 8.03 – 7.97 (m, 2H), 7.91 (s, 1H), 7.83 – 7.79 (m, 1H), 7.65 – 7.61 (m, 3H), 7.57 – 7.53 (m, 1H), 7.51 – 7.42 (m, 3H), 7.09 – 6.99 (m, 3H), 3.90 (s, 3H), 3.74 (s, 3H), 2.71 (q, *J* = 7.2, 6.7 Hz, 2H), 2.20 (s, 6H), 2.11 (s, 3H), 1.81 (s, 6H), 1.32 – 1.27 (m, 3H) ppm.

^{13}C NMR (101 MHz, $CDCl_3$): δ 169.0, 159.8 (d, $J_{C\text{-}F}$ = 248.5 Hz), 158.9, 145.9 (d, $J_{C\text{-}F}$ = 7.1 Hz), 141.4, 140.6, 139.0, 138.8, 134.9, 134.6, 132.5, 131.0, 130.4 (d, $J_{C\text{-}F}$ = 4.0 Hz), 130.3, 130.0, 128.9, 128.6, 128.5 (d, $J_{C\text{-}F}$ = 3.0 Hz), 128.4, 126.6, 126.0, 125.8 (d, $J_{C\text{-}F}$ = 12.1 Hz), 125.7, 124.8, 123.8 (d, $J_{C\text{-}F}$ = 3.0 Hz), 115.4 (d, $J_{C\text{-}F}$ = 22.2 Hz), 112.1, 55.1, 52.1, 40.6, 37.2, 37.1, 29.1, 28.4, 15.2 ppm.

HRMS (ESI-TOF) m/z: $[M+H]^+$ Calcd for $C_{43}H_{42}FO_3^+$ 625.3112; Found 625.3109.

Gram-scale Reaction

General Procedure C: An oven-dried vessel was charged with $[Ru(p\text{-cymene})Cl_2]_2$ (80.0 mg, 0.125 mmol), K_2CO_3 (840.0 mg, 6.0 mmol), P(Cy)3 (140.2 mg, 0.5 mmol), arenecarboxylic acids (5.0 mmol), and arylthianthrenium salts (6.0 mmol). Under exclusion of air, NMP (100 mL) was added via syringe. The resulting reaction mixture was stirred at rt for 24 h. 100 mL of K_2CO_3 (1.38 g, 10 mmol) solution were added and the mixture was stirred at rt for 12 h. Brine (100 mL) was added and the resulting mixture was extracted with ethyl acetate (3×100 mL) to remove most of NMP. The inorganic phase was acidified with 1 mol/L hydrochloric acid (HCl) to adjust the pH to 1-2. The mixture was then extracted with ethyl acetate (3 × 100 mL). The combined organic layers were dried over $MgSO_4$, filtered, and the volatiles were removed under reduced pressure. The

residue was purified by column chromatography (SiO2, Petroleum Ether: EtOAc = 3:1, 2.5% formic acid and 2.5% methanol in ethyl acetate), **12** was selected as an example and was afforded as yellow solid (1.06 g, 88%).

4'-methoxy-4-methyl-[1,1'-biphenyl]-2-carboxylic acid (12)

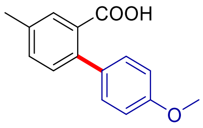

M.P.: 108 – 109 °C.

^{1}H NMR (400 MHz, $CDCl_3$): δ 9.49 (bs, 1H), 7.76 – 7.72 (m, 1H), 7.37 – 7.32 (m, 1H), 7.27 – 7.23 (m, 3H), 6.93 – 6.89 (m, 2H), 3.84 (s, 3H), 2.41 (s, 3H) ppm.

^{13}C NMR (101 MHz, $CDCl_3$): δ 173.8, 158.9, 140.1, 136.7, 133.3, 132.8, 131.1, 129.6, 129.1, 113.5, 55.2, 20.8 ppm.

Spectra data are consistent with the reported literature.[3]

Further Derivatization

3-methoxy-8-methyl-6H-benzo[c]chromen-6-one (95)[11]

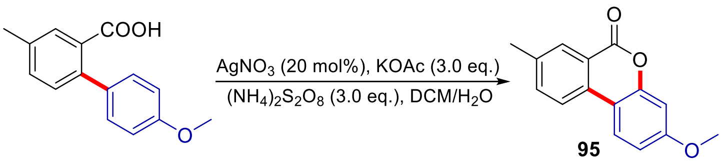

An oven-dried vessel was charged with $(NH_4)_2S_2O_8$ (136.9 mg, 0.60 mmol, 3 equiv.), $AgNO_3$ (6.8 mg, 0.04 mol, 0.2 equiv.), 4'-methoxy-4-methyl-[1,1'-biphenyl]-2-carboxylic acid (48.4 mg, 0.20 mmol, 1 equiv.), KOAc (59.5 mg, 0.60 mmol, 3 equiv.). Under exclusion of air, 2.0 mL of DCM/H_2O (1:1) was added via syringe. The mixture was stirred at room temperature for 12 h. The resulting mixture was extracted with ethyl acetate (10 mL × 3), and the combined organic layers were washed with brine (10 mL), dried over anhydrous Na_2SO_4, filtered and concentrated. The residue was purified by flash chromatography on silica gel with petroleum ether/ethyl acetate to afford the desired product **95** as white oil liquid (28.8 mg, 60%).

^{1}H NMR (400 MHz, $CDCl_3$): δ 8.07 (s, 1H), 7.84 – 7.79 (m, 2H), 7.54 – 7.48 (m, 1H), 6.87 – 6.74 (m, 2H), 3.80 (s, 3H), 2.40 (s, 3H) ppm.

^{13}C NMR (101 MHz, $CDCl_3$): δ 161.6, 161.1, 152.3, 137.9, 136.1, 132.6, 130.2, 123.5, 121.0, 119.8, 112.3, 111.3, 101.6, 55.6, 21.2 ppm.

Spectra data are consistent with the reported literature.[12]

(4'-methoxy-4-methyl-[1,1'-biphenyl]-2-yl)methanol (96)

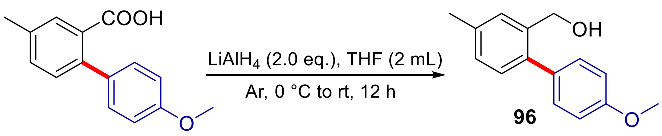

4'-methoxy-4-methyl-[1,1'-biphenyl]-2-carboxylic acid (48.4 mg, 0.2 mmol, 1 equiv.), THF (3 mL) was added to an oven-dried vessel under Ar and cooled to 0 °C, $LiAlH_4$ (15.2 mg, 0.4 mmol, 2 equiv.) was slowly added to the solution, the reaction was allowed to warm to room temperature stirred overnight. The resulting mixture was extracted with ethyl acetate (10 mL × 3), and the combined organic layers were washed with brine (10 mL), dried over anhydrous Na_2SO_4, filtered and concentrated. The residue was purified by flash chromatography on silica gel with petroleum ether/ethyl acetate to afford the desired product **96** as a white solid (34.2 mg, 75%).

M.P.: 97 – 98 °C.

^{1}H NMR (400 MHz, $CDCl_3$): δ 7.35 (s, 1H), 7.32 – 7.27 (m, 2H), 7.19 – 7.14 (m, 2H), 6.98 – 6.93 (m, 2H), 4.60 (s, 2H), 3.85 (s, 3H), 2.41 (s, 3H) ppm.

^{13}C NMR (101 MHz, $CDCl_3$): δ 158.8, 138.1, 137.9, 137.1, 133.0, 130.3, 130.2, 129.2, 128.4, 113.7, 63.3, 55.3, 21.1 ppm.

Spectra data are consistent with the reported literature.[13]

4'-methoxy-4-methyl-[1,1'-biphenyl]-2-carbonitrile (97) [14]

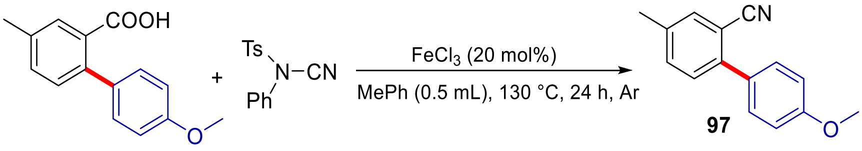

An oven-dried vessel was charged with $FeCl_3$ (6.5 mg, 0.04 mmol, 0.2 equiv.), 4'-methoxy-4-methyl-[1,1'-biphenyl]-2-carboxylic acid (48.4 mg, 0.2 mmol, 1 equiv.), and *N*-cyano-4-methyl-*N*-phenylbenzenesulfonamide (81.6 mg, 0.30 mmol, 1.5 equiv.). Under an argon atmosphere, toluene (0.5 mL) was added via syringe. The resulting mixture was stirred at 130 °C for 24 h. The resulting mixture was extracted with ethyl acetate (10 mL

× 3), and the combined organic layers were washed with brine (10 mL), dried over anhydrous Na_2SO_4, filtered and concentrated. The residue was purified by flash chromatography on silica gel with petroleum ether/ethyl acetate to afford the desired product **97** as a white solid (33.9 mg, 76%).

M.P.: 113 – 115 °C.

^{1}H NMR (400 MHz, $CDCl_3$): δ 7.55 – 7.51 (m, 1H), 7.50 – 7.46 (m, 2H), 7.43 – 7.39 (m, 1H), 7.38 – 7.35 (m, 1H), 7.02 – 6.98 (m, 2H), 3.85 (s, 3H), 2.40 (s, 3H) ppm.

^{13}C NMR (101 MHz, $CDCl_3$): δ 159.8, 142.3, 137.1, 133.9, 133.7, 130.4, 129.9, 129.7, 119.1, 114.1, 110.7, 55.3, 20.6 ppm.

Spectra data are consistent with the reported literature.[15]

Methyl 4'-methoxy-3-((1-phenylethyl)carbamoyl)-[1,1'-biphenyl]-2-carboxylate (98)

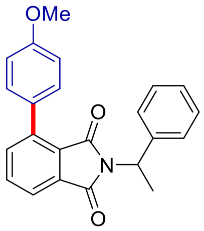

Compound **98** was prepared following the procedure **A**, starting from 2-((1-phenylethyl)carbamoyl)benzoic acid (55.0 mg, 0.20 mmol, 1 equiv.) and 5-(4-methoxyphenyl)-5*H*-thianthren-5-ium trifluoromethanesulfonate (113.4 mg, 0.24 mmol, 1.2 equiv.) with [Ru(p-cymene)Cl_2]$_2$ (6.2 mg, 0.01 mmol, 0.05 equiv.) and P(Cy)$_3$ (11.2 mg, 0.04 mmol, 0.2 equiv.). Under exclusion of air, NMP (4 mL) was added via syringe. The resulting reaction mixture was stirred at 60 °C for 24 h. MeCN (2 mL), K_2CO_3 (83.0 mg, 0.6 mmol, 3 equiv.) were added and the mixtures were stirred at 60 °C for 2.5 h. Brine (10 mL) was added and the resulting mixture was extracted with ethyl acetate (3×20 mL). The combined organic layers were dried over $MgSO_4$, filtered, and the volatiles were removed under reduced pressure. The residue was purified by column chromatography (SiO_2, ethyl acetate/cyclohexane gradient). After column chromatography on silica (Petroleum Ether: EtOAc = 1:1, 2.5% formic acid and 2.5% methanol in ethyl acetate), **98** was afforded as yellow oil liquid (62.2 mg, 87%).

^{1}H NMR (400 MHz, $CDCl_3$): δ 7.79 – 7.73 (m, 1H), 7.69 – 7.64 (m, 1H), 7.61 – 7.56 (m, 1H), 7.54 – 7.47 (m, 4H), 7.34 – 7.28 (m, 2H), 7.27 – 7.22 (m, 1H), 7.05 – 6.95 (m, 2H), 5.56 (q, *J* = 7.4 Hz, 1H), 3.87 (s, 3H), 1.92 (d, *J* = 7.3 Hz, 3H) ppm.

^{13}C NMR (101 MHz, $CDCl_3$): δ 167.8, 167.7, 160.0, 140.7, 140.3, 135.9, 133.5, 133.1, 130.7, 128.4, 127.51, 127.46, 126.8, 121.5, 113.5, 55.2, 49.5, 17.4 ppm.

HRMS (ESI-TOF) m/z: $[M+H]^+$ Calcd for $C_{23}H_{20}NO_3^+$ 358.1438; Found 358.1436.

***N*-(1-(2,6-dimethylphenoxy)propan-2-yl)-4'-methoxy-4-methyl-[1,1'-biphenyl]-2-carboxamide (99)**

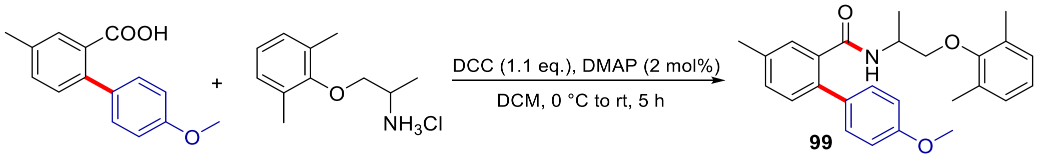

An oven-dried vessel was charged with 4'-methoxy-4-methyl-[1,1'-biphenyl]-2-carboxylic acid (48.4 mg, 0.2 mmol, 1 equiv.), DCC (45.9 mg, 0.22 mmol, 1.1equiv.), DMAP (0.5 mg, 0.004 mmol, 0.02 equiv.) and 1-(2,6-dimethylphenoxy)-2-aminopropanehydrochloride (44.5 mg, 0.2 mmol, 1 equiv.) were added at 0 °C. The reaction mixture was stirred at room temperature for 5 h .The resulting mixture was extracted with ethyl acetate (10 mL × 3), and the combined organic layers were washed with brine (10 mL), dried over anhydrous Na_2SO_4, filtered and concentrated. The residue was purified by flash chromatography on silica gel with petroleum ether/ethyl acetate to afford the desired product **99** as a white solid (56.4 mg, 70%).

M.P.: 96 – 99 °C.

^{1}H NMR (400 MHz, $CDCl_3$): δ 7.52 (s, 1H), 7.35 – 7.31 (m, 2H), 7.248 – 7.22 (m, 2H), 6.98 – 6.93 (m, 2H), 6.92 – 6.86 (m, 3H), 5.68 – 5.60 (m, 1H), 4.32 – 4.24 (m, 1H), 3.74 (s, 3H), 3.59 – 3.55 (m, 2H), 2.41 (s, 3H), 2.14 (s, 6H), 1.13 (d, J = 6.8 Hz, 3H) ppm.

^{13}C NMR (101 MHz, $CDCl_3$): δ 169.2, 159.2, 154.9, 137.1, 136.3, 135.4, 132.5, 130.8, 130.6, 130.1, 129.9, 129.2, 128.8, 123.9, 114.0, 73.6, 55.2, 45.7, 20.9, 17.1, 16.2 ppm.

HRMS (ESI-TOF) m/z: $[M+H]^+$ Calcd for $C_{26}H_{30}NO_3^+$ 404.2220; Found 404.2220.

Further subsequent couplings with benzoic acids

Dimethyl 3-methoxy-3''-methyl-[1,1':4',1''-terphenyl]-2,2''-dicarboxylate (100)

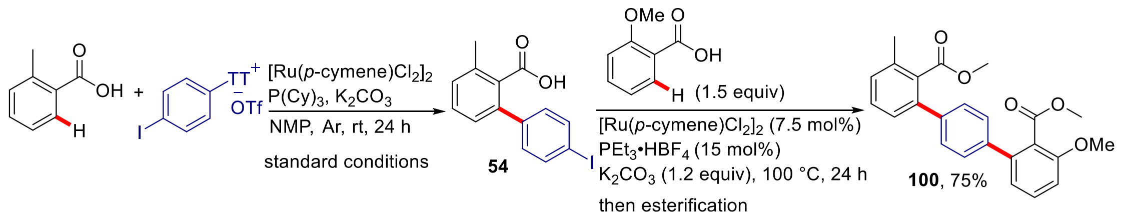

One-pot procedure: An oven-dried vessel was charged with 2-methylbenzoic acid (28.2 mg, 0.20 mmol, 1 equiv.), and 5-(4-iodophenyl)-5*H*-thianthren-5-ium trifluoromethanesulfonate (136.3 mg, 0.24 mmol, 1.2 equiv.), [Ru(*p*-cymene)Cl_2]$_2$ (3.2 mg, 5.00 μmol, 0.025 equiv.), K_2CO_3 (33.6 mg, 0.24 mmol, 1.2 equiv.), P(Cy)$_3$ (5.6 mg, 0.02 mmol, 0.1 equiv.). Under exclusion of air, NMP (4 mL) was added via syringe. The resulting reaction mixture was stirred at rt for 24 h. And then 2-methoxybenzoic acid (45.8 mg, 0.30 mmol, 1.5 equiv.), [Ru(*p*-cymene)Cl_2]$_2$ (9.6 mg, 15.00 μmol, 0.075 equiv.), K_2CO_3 (33.6 mg, 0.24 mmol, 1.2 equiv.), PEt_3•HBF_4 (6.2 mg, 0.03 mmol, 0.15 equiv.) were added under exclusion of air. The resulting reaction mixture was further stirred at 100 ^{o}C for 24 h. MeCN (2 mL), K_2CO_3 (138 mg, 1.0 mmol, 5 equiv.) and CH_3I (283.6 mg, 2.0 mmol, 10 equiv.) were added and the mixtures were stirred at 60 °C for 2.5 h. Brine (10 mL) was added and the resulting mixture was extracted with ethyl acetate (3×20 mL). The combined organic layers were dried over $MgSO_4$, filtered, and the volatiles were removed under reduced pressure. The residue was purified by column chromatography (SiO_2, ethyl acetate/cyclohexane gradient), affording the corresponding product **100** as oil liquid (58.5 mg, 75%).

^{1}H NMR (400 MHz, $CDCl_3$): δ 7.45 – 7.35 (m, 6H), 7.27 – 7.21 (m, 2H), 7.03 (d, *J* = 7.7 Hz, 1H), 6.96 (d, J = 8.4 Hz, 1H), 3.89 (s, 3H), 3.67 (s, 3H), 3.61 (s, 3H), 2.41 (s, 3H) ppm.

^{13}C NMR (101 MHz, $CDCl_3$): δ 170.2, 168.4, 156.5, 140.7, 140.2, 139.6, 139.1, 135.6, 133.1, 130.6, 129.5, 129.2, 128.2, 128.2, 127.1, 123.0, 121.9, 110.0, 56.1, 52.1, 51.8, 19.7 ppm.

HRMS (ESI-TOF) m/z: $[M+H]^+$ Calcd for $C_{24}H_{23}O_5^+$ 391.1540; Found 391.1528.

dimethyl 5-chloro-4-methoxy-4''-methyl-[1,1':4',1''-terphenyl]-2,2''-dicarboxylate (101)

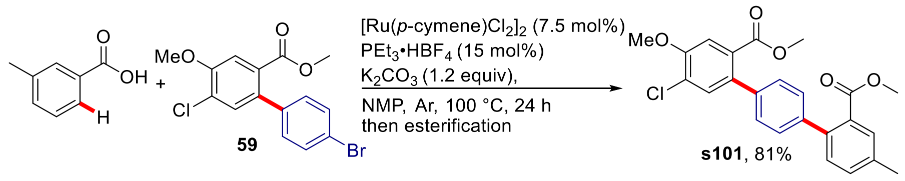

An oven-dried vessel was charged with 3-methylbenzoic acid (27.3 mg, 0.20 mmol, 1 equiv.), **59** (159.8 mg, 0.30 mmol, 1.5 equiv.), [Ru(*p*-cymene)Cl_2]$_2$ (9.6 mg, 15.0 μmol, 0.075 equiv.), K_2CO_3 (33.6 mg, 0.24 mmol, 1.2 equiv.), PEt_3•HBF_4 (6.2 mg, 0.03 mmol, 0.15 equiv.), NMP (1 mL) were added under exclusion of air. The resulting reaction mixture was stirred at 100 ºC for 24 h. MeCN (2 mL), K_2CO_3 (83.0 mg, 0.60 mmol, 3 equiv.) and CH_3I (141.9 mg, 1.0 mmol, 5 equiv.) were added and the mixtures were stirred at 60 °C for 2.5 h. Brine (10 mL) was added and the resulting mixture was extracted with ethyl acetate (3×20 mL). The combined organic layers were dried over $MgSO_4$, filtered, and the volatiles were removed under reduced pressure. The residue was purified by column chromatography (SiO_2, ethyl acetate/cyclohexane gradient), affording the corresponding product **101** as yellow oil liquid (68.8 mg, 81%)

^{1}H NMR (400 MHz, $CDCl_3$): 7.64 (s, 1H), 7.40 (s, 1H), 7.37 – 7.24 (m, 6H), 3.98 (s, 3H), 3.66 (s, 6H), 2.42 (s, 3H) ppm.

^{13}C NMR (101 MHz, $CDCl_3$): δ 169.3, 168.2, 154.0, 140.3, 139.1, 138.6, 137.2, 135.6, 132.3, 132.0, 130.7, 130.6, 130.3, 129.8, 128.1, 128.0, 125.6, 113.1, 56.4, 52.1, 51.9, 20.9 ppm.

HRMS (ESI-TOF) m/z: $[M+H]^+$ Calcd for $C_{24}H_{22}ClO_5^+$ 425.1150; Found 425.1138.

Copies of ^{1}H and ^{13}C NMR Spectra

^{1}H NMR spectra (400 MHz) of **3** in $CDCl_3$.

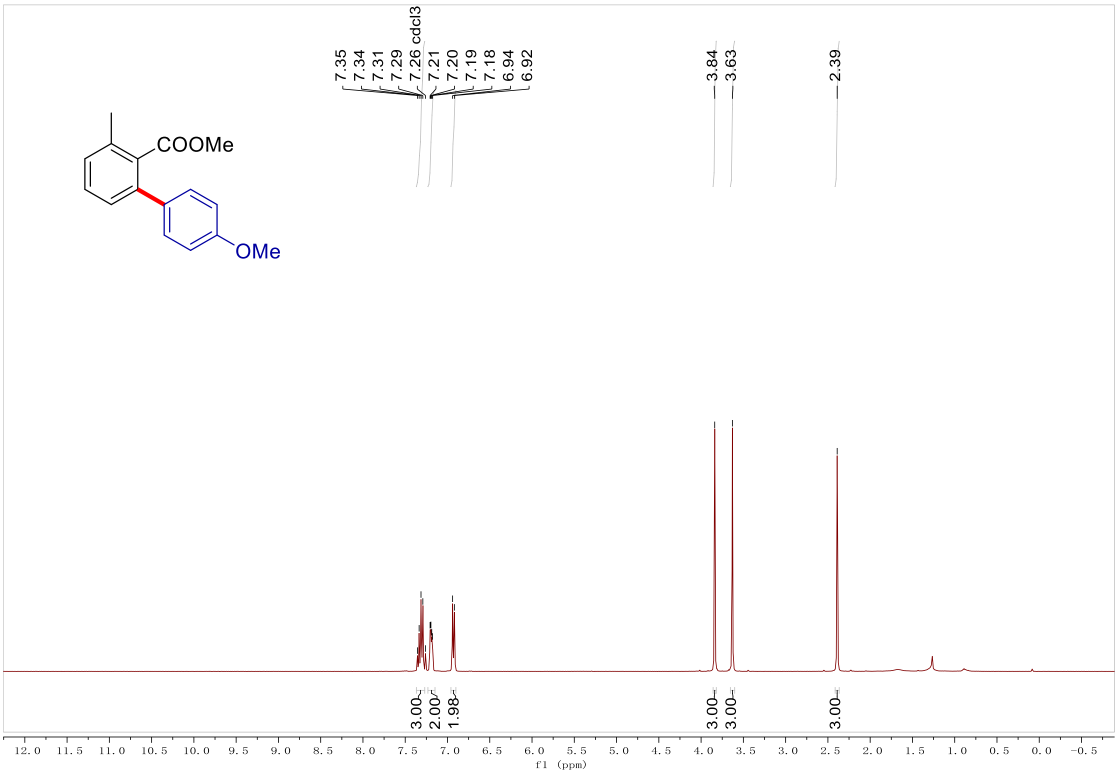

^{13}C NMR spectra (101 MHz) of **3** in $CDCl_3$.

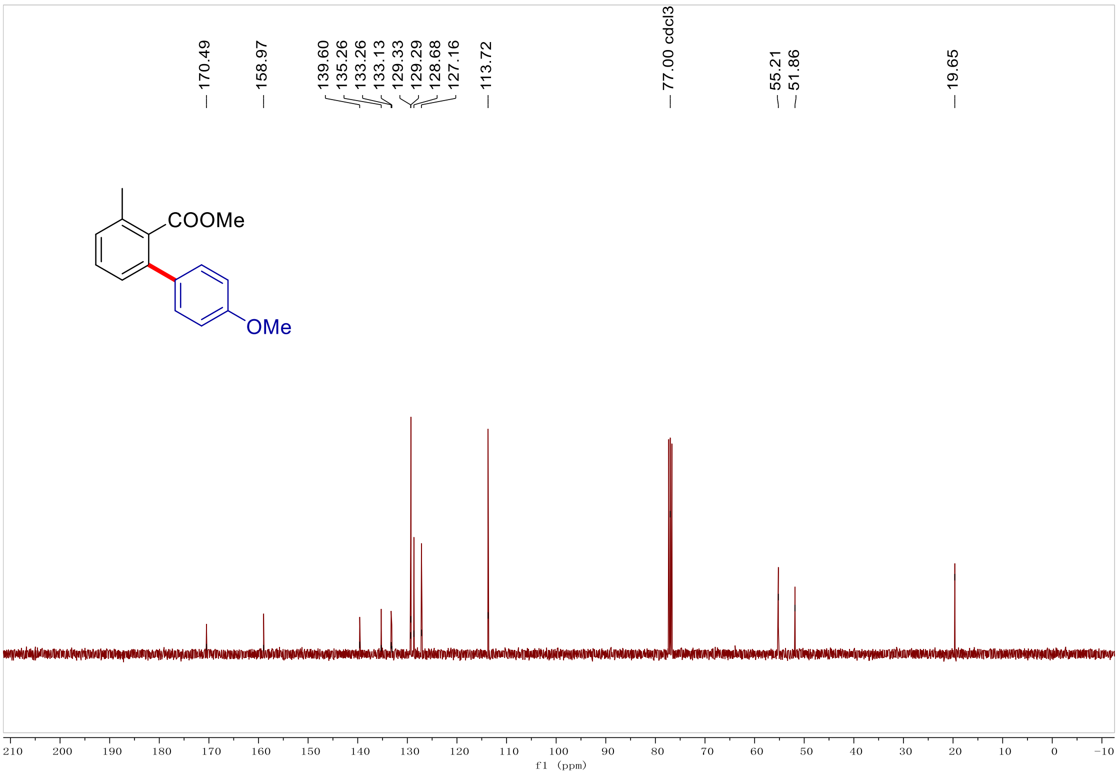

^{1}H NMR spectra (400 MHz) of **4** in $CDCl_3$.

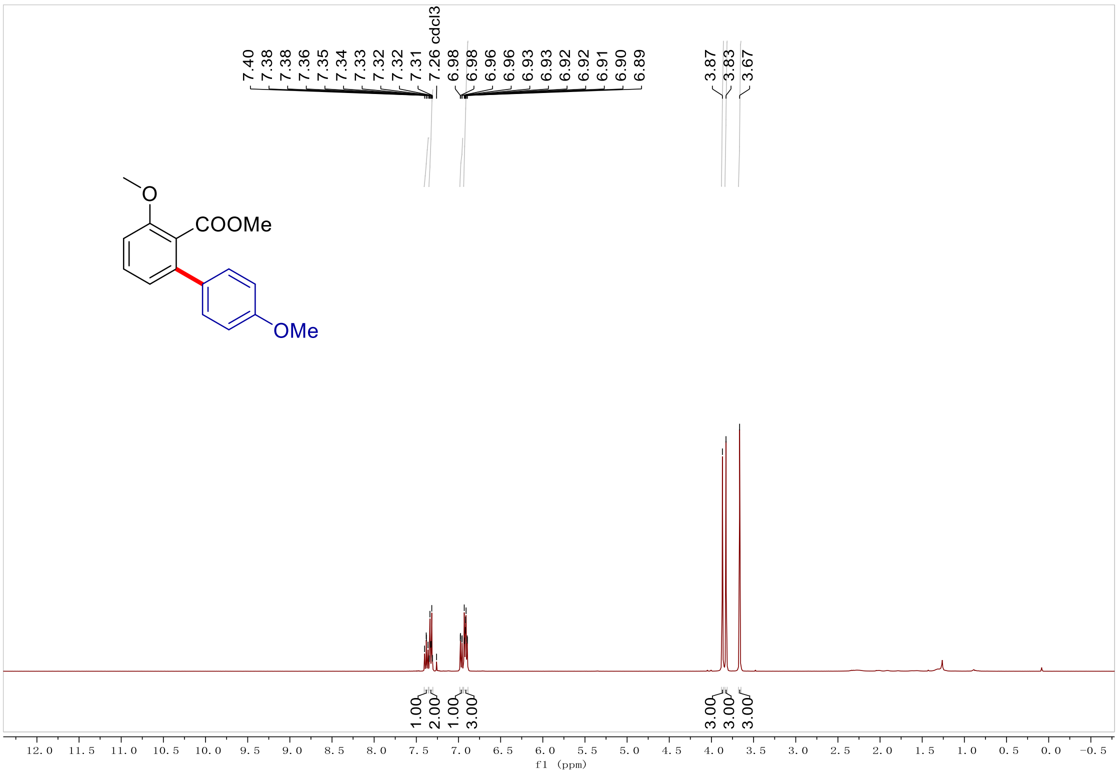

^{13}C NMR spectra (101 MHz) of **4** in $CDCl_3$.

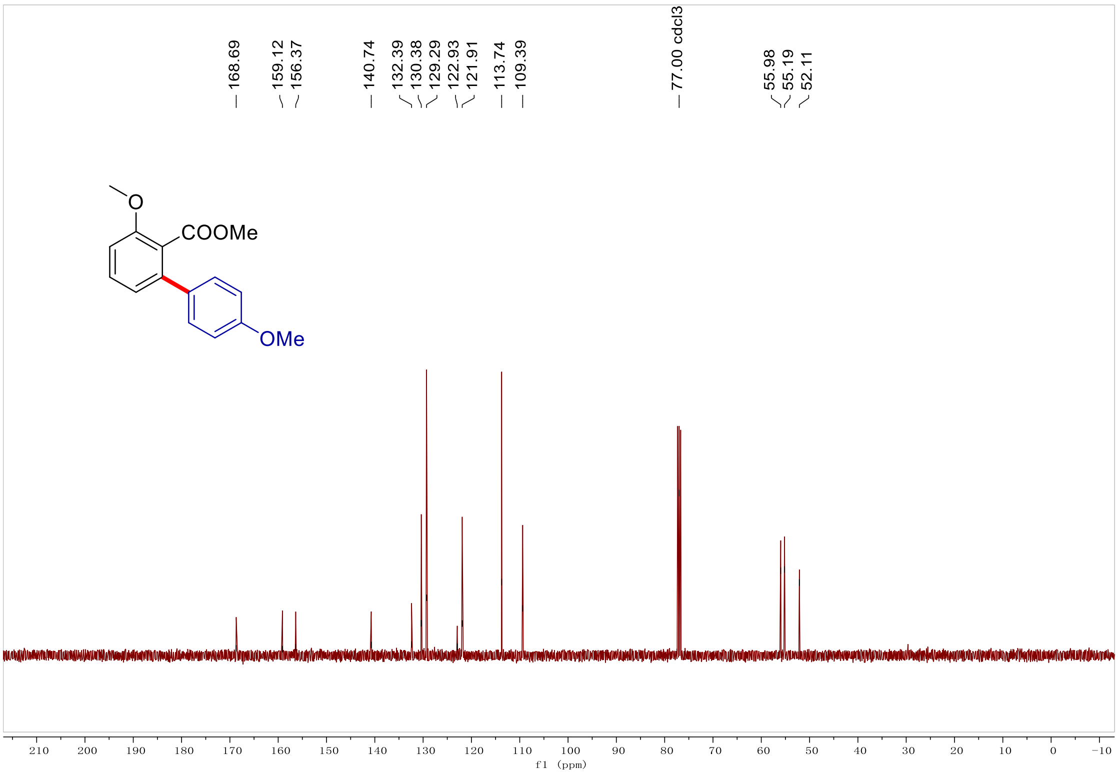

^{1}H NMR spectra (400 MHz) of **5** in $CDCl_3$.

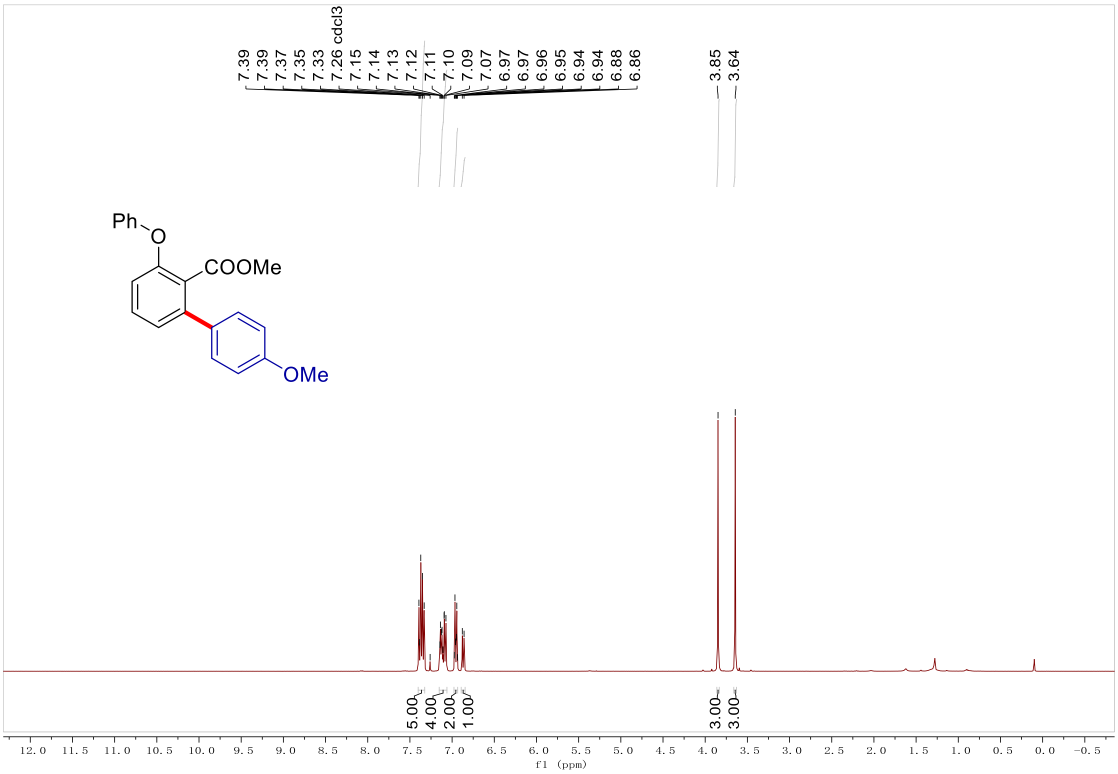

^{13}C NMR spectra (101 MHz) of **5** in $CDCl_3$.

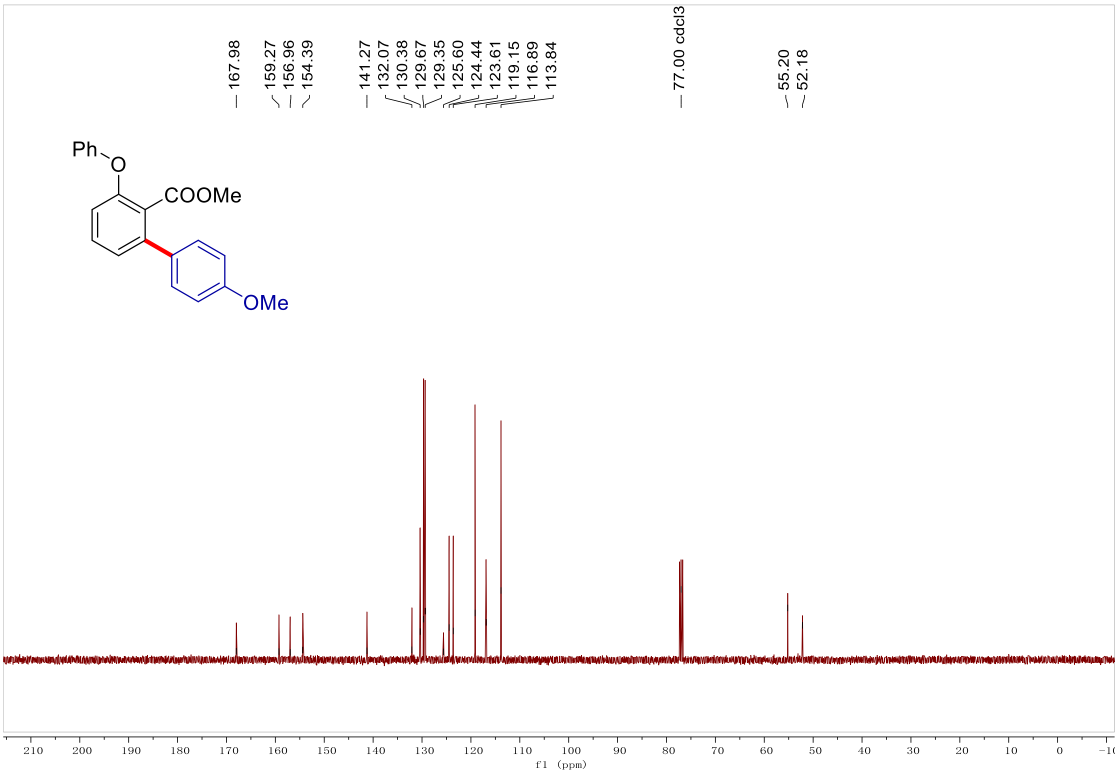

^{1}H NMR spectra (400 MHz) of **6** in $CDCl_3$.

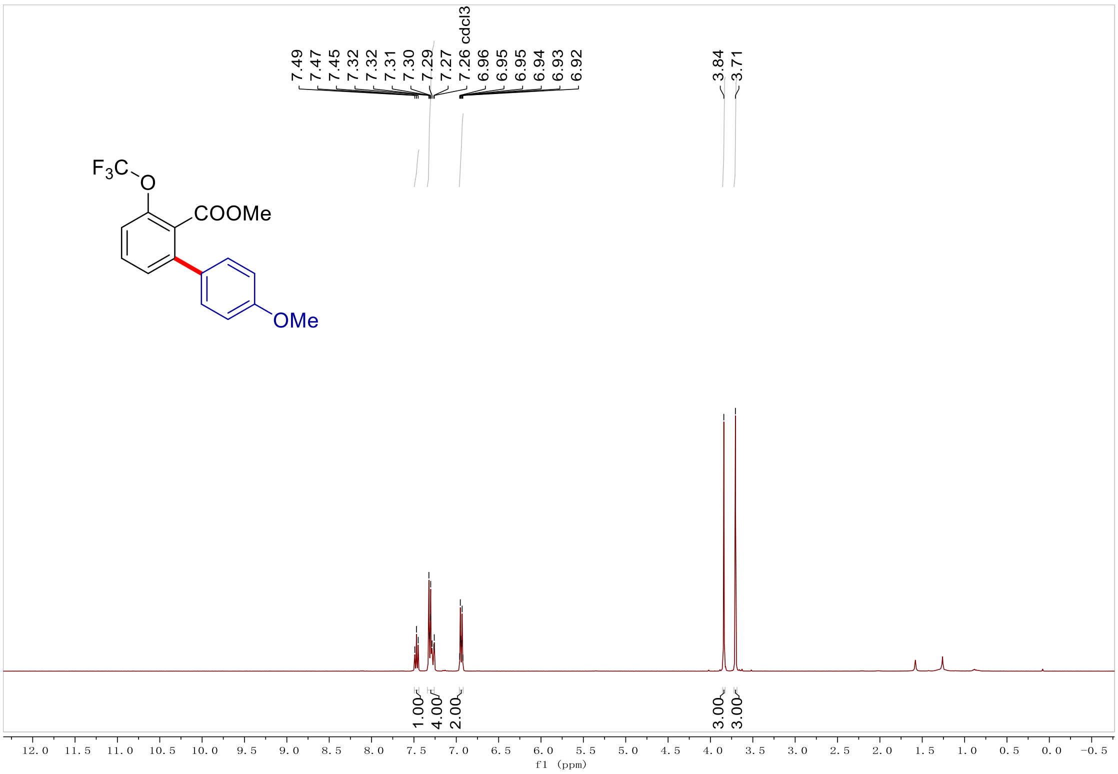

^{13}C NMR spectra (101 MHz) of **6** in $CDCl_3$.

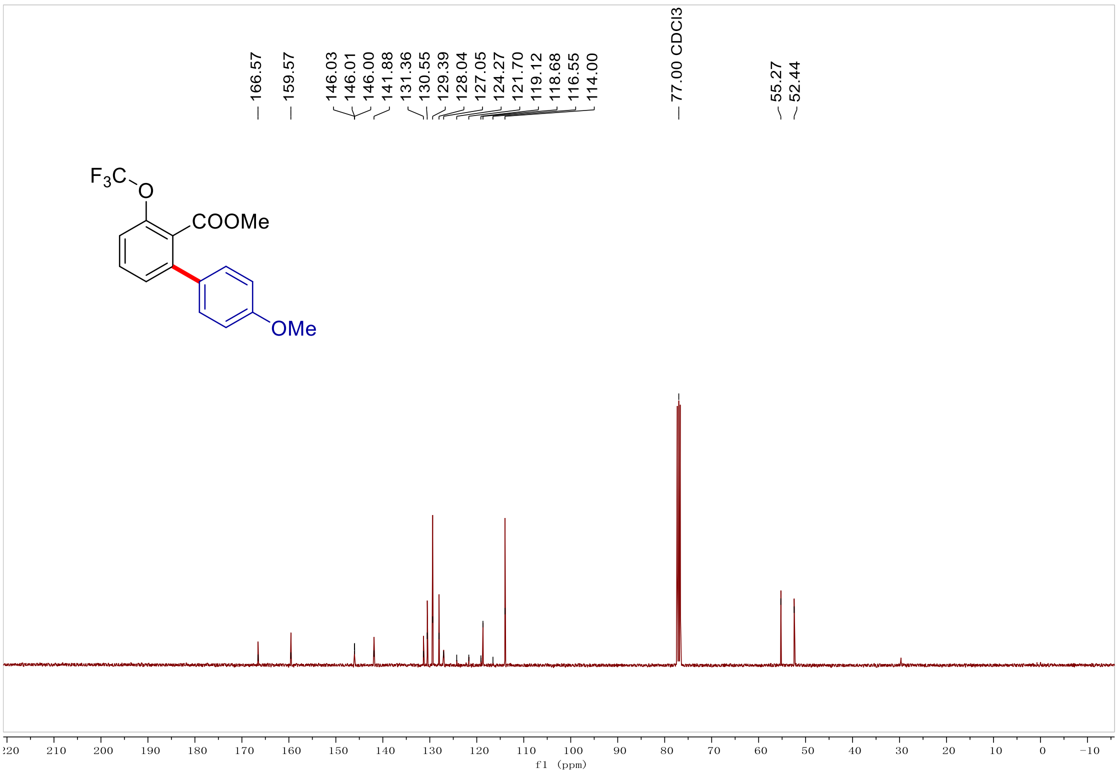

^{1}H NMR spectra (400 MHz) of **7** in $CDCl_3$.

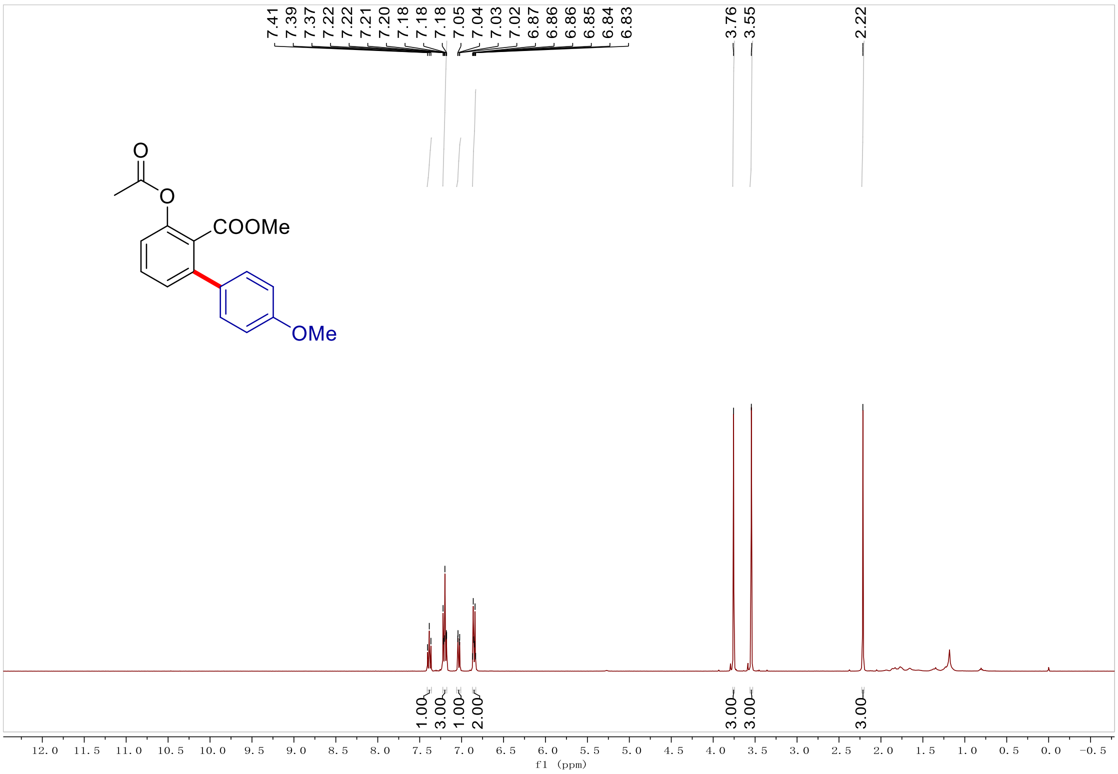

^{13}C NMR spectra (101 MHz) of **7** in $CDCl_3$.

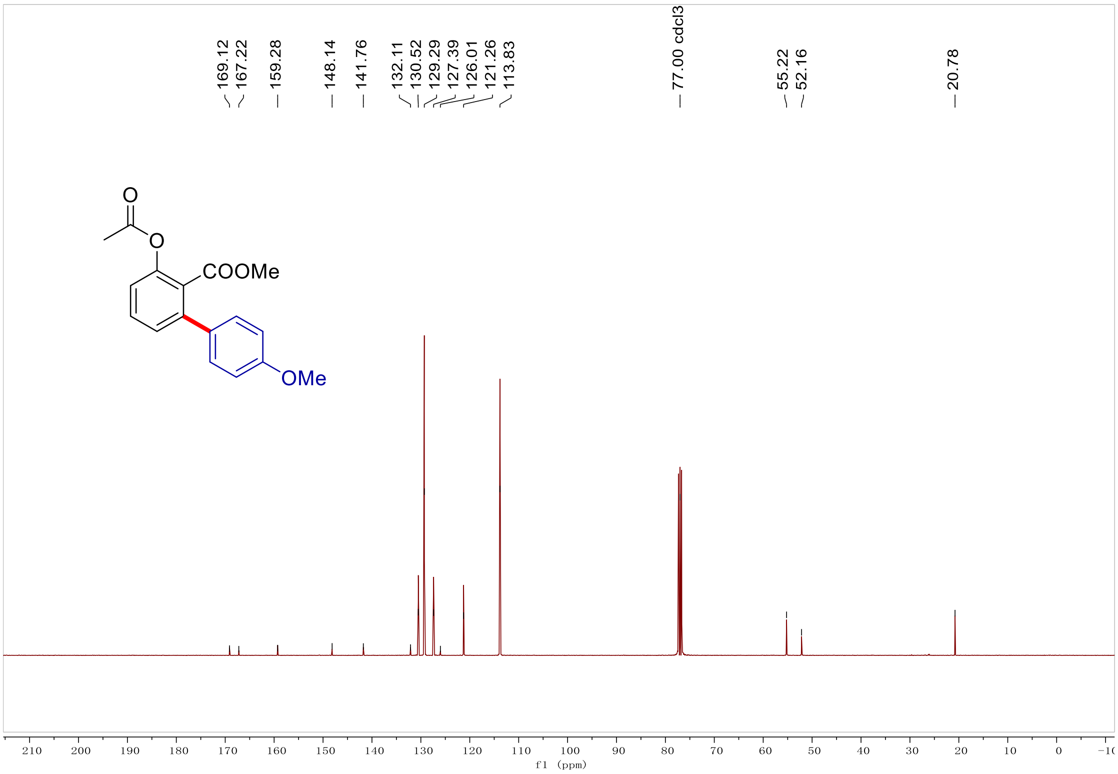

^{1}H NMR spectra (400 MHz) of **8** in $CDCl_3$.

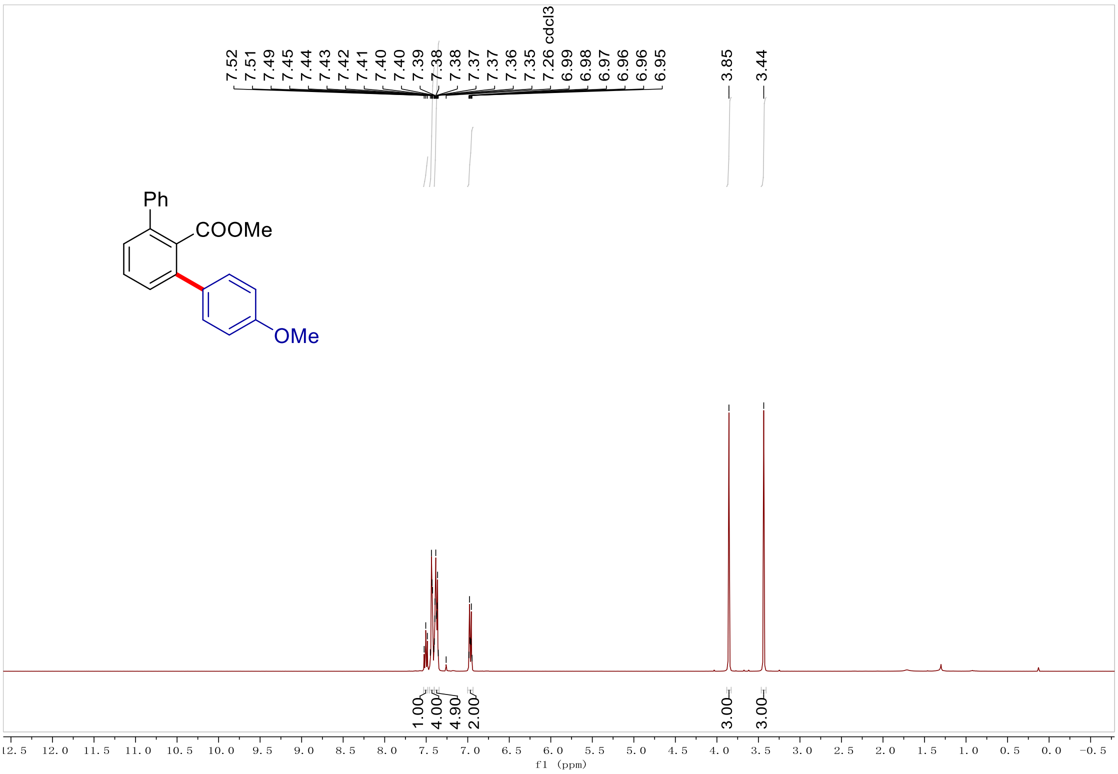

^{13}C NMR spectra (101 MHz) of **8** in $CDCl_3$.

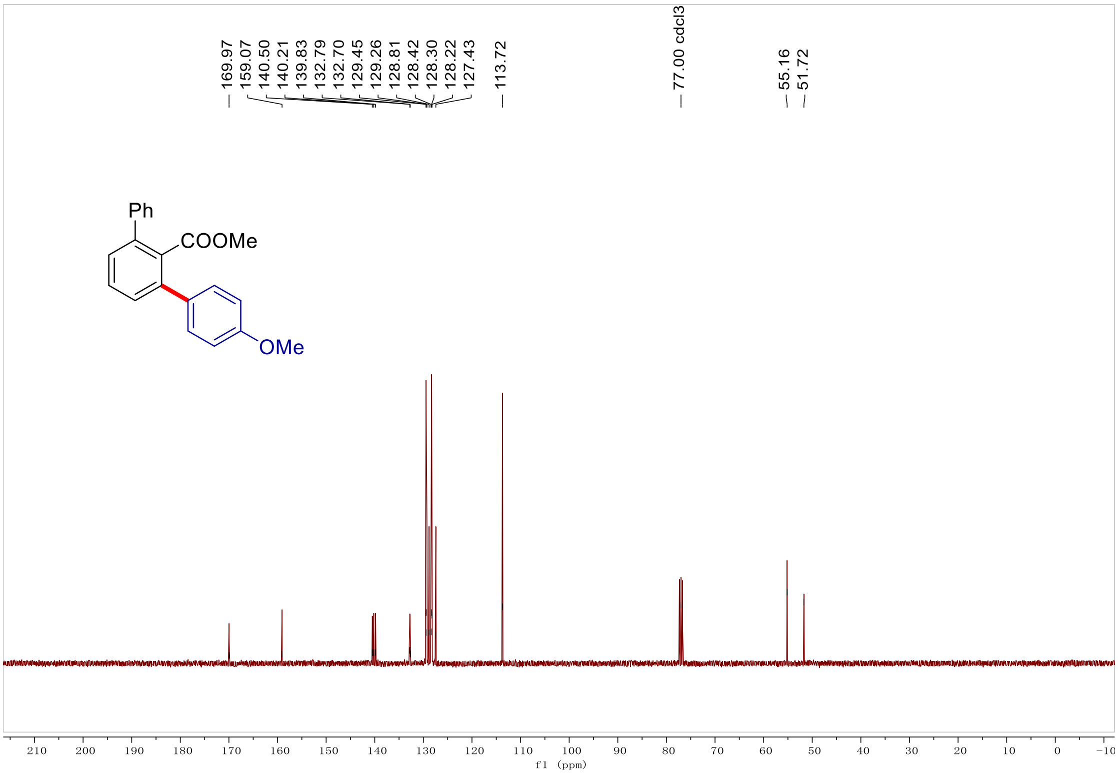

^{1}H NMR spectra (400 MHz) of **9** in $CDCl_3$.

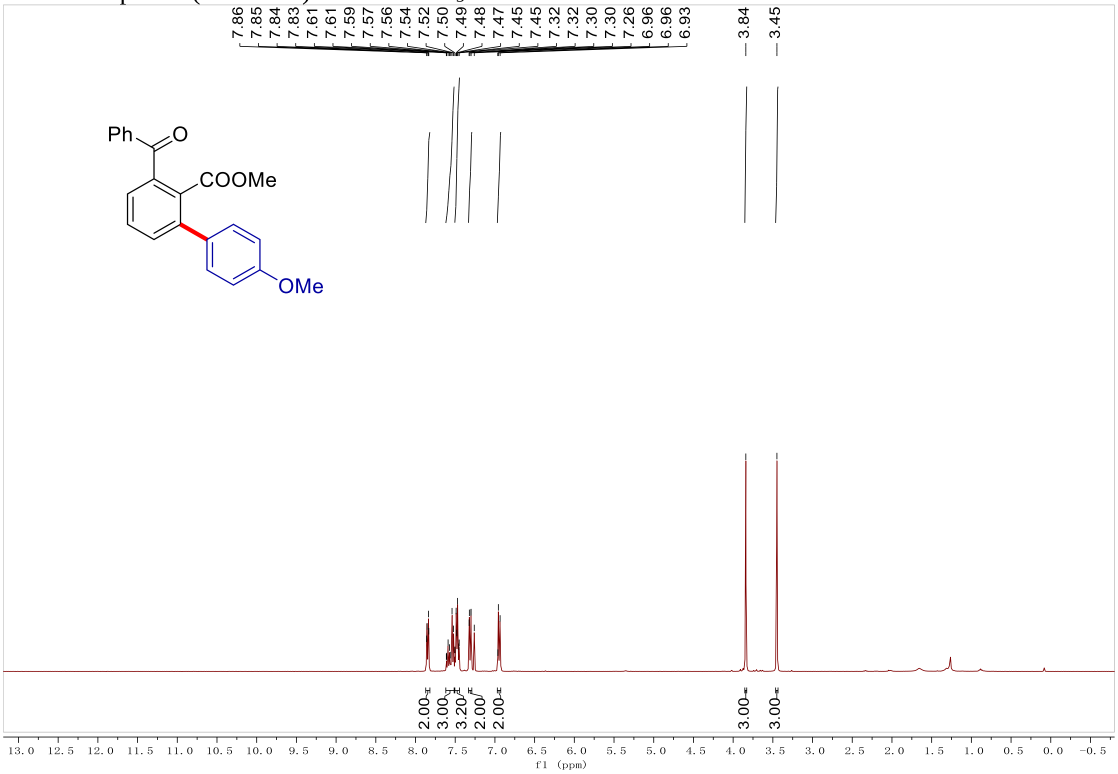

^{13}C NMR spectra (101 MHz) of **9** in $CDCl_3$.

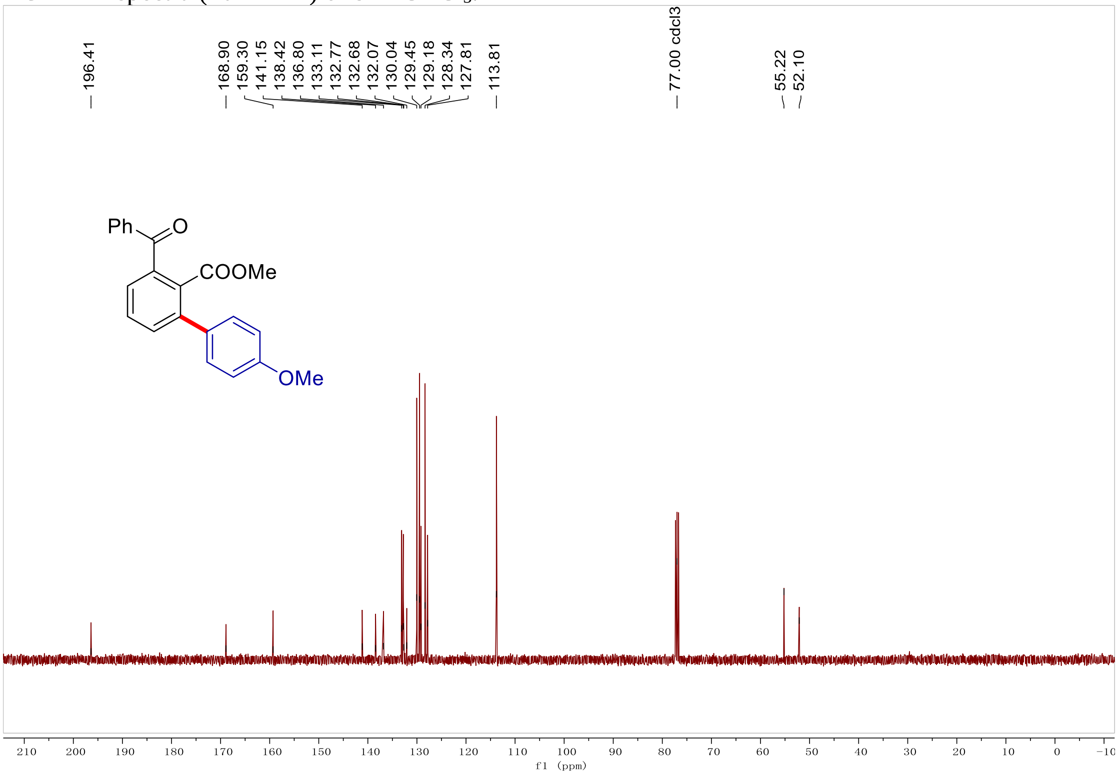

^{1}H NMR spectra (400 MHz) of **10** in $CDCl_3$.

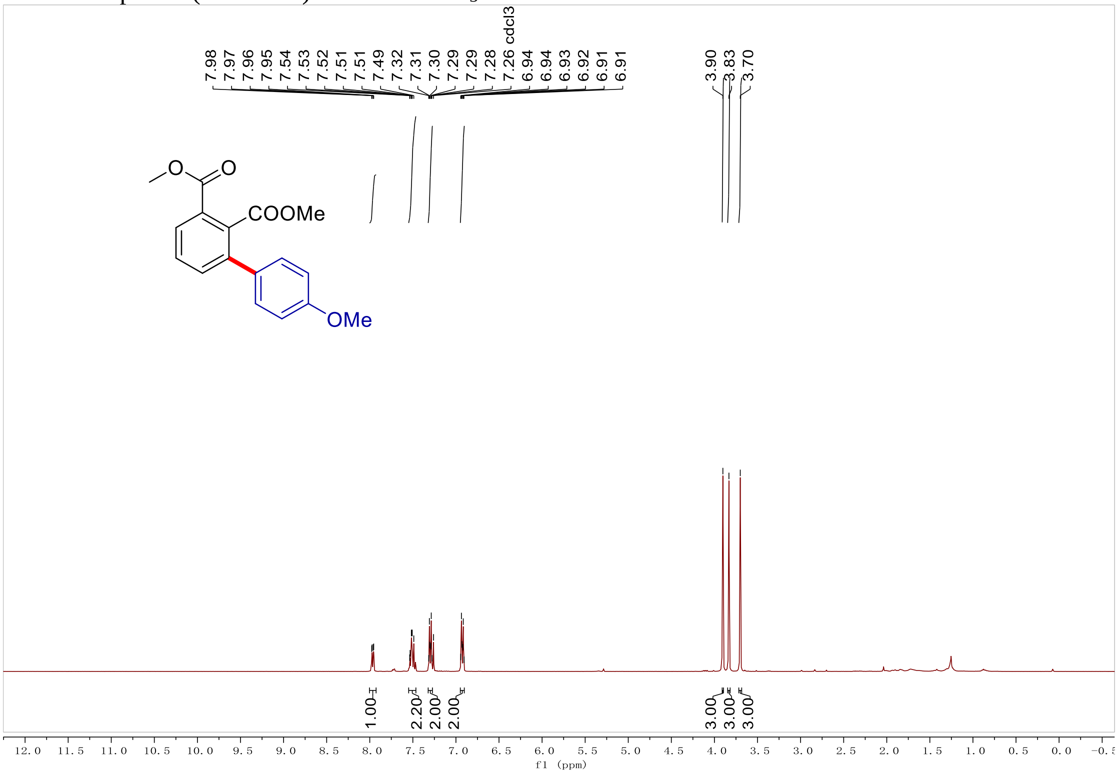

^{13}C NMR spectra (101 MHz) of **10** in $CDCl_3$.

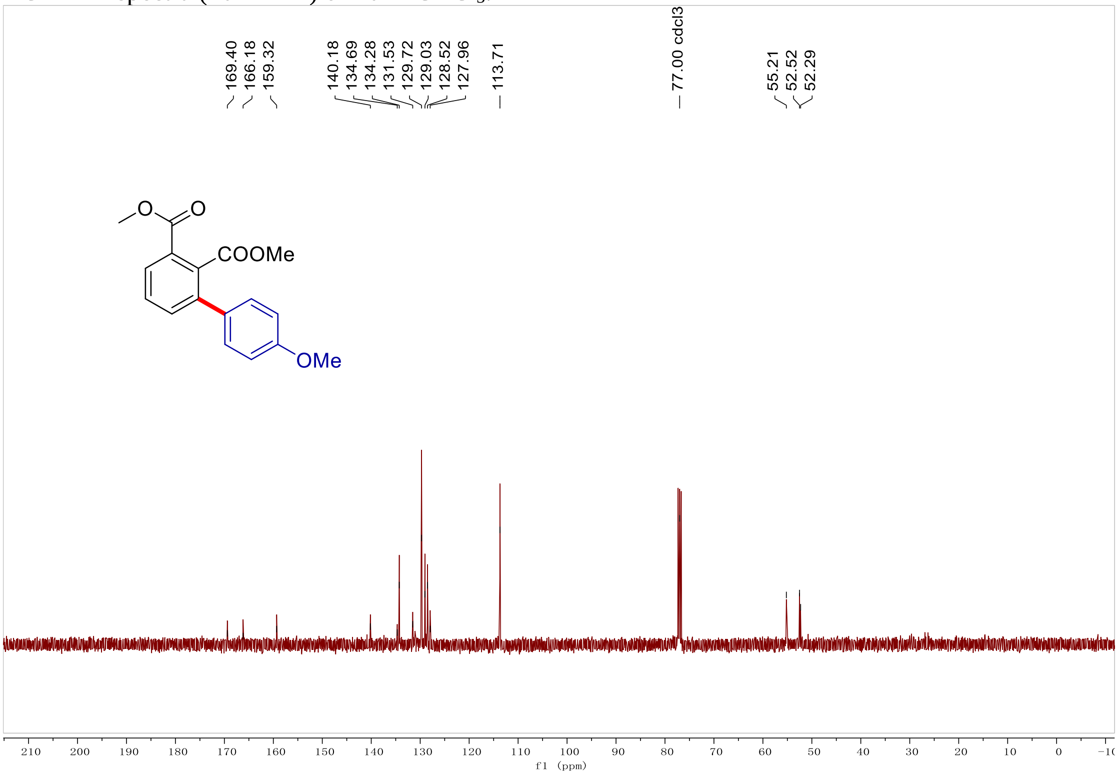

[1]**H NMR** spectra (400 MHz) of **11** in $CDCl_3$.

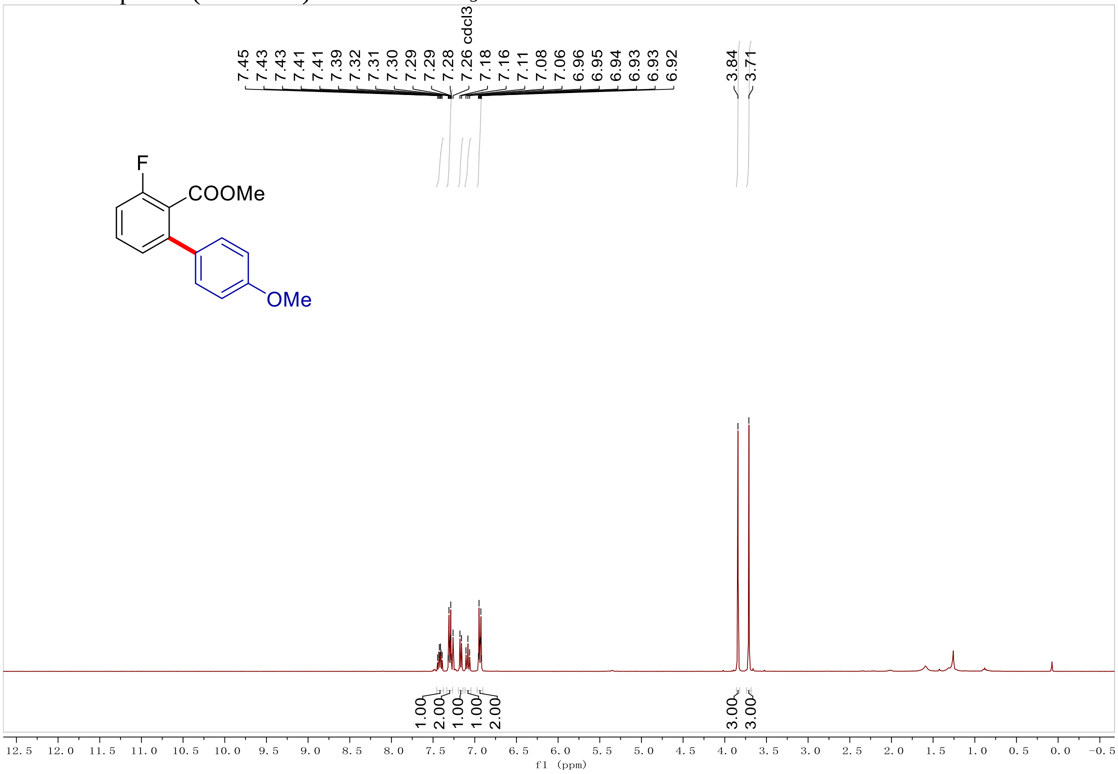

[13]**C NMR** spectra (101 MHz) of **11** in $CDCl_3$.

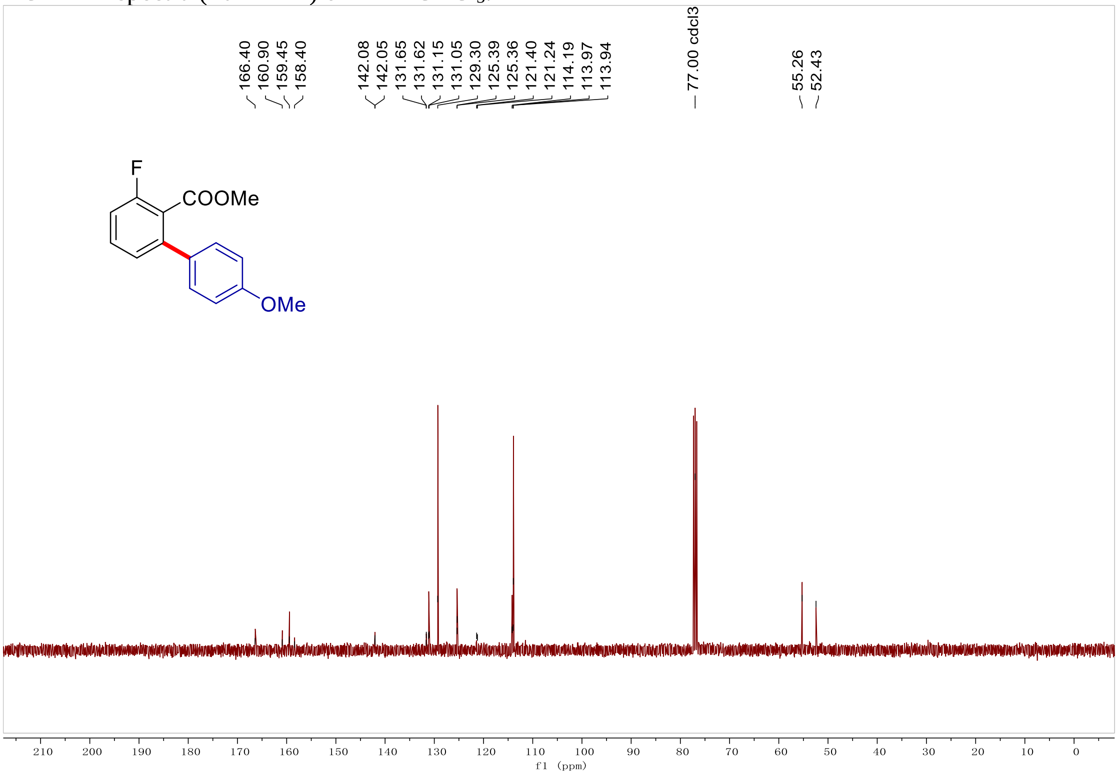

^{1}H NMR spectra (400 MHz) of **12** in $CDCl_3$.

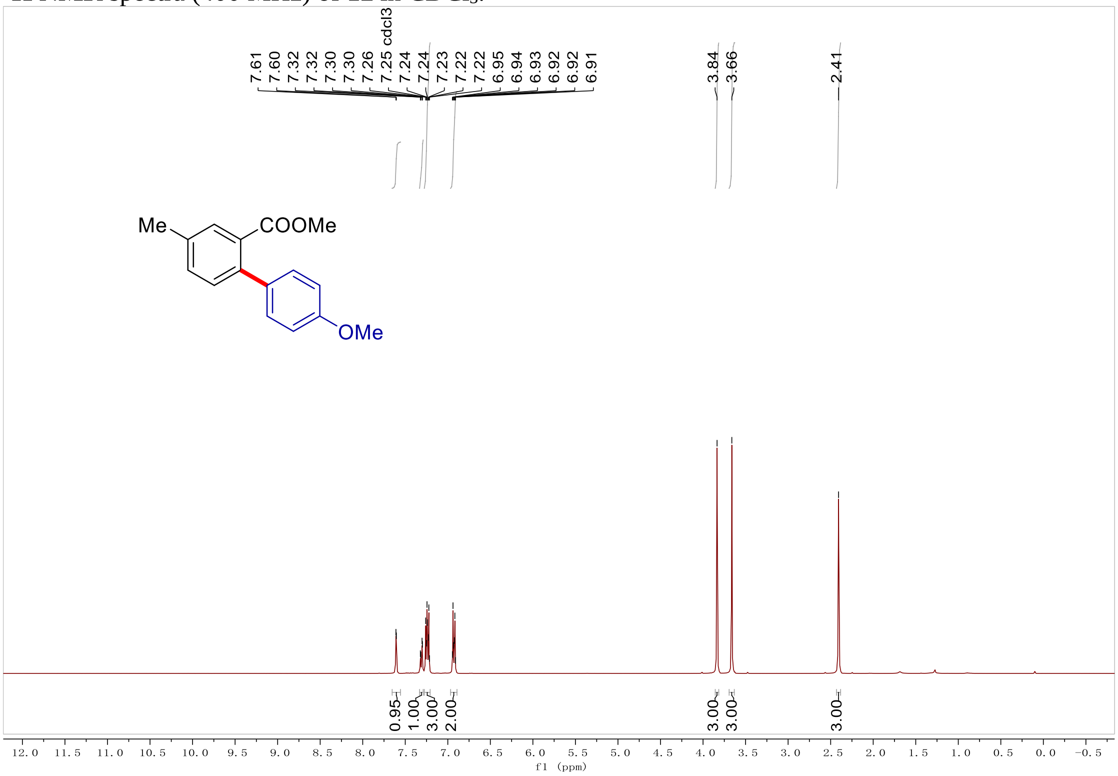

^{13}C NMR spectra (101 MHz) of **12** in $CDCl_3$.

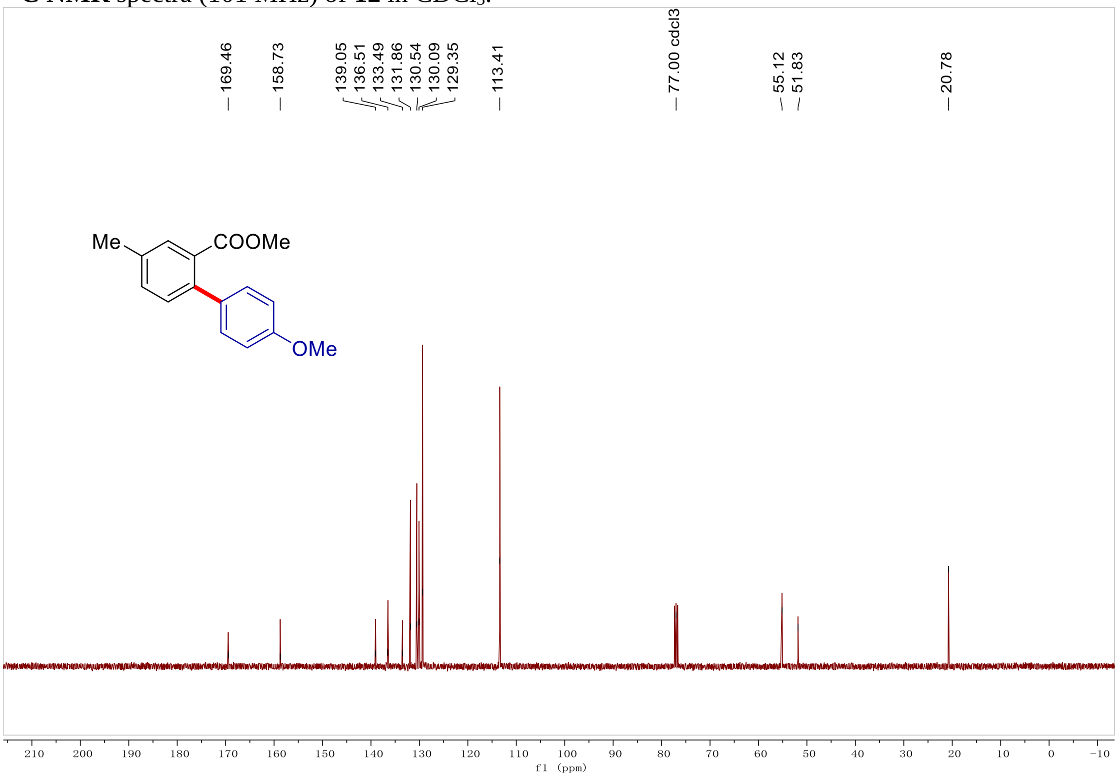

[1]**H NMR** spectra (400 MHz) of **13** in $CDCl_3$.

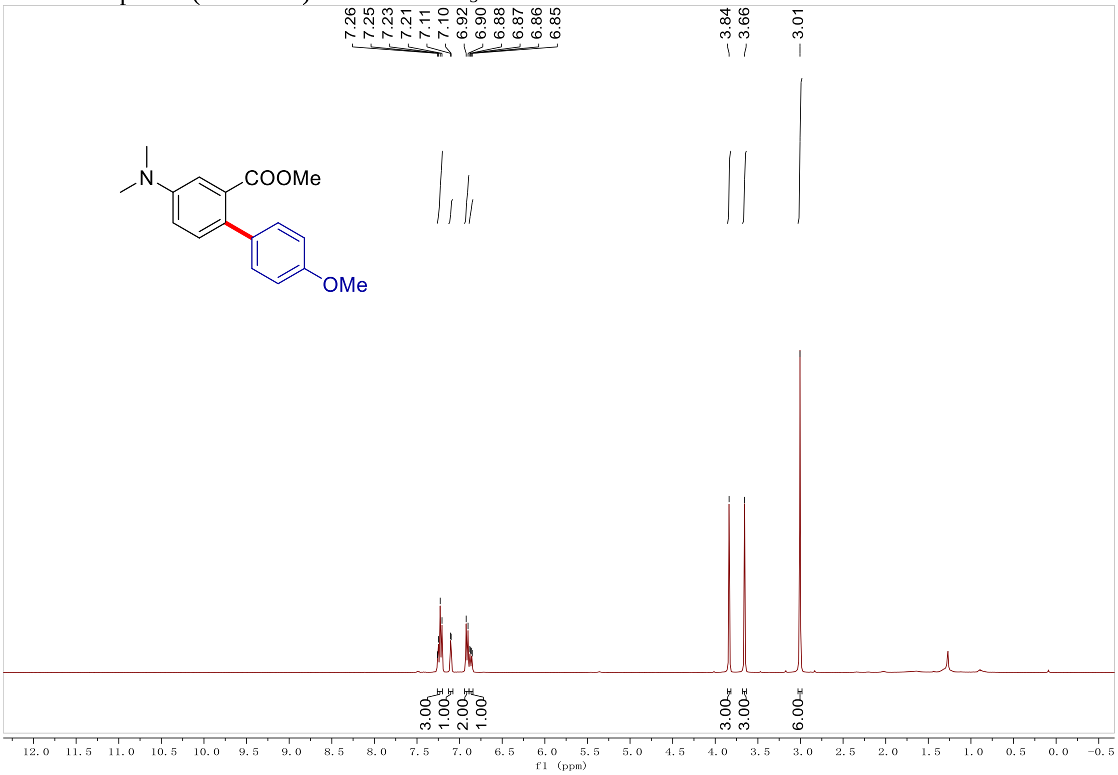

[13]**C NMR** spectra (101 MHz) of **13** in $CDCl_3$.

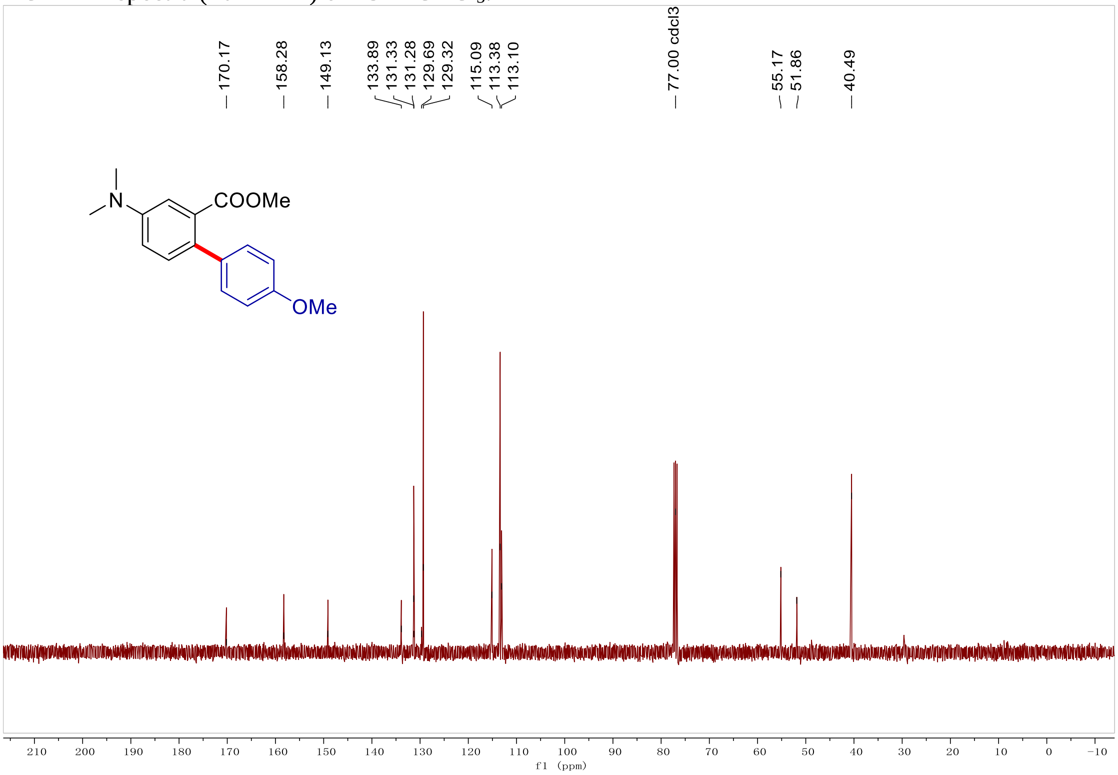

^{1}H NMR spectra (400 MHz) of **14** in $CDCl_3$.

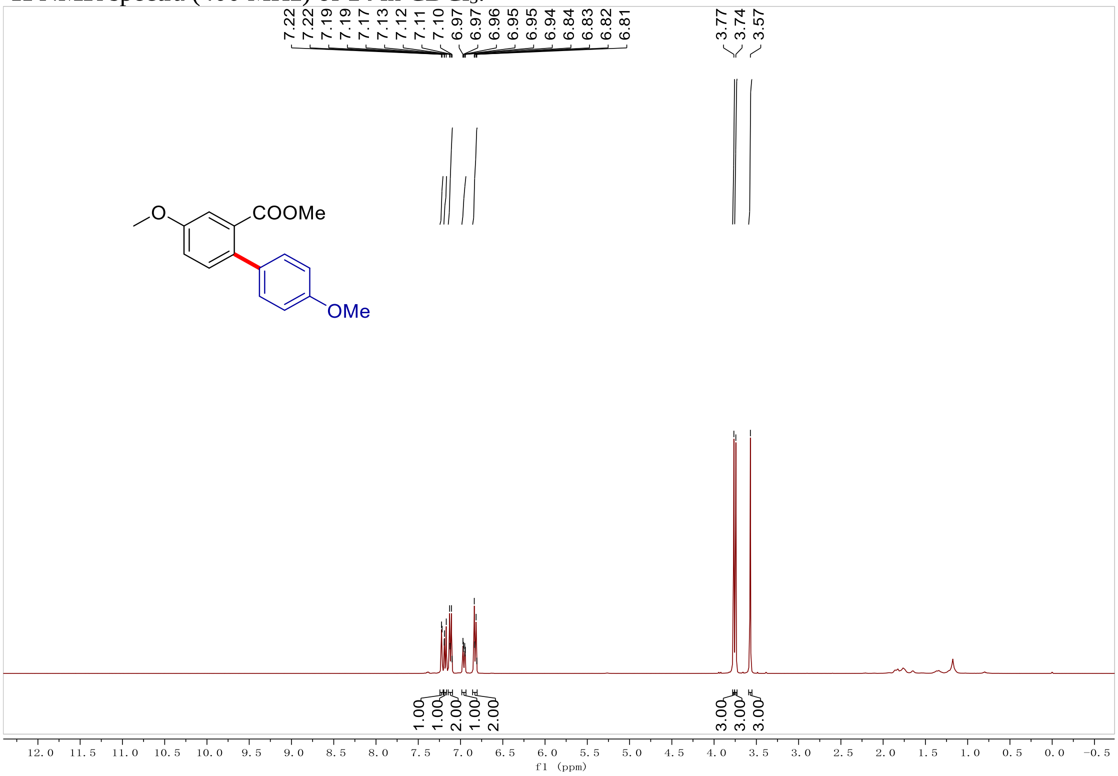

^{13}C NMR spectra (101 MHz) of **14** in $CDCl_3$.

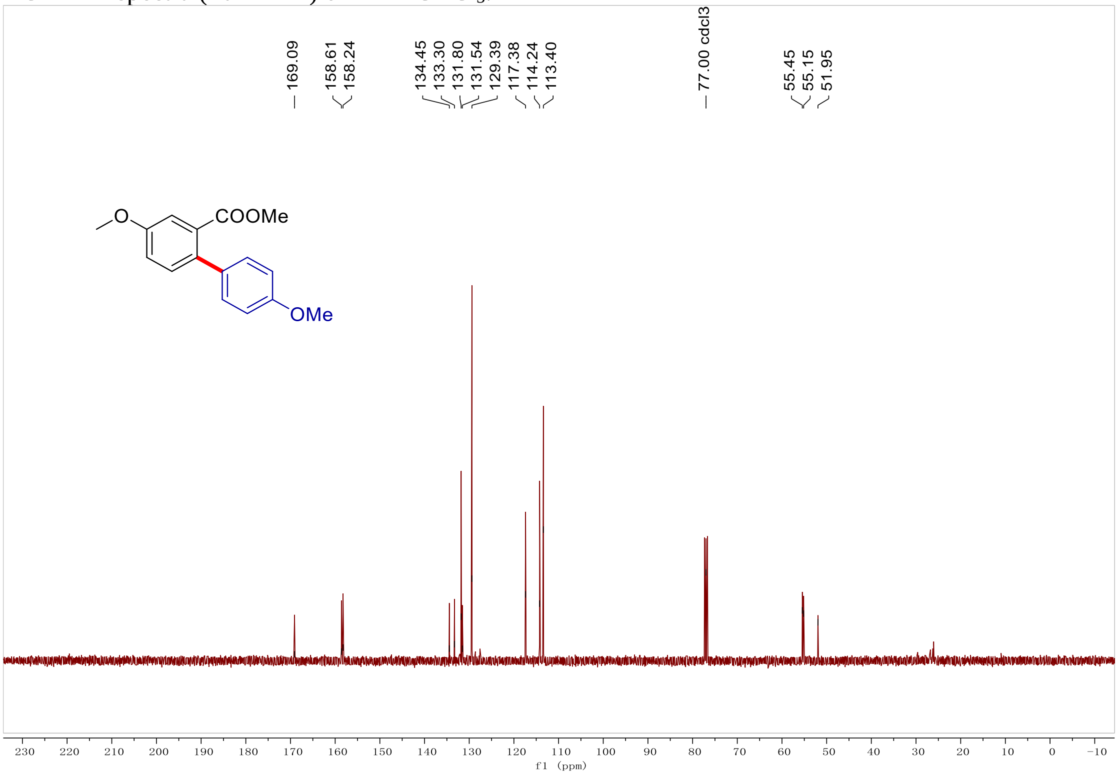

^{1}H NMR spectra (400 MHz) of **15** in $CDCl_3$.

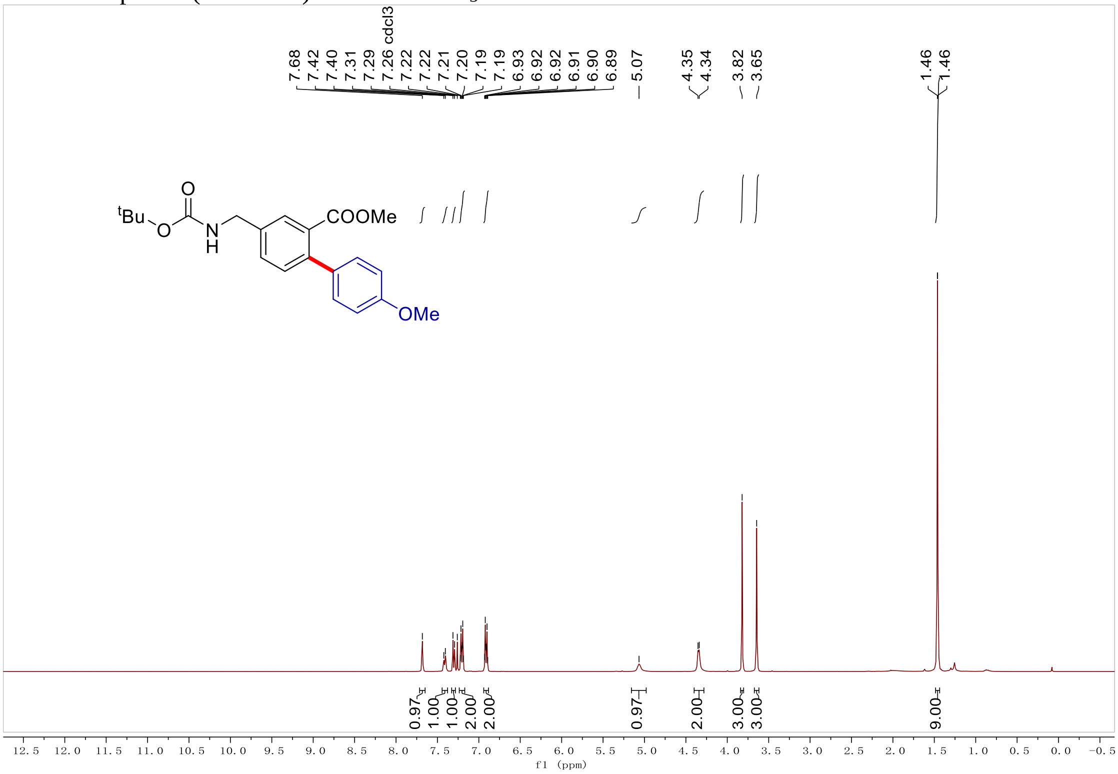

^{13}C NMR spectra (101 MHz) of **15** in $CDCl_3$.

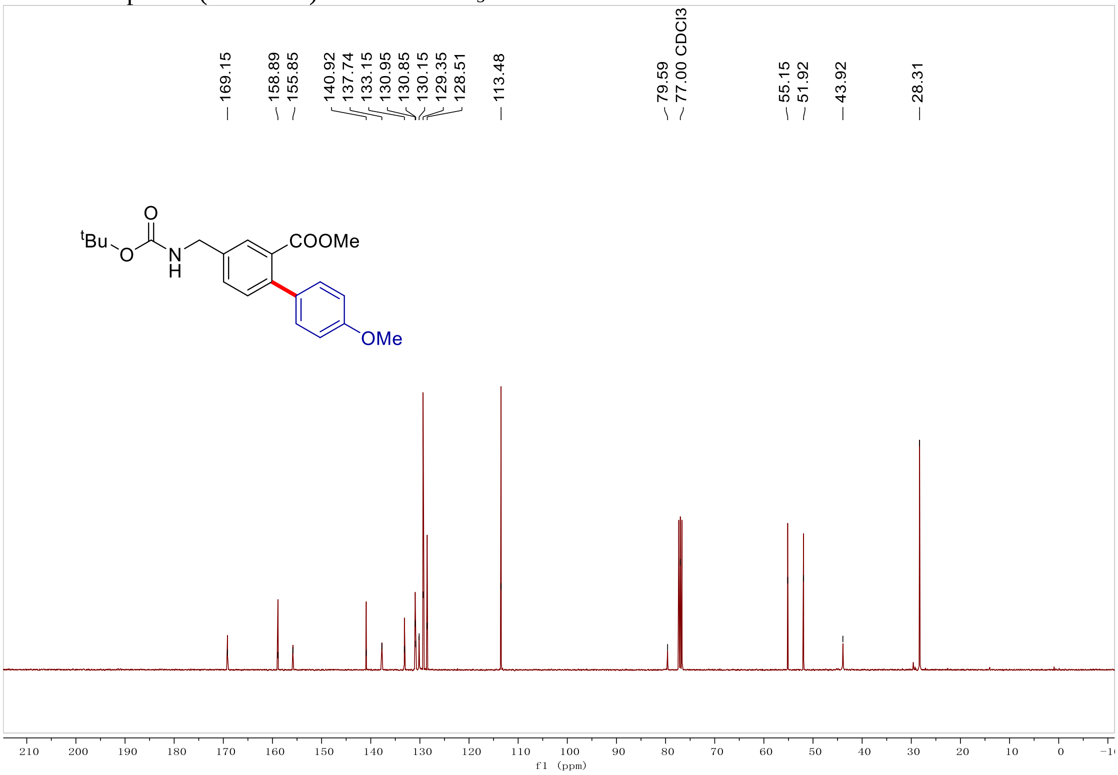

[1]**H NMR** spectra (400 MHz) of **16** in $CDCl_3$.

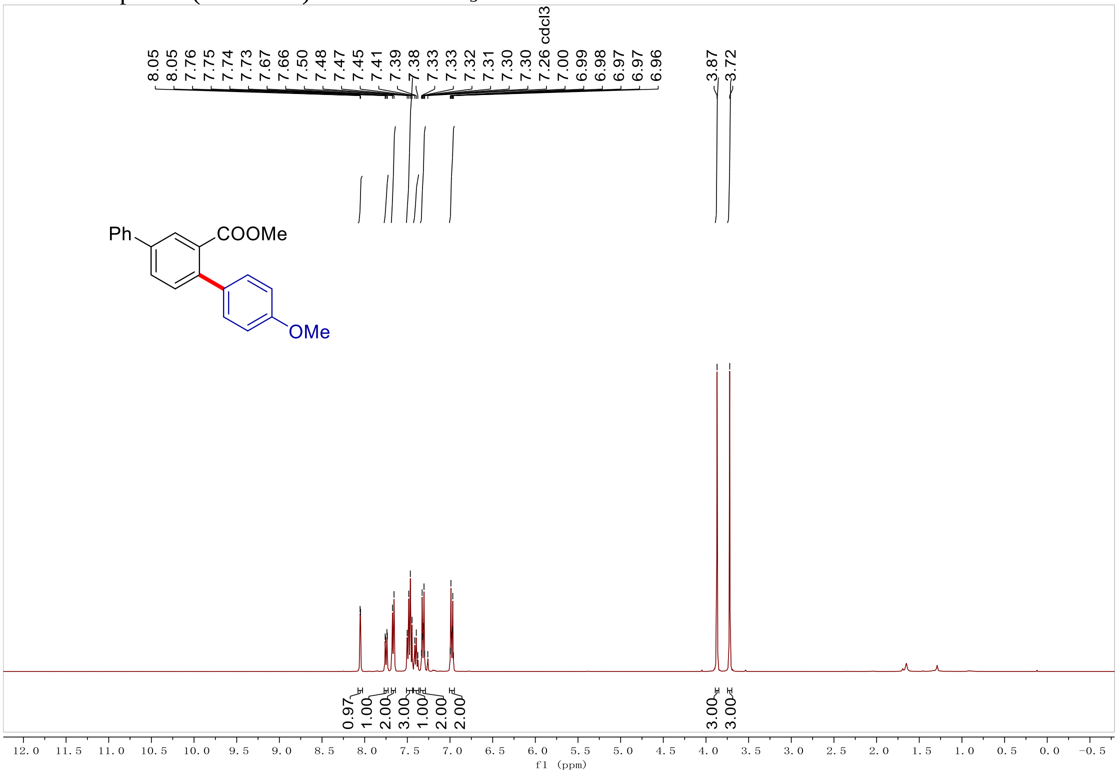

[13]**C NMR** spectra (101 MHz) of **16** in $CDCl_3$.

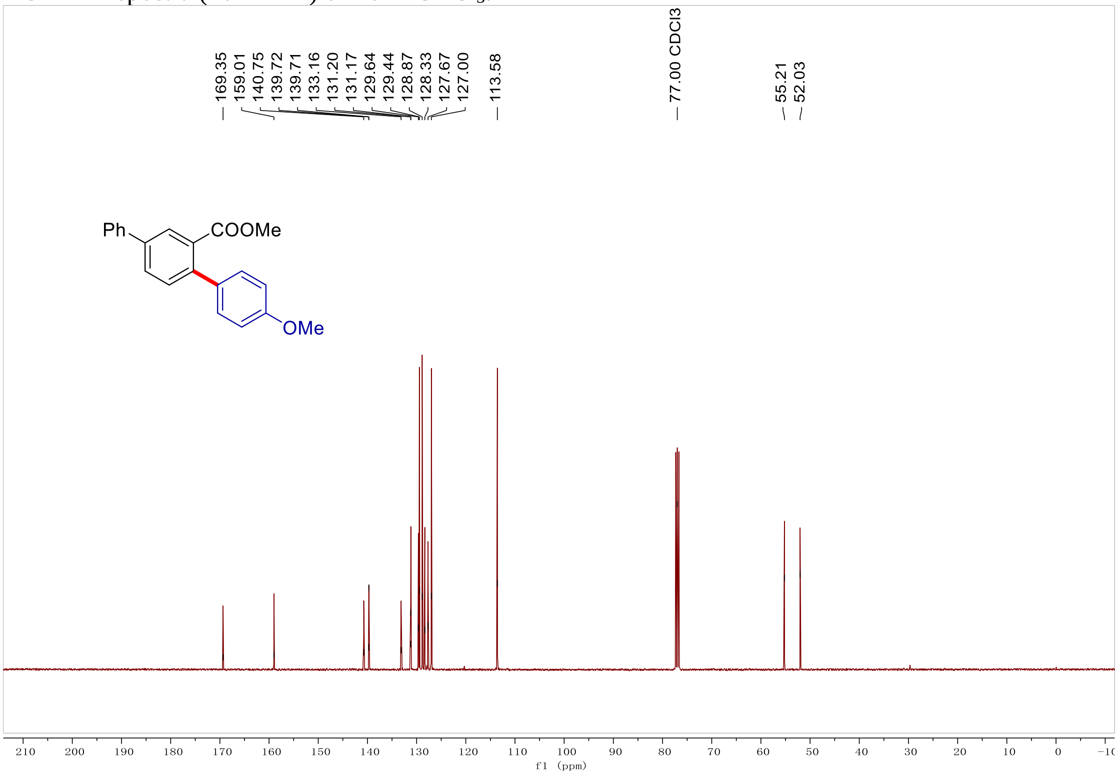

[1]**H NMR** spectra (400 MHz) of **17** in $CDCl_3$.

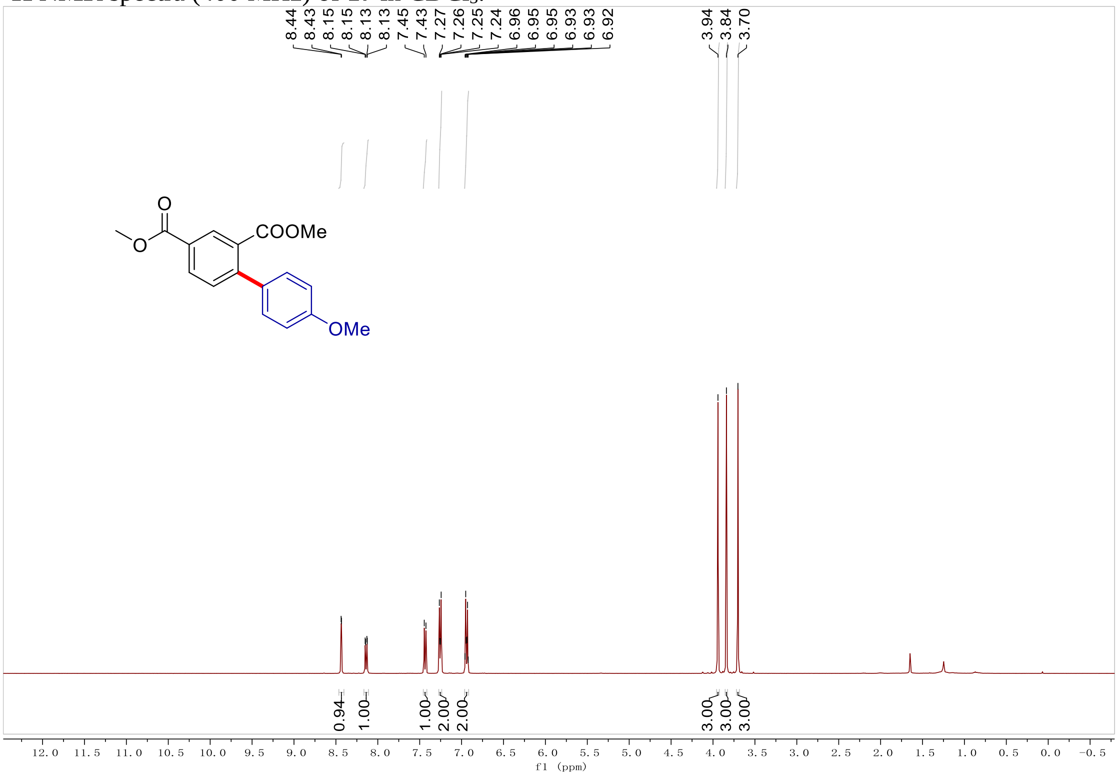

[13]**C NMR** spectra (101 MHz) of **17** in $CDCl_3$.

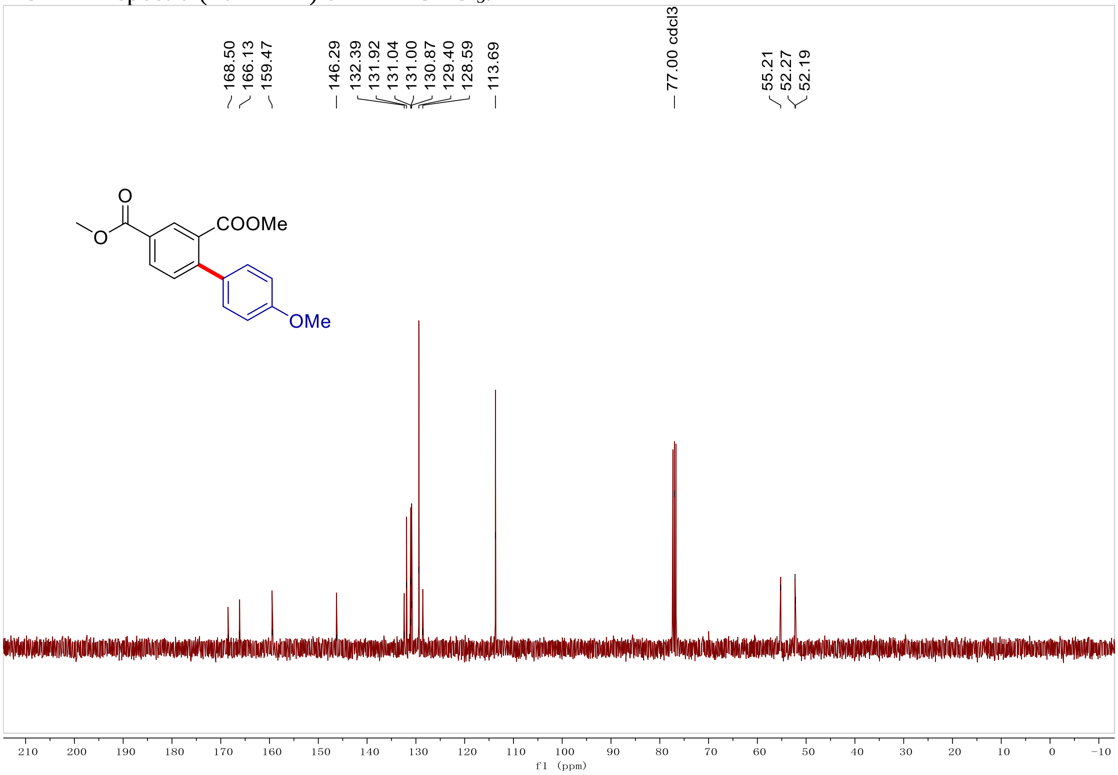

[1]**H NMR** spectra (400 MHz) of **18** in $CDCl_3$.

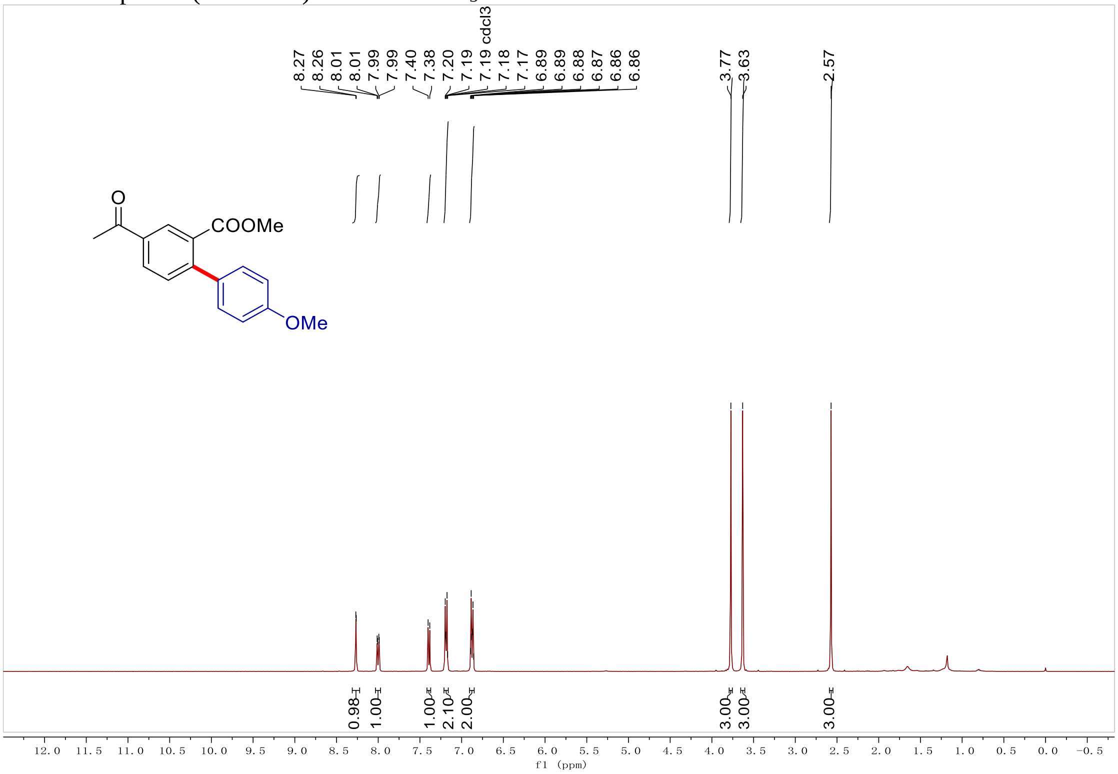

[13]**C NMR** spectra (101 MHz) of **18** in $CDCl_3$.

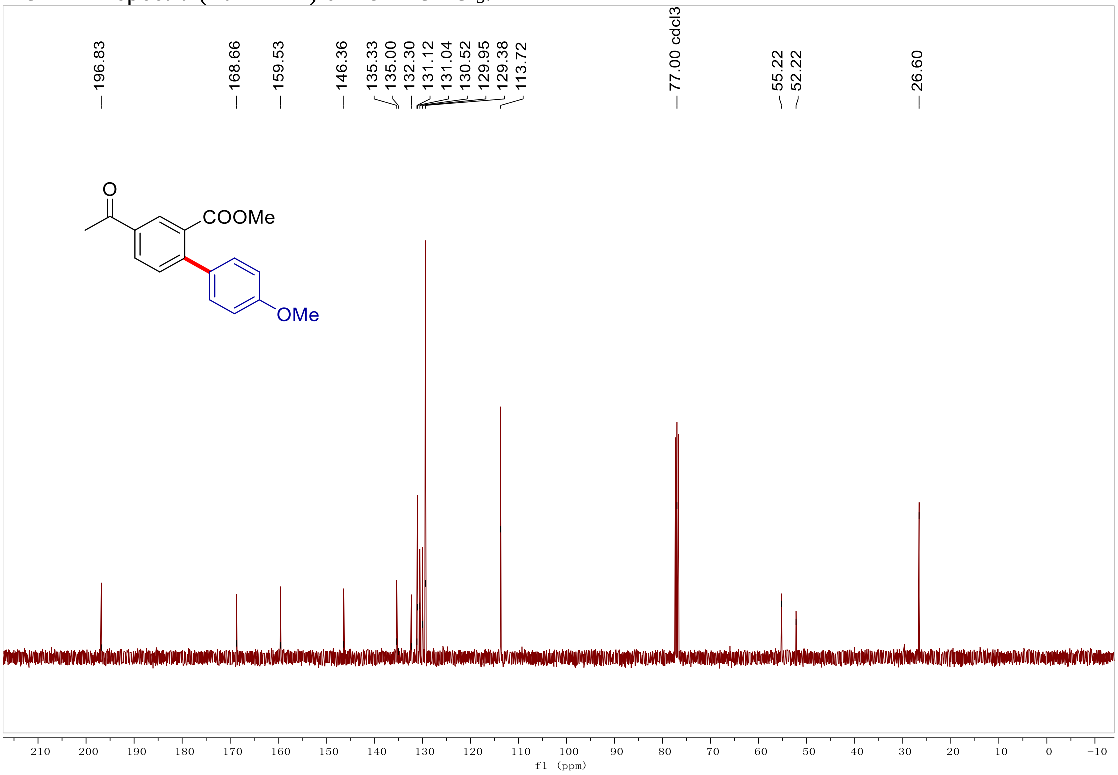

^{1}H NMR spectra (400 MHz) of **19** in $CDCl_3$.

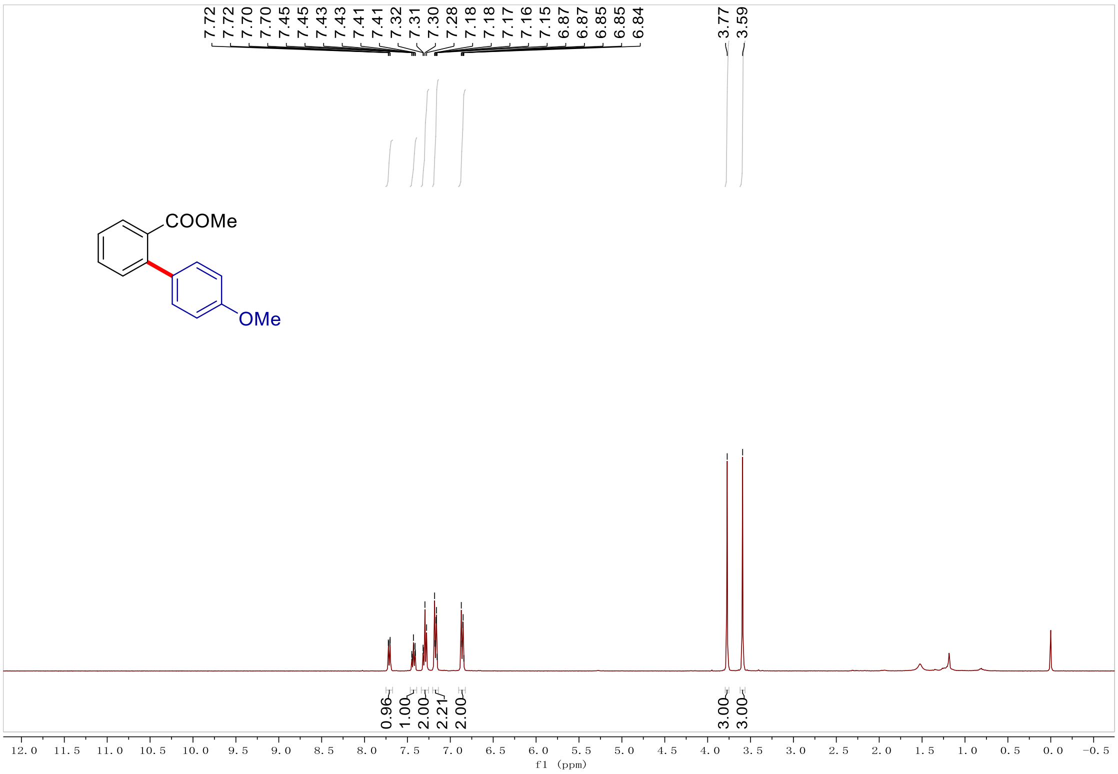

^{13}C NMR spectra (101 MHz) of **19** in $CDCl_3$.

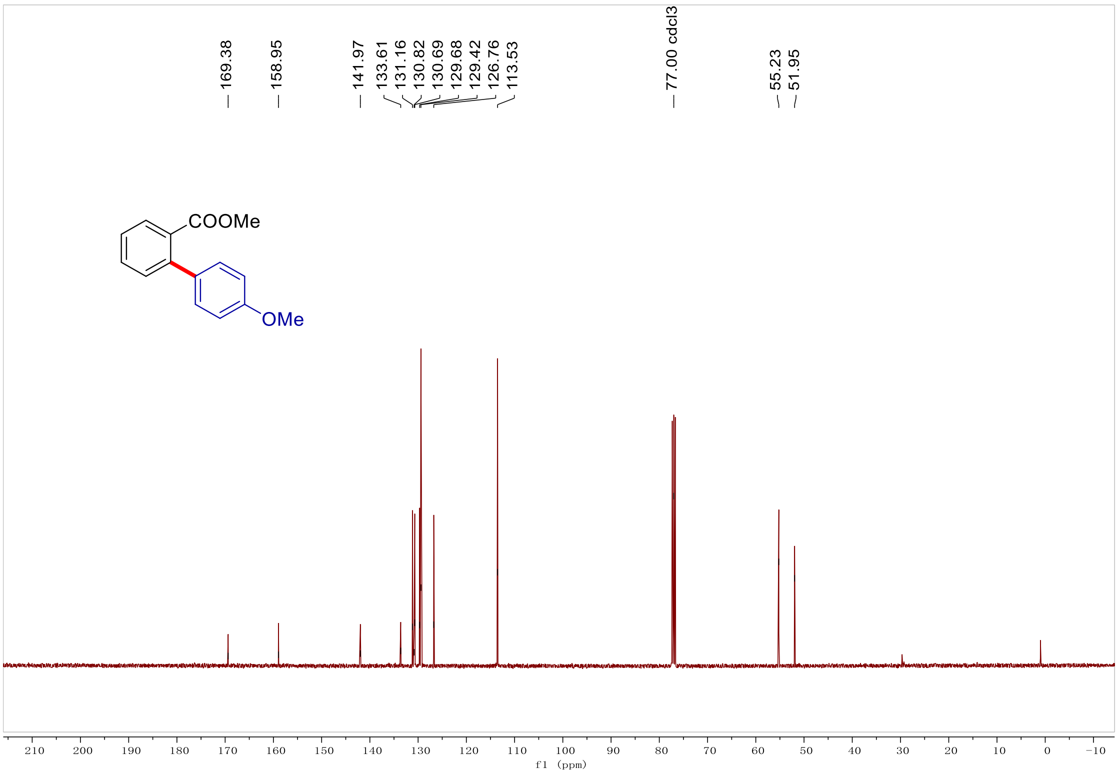

[1]**H NMR** spectra (400 MHz) of **19‘** in $CDCl_3$.

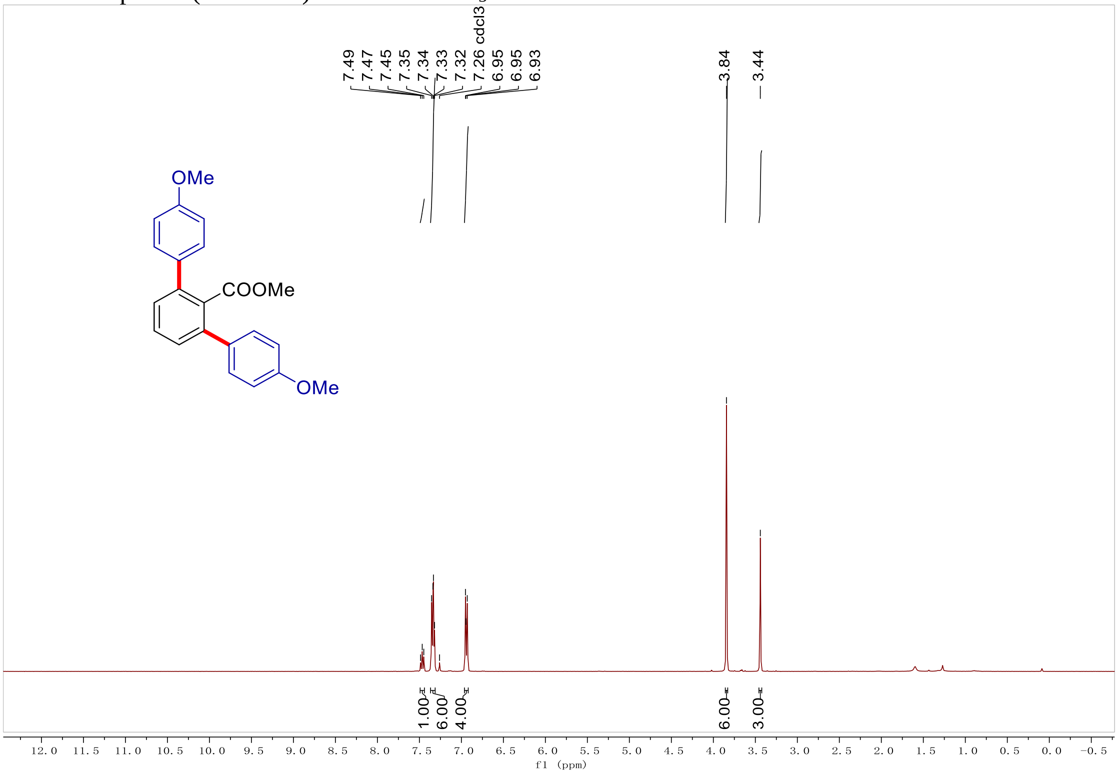

[13]**C NMR** spectra (101 MHz) of **19‘** in $CDCl_3$.

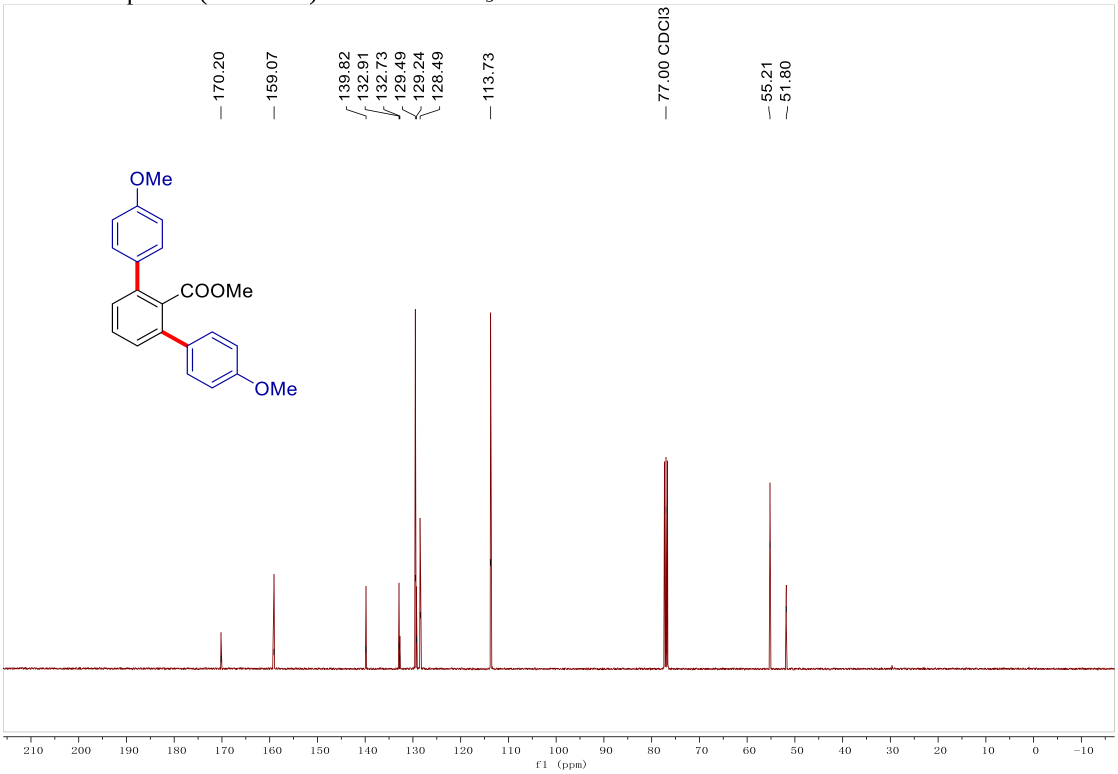

^{1}H NMR spectra (400 MHz) of **20** in $CDCl_3$.

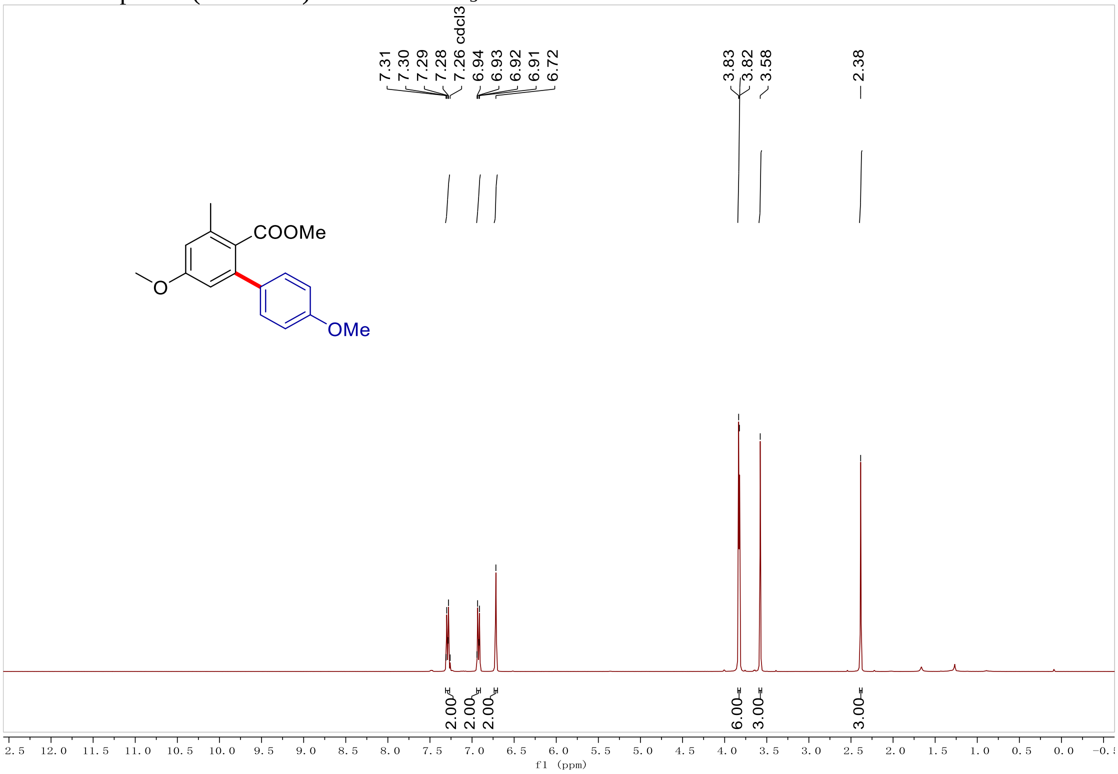

^{13}C NMR spectra (101 MHz) of **20** in $CDCl_3$.

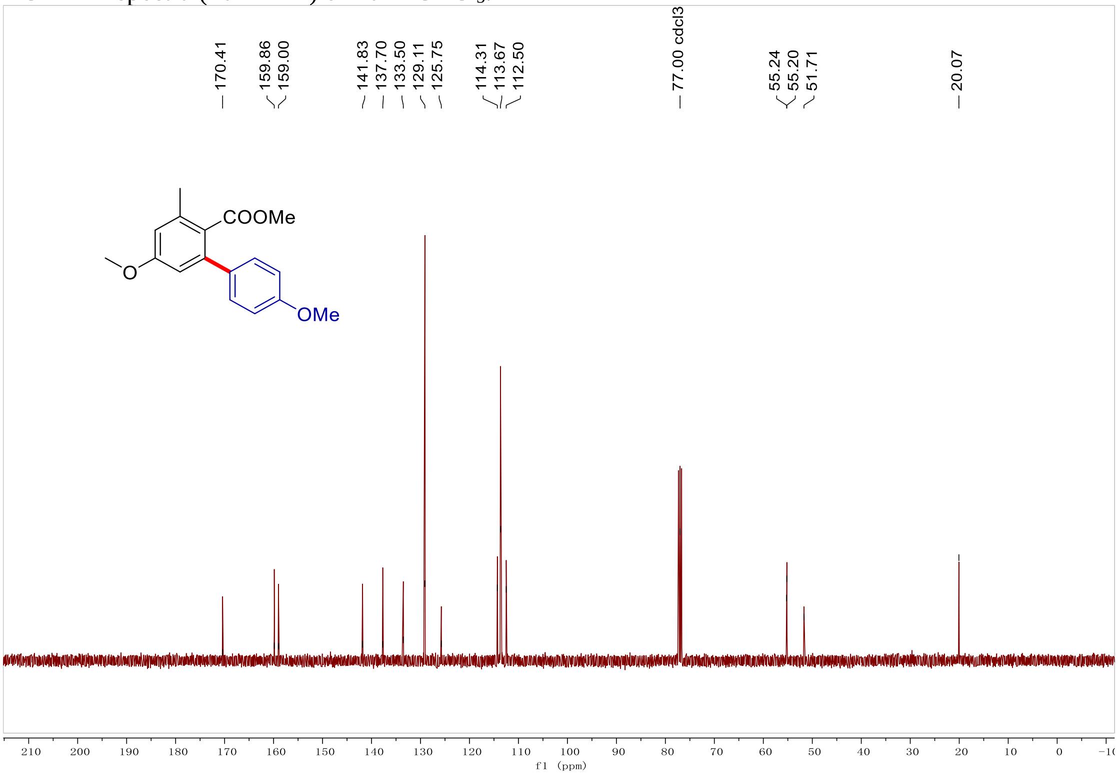

[1]**H NMR** spectra (400 MHz) of **21** in $CDCl_3$.

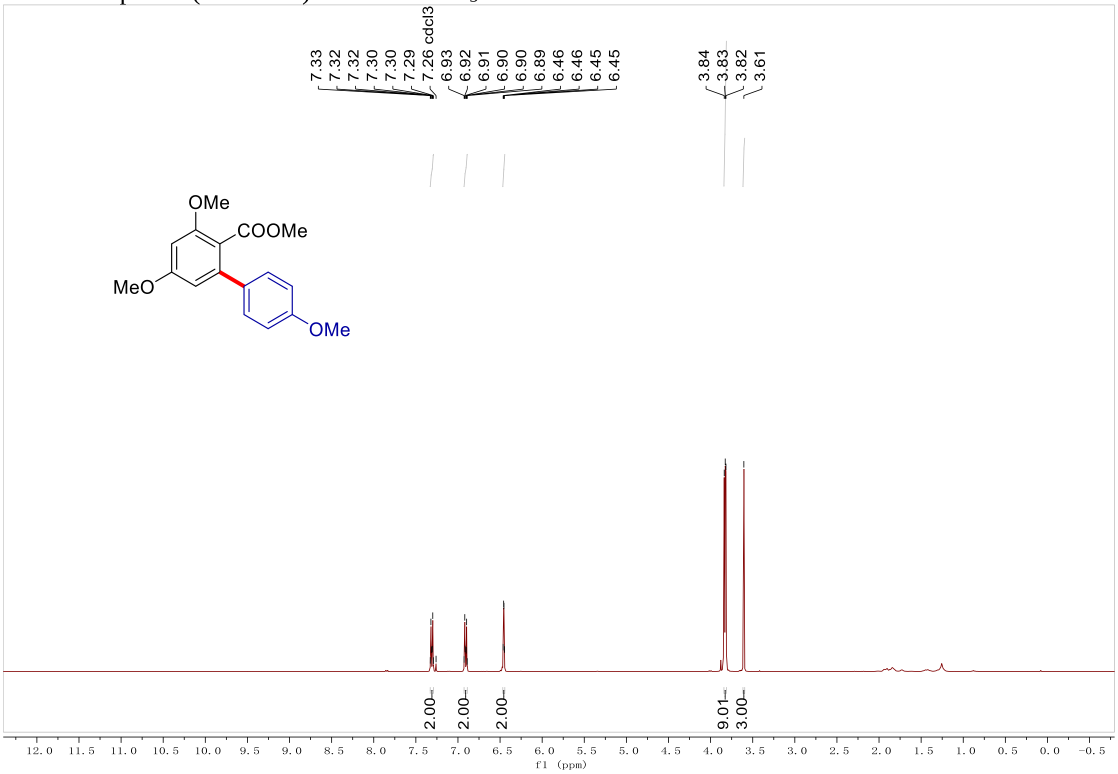

[13]**C NMR** spectra (101 MHz) of **21** in $CDCl_3$.

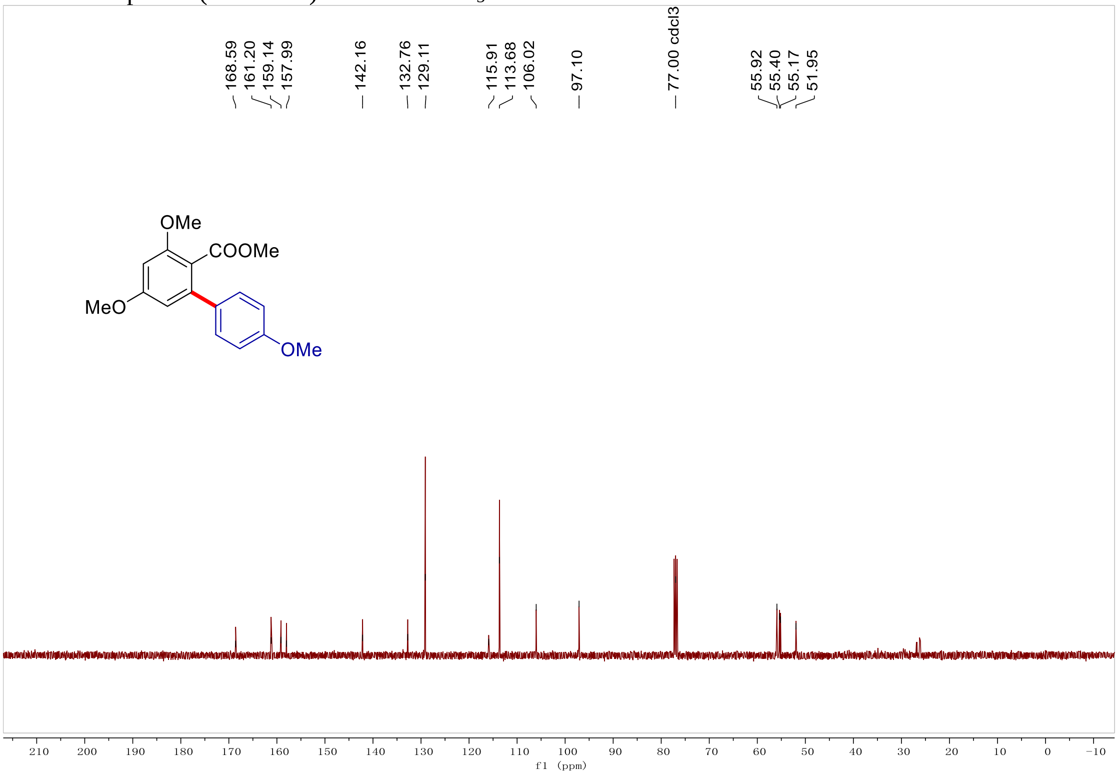

^{1}H NMR spectra (400 MHz) of **22** in CDCl3.

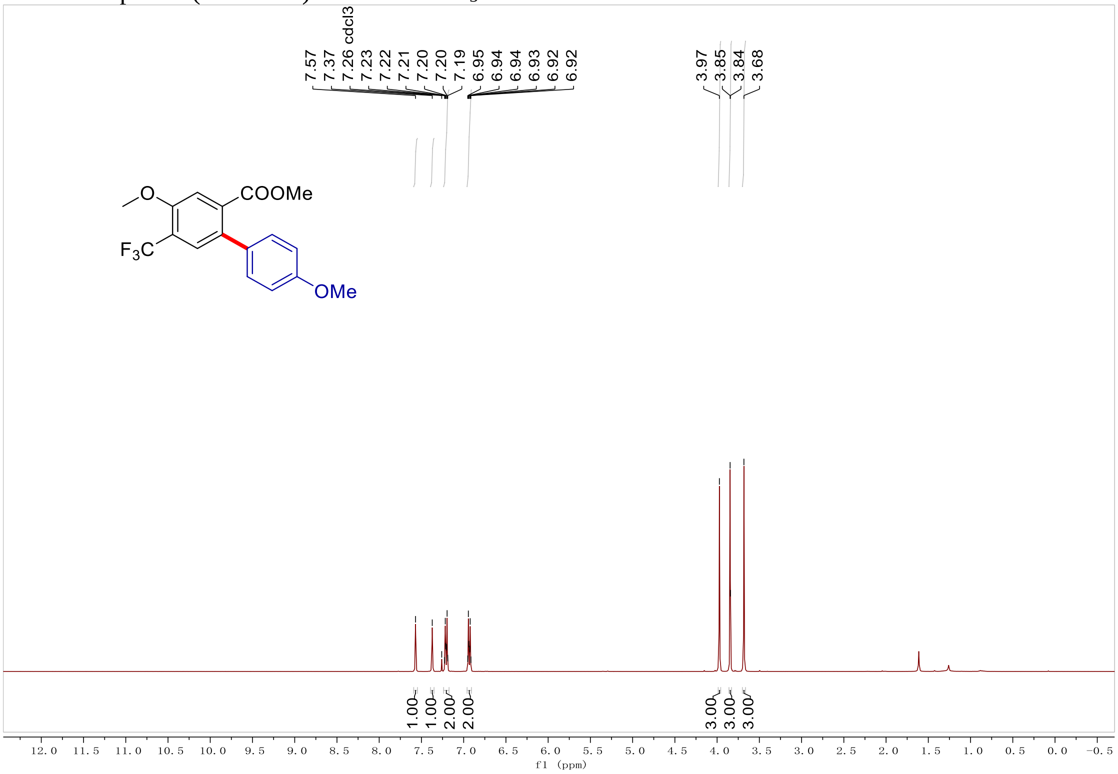

^{13}C NMR spectra (101 MHz) of **22** in CDCl3.

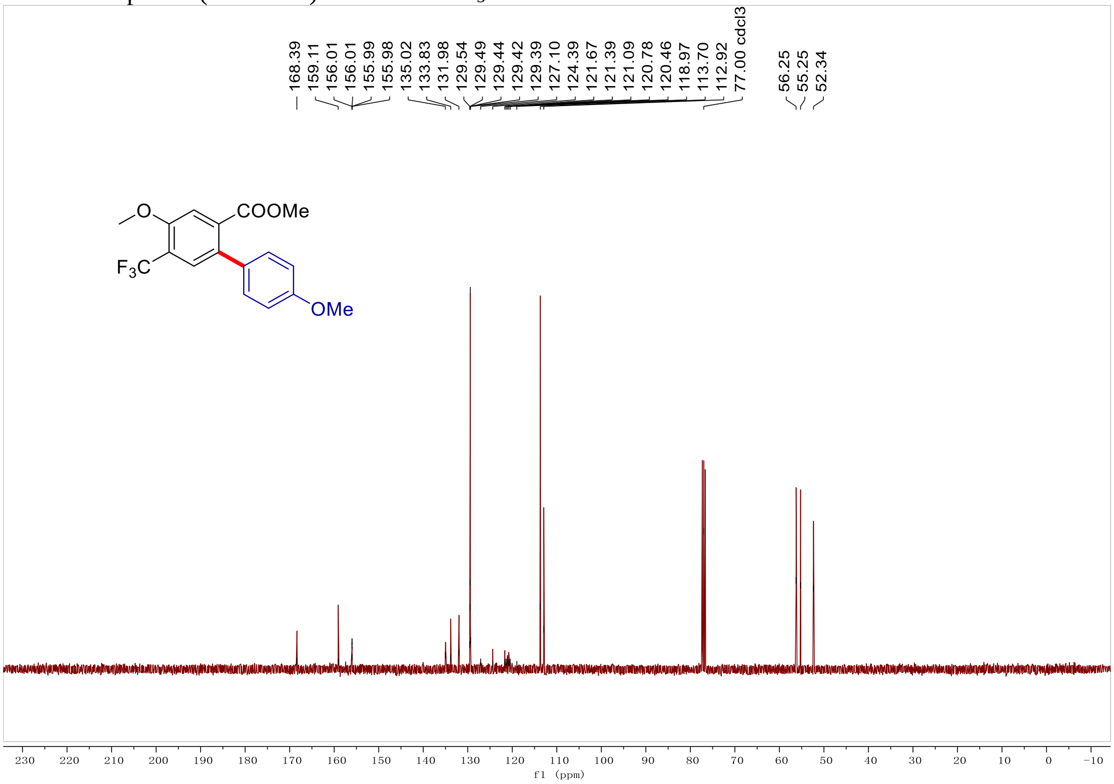

[1]**H NMR** spectra (400 MHz) of **23** in $CDCl_3$.

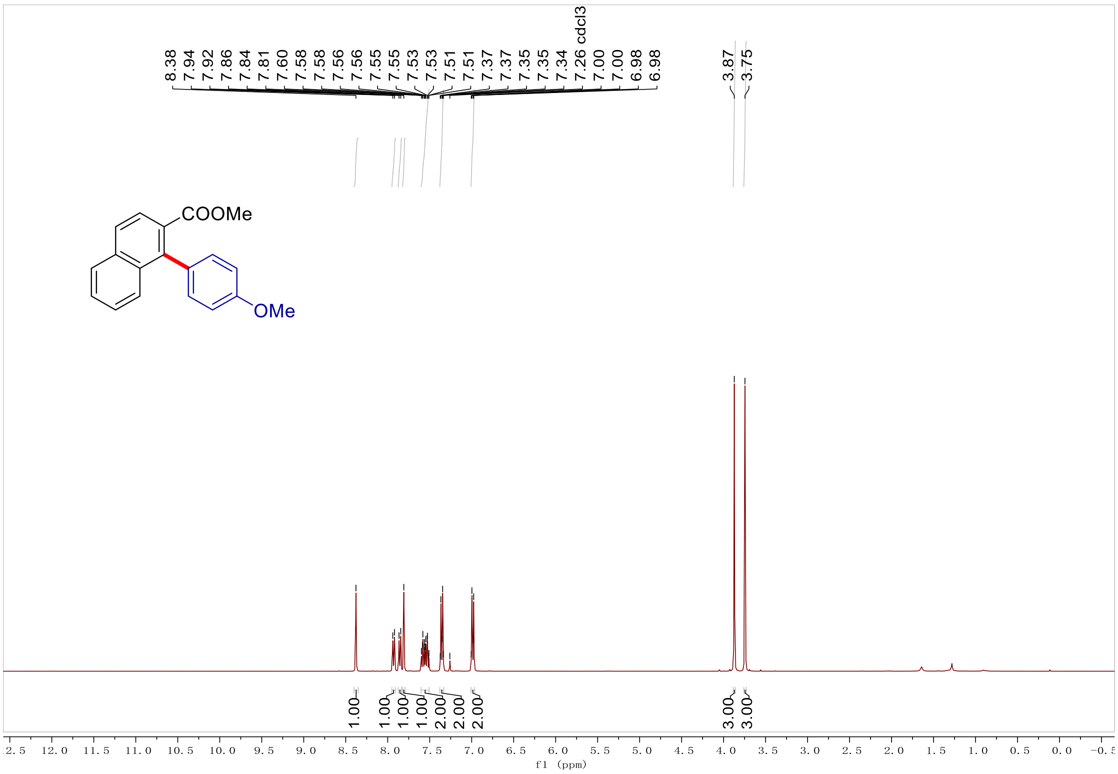

[13]**C NMR** spectra (101 MHz) of **23** in $CDCl_3$.

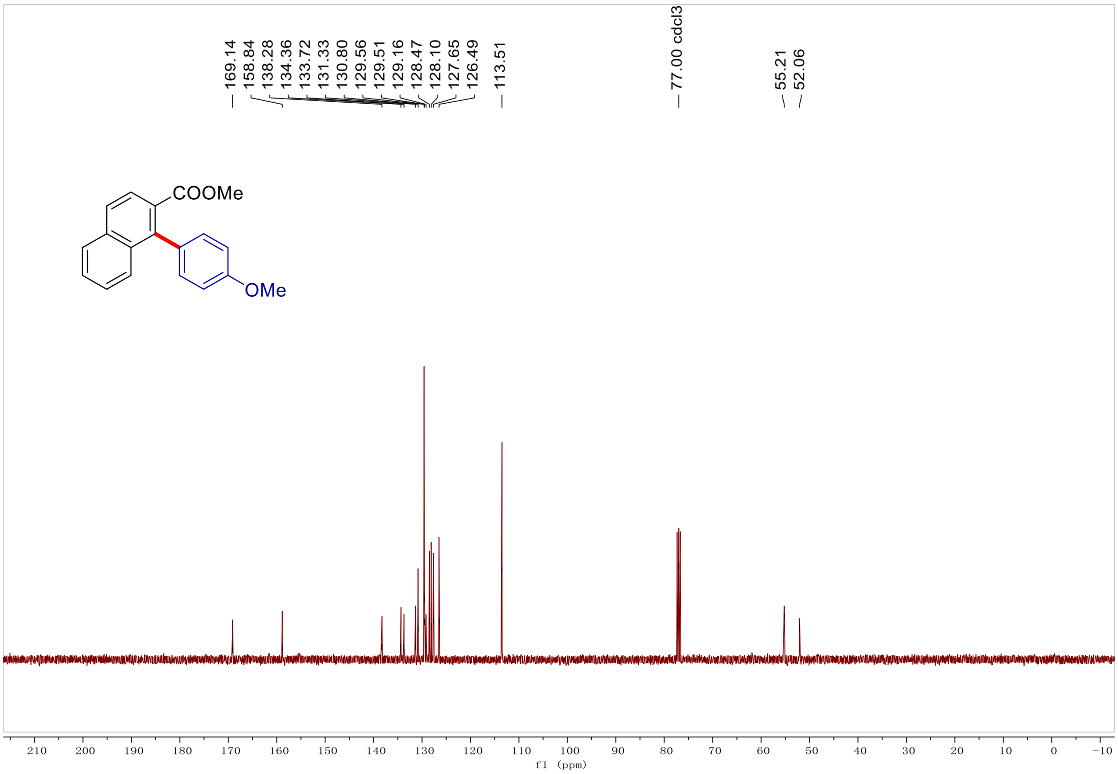

^{1}H NMR spectra (400 MHz) of **24** in $CDCl_3$.

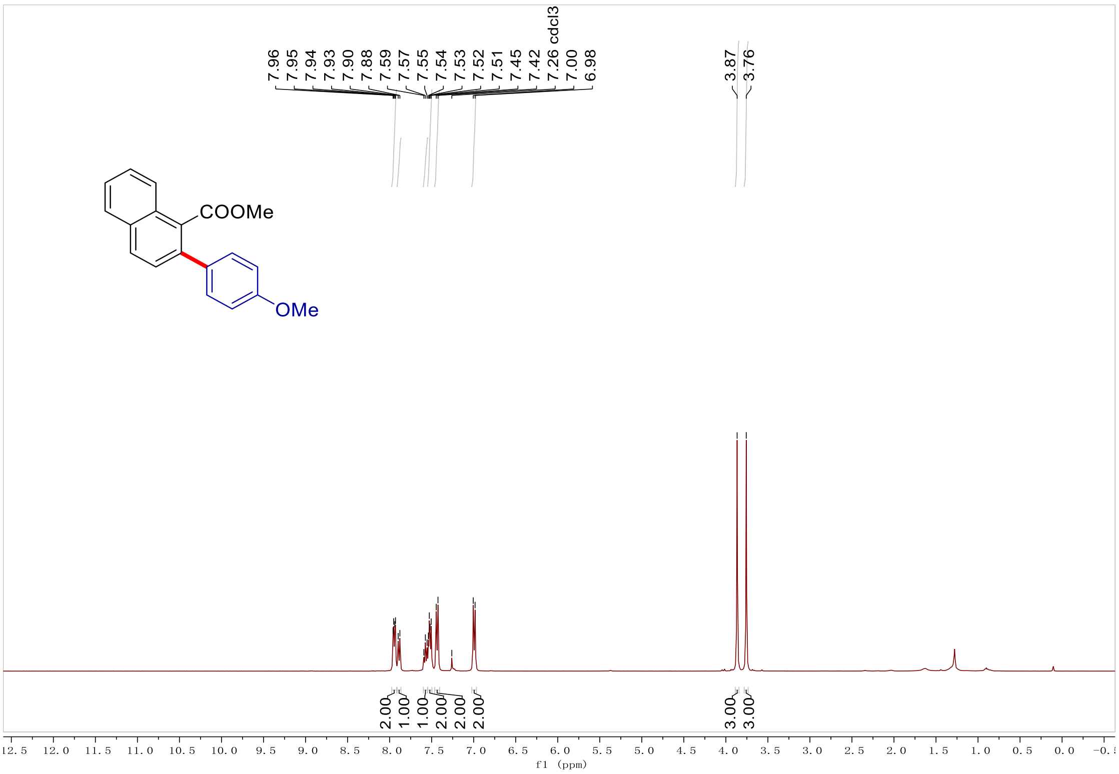

^{13}C NMR spectra (101 MHz) of **24** in $CDCl_3$.

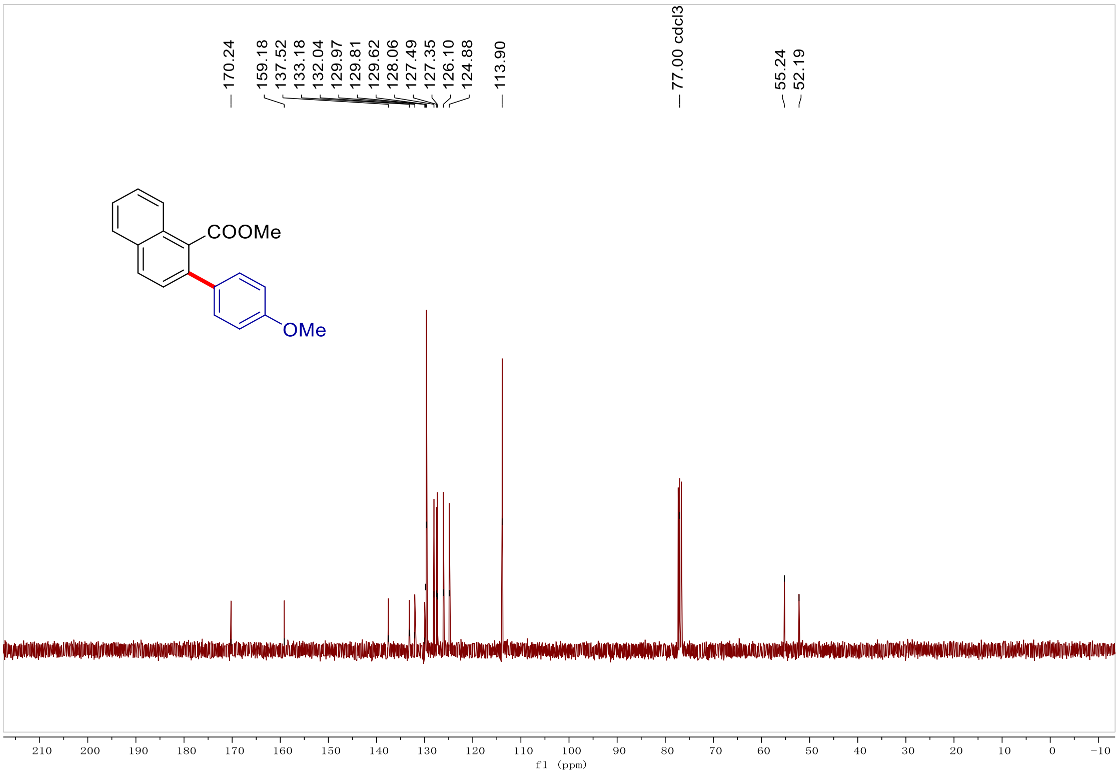

^{1}H NMR spectra (400 MHz) of **25** in $CDCl_3$.

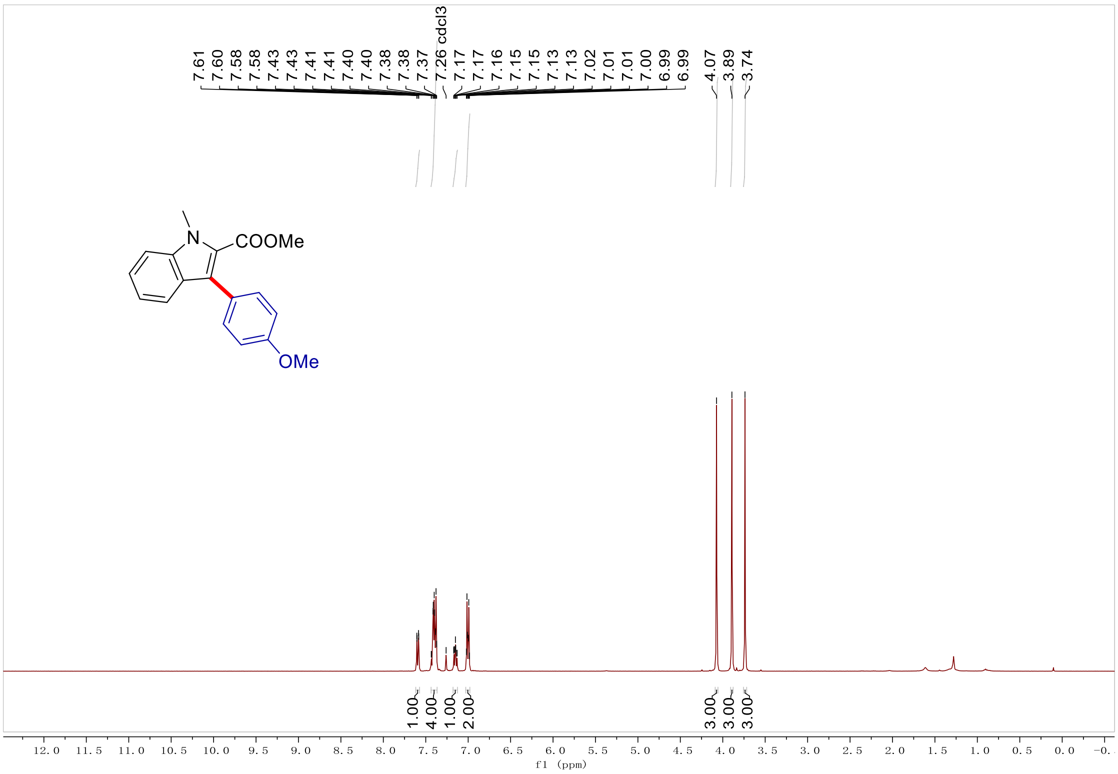

^{13}C NMR spectra (101 MHz) of **25** in $CDCl_3$.

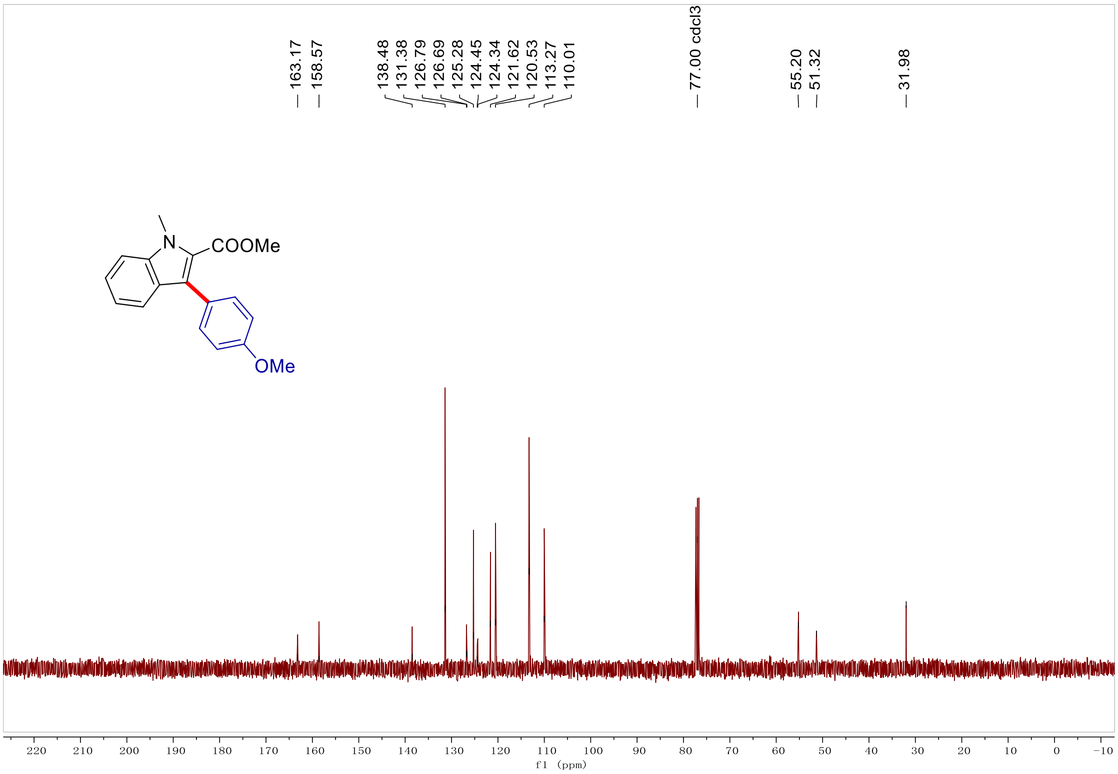

^{1}H NMR spectra (400 MHz) of **26** in $CDCl_3$.

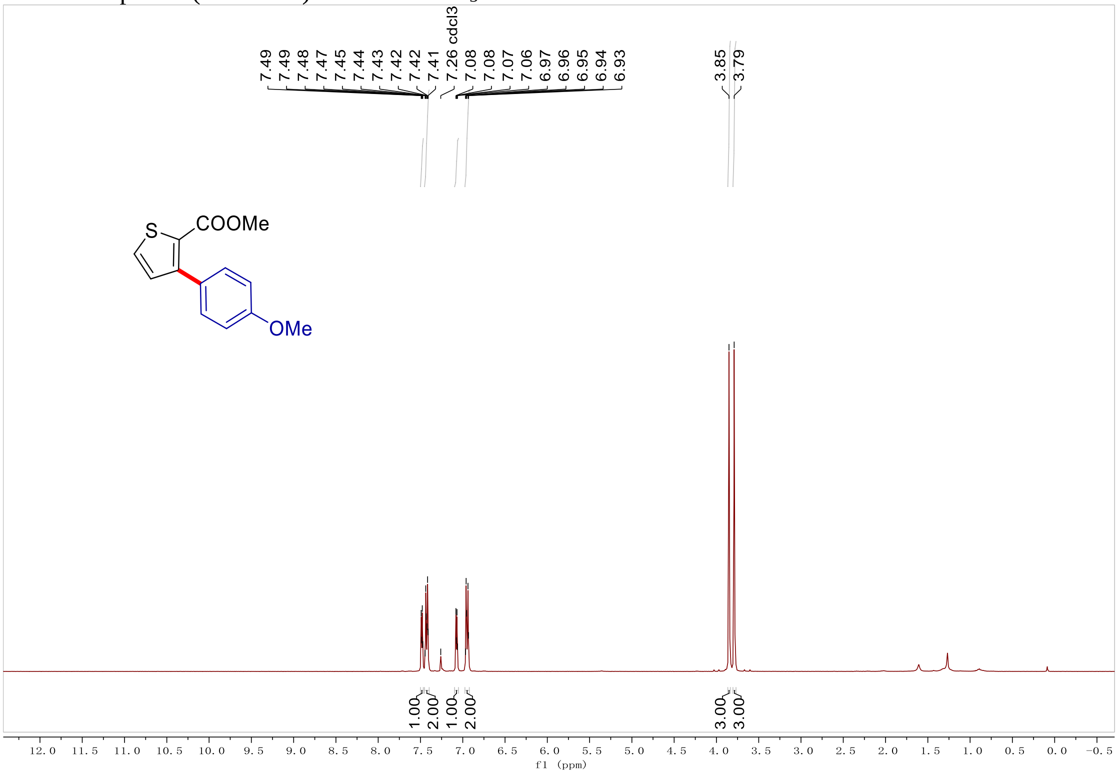

^{13}C NMR spectra (101 MHz) of **26** in $CDCl_3$.

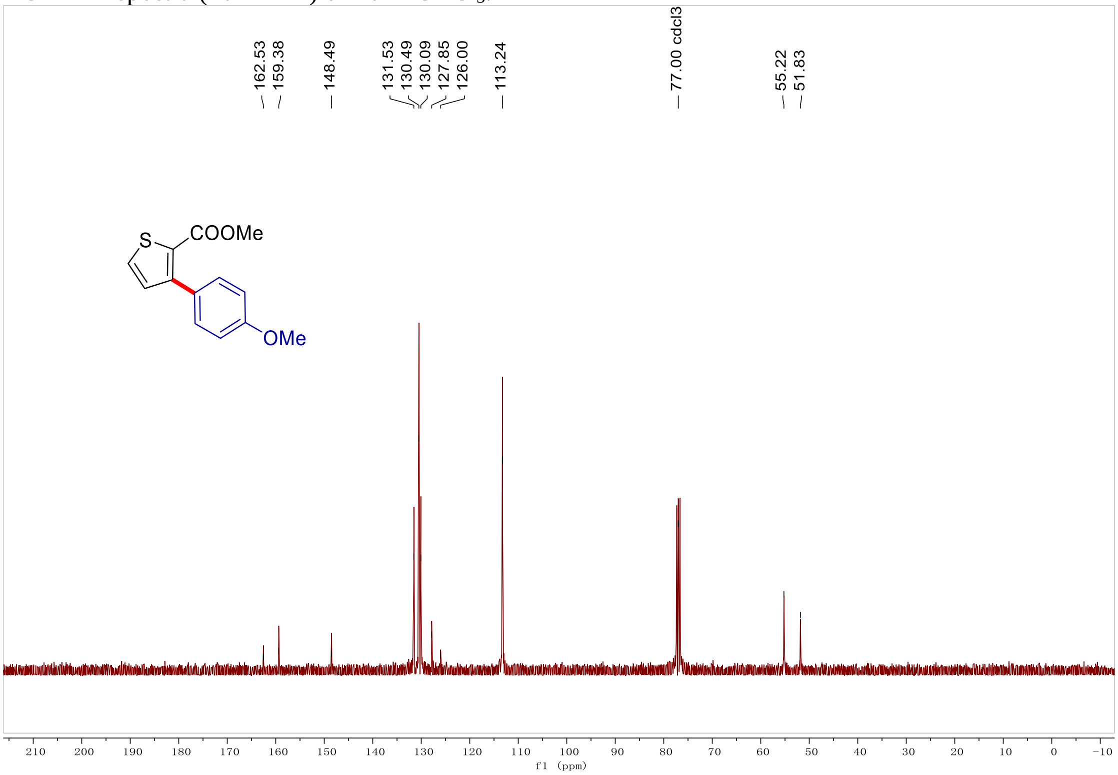

[1]**H NMR** spectra (400 MHz) of **27** in $CDCl_3$.

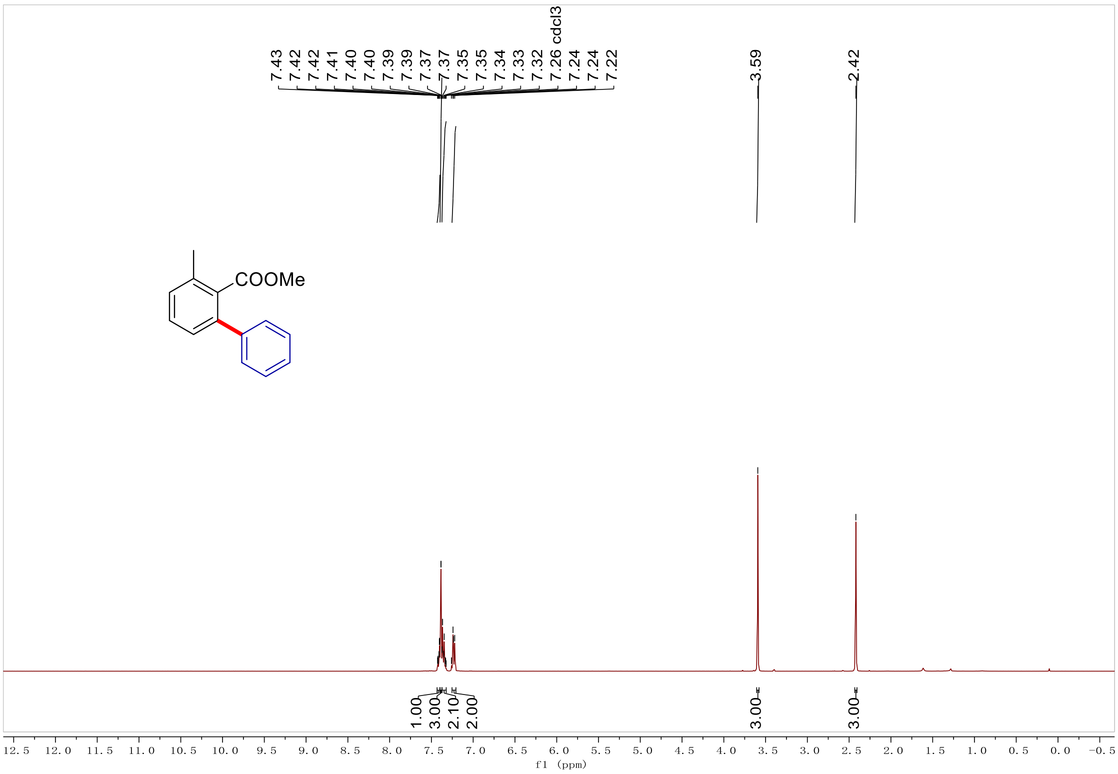

[13]**C NMR** spectra (101 MHz) of **27** in $CDCl_3$.

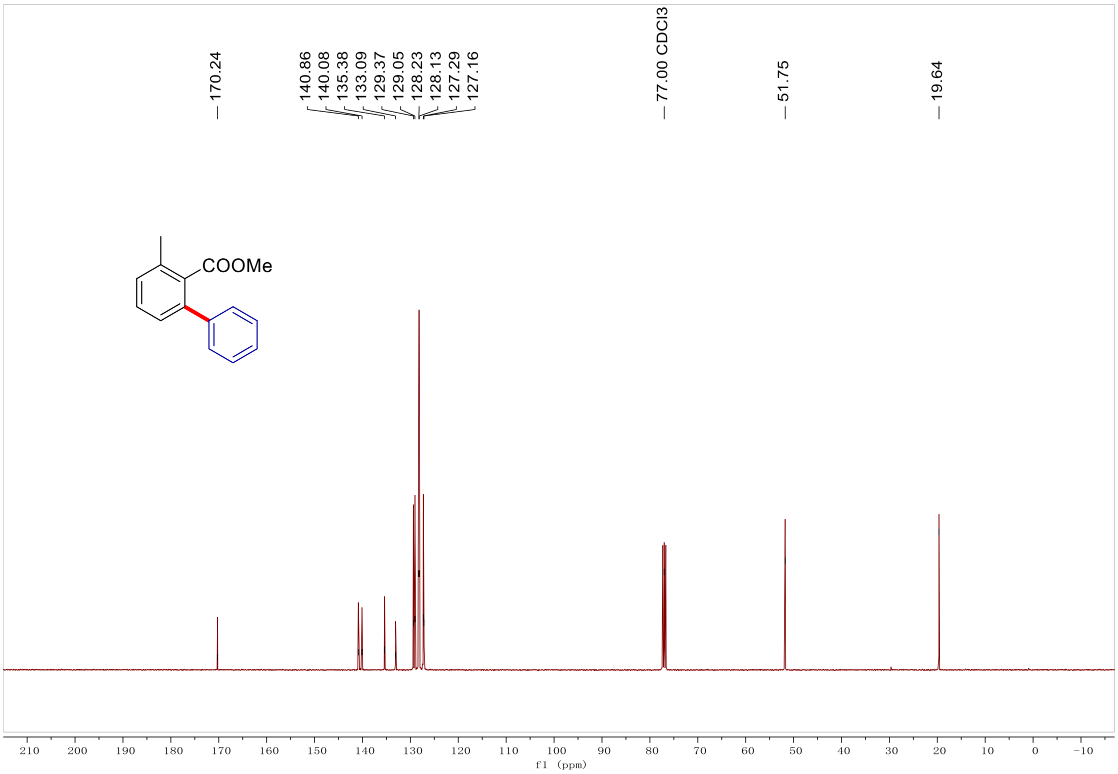

[1]**H NMR** spectra (400 MHz) of **28** in $CDCl_3$.

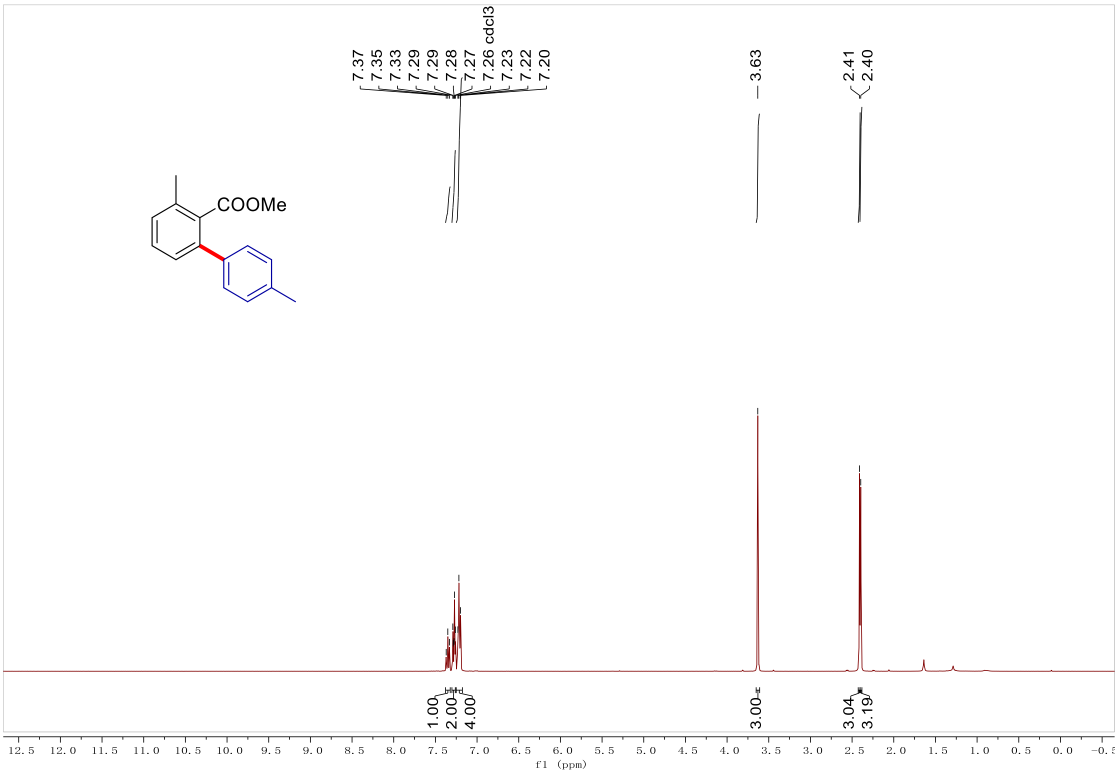

[13]**C NMR** spectra (101 MHz) of **28** in $CDCl_3$.

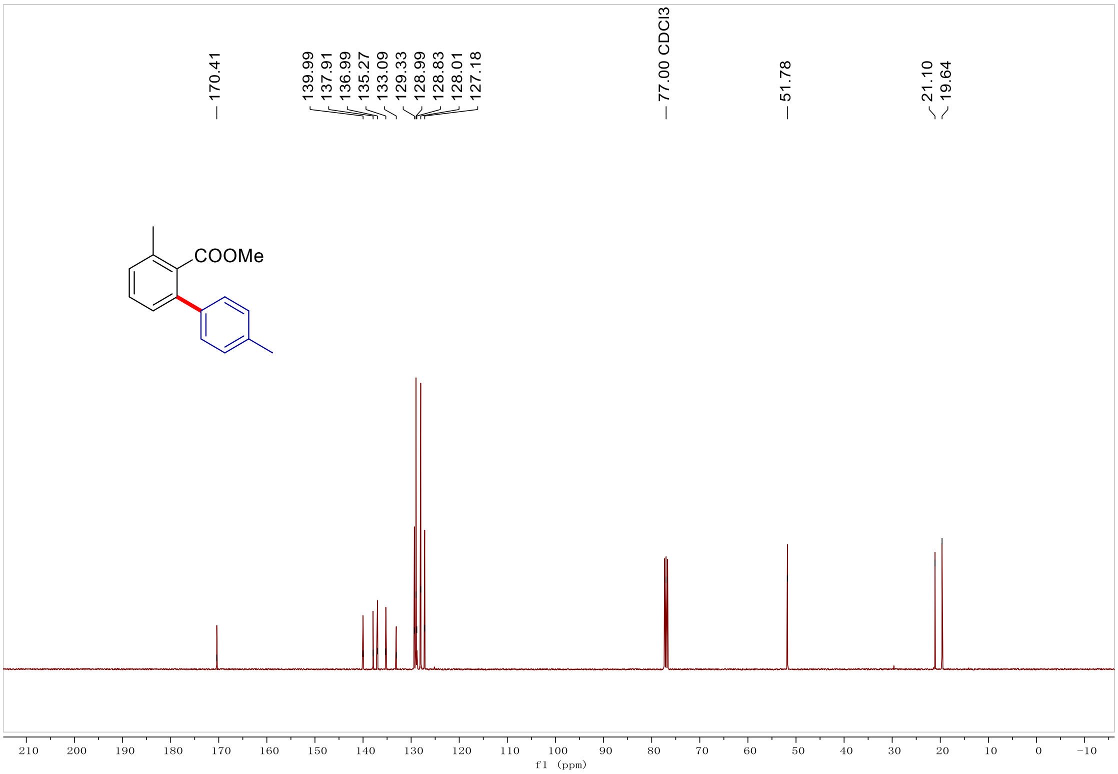

^{1}H NMR spectra (400 MHz) of **29** in $CDCl_3$.

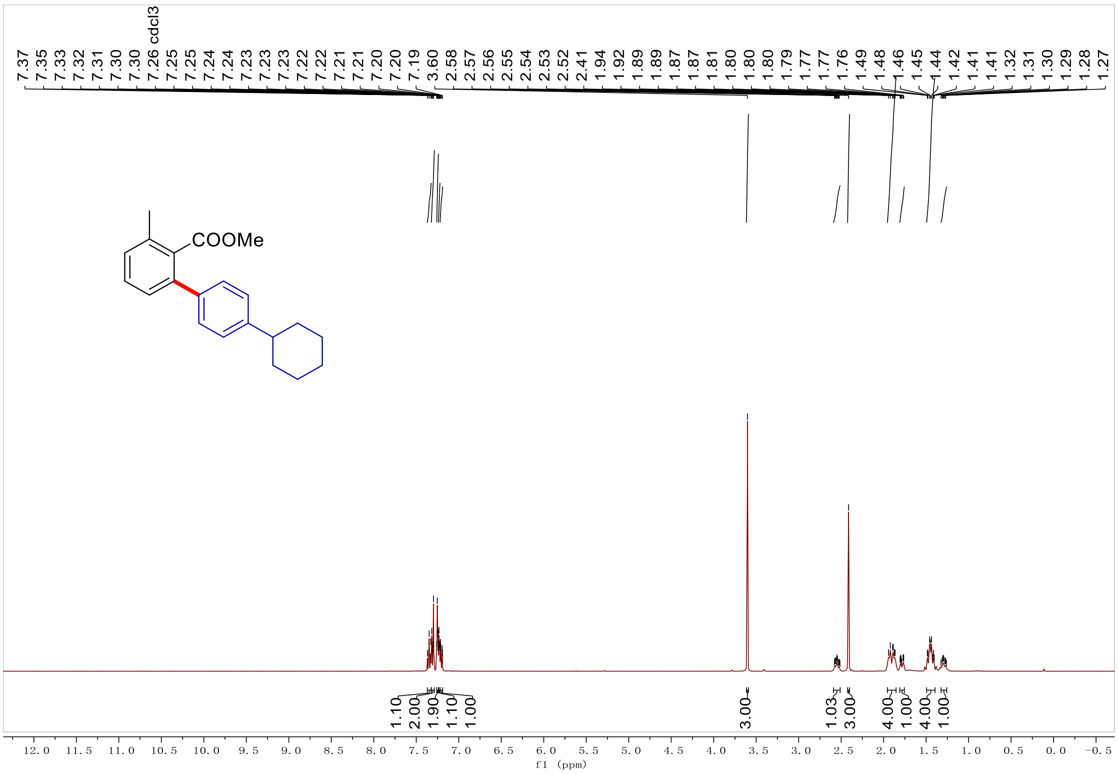

^{13}C NMR spectra (101 MHz) of **29** in $CDCl_3$.

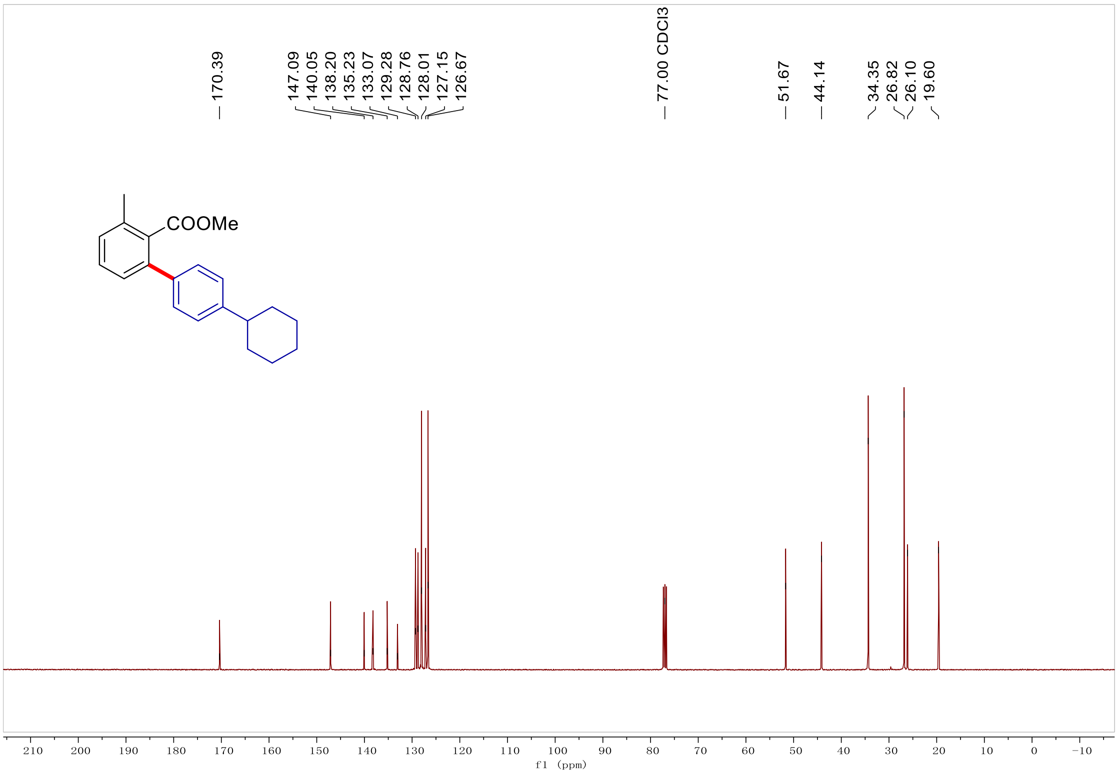

[1]**H NMR** spectra (400 MHz) of **30** in $CDCl_3$.

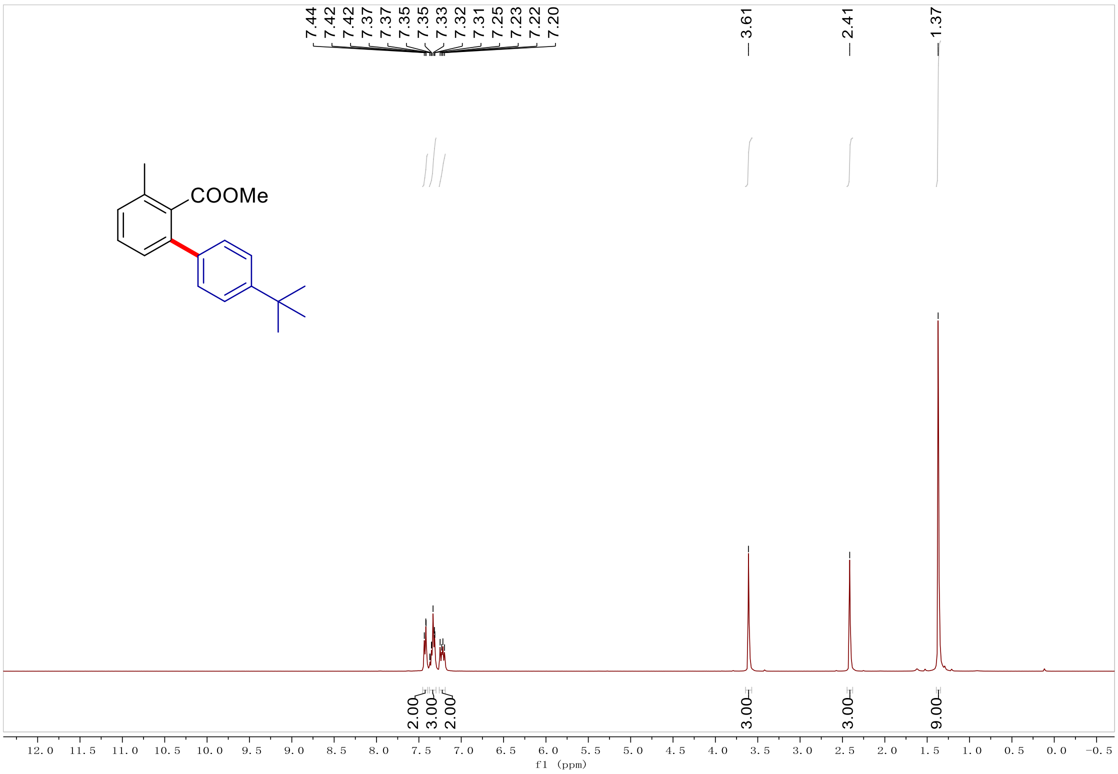

[13]**C NMR** spectra (101 MHz) of **30** in $CDCl_3$.

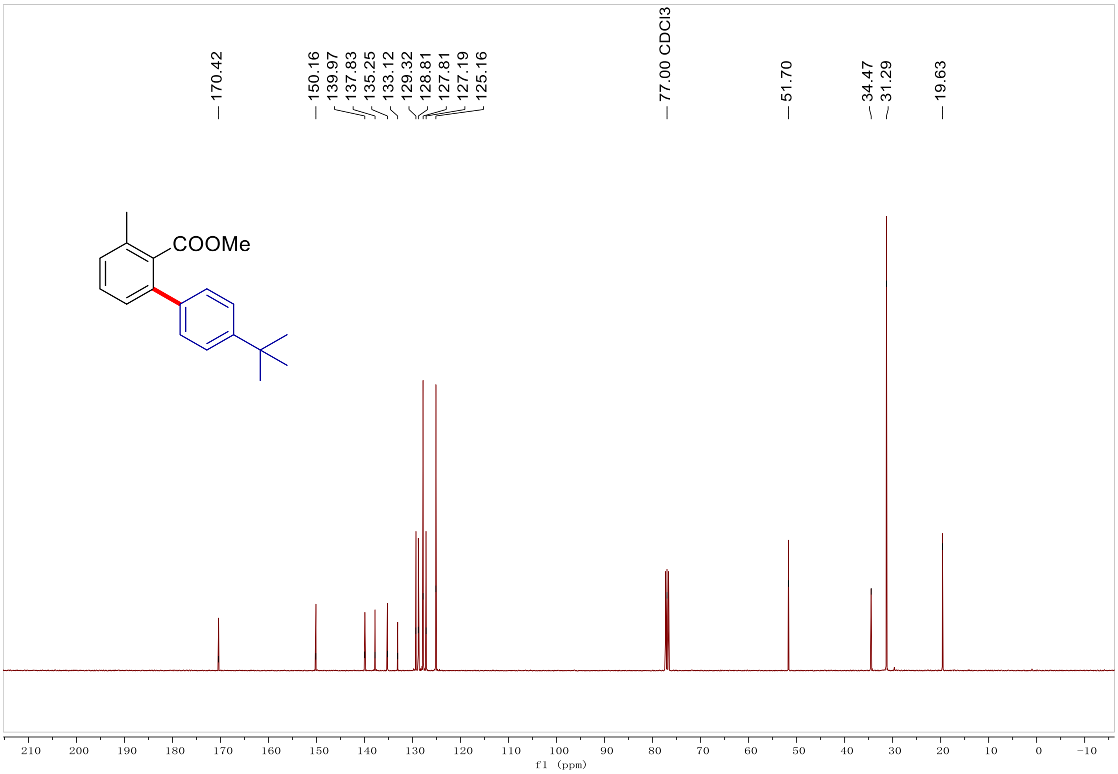

^{1}H NMR spectra (400 MHz) of **31** in $CDCl_3$.

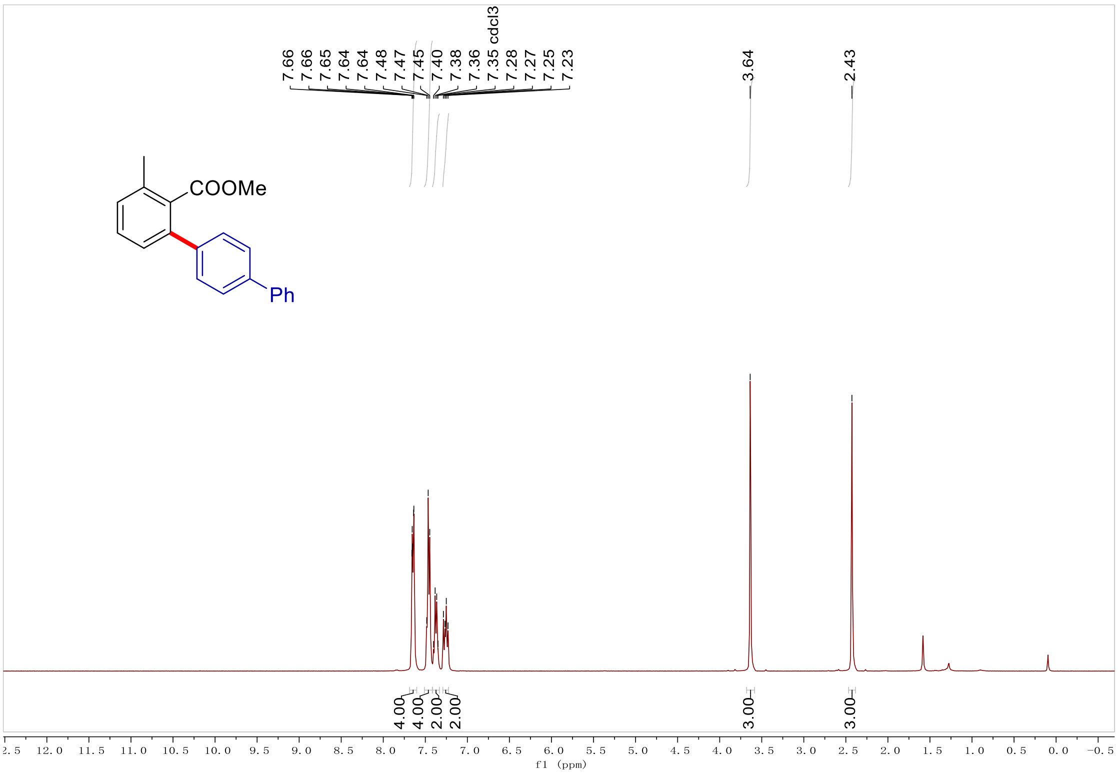

^{13}C NMR spectra (101 MHz) of **31** in $CDCl_3$.

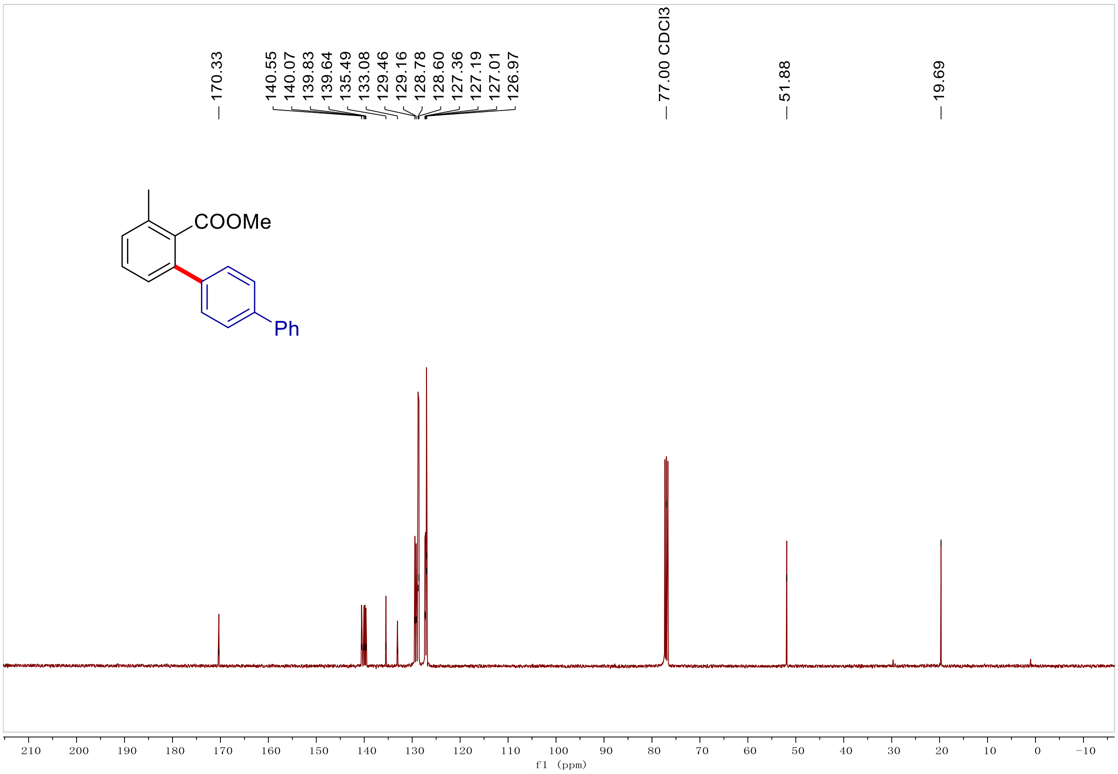

^{1}H NMR spectra (400 MHz) of **32** in $CDCl_3$.

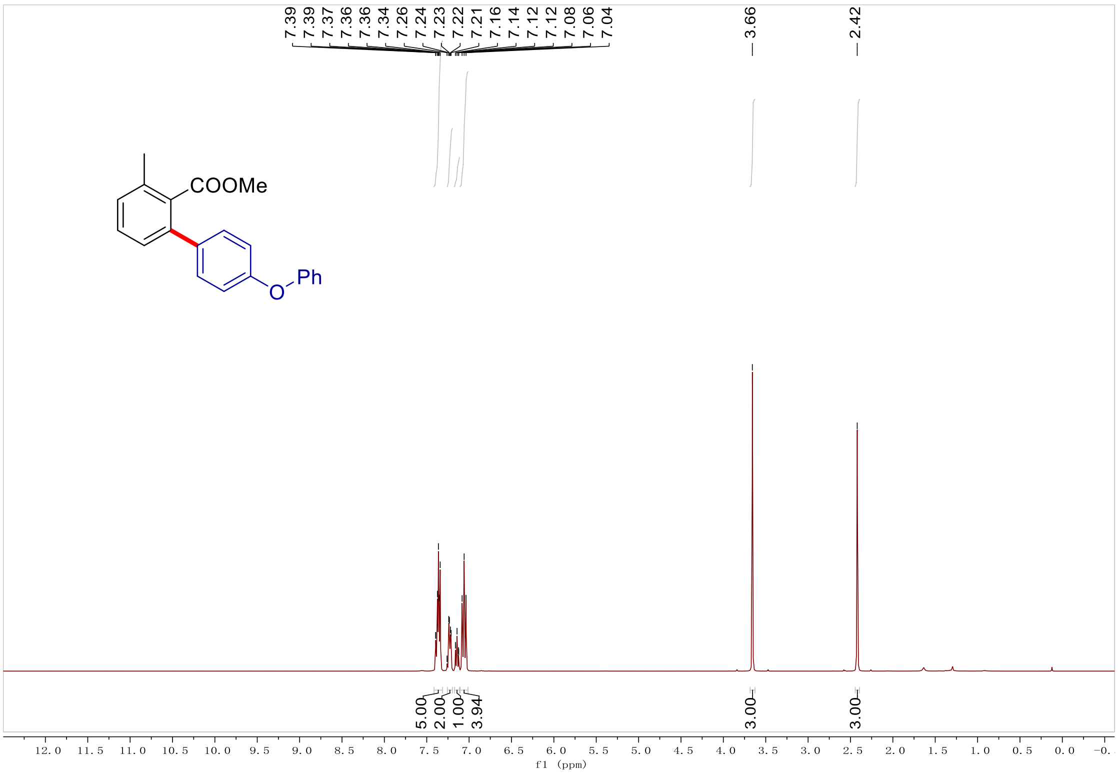

^{13}C NMR spectra (101 MHz) of **32** in $CDCl_3$.

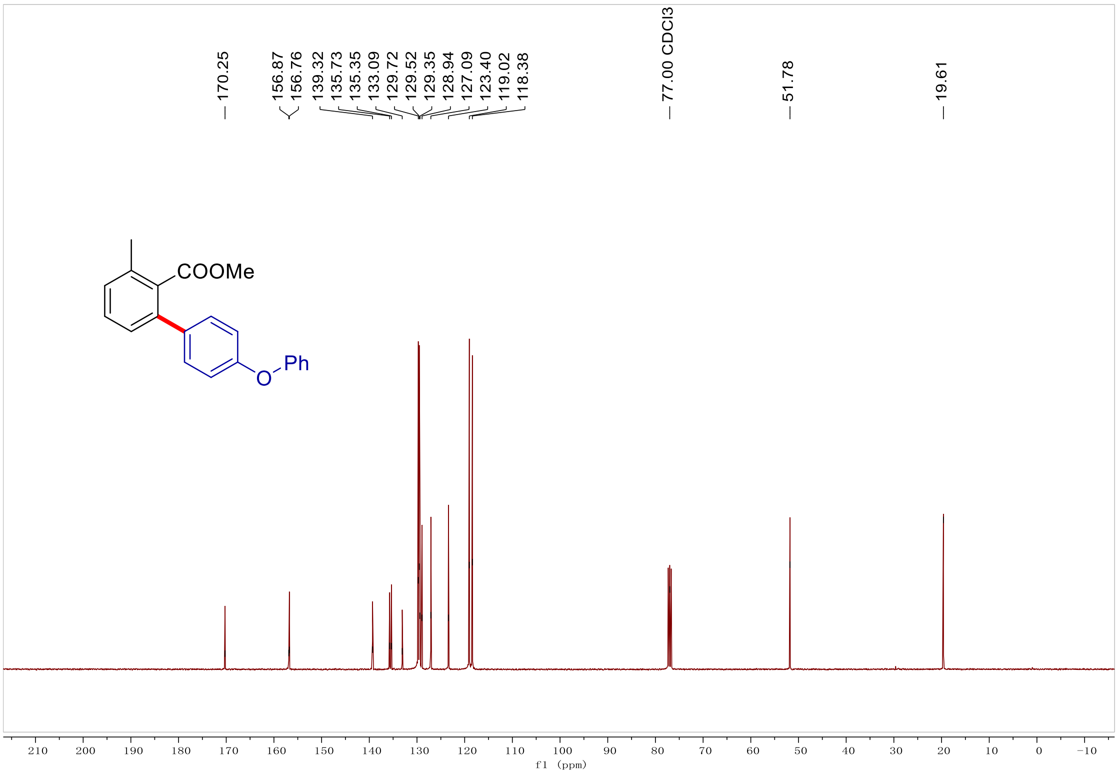

[1]**H NMR** spectra (400 MHz) of **33** in $CDCl_3$.

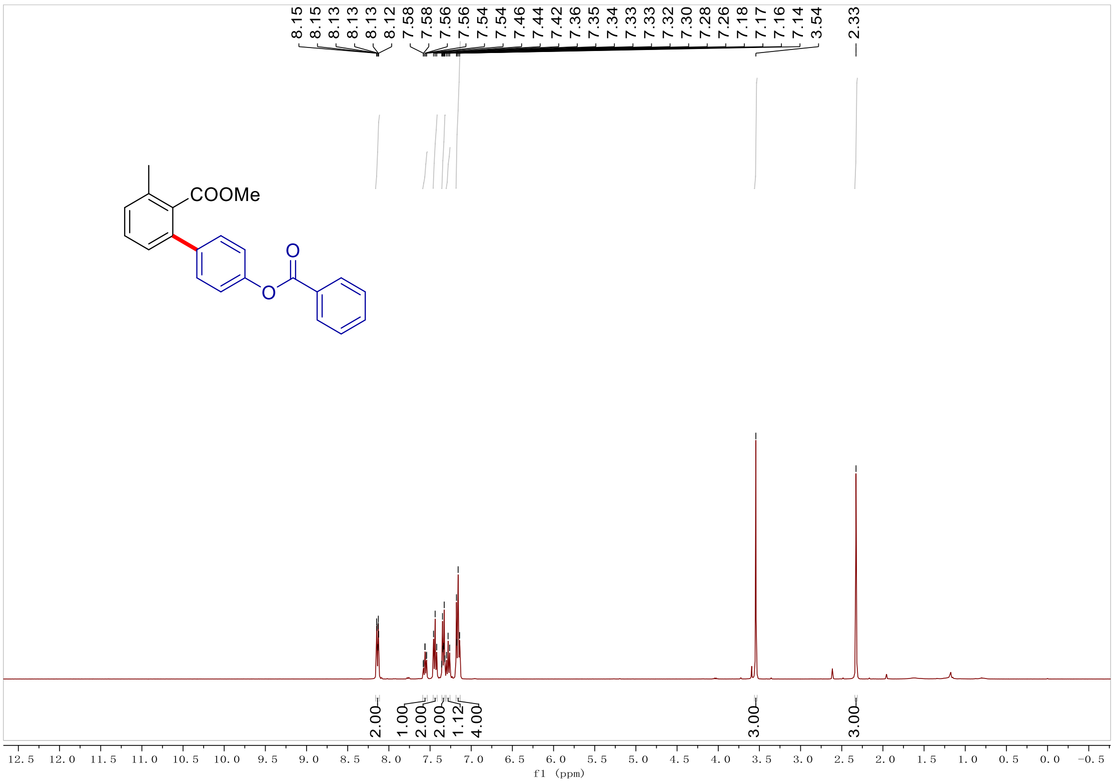

[13]**C NMR** spectra (101 MHz) of **33** in $CDCl_3$.

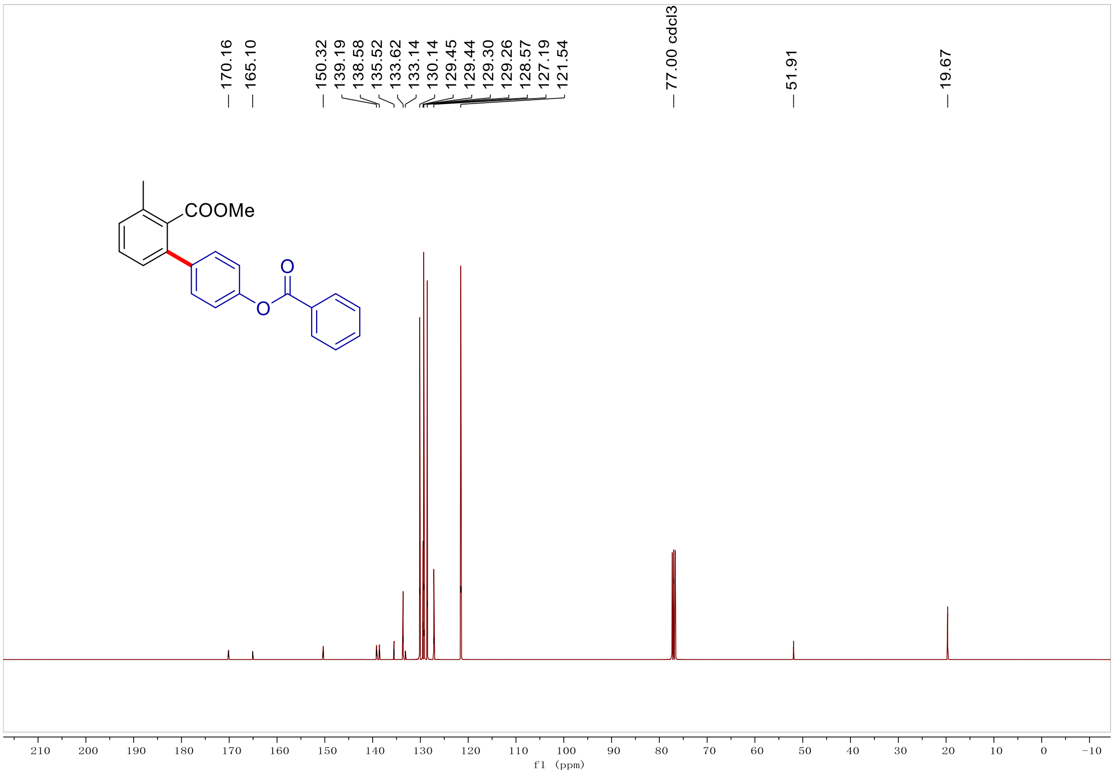

[1]**H NMR** spectra (400 MHz) of **34** in $CDCl_3$.

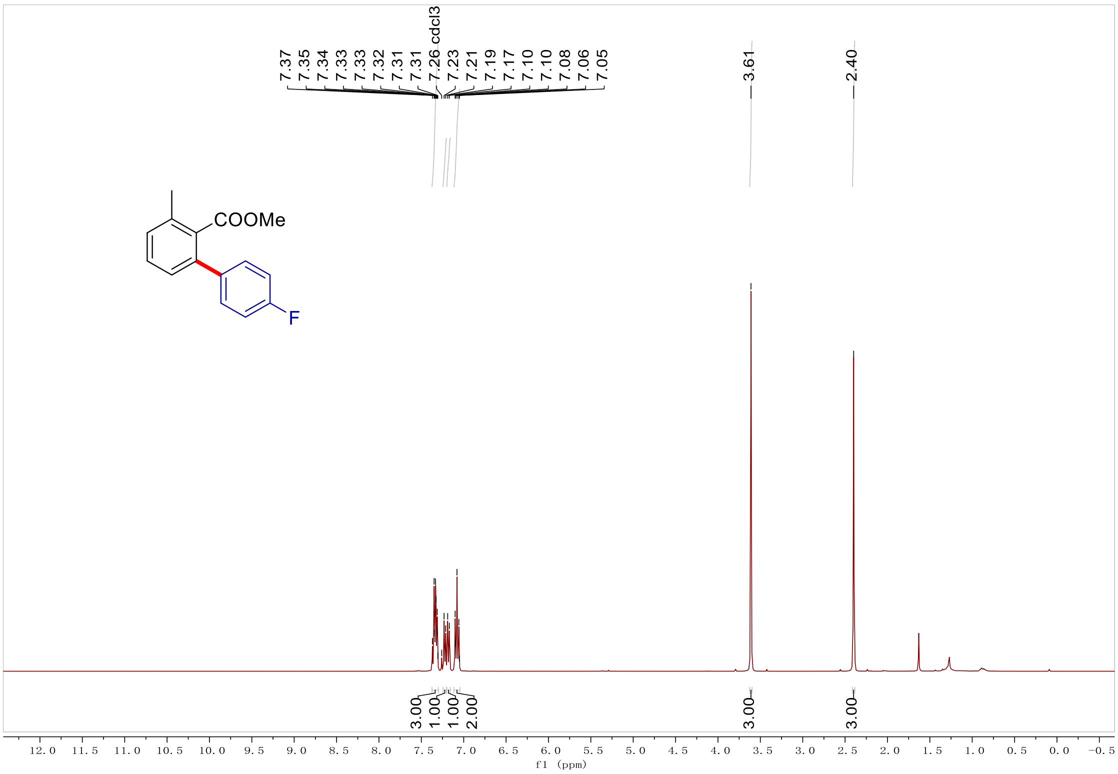

[13]**C NMR** spectra (101 MHz) of **34** in $CDCl_3$.

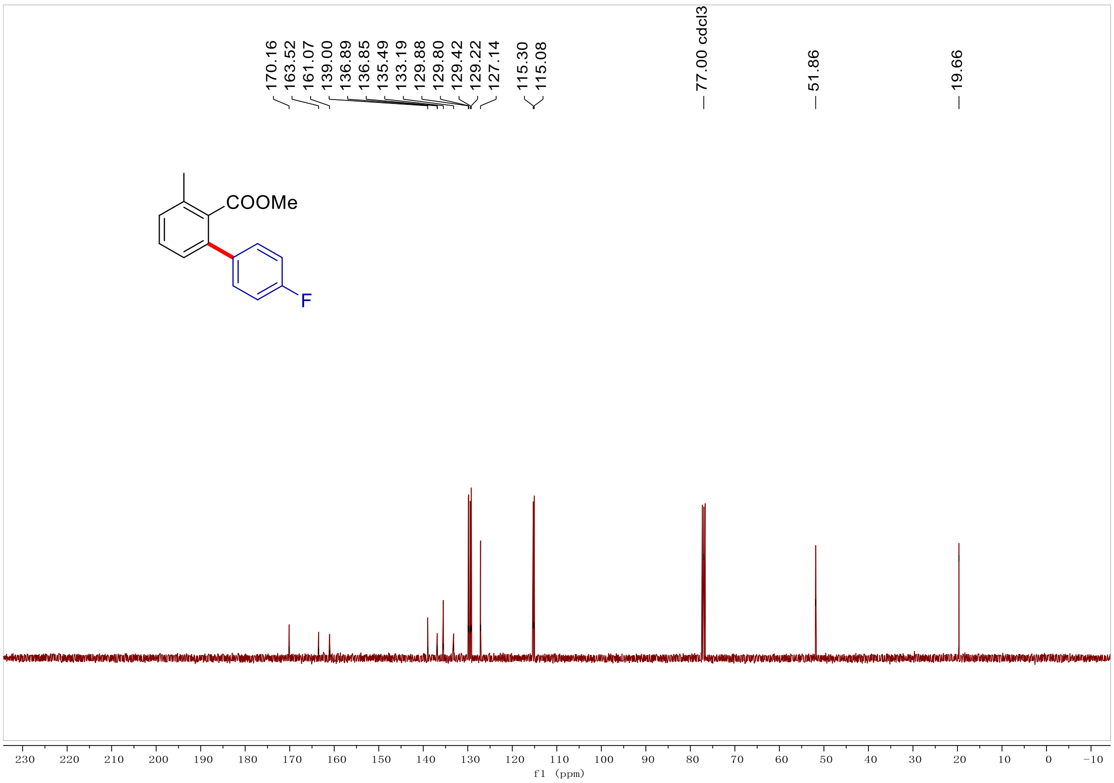

^{1}H NMR spectra (400 MHz) of **35** in $CDCl_3$.

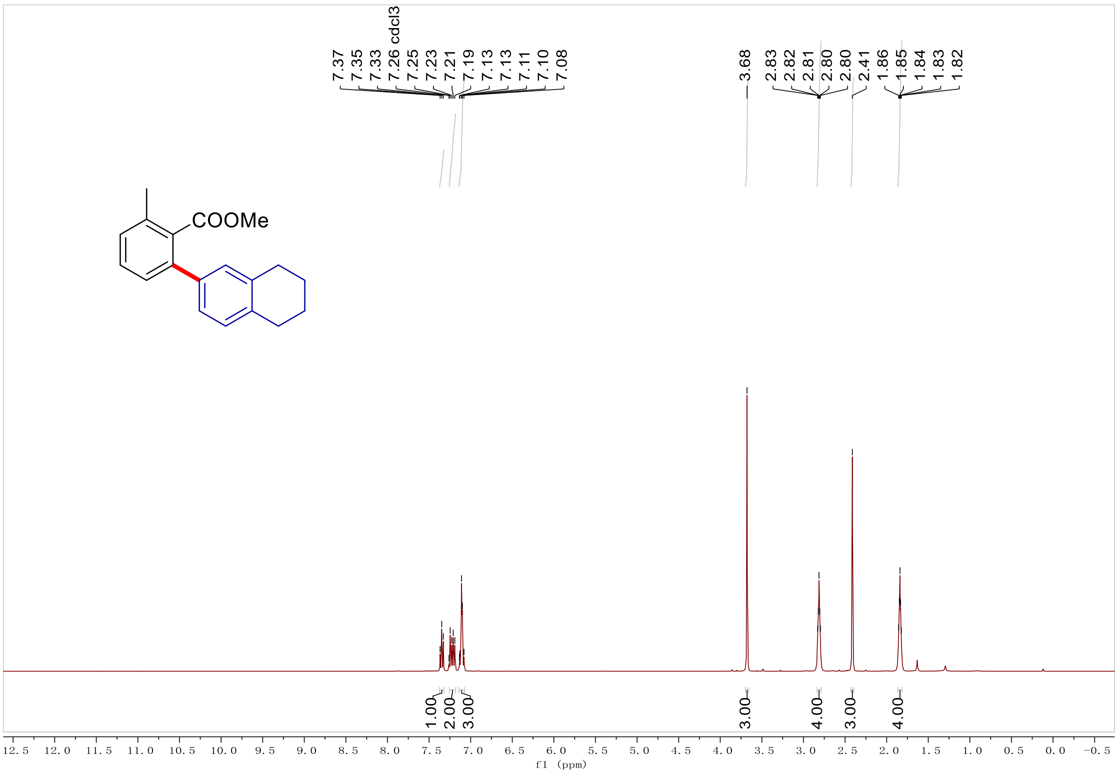

^{13}C NMR spectra (101 MHz) of **35** in $CDCl_3$.

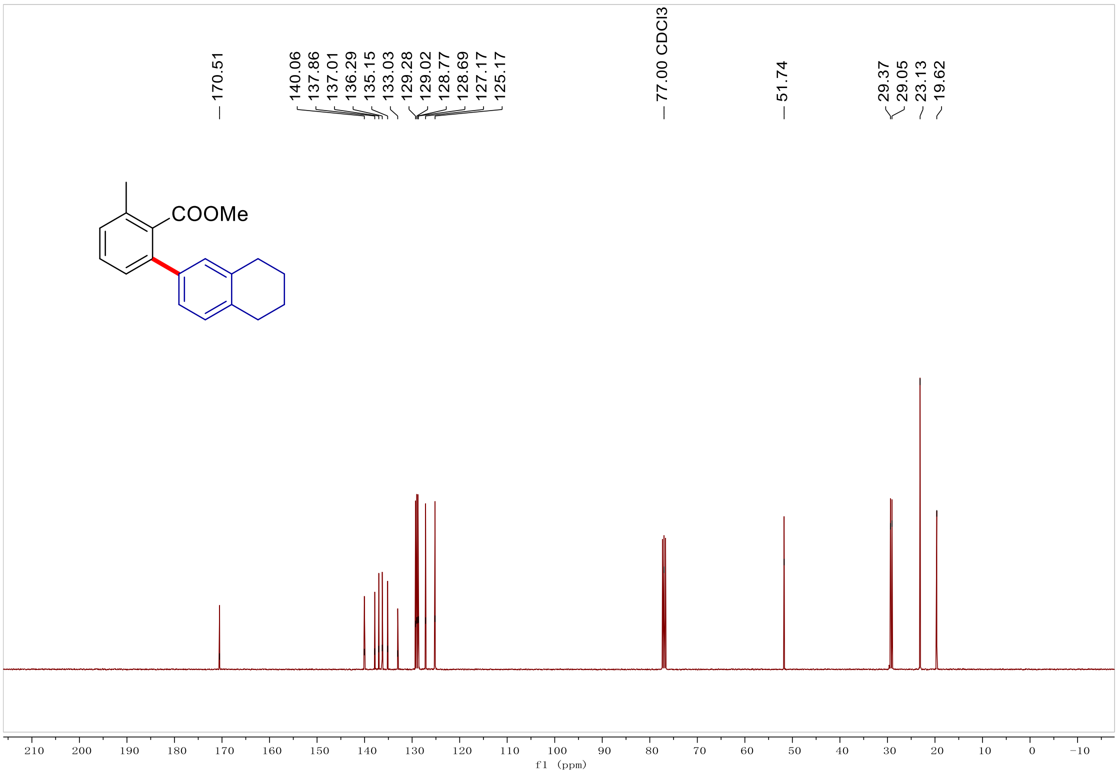

^{1}H NMR spectra (400 MHz) of **36** in $CDCl_3$.

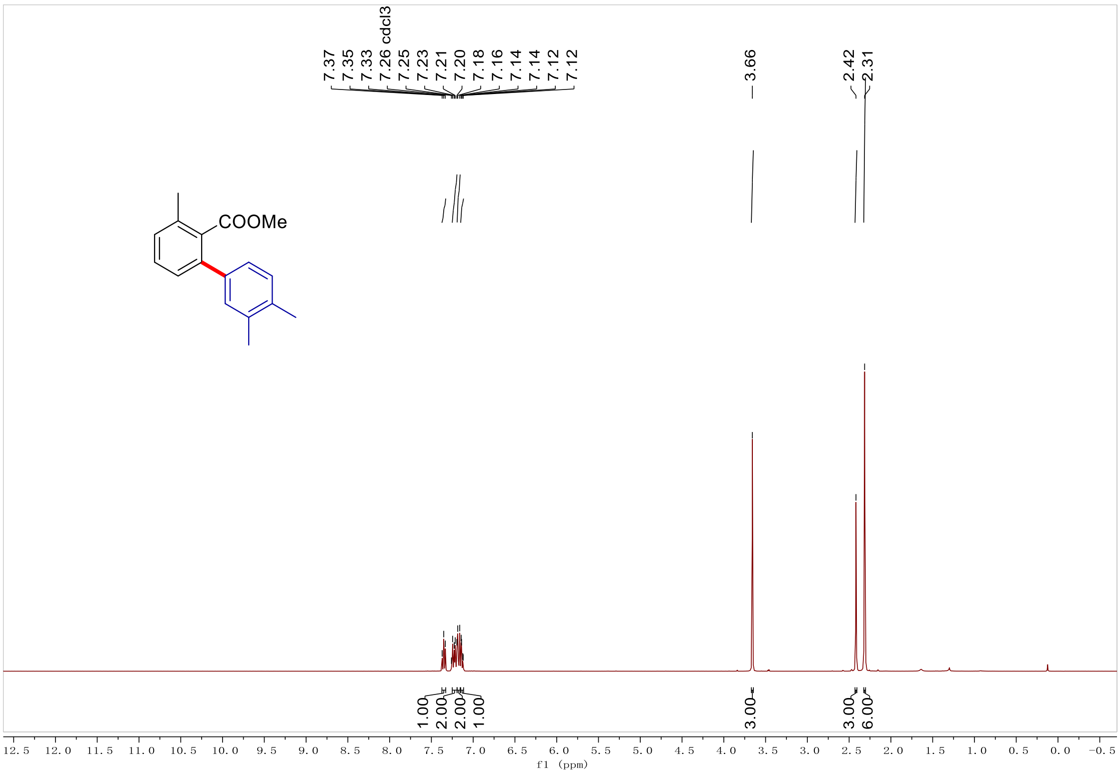

^{13}C NMR spectra (101 MHz) of **36** in $CDCl_3$.

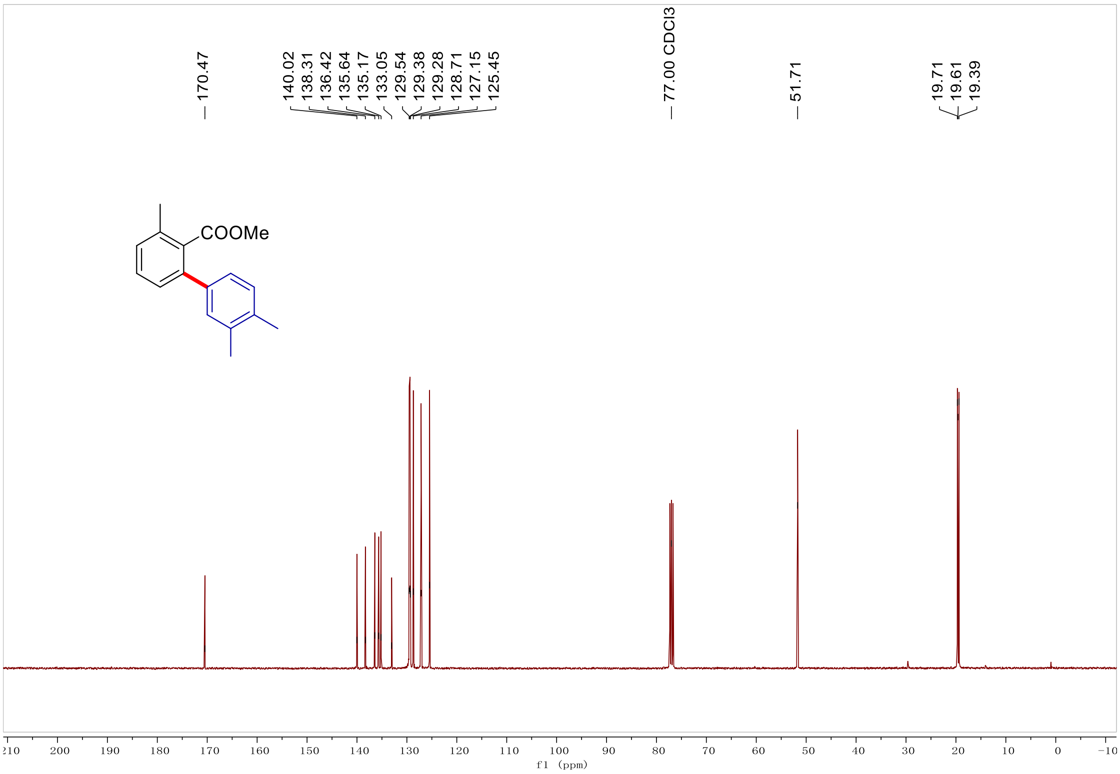

^{1}**H NMR** spectra (400 MHz) of **37** in $CDCl_3$.

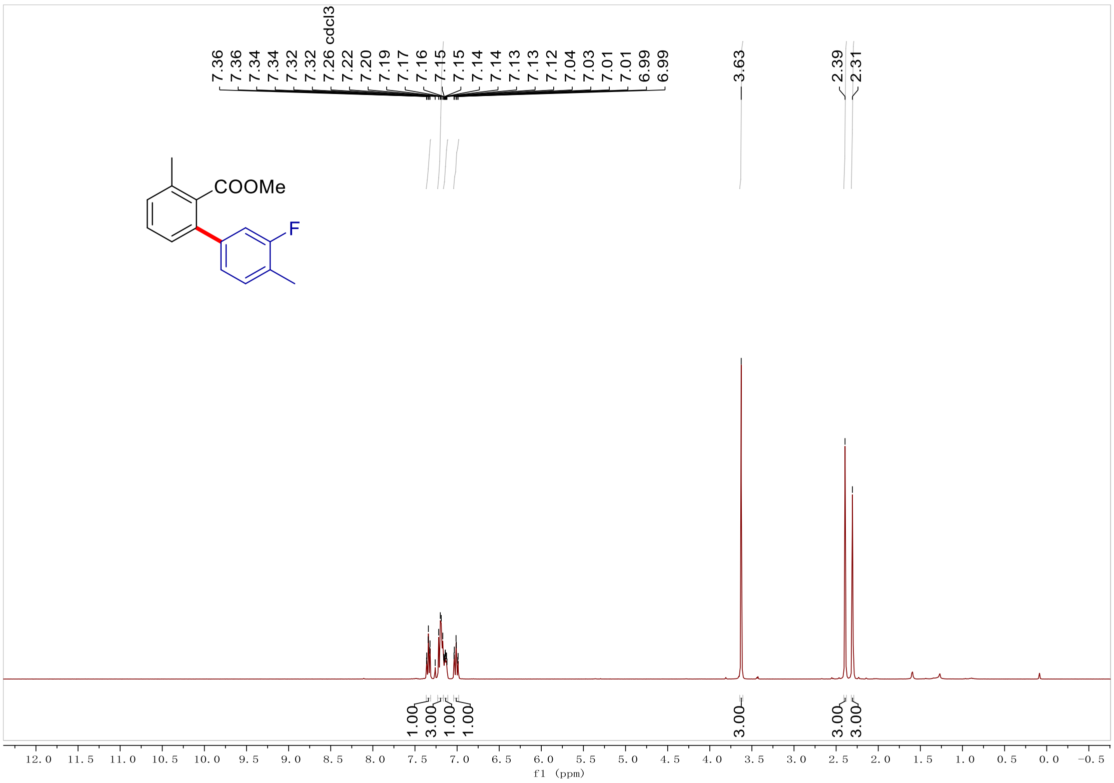

^{13}**C NMR** spectra (101 MHz) of **37** in $CDCl_3$.

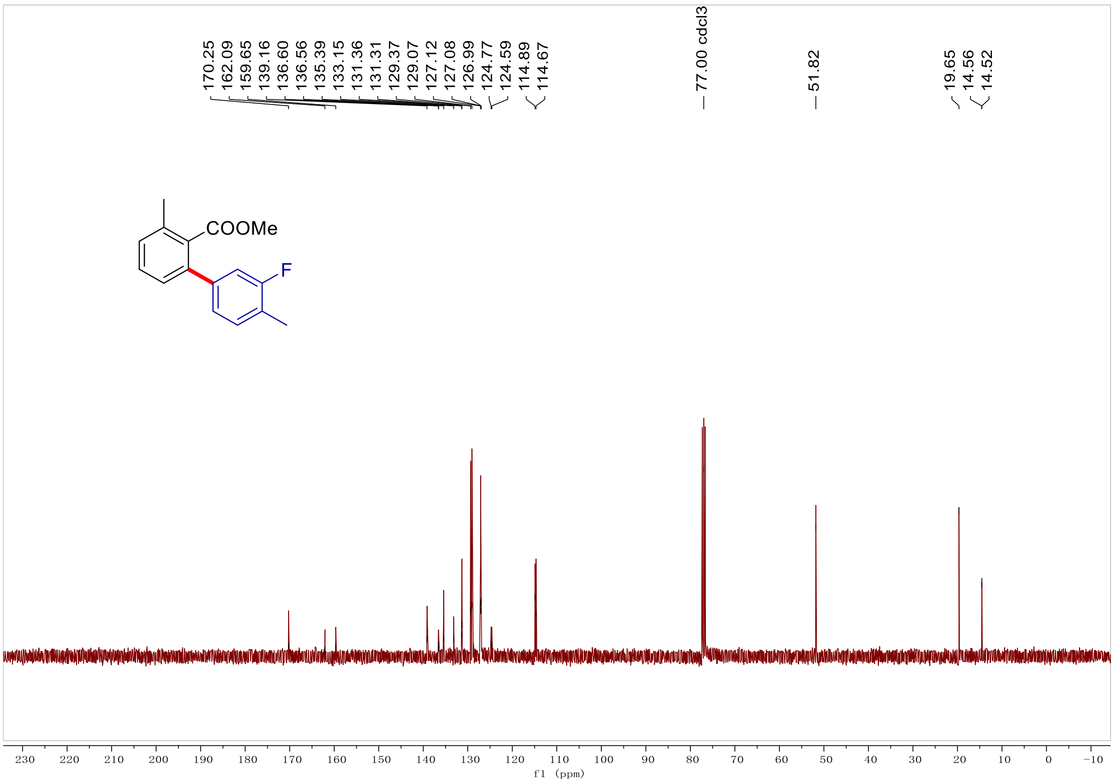

^{1}H NMR spectra (400 MHz) of **38** in $CDCl_3$.

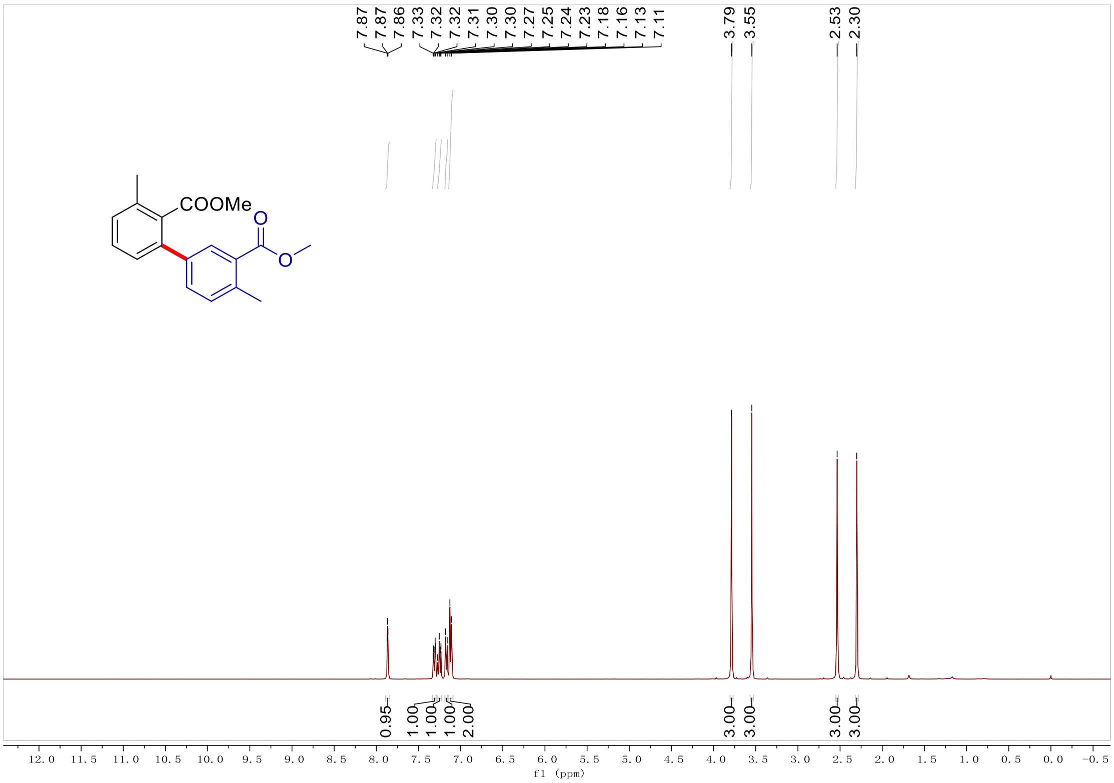

^{13}C NMR spectra (101 MHz) of **38** in $CDCl_3$.

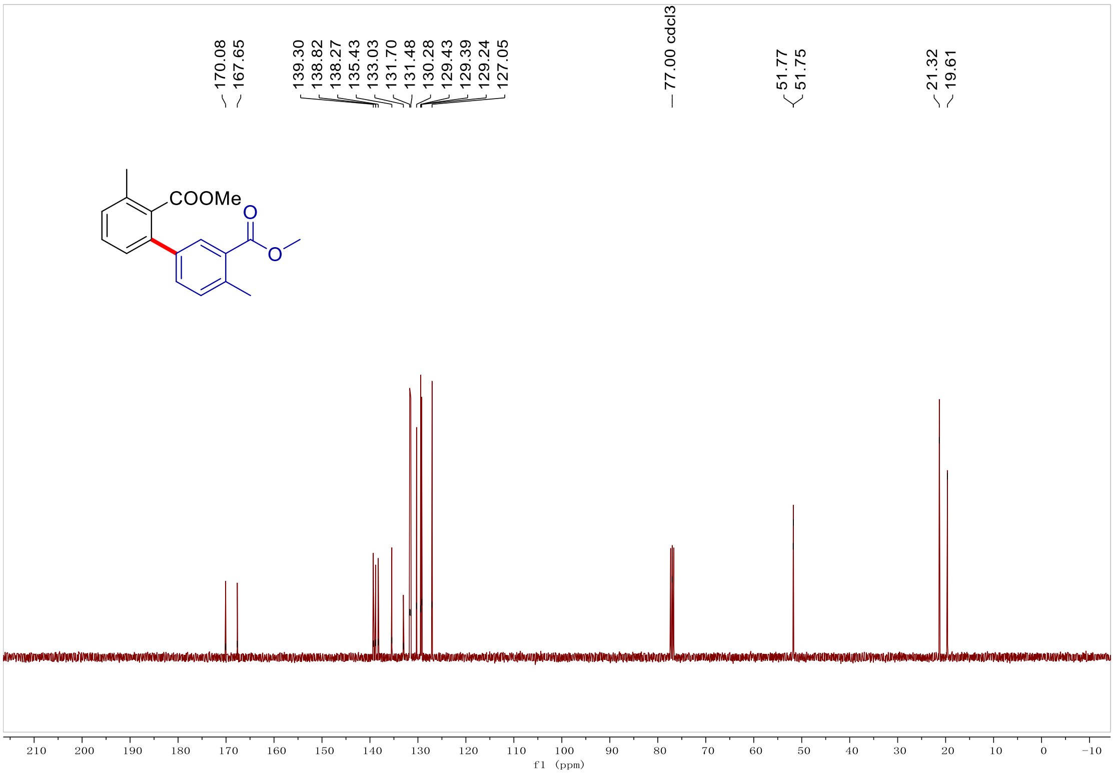

^{1}H NMR spectra (400 MHz) of **39** in $CDCl_3$.

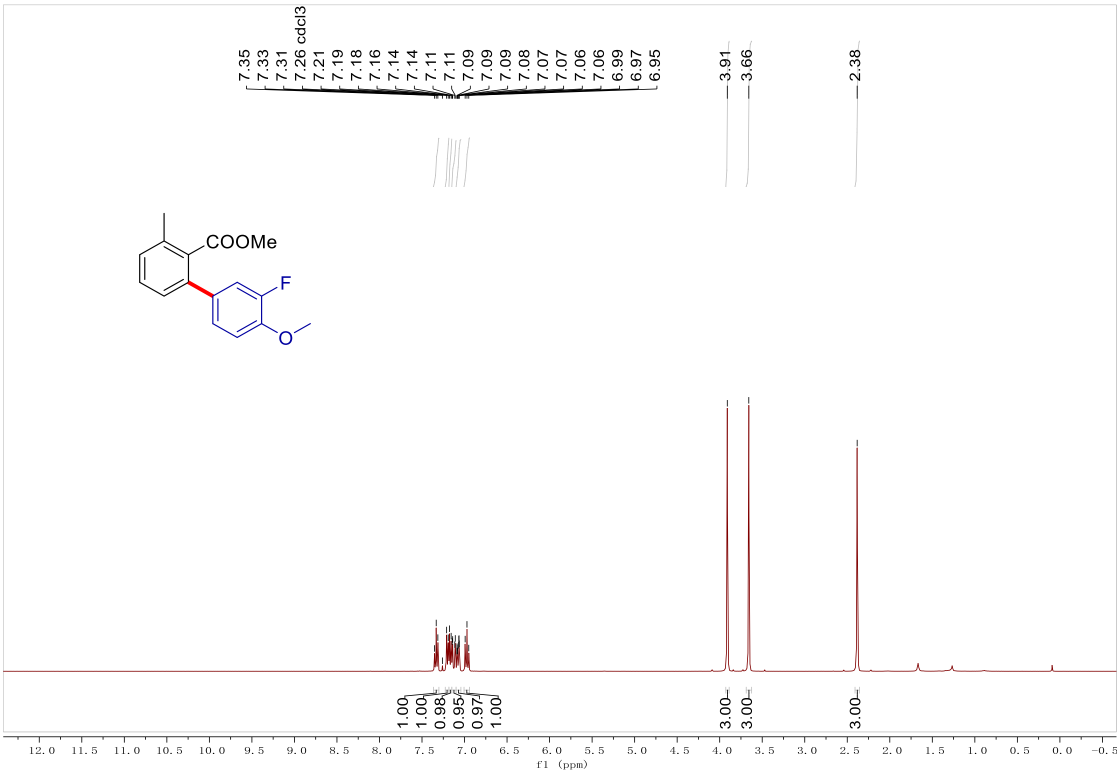

^{13}C NMR spectra (101 MHz) of **39** in $CDCl_3$.

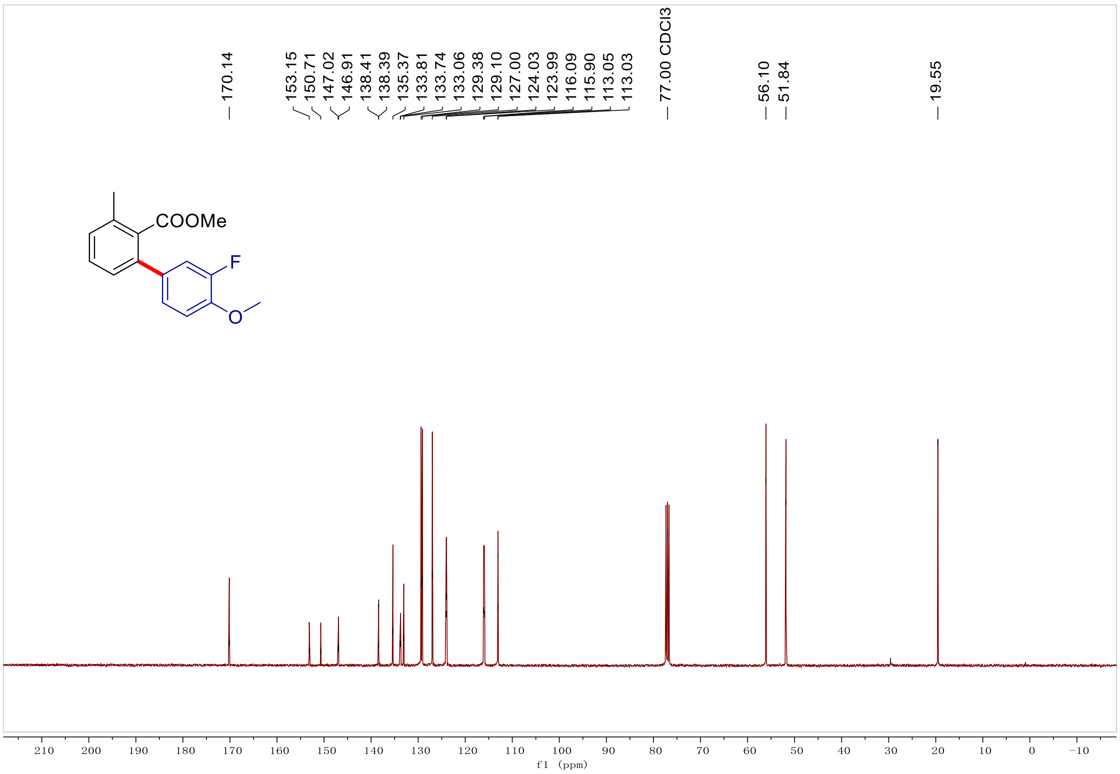

^{1}H NMR spectra (400 MHz) of **40** in $CDCl_3$.

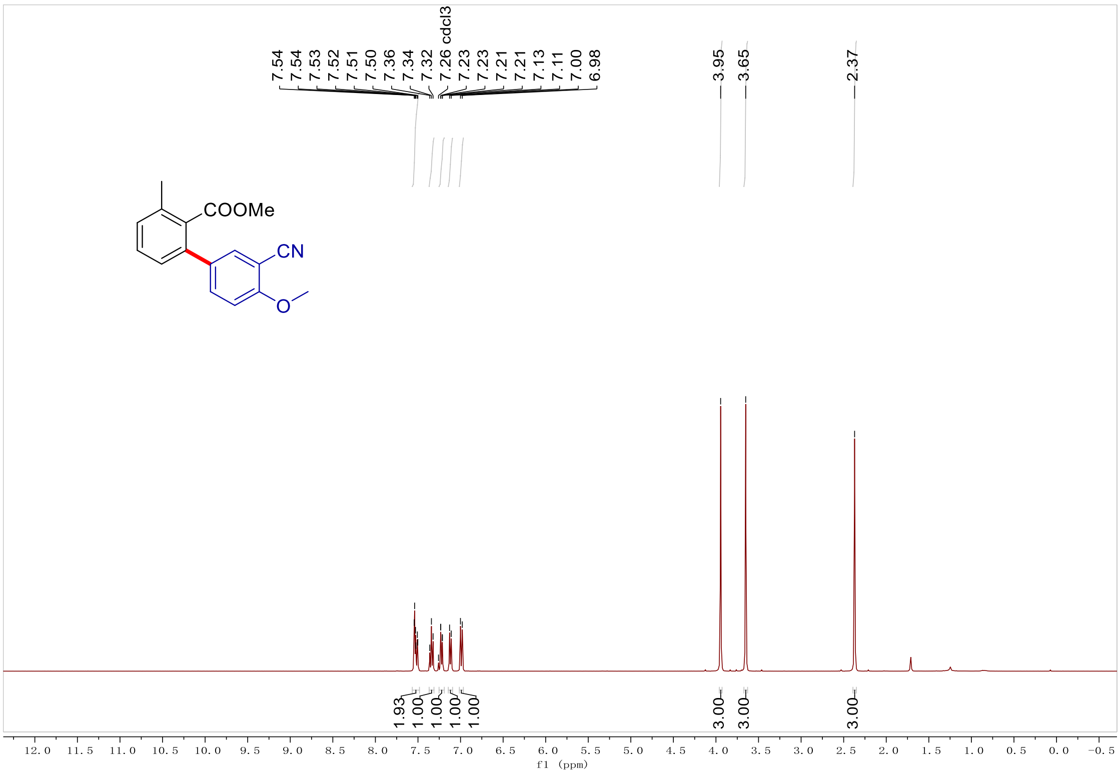

^{13}C NMR spectra (101 MHz) of **40** in $CDCl_3$.

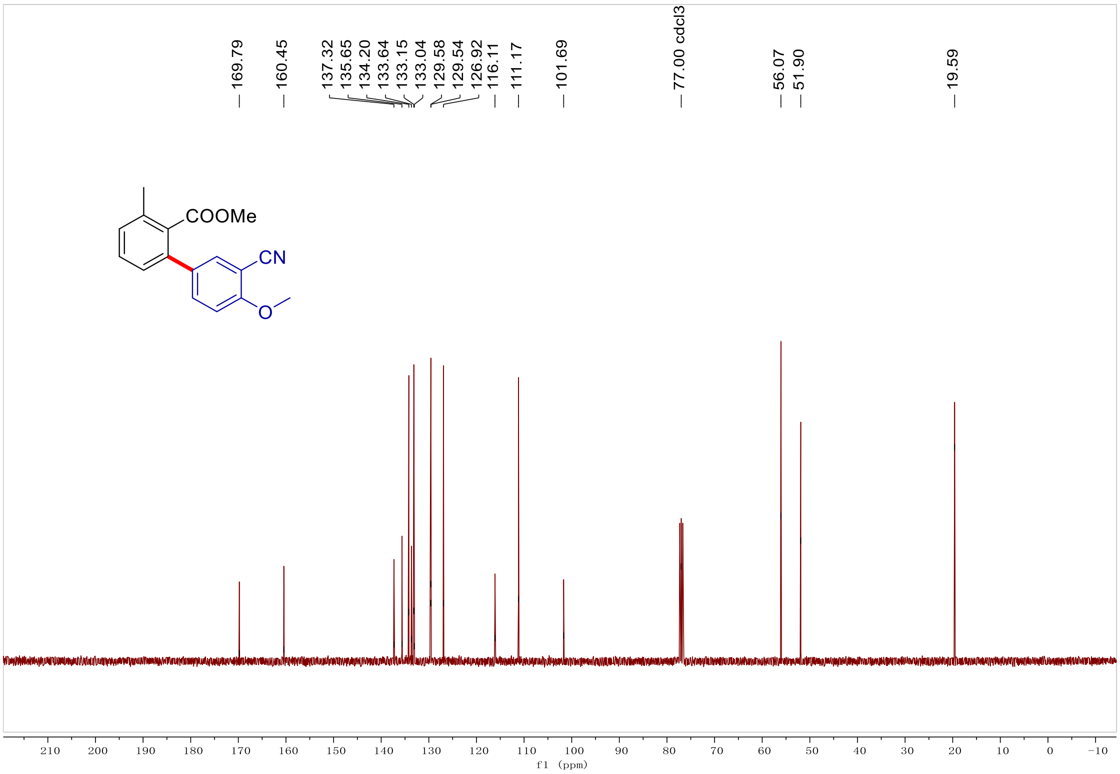

^{1}H NMR spectra (400 MHz) of **41** in CDCl3.

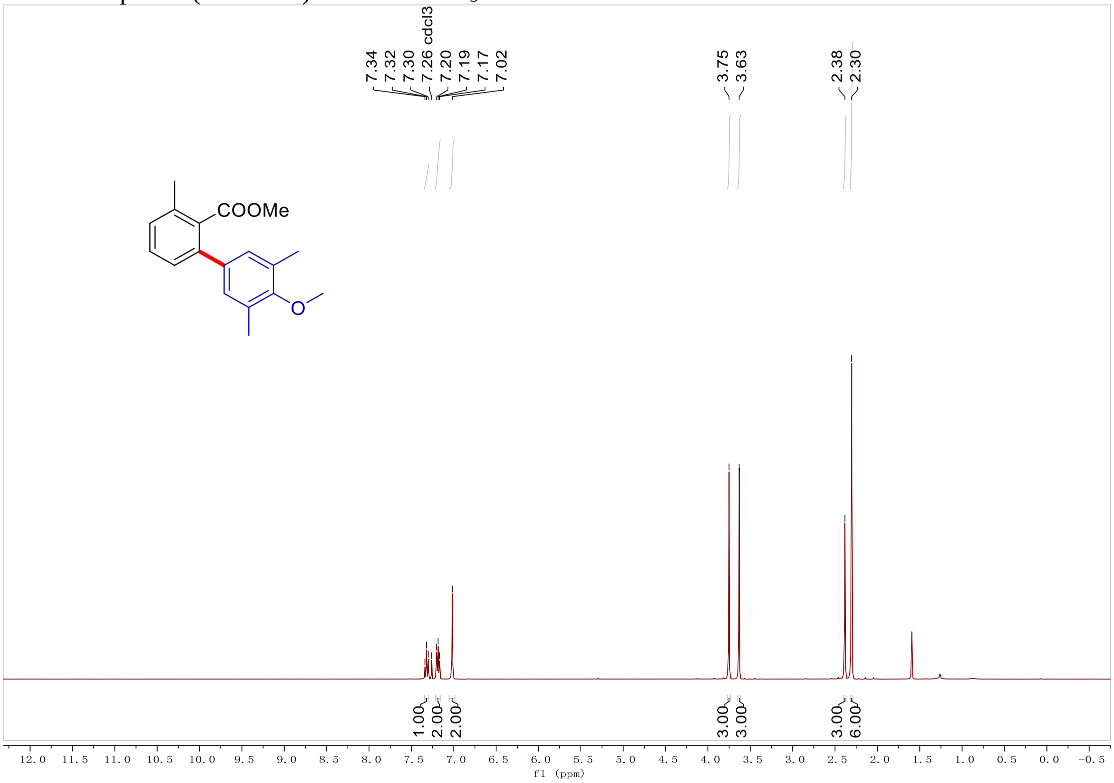

^{13}C NMR spectra (101 MHz) of **41** in CDCl3.

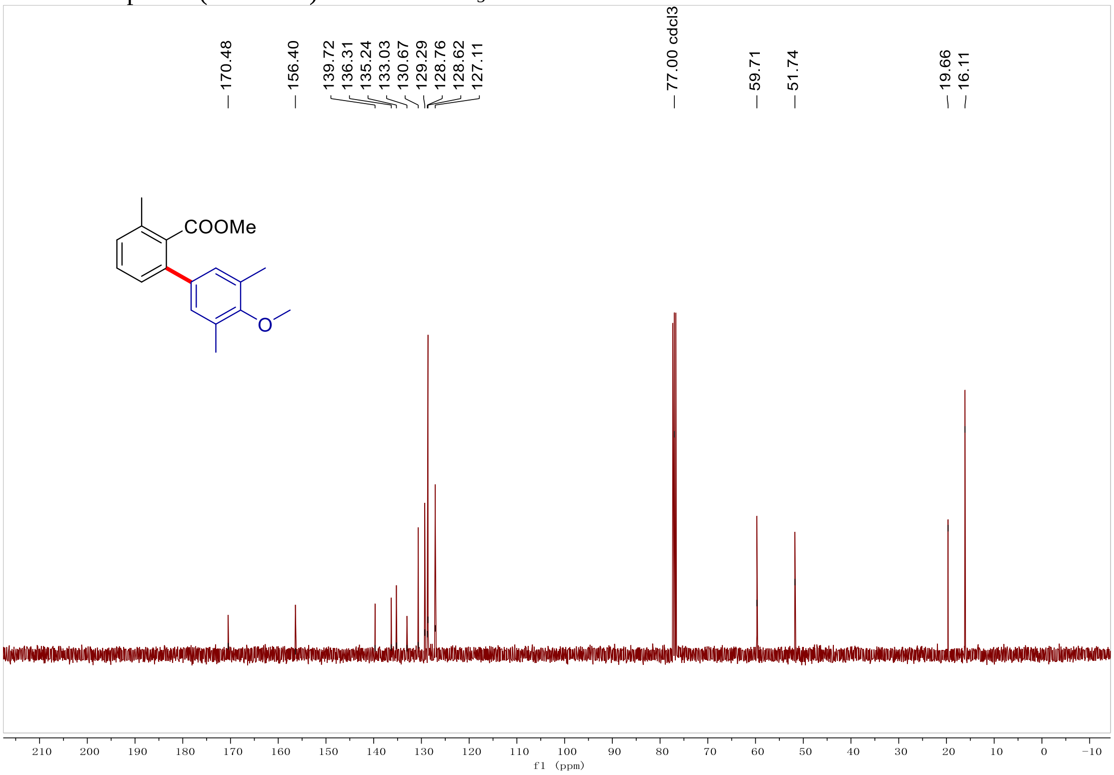

^{1}H NMR spectra (400 MHz) of **42** in $CDCl_3$.

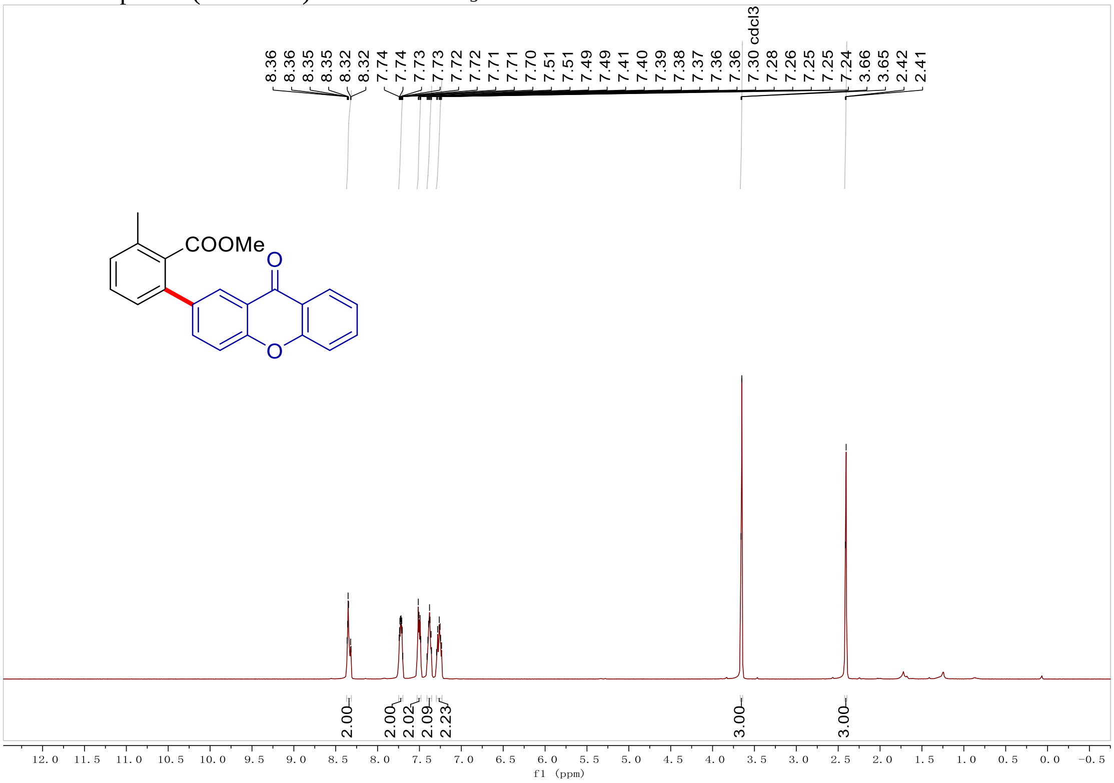

^{13}C NMR spectra (101 MHz) of **42** in $CDCl_3$.

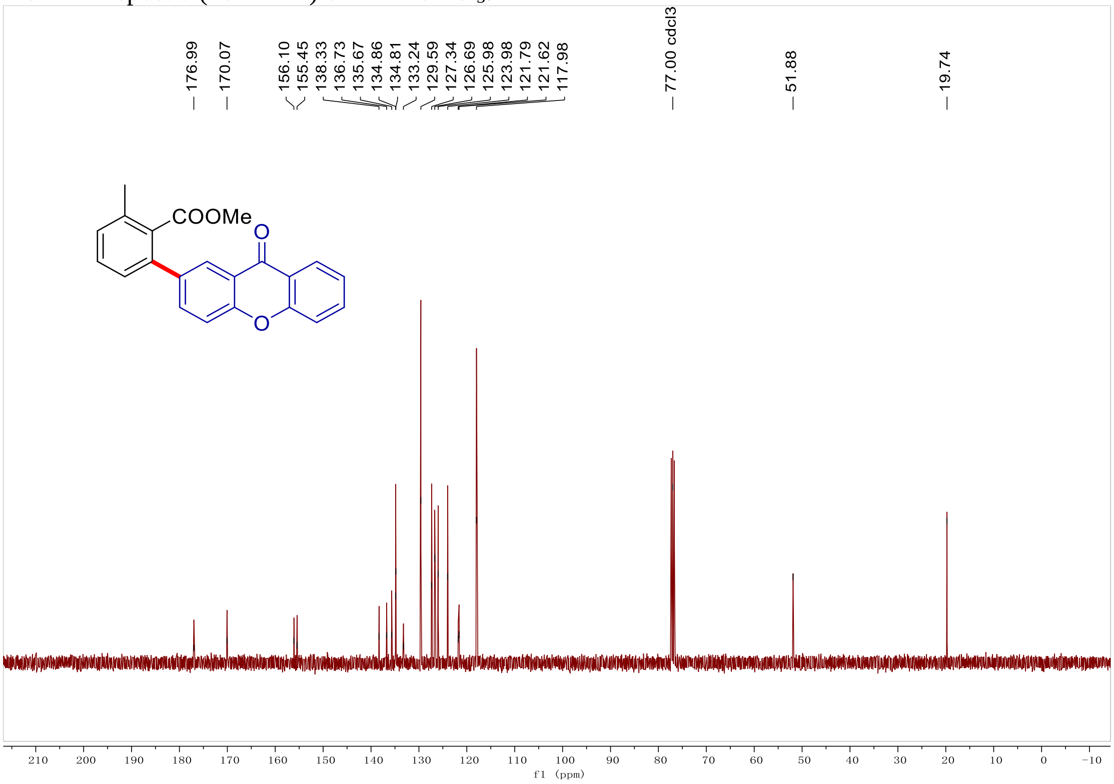

^{1}H NMR spectra (400 MHz) of **43** in $CDCl_3$.

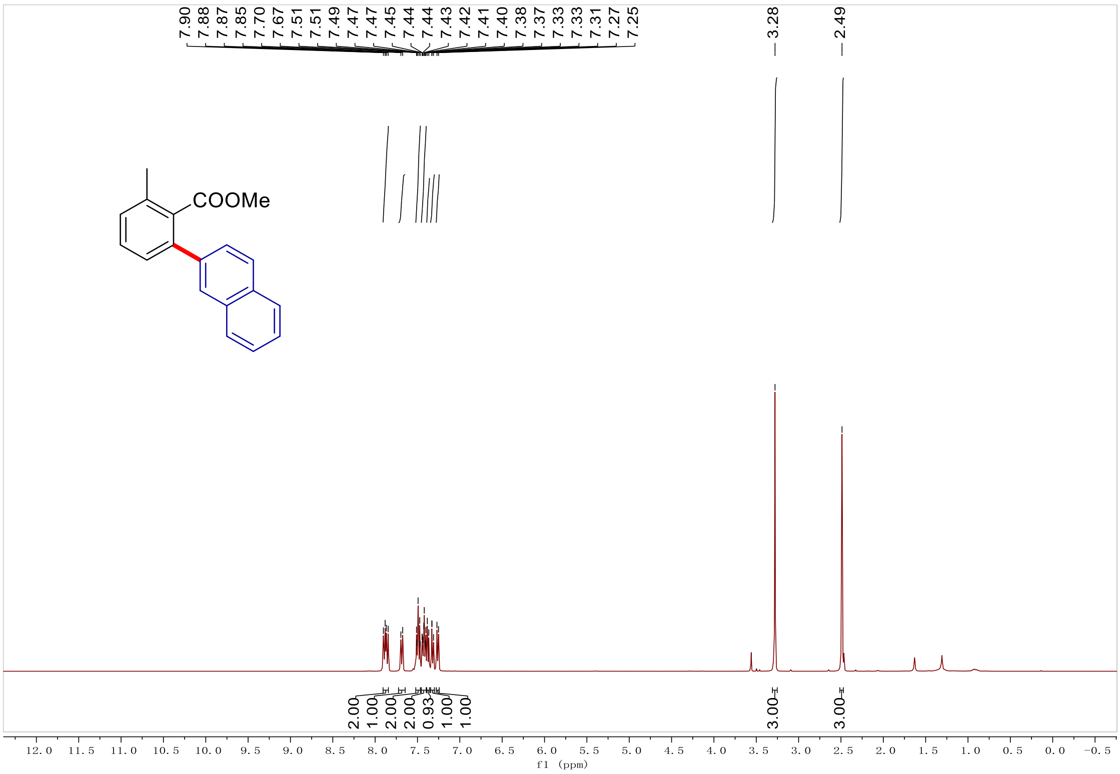

^{13}C NMR spectra (101 MHz) of **43** in $CDCl_3$.

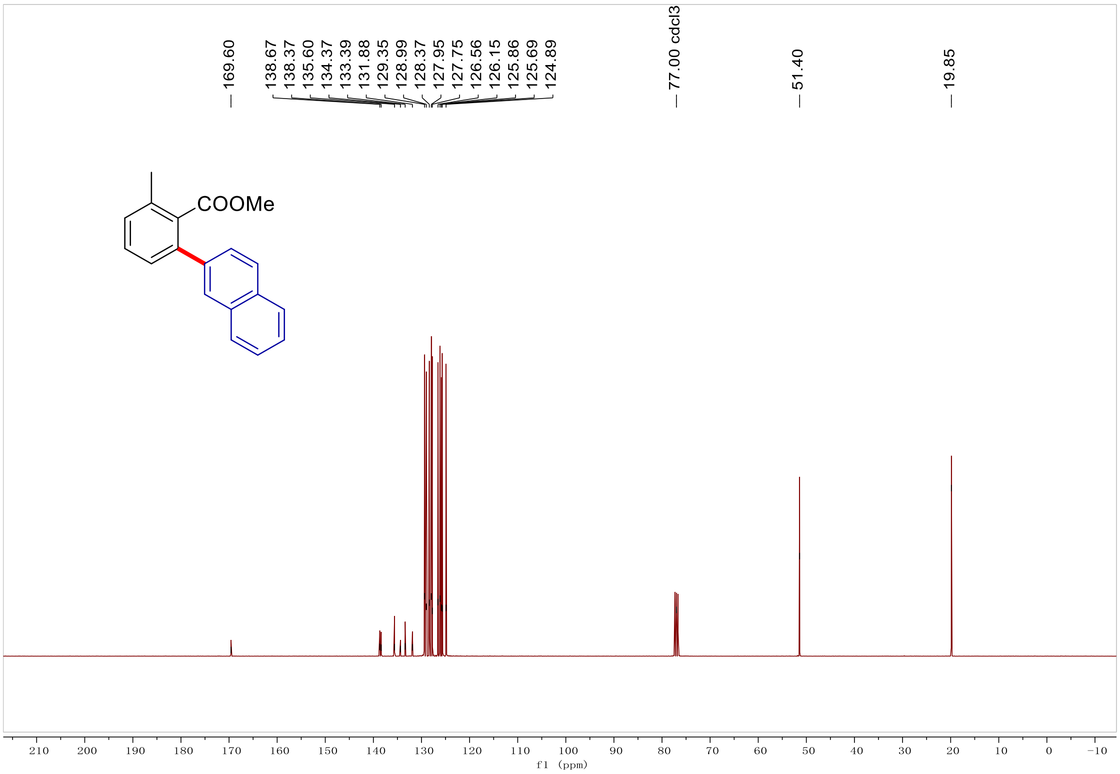

1H NMR spectra (400 MHz) of **44** in $CDCl_3$.

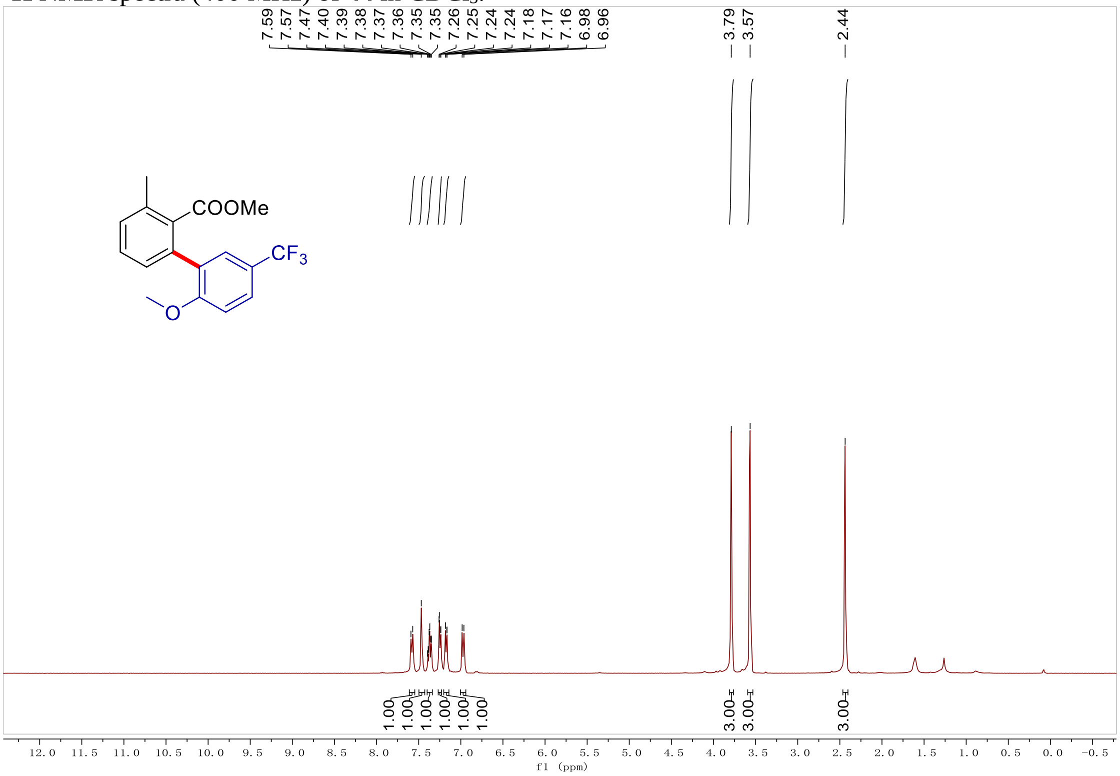

13C NMR spectra (101 MHz) of **44** in $CDCl_3$.

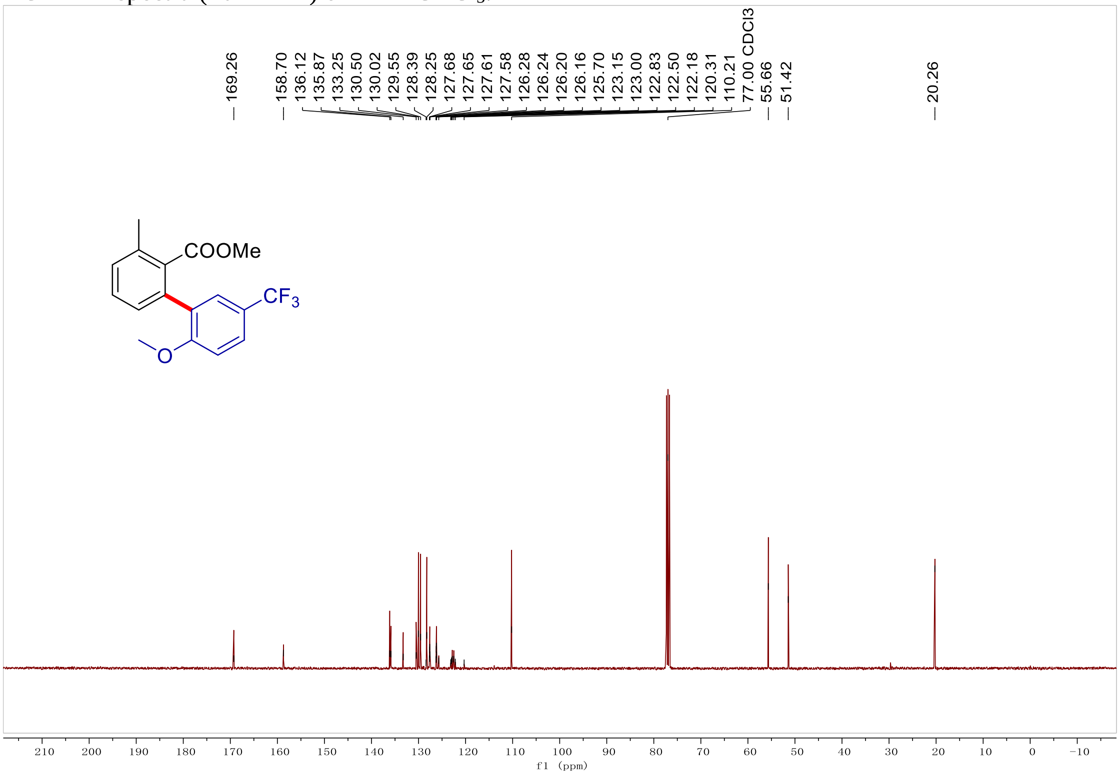

^{1}**H NMR** spectra (400 MHz) of **45** in CDCl$_3$.

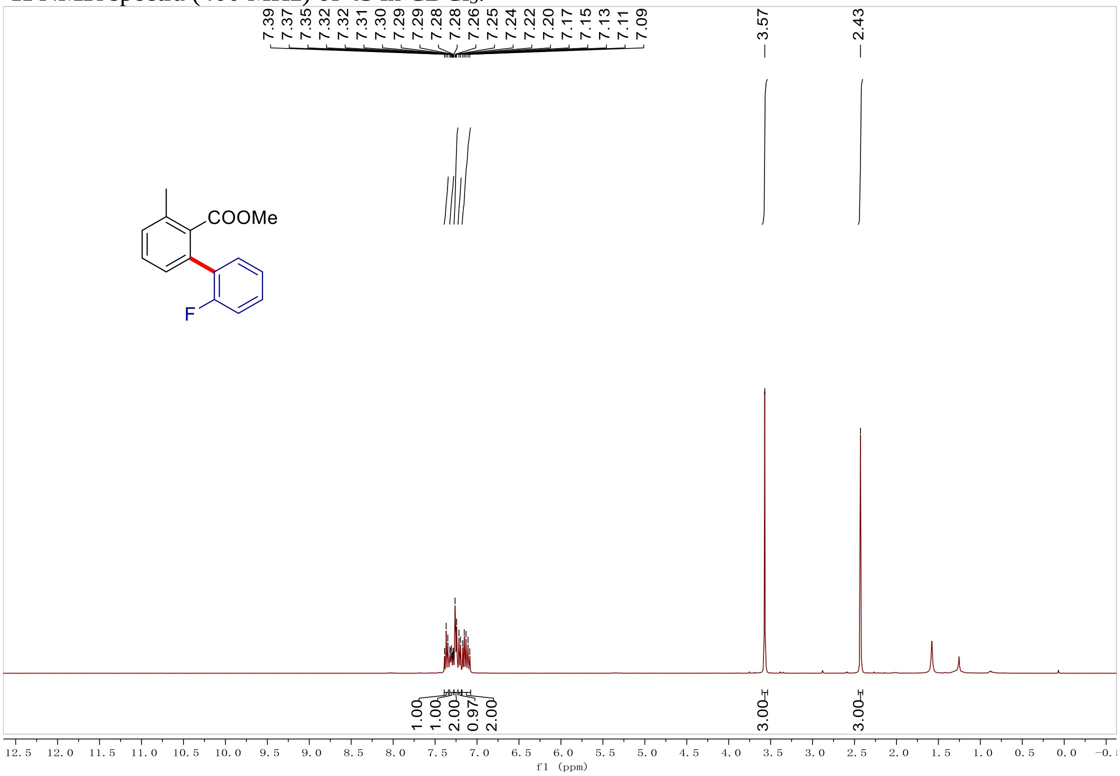

^{13}**C NMR** spectra (101 MHz) of **45** in CDCl$_3$.

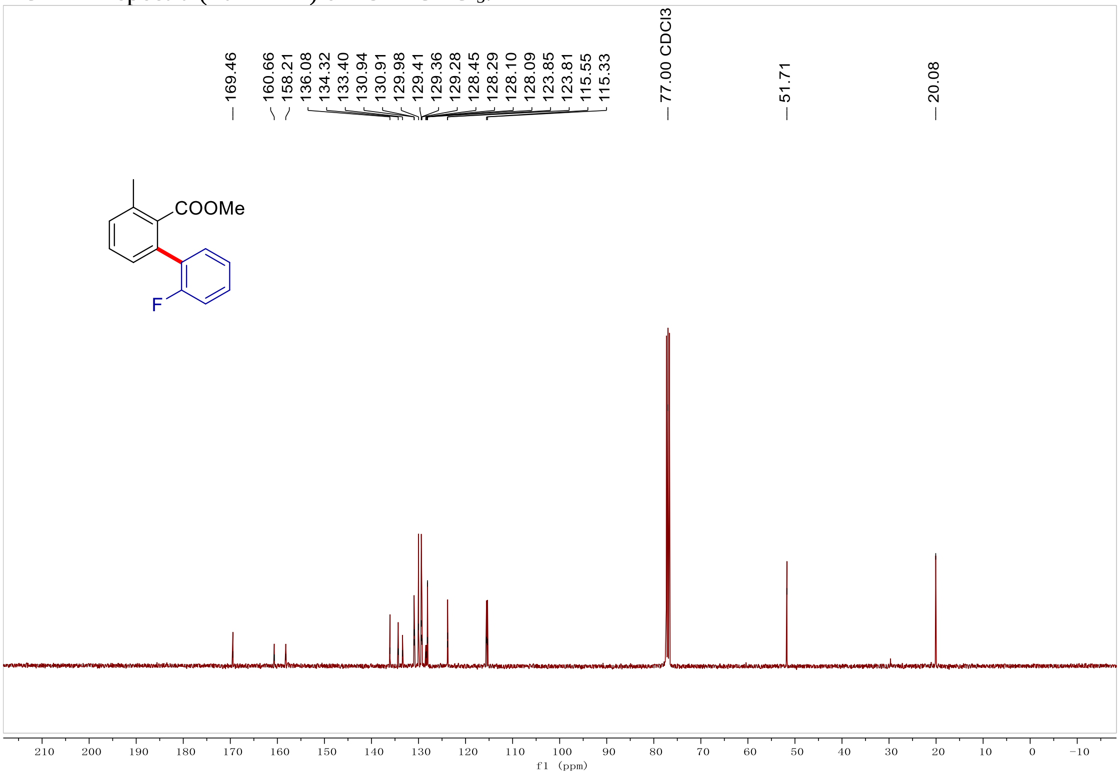

^{1}H NMR spectra (400 MHz) of **46** in $CDCl_3$.

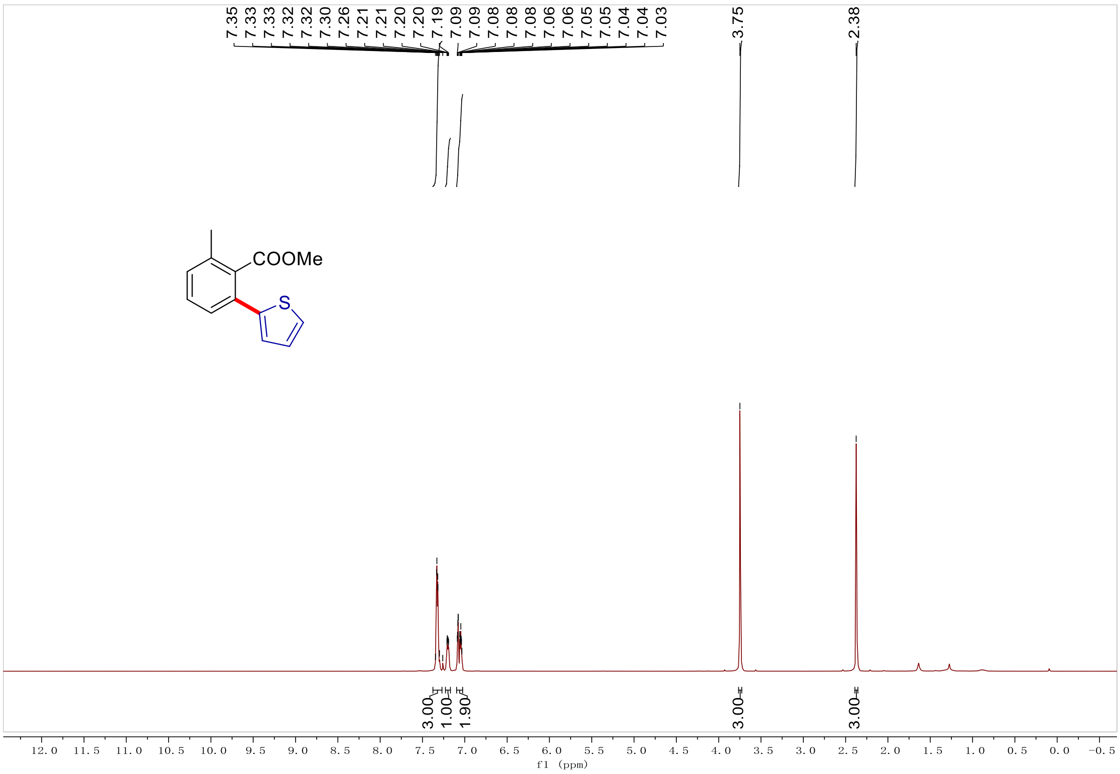

^{13}C NMR spectra (101 MHz) of **46** in $CDCl_3$.

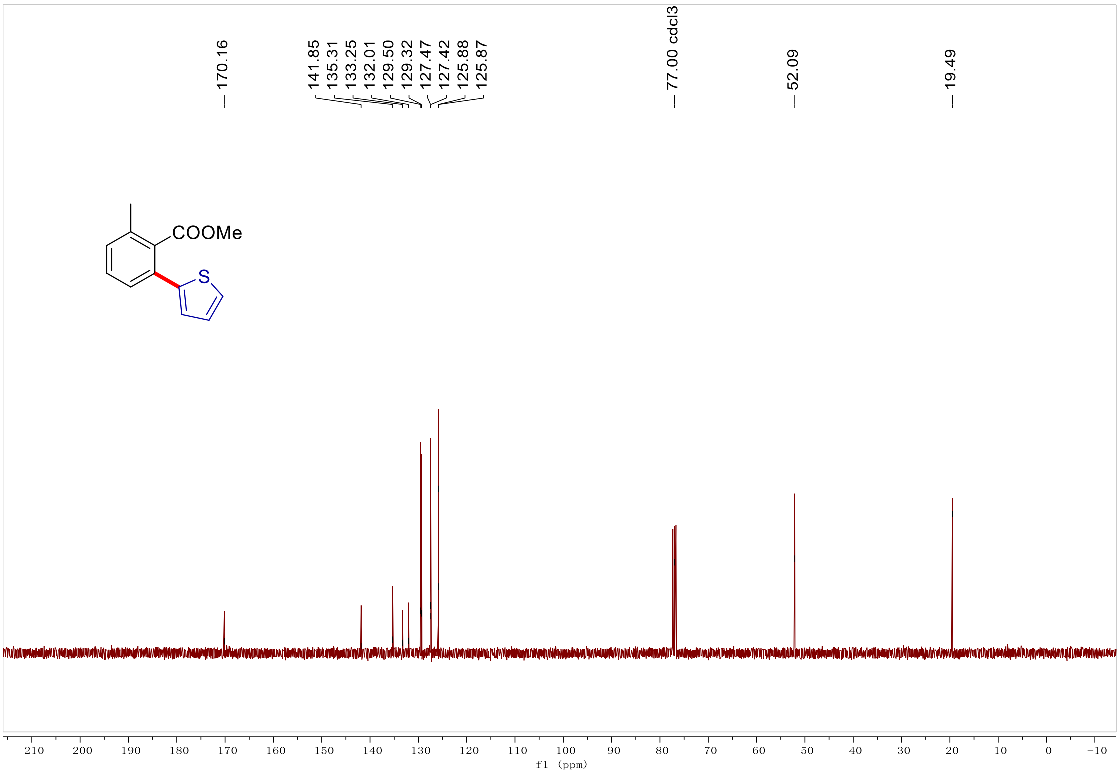

^{1}H NMR spectra (400 MHz) of **47** in $CDCl_3$.

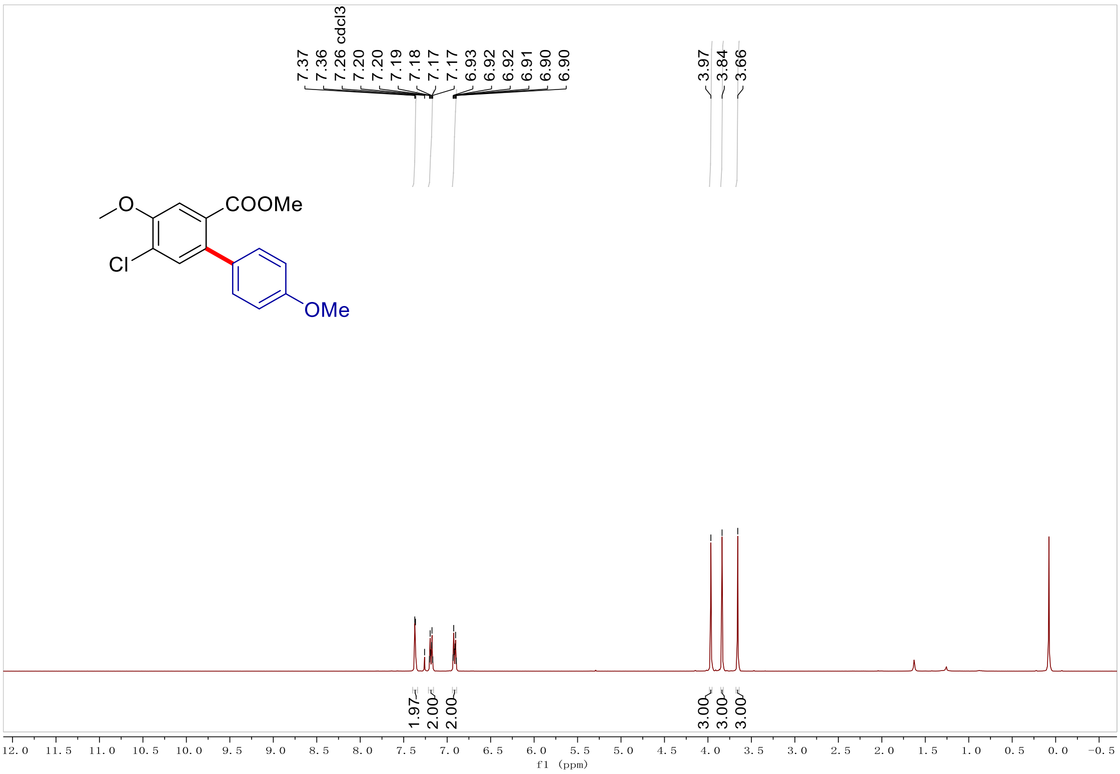

^{13}C NMR spectra (101 MHz) of **47** in $CDCl_3$.

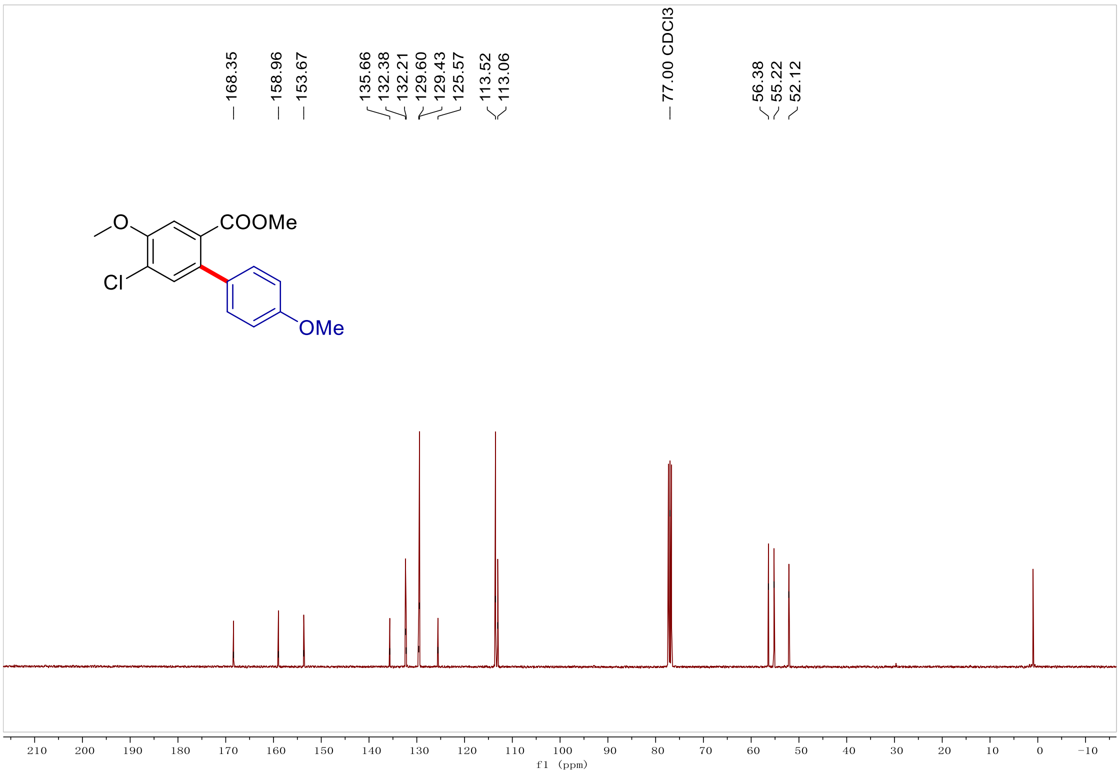

^{1}H NMR spectra (400 MHz) of **48** in $CDCl_3$.

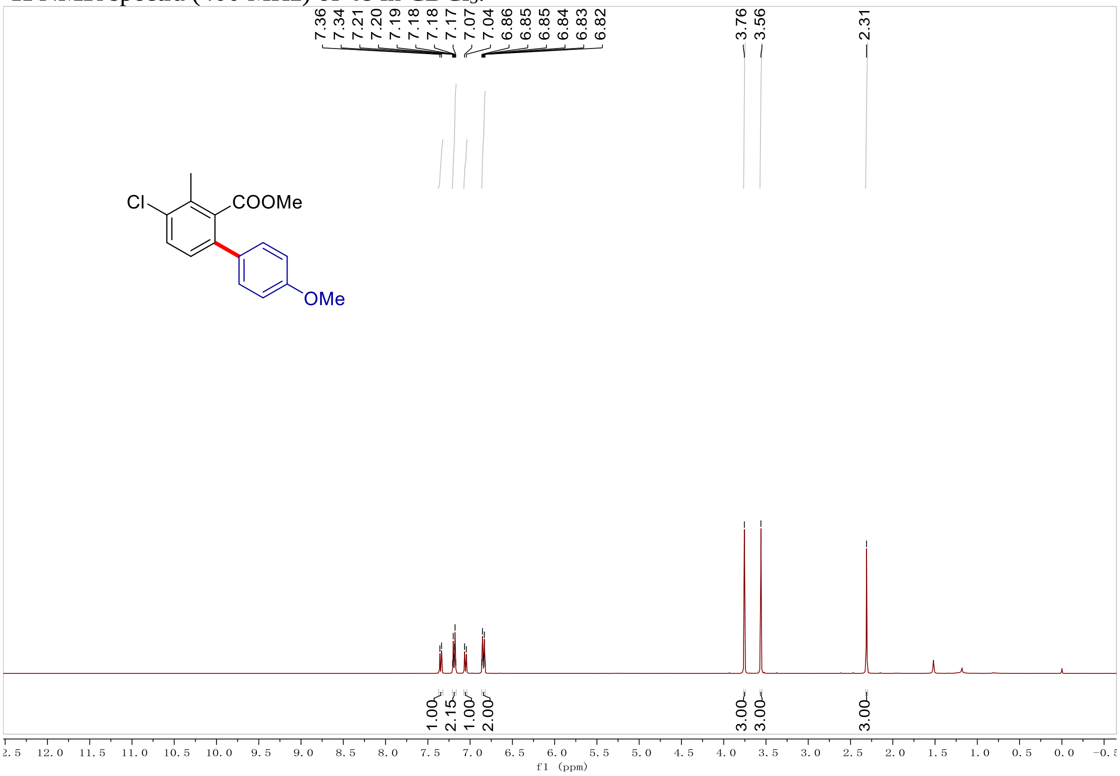

^{13}C NMR spectra (101 MHz) of **48** in $CDCl_3$.

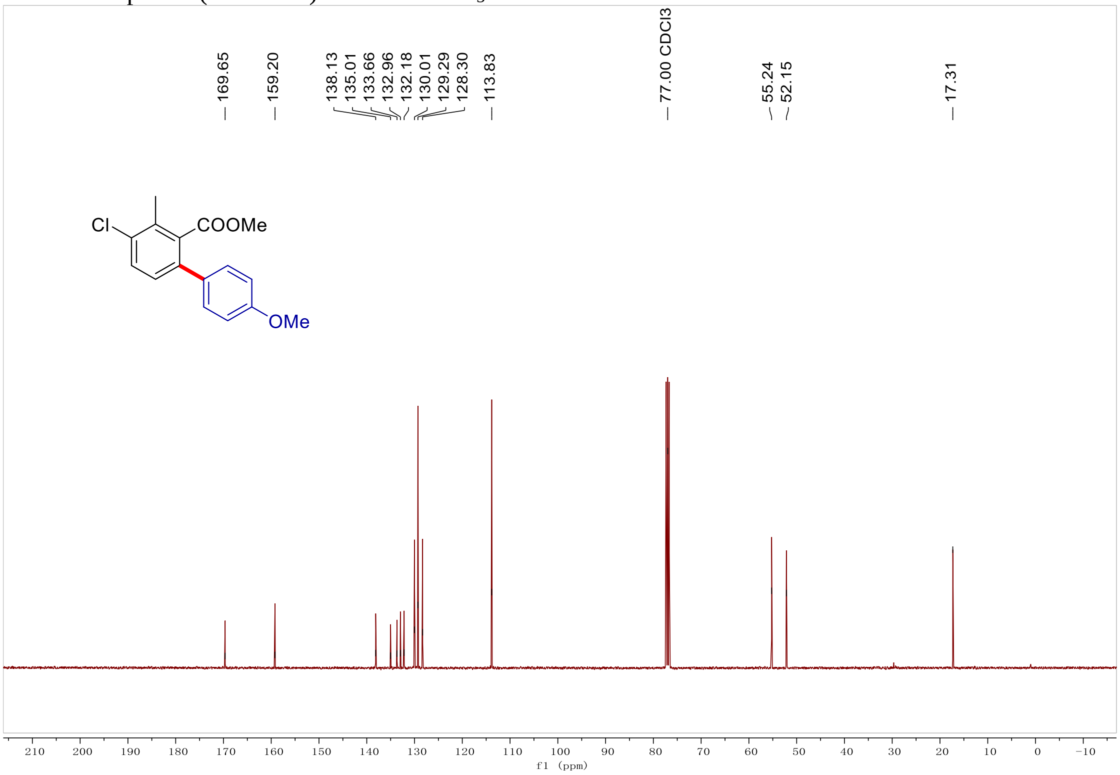

[1]**H NMR** spectra (400 MHz) of **49** in $CDCl_3$.

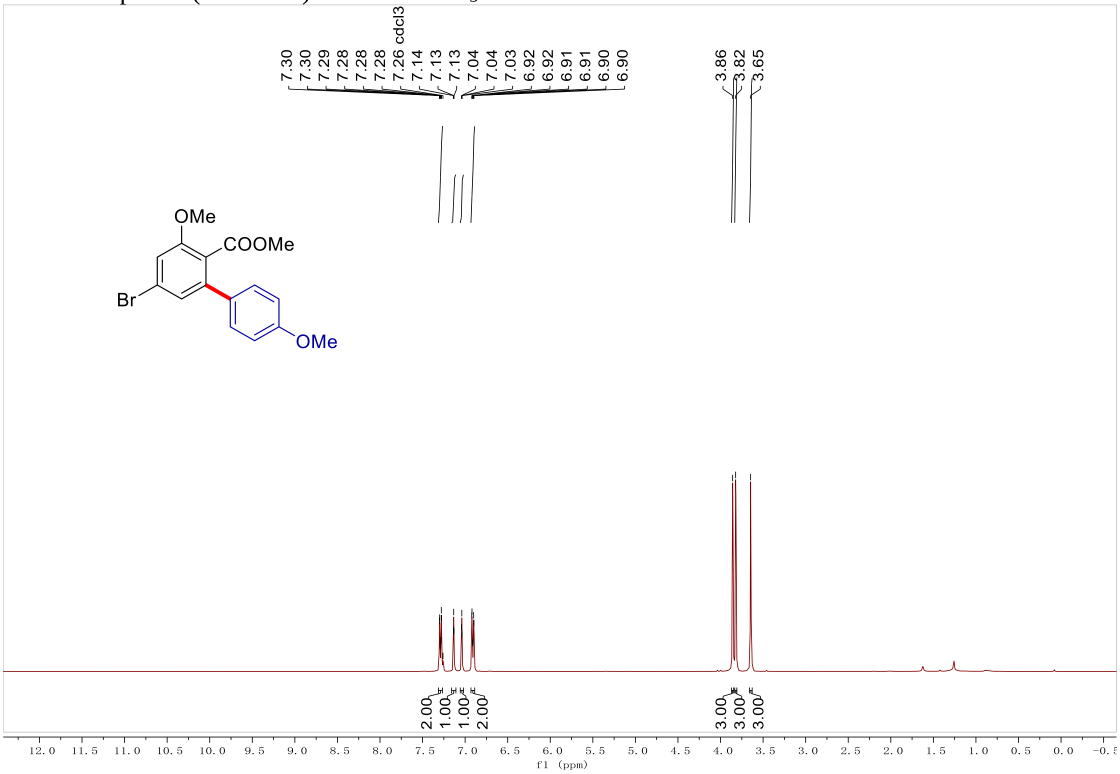

[13]**C NMR** spectra (101 MHz) of **49** in $CDCl_3$.

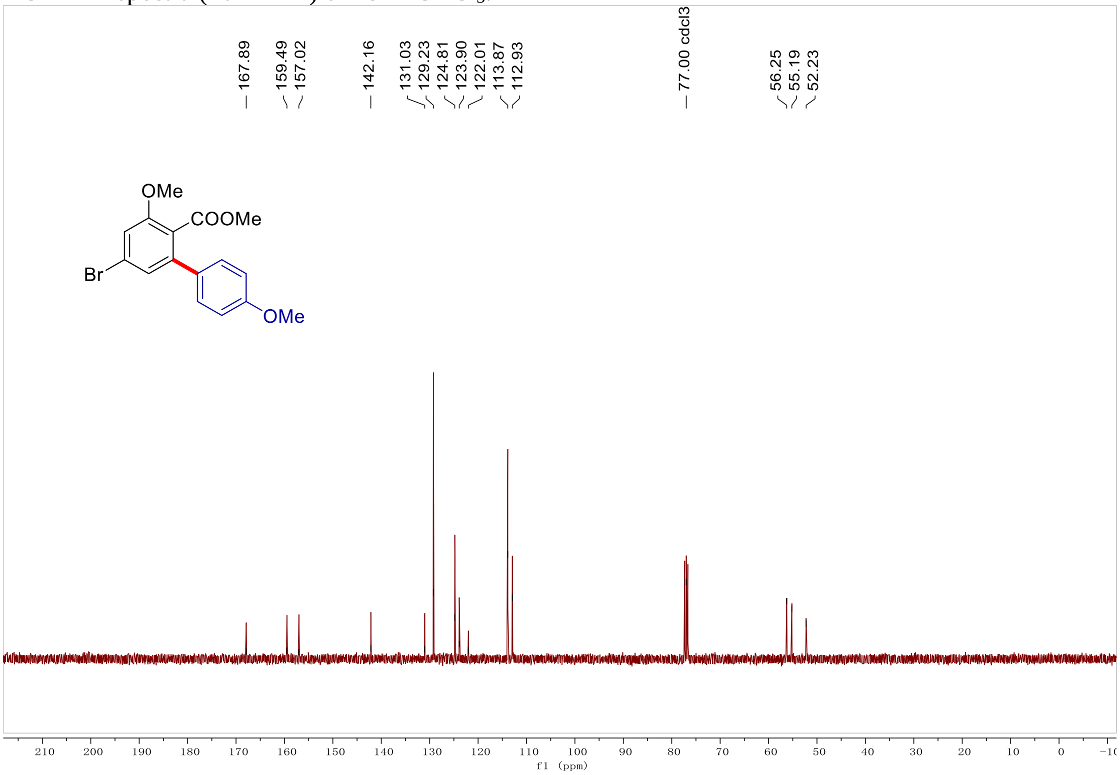

^{1}H NMR spectra (400 MHz) of **50** in $CDCl_3$.

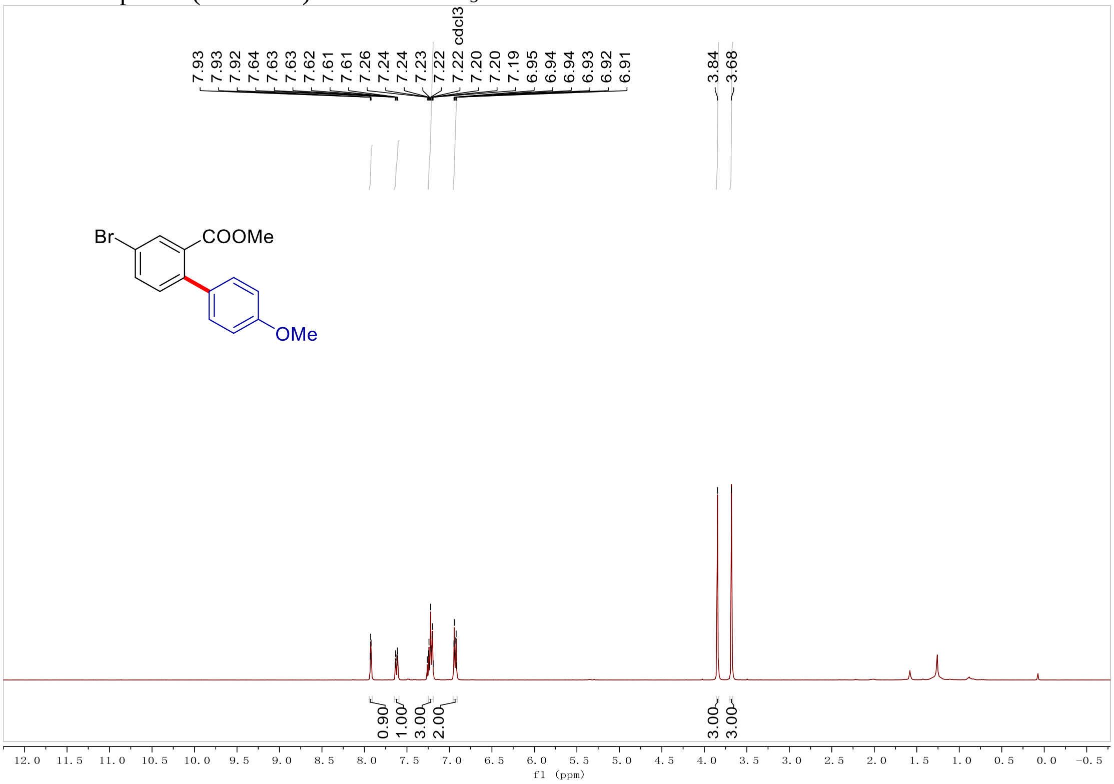

^{13}C NMR spectra (101 MHz) of **50** in $CDCl_3$.

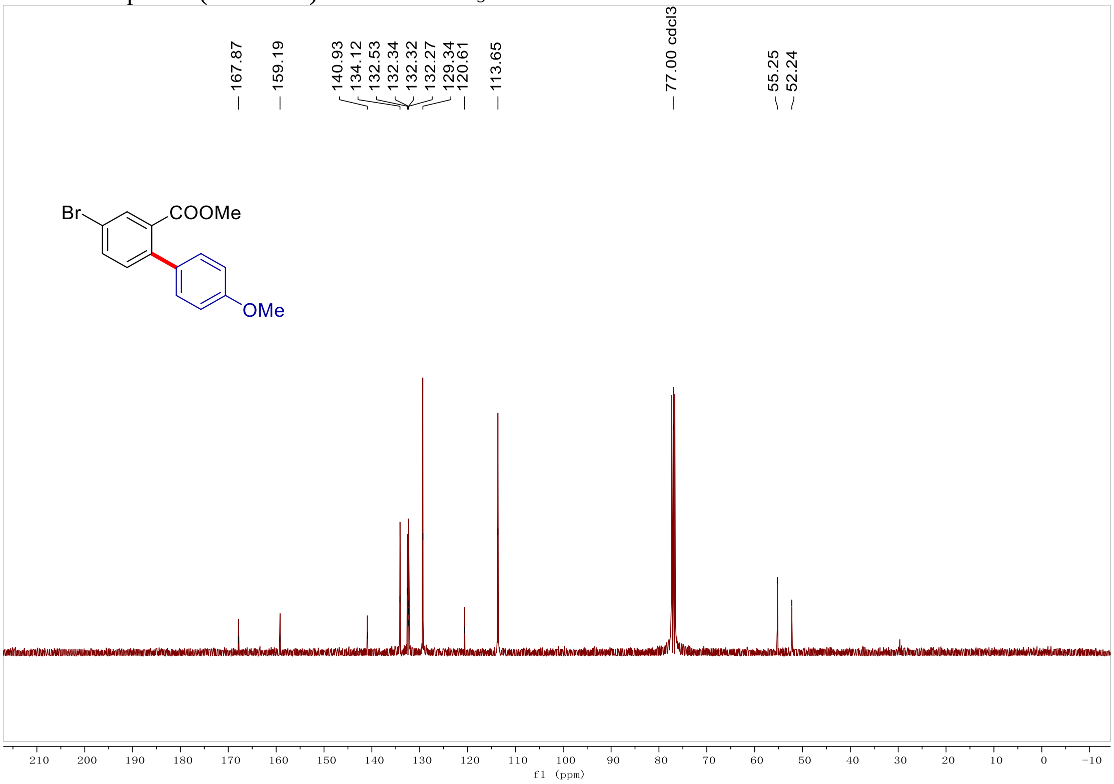

^{1}H NMR spectra (400 MHz) of **51** in $CDCl_3$.

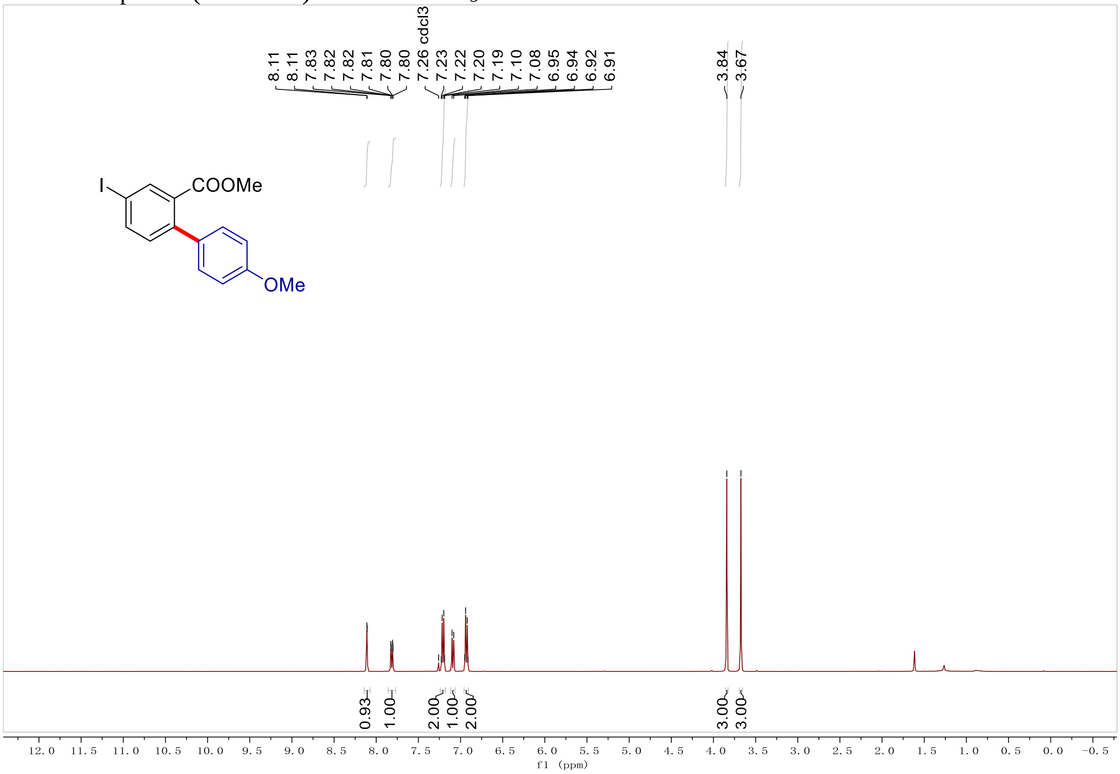

^{13}C NMR spectra (101 MHz) of **51** in $CDCl_3$.

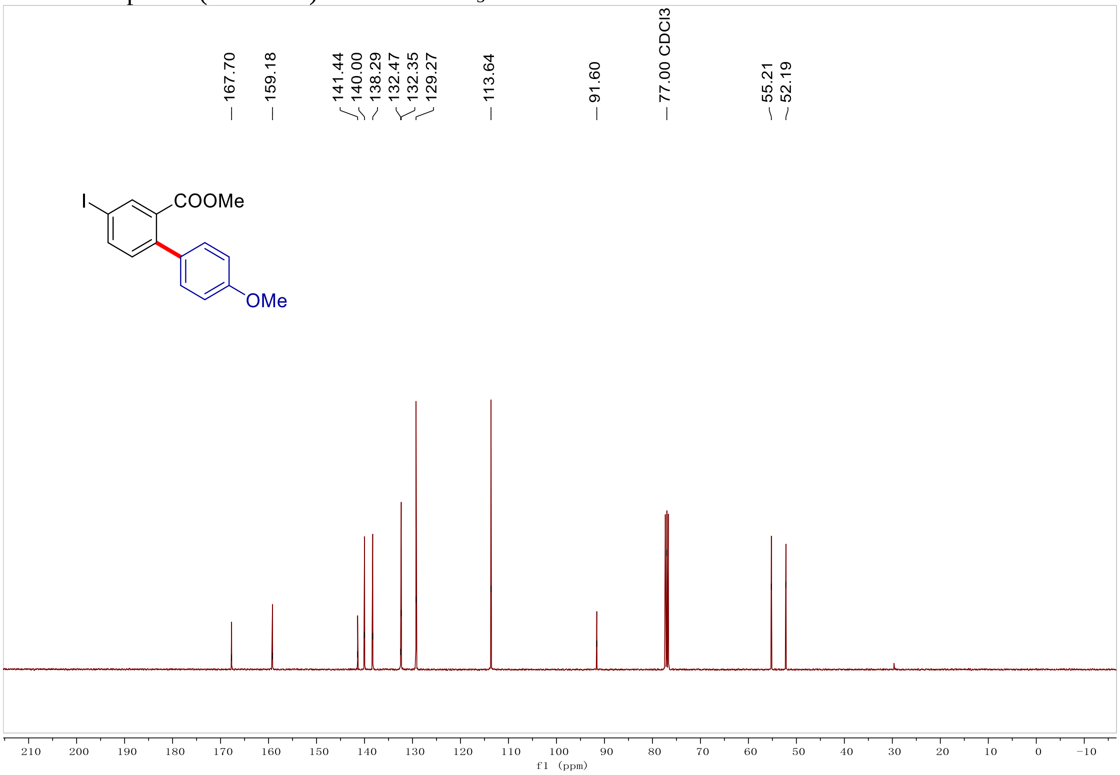

[1]**H NMR** spectra (400 MHz) of **52** in $CDCl_3$.

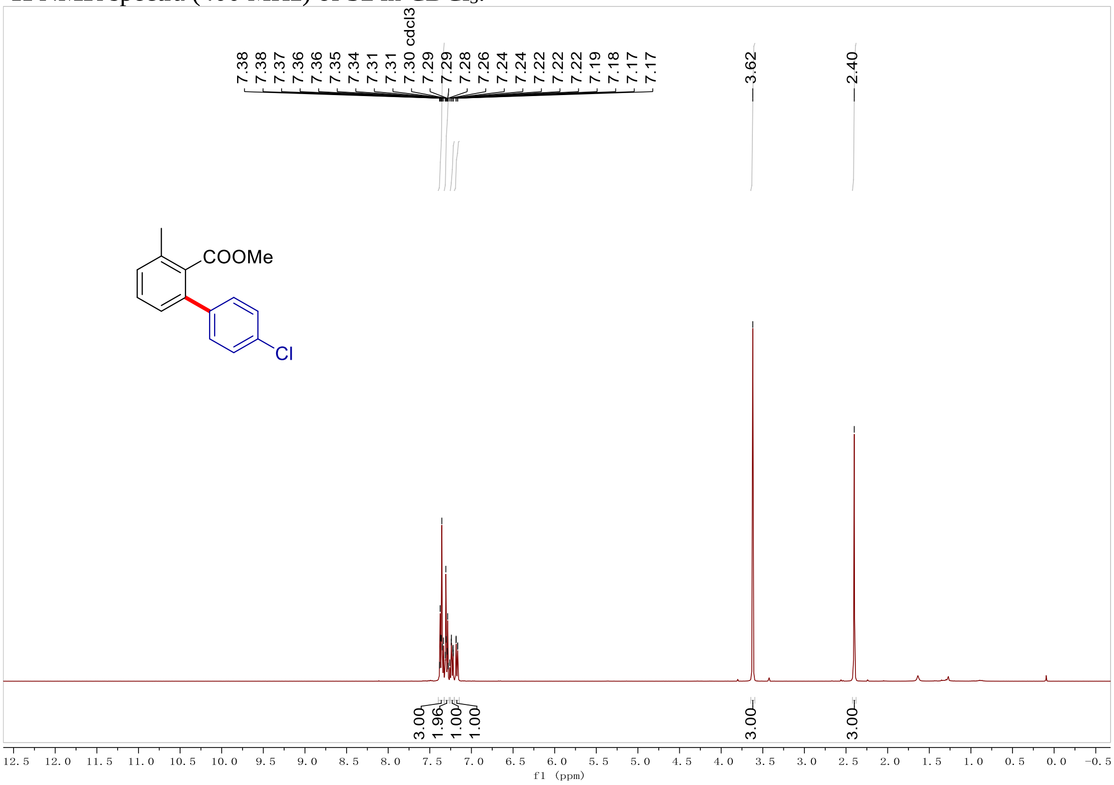

[13]**C NMR** spectra (101 MHz) of **52** in $CDCl_3$.

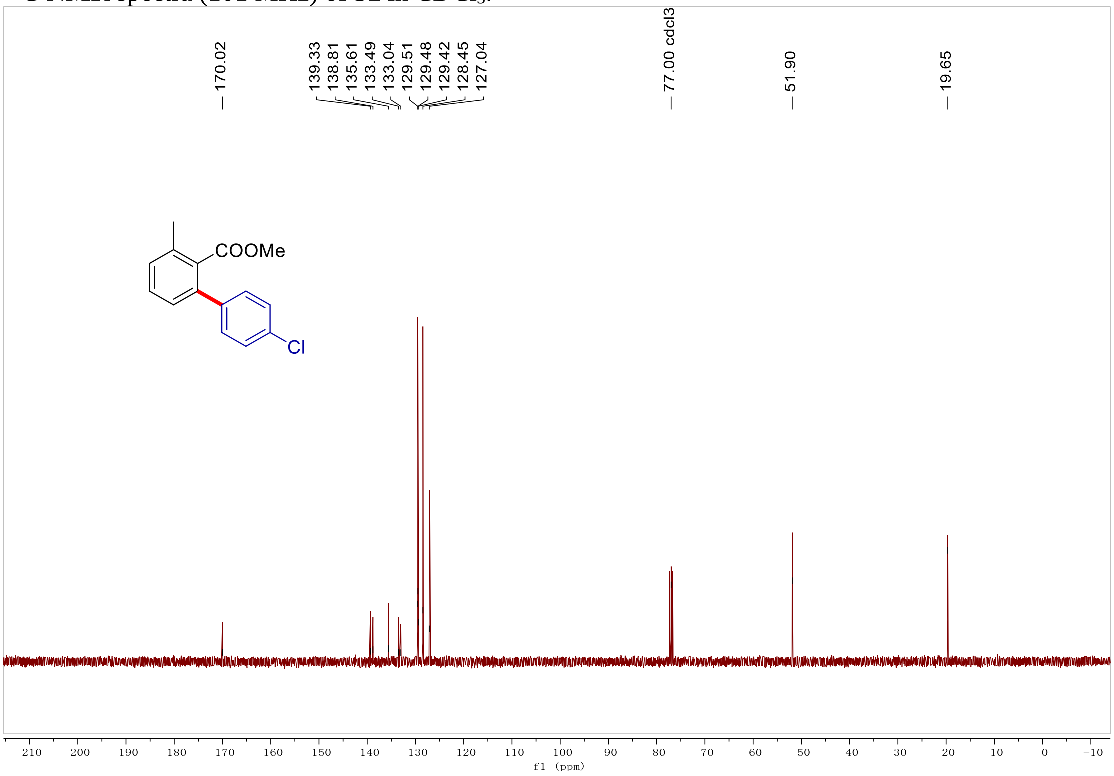

^{1}H NMR spectra (400 MHz) of **53** in $CDCl_3$.

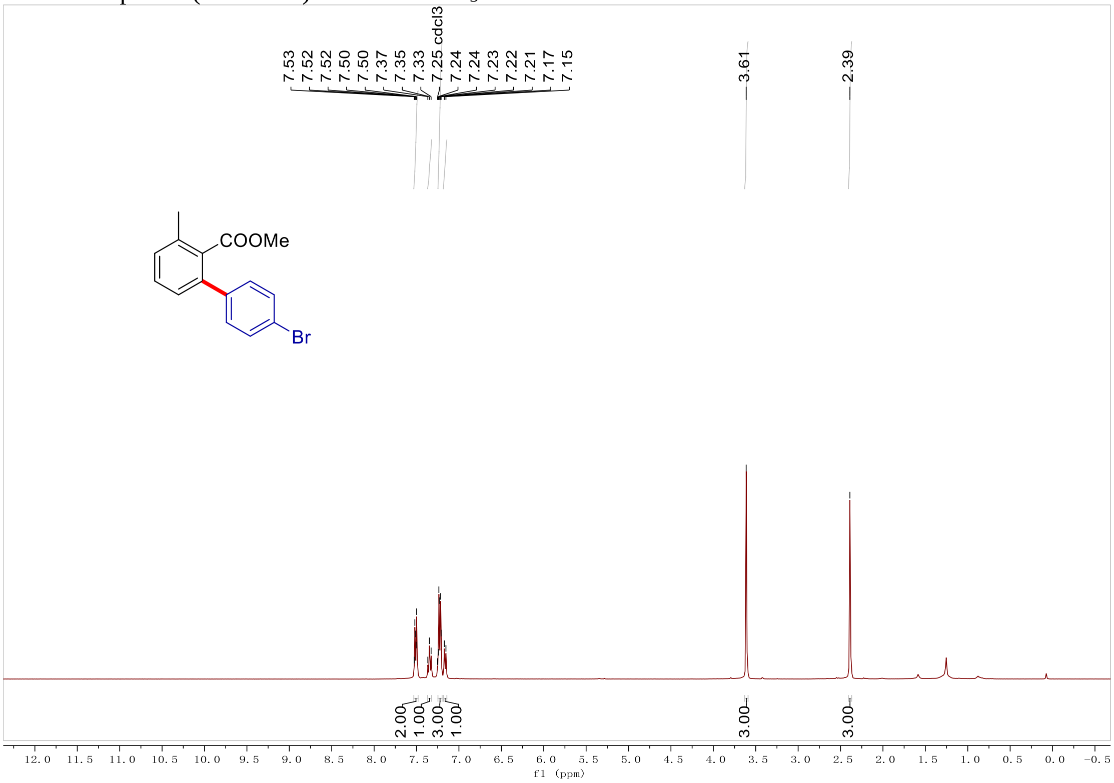

^{13}C NMR spectra (101 MHz) of **53** in $CDCl_3$.

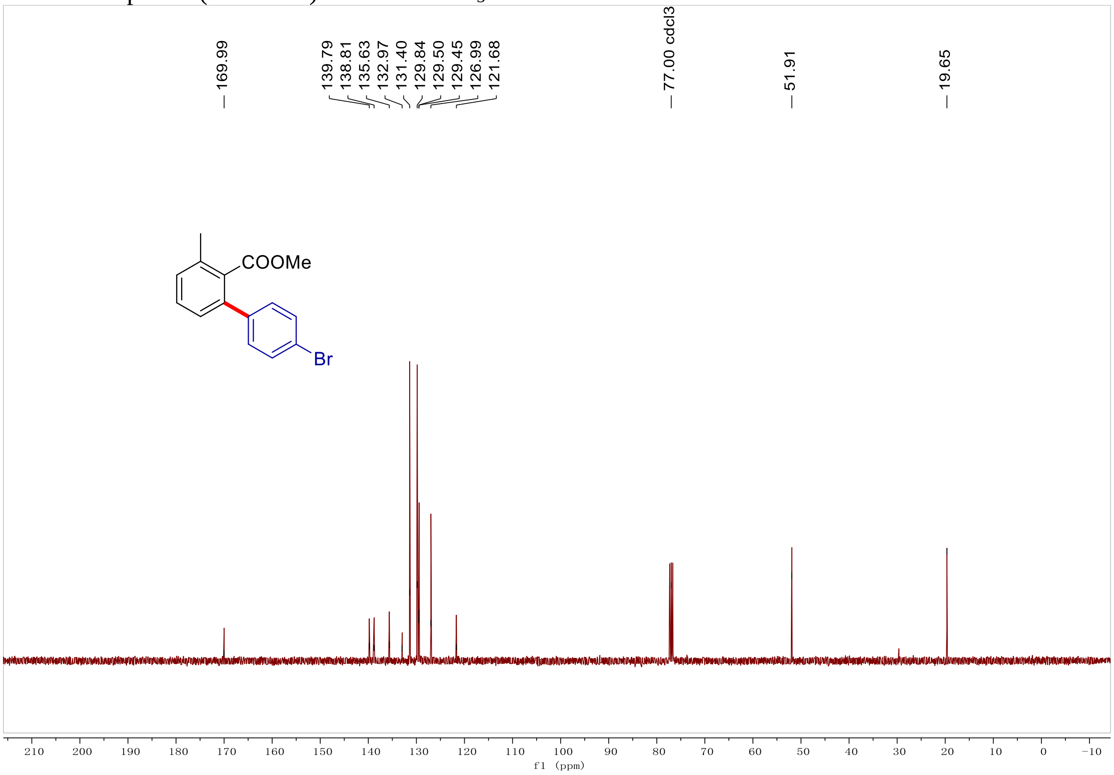

^{1}H NMR spectra (400 MHz) of **54** in $CDCl_3$.

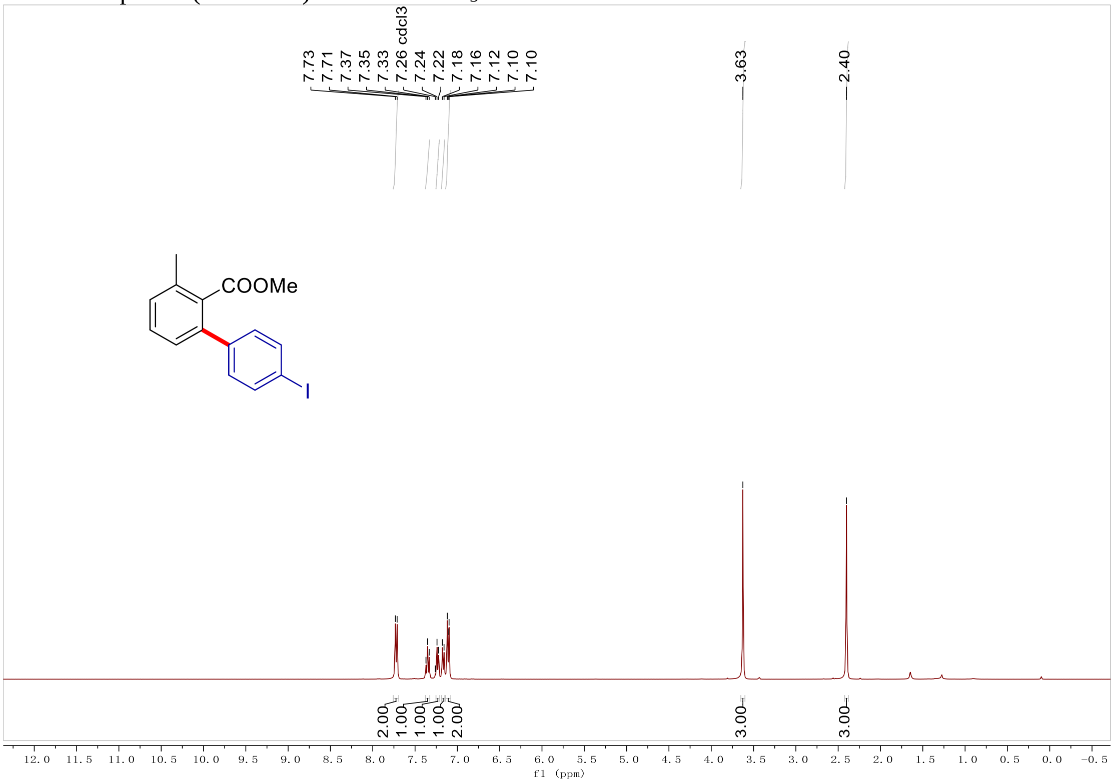

^{13}C NMR spectra (101 MHz) of **54** in $CDCl_3$.

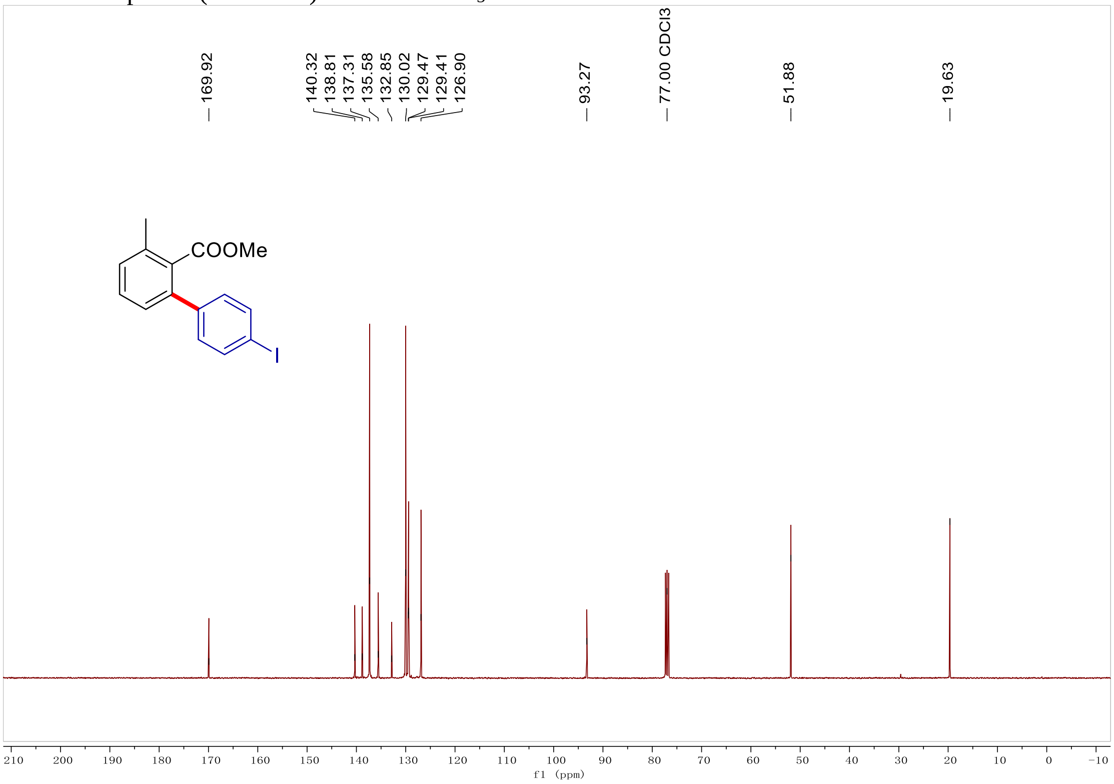

^{1}H NMR spectra (400 MHz) of **55** in $CDCl_3$.

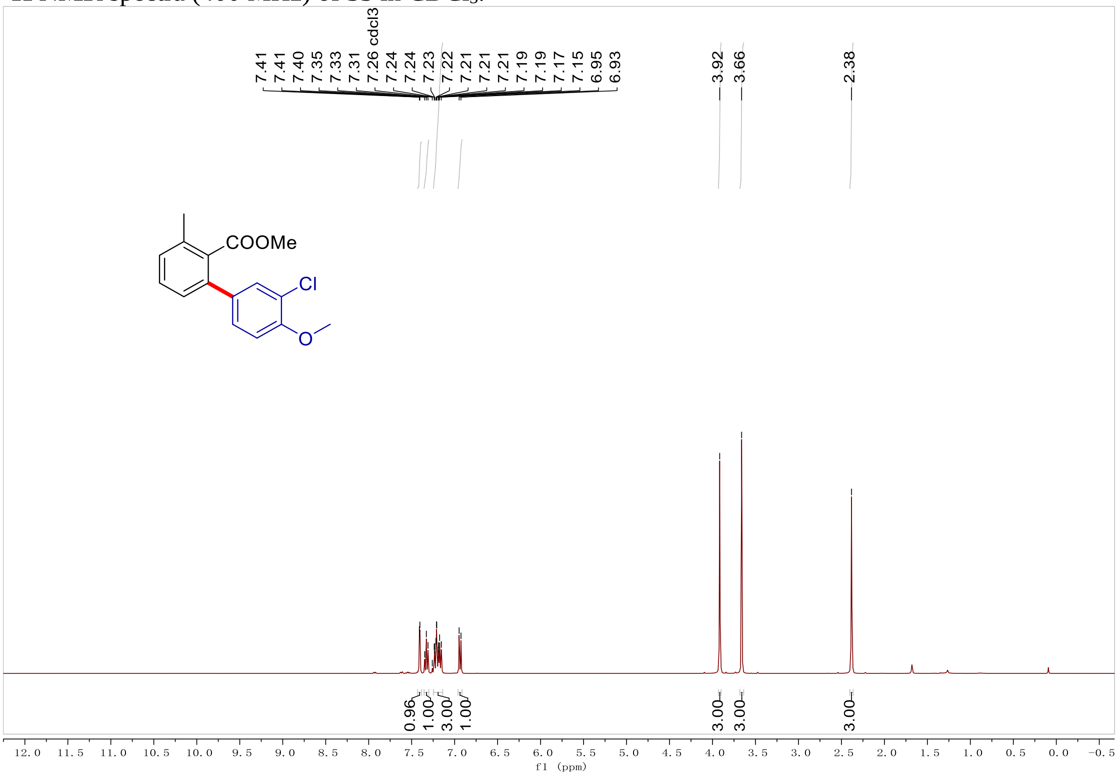

^{13}C NMR spectra (101 MHz) of **55** in $CDCl_3$.

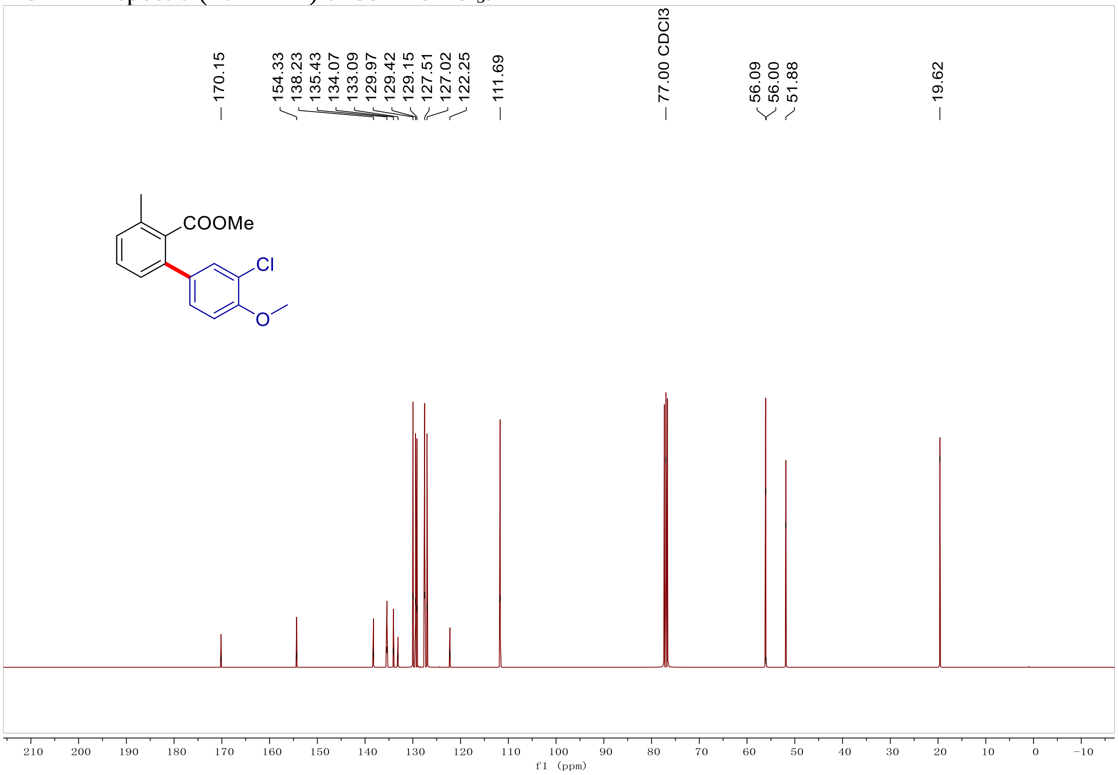

^{1}H NMR spectra (400 MHz) of **56** in $CDCl_3$.

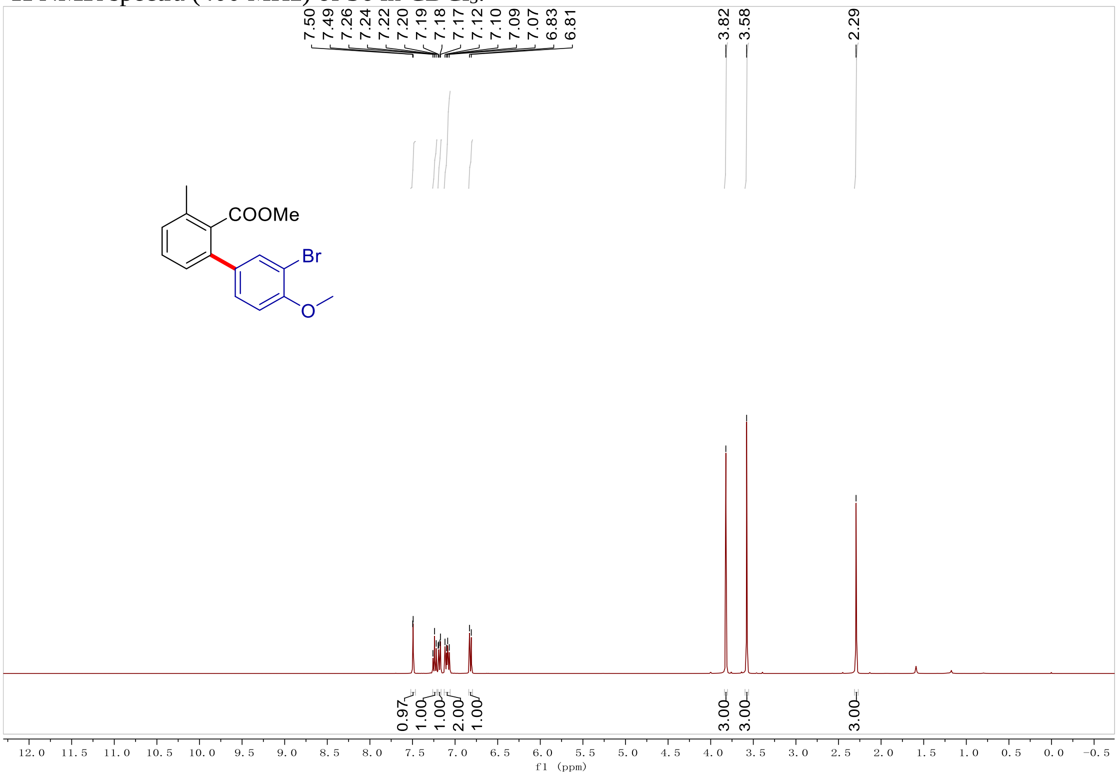

^{13}C NMR spectra (101 MHz) of **56** in $CDCl_3$.

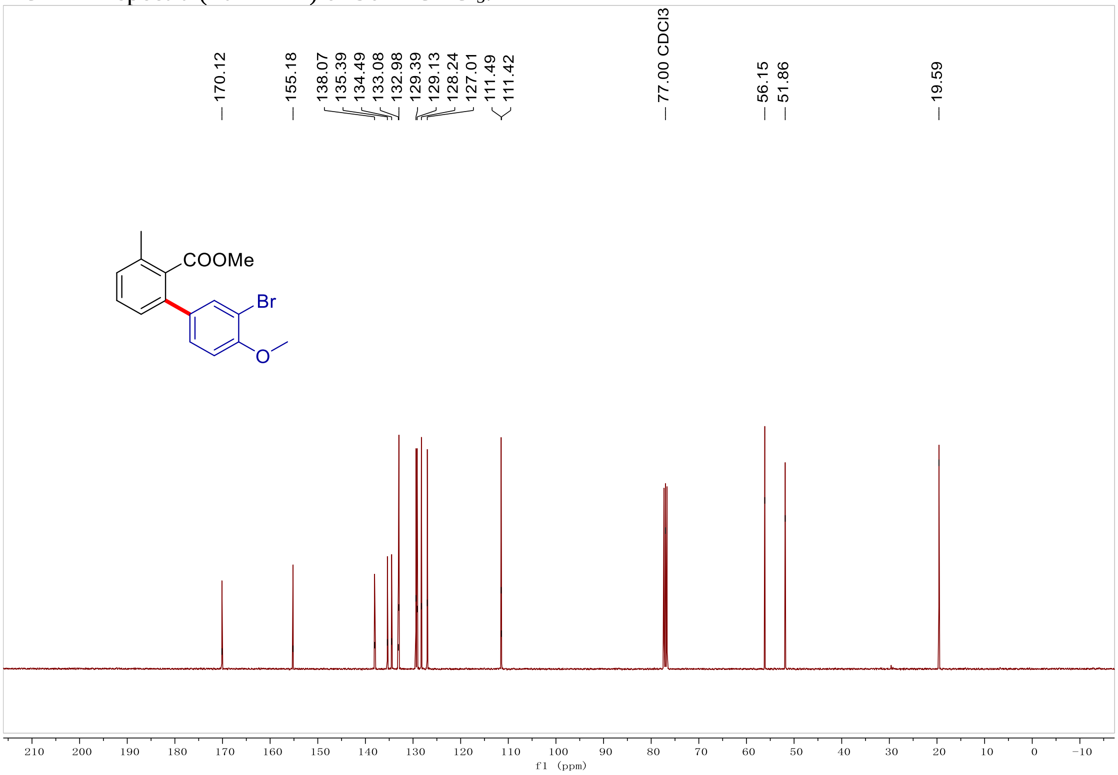

^{1}H NMR spectra (400 MHz) of **57** in $CDCl_3$.

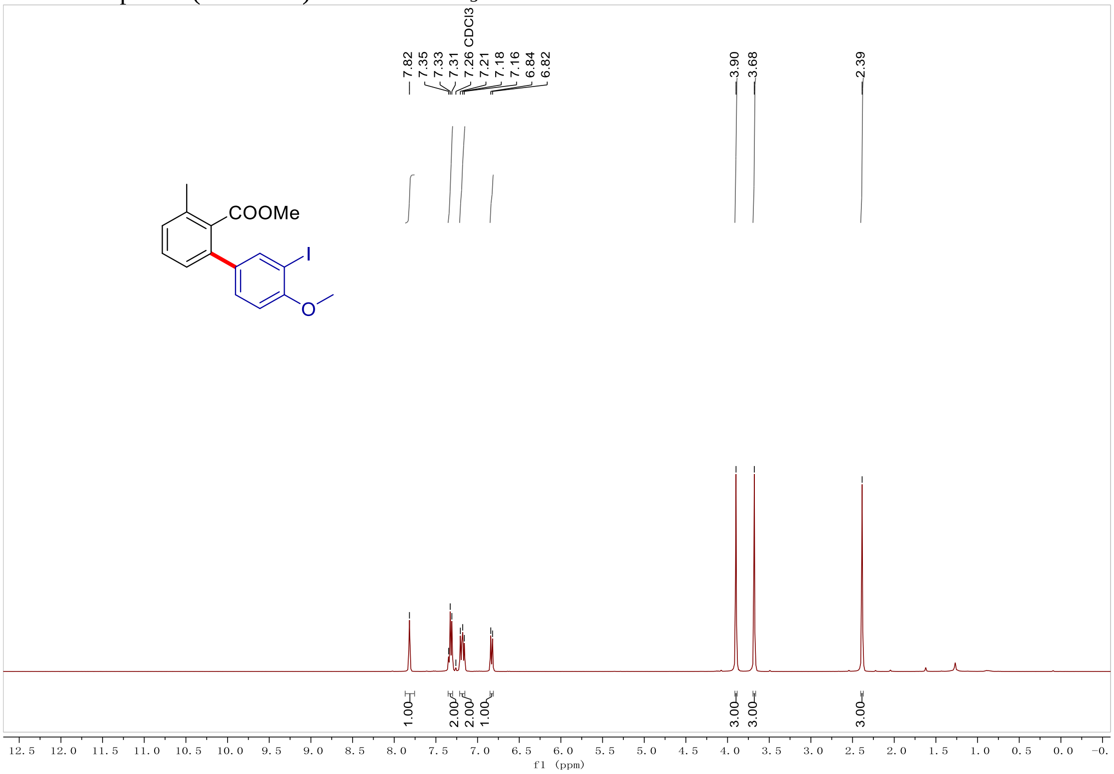

^{13}C NMR spectra (101 MHz) of **57** in $CDCl_3$.

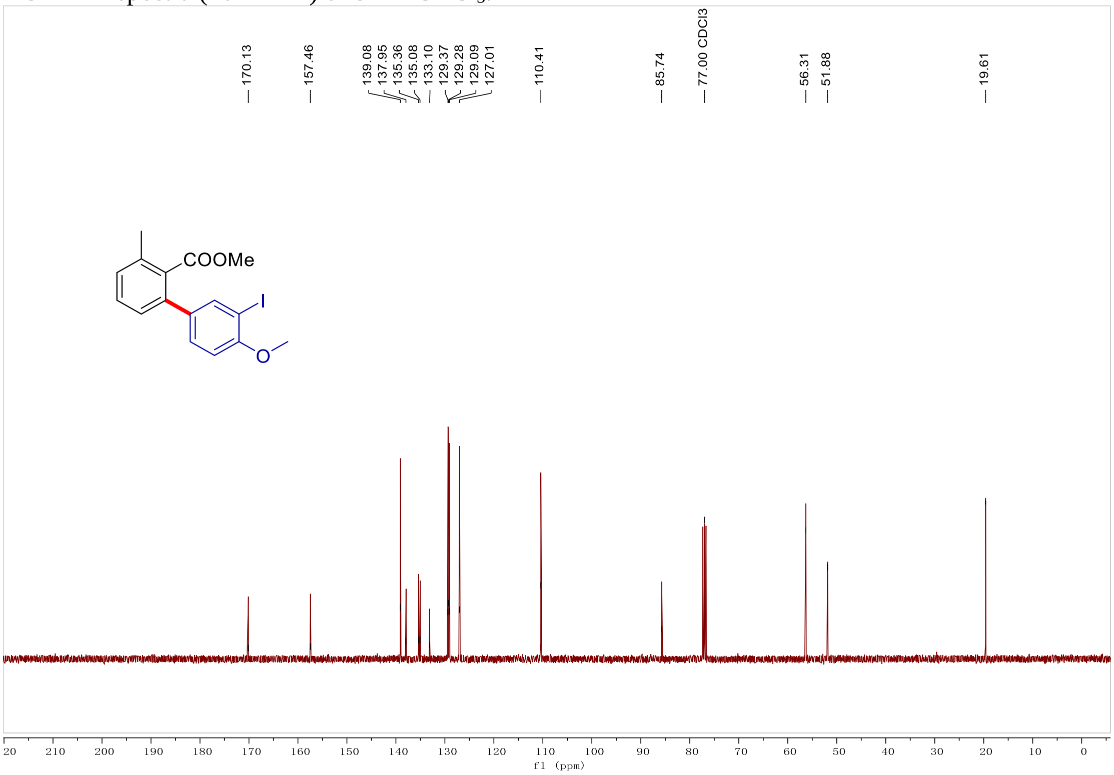

^{1}H NMR spectra (400 MHz) of **58** in $CDCl_3$.

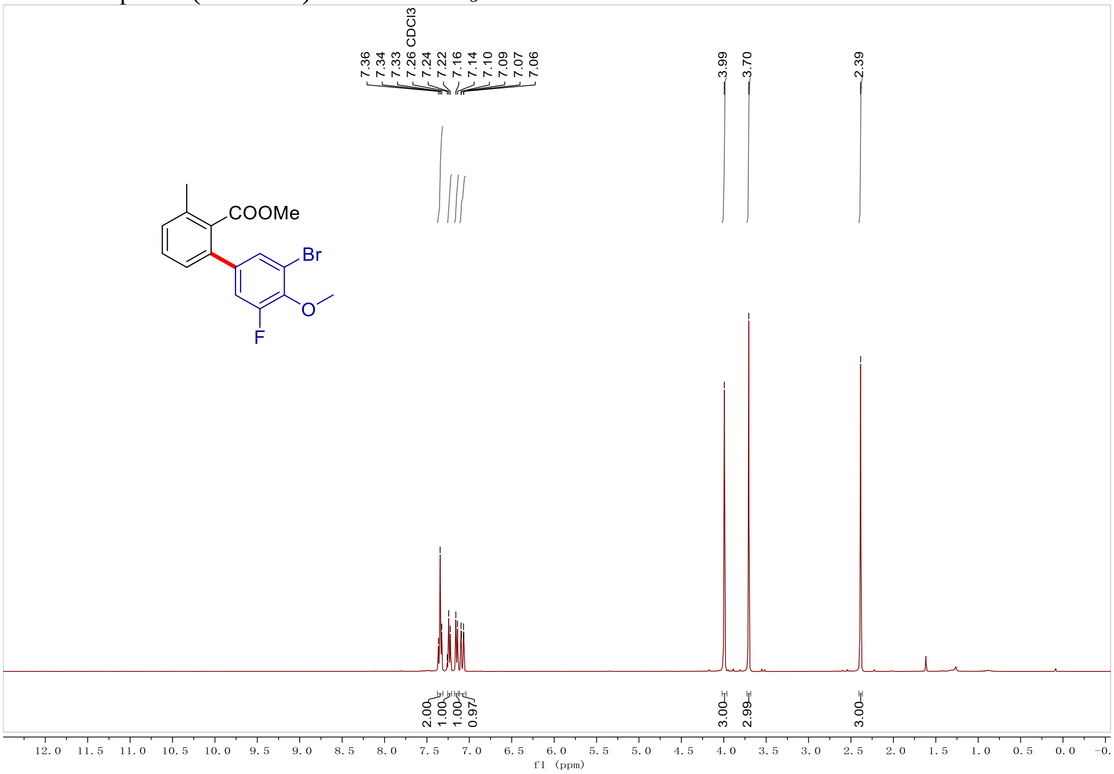

^{13}C NMR spectra (101 MHz) of **58** in $CDCl_3$.

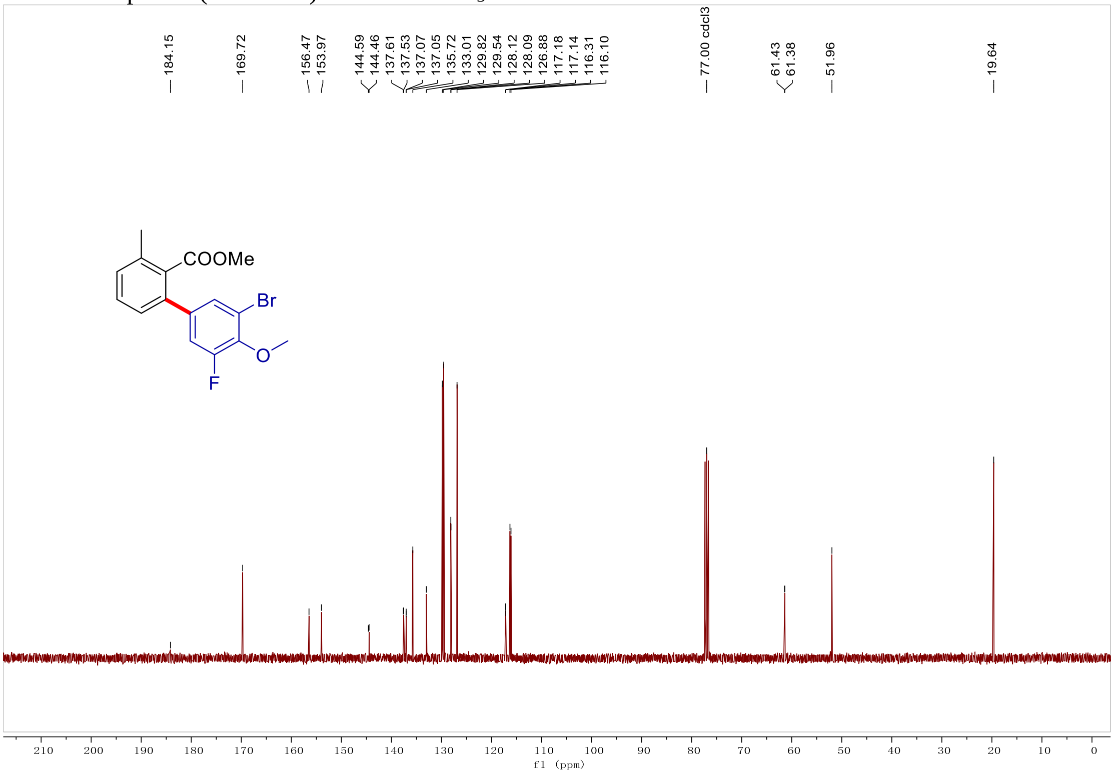

^{1}H NMR spectra (400 MHz) of **59** in $CDCl_3$.

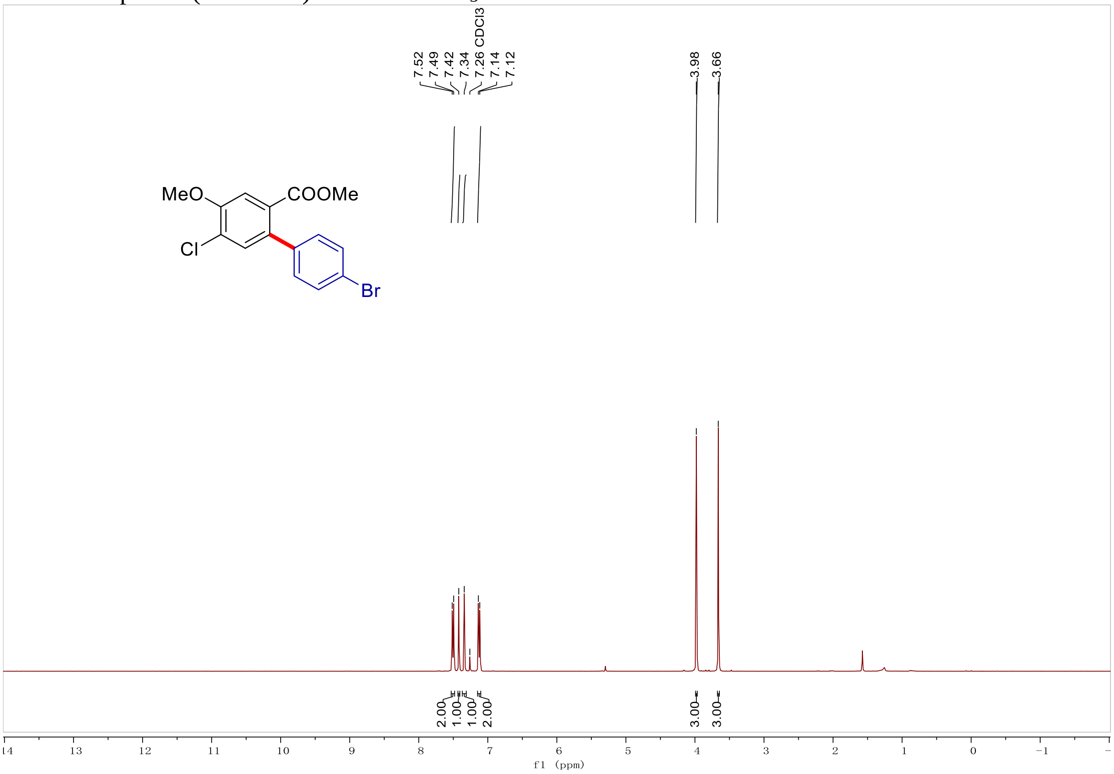

^{13}C NMR spectra (101 MHz) of **59** in $CDCl_3$.

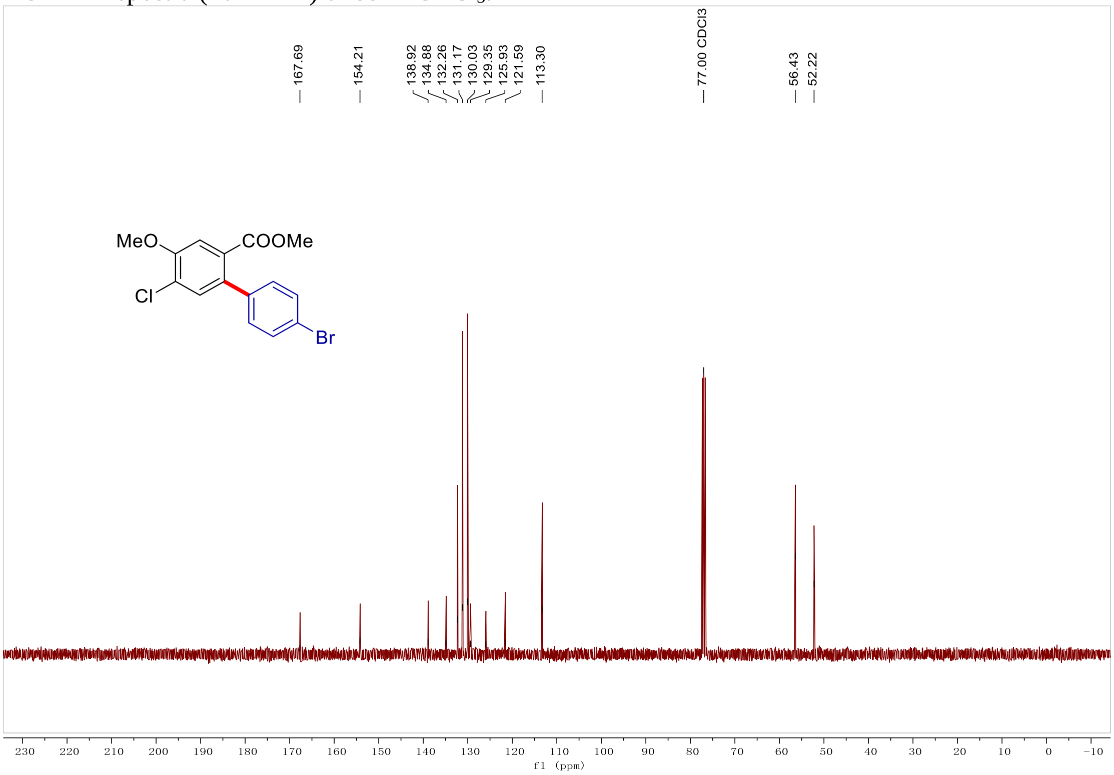

^{1}H NMR spectra (400 MHz) of **60** in $CDCl_3$.

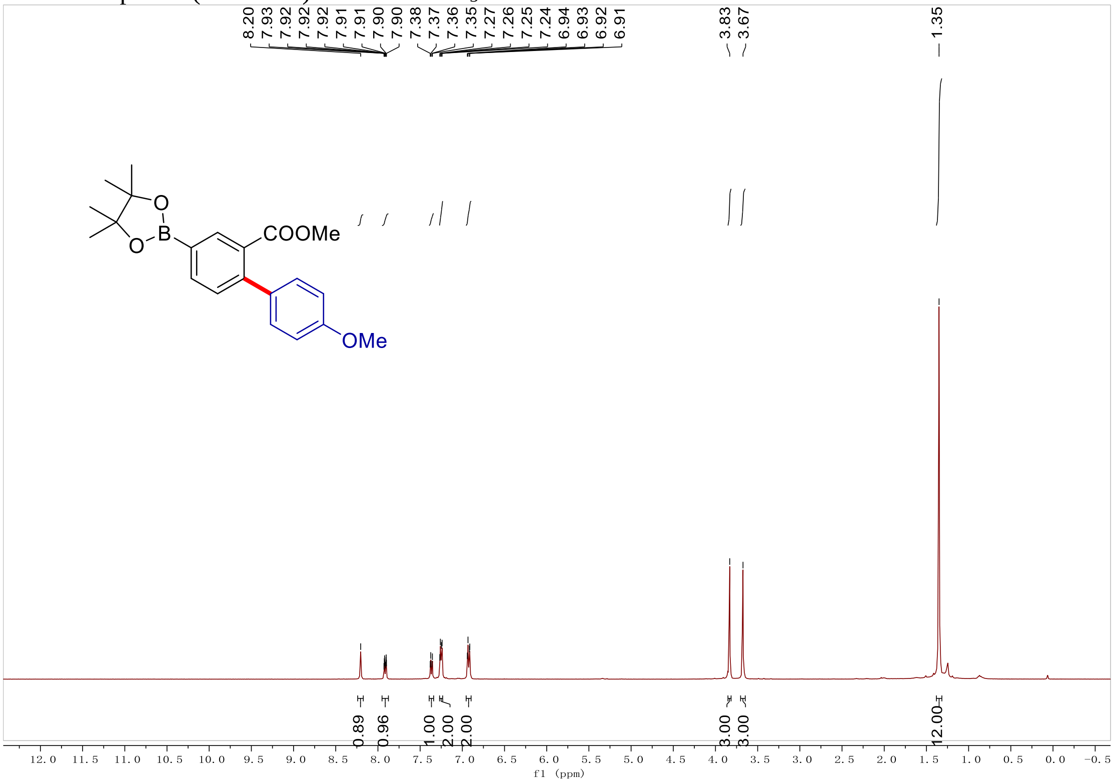

^{13}C NMR spectra (101 MHz) of **60** in $CDCl_3$.

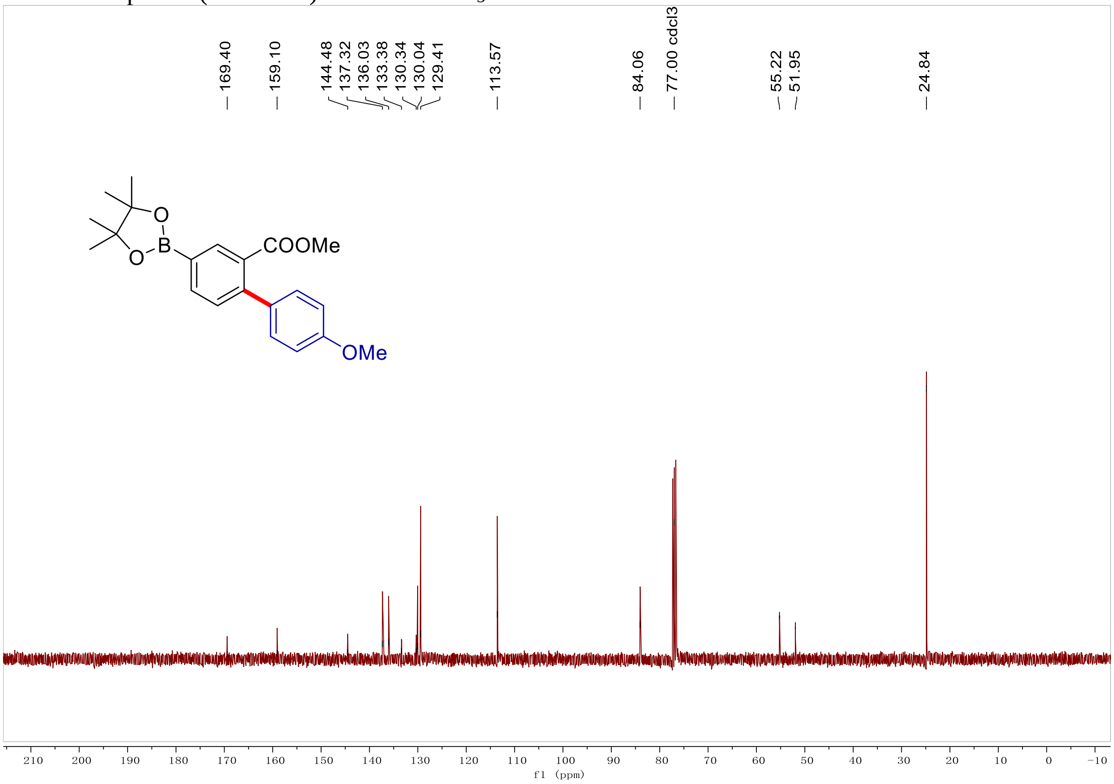

^{1}**H NMR** spectra (400 MHz) of **61** in $CDCl_3$.

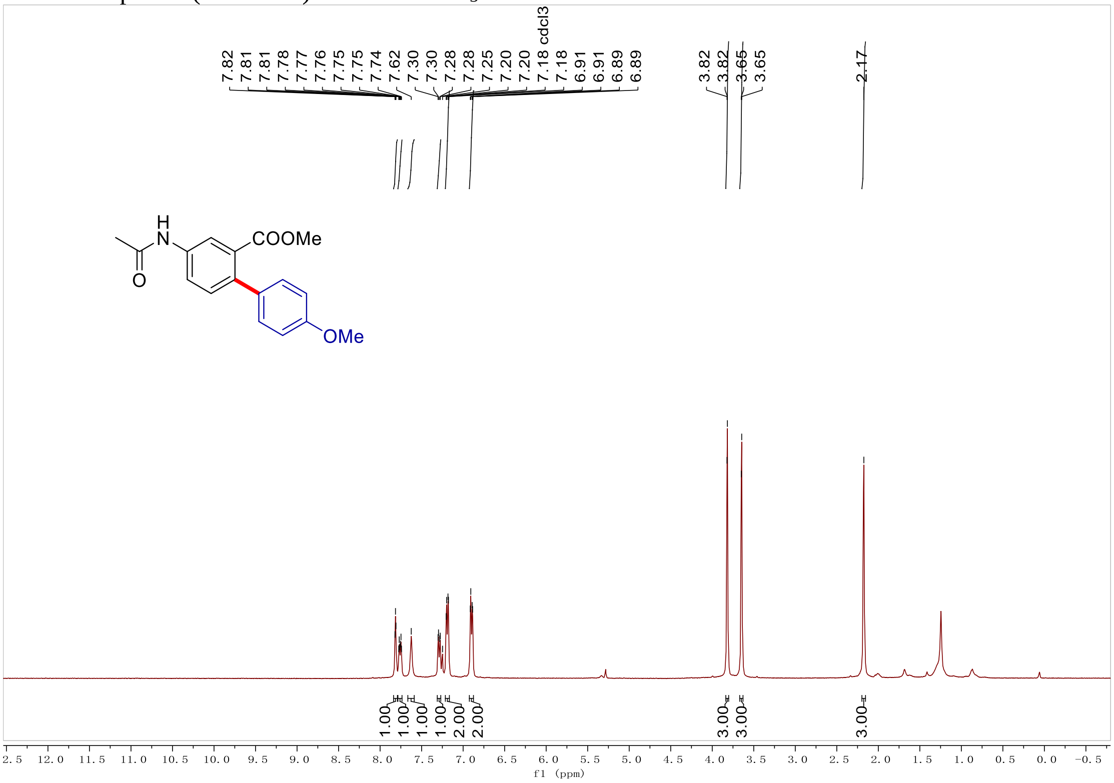

^{13}**C NMR** spectra (101 MHz) of **61** in $CDCl_3$.

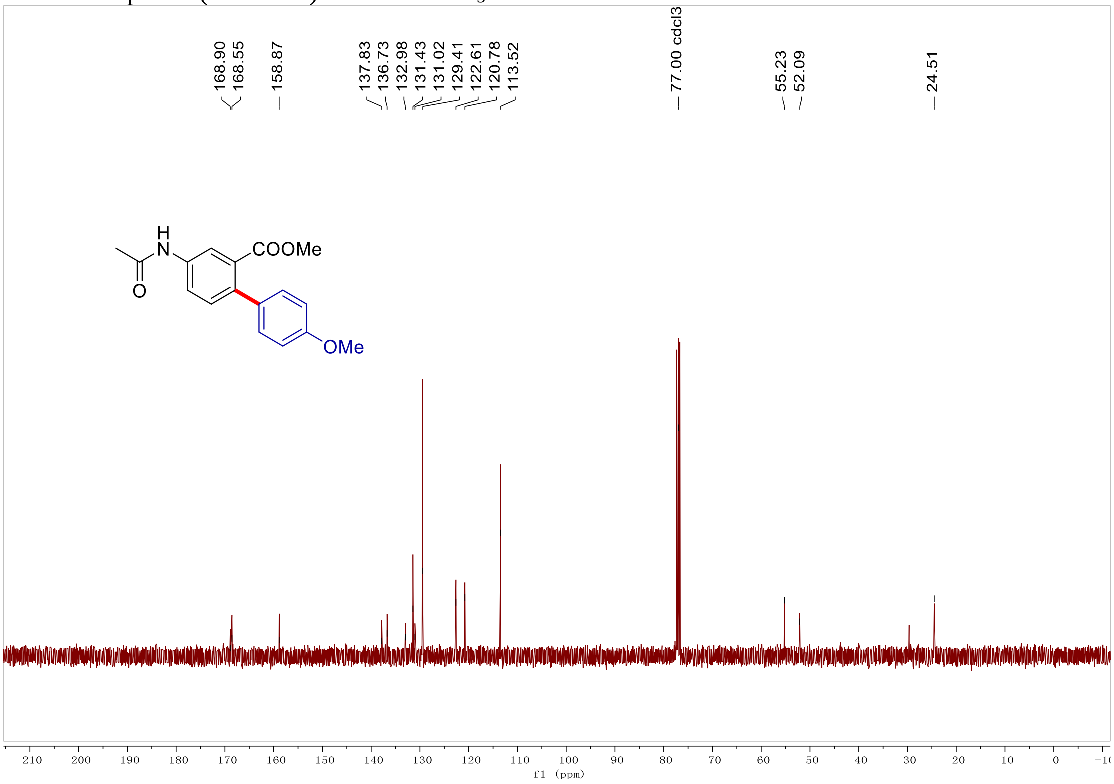

^{1}H NMR spectra (400 MHz) of **62** in $CDCl_3$.

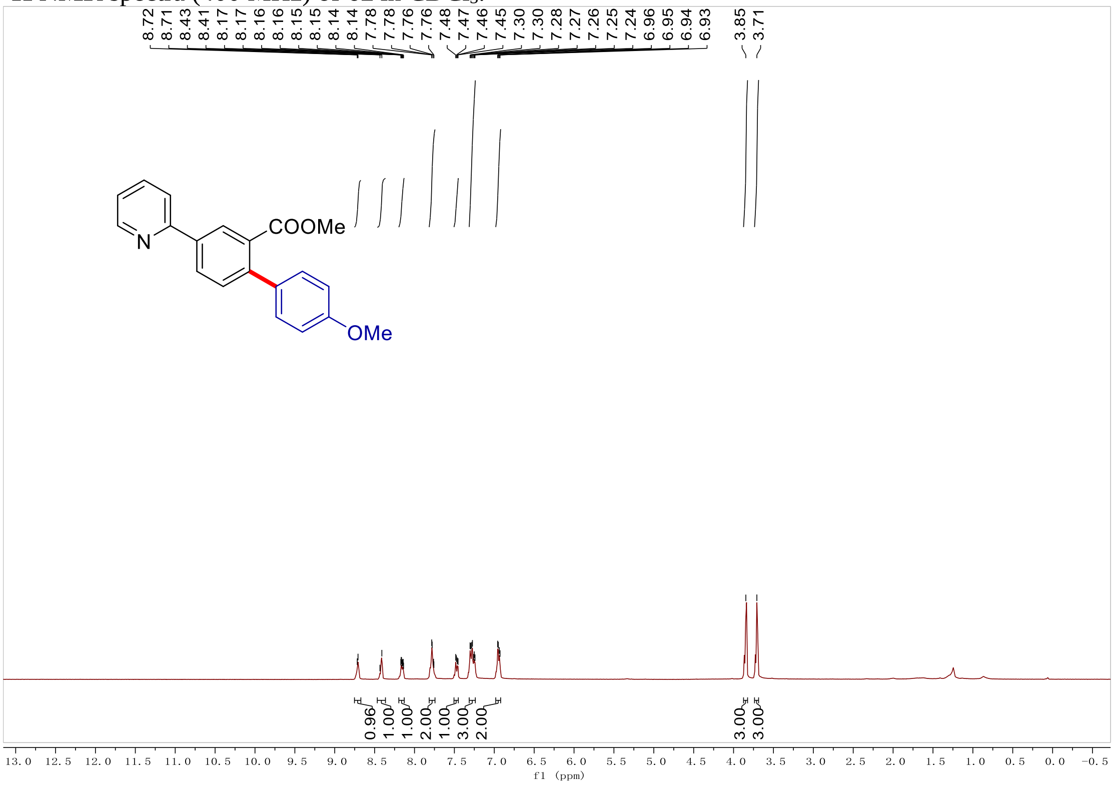

^{13}C NMR spectra (101 MHz) of **62** in $CDCl_3$.

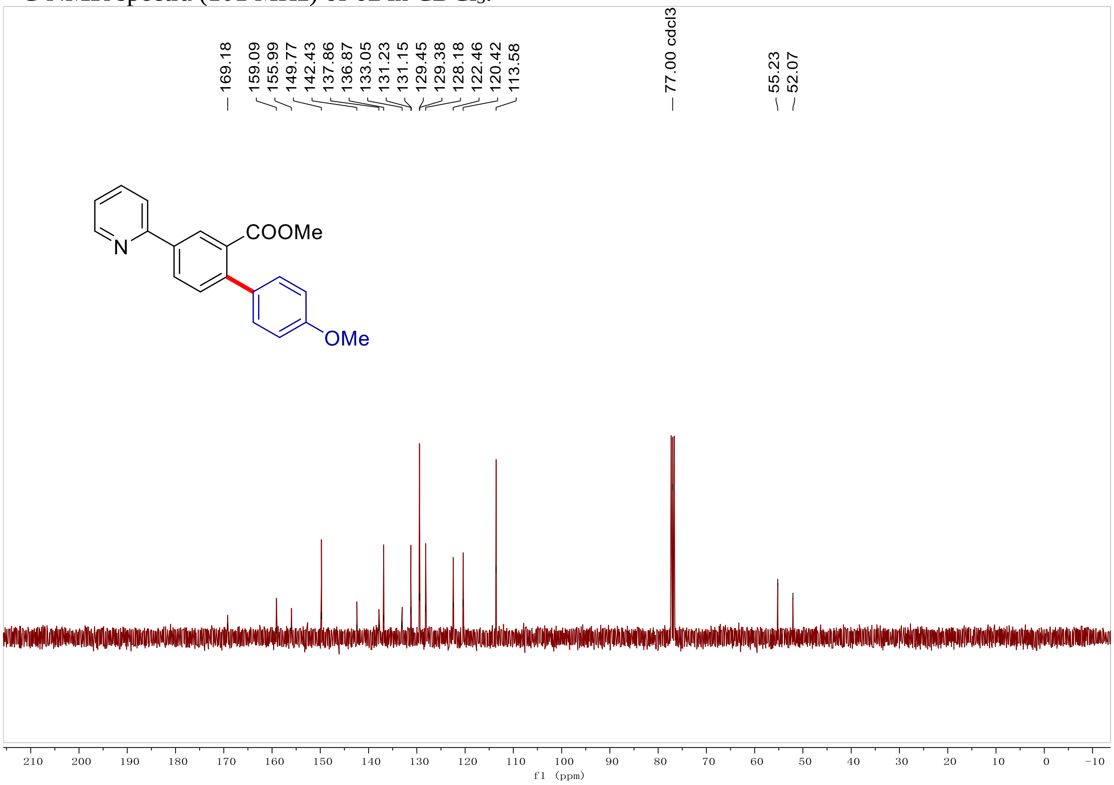

^{1}H NMR spectra (400 MHz) of **63** in DMSO-d_6.

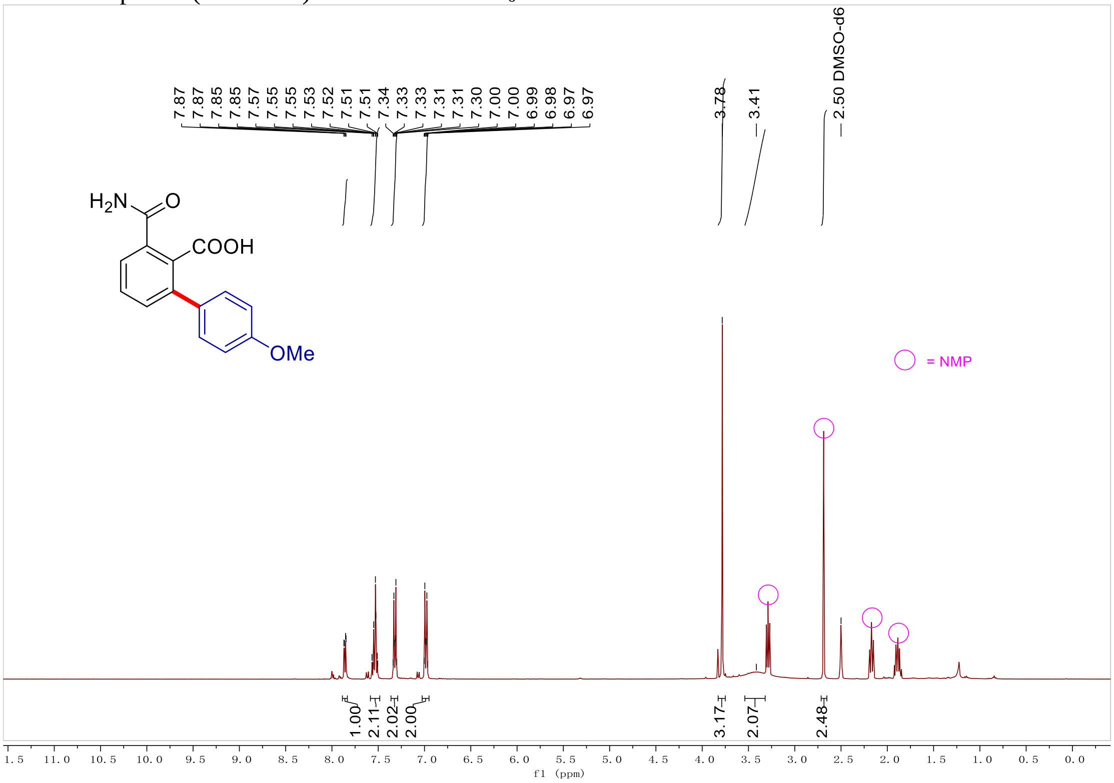

^{13}C NMR spectra (101 MHz) of **63** in DMSO-d_6.

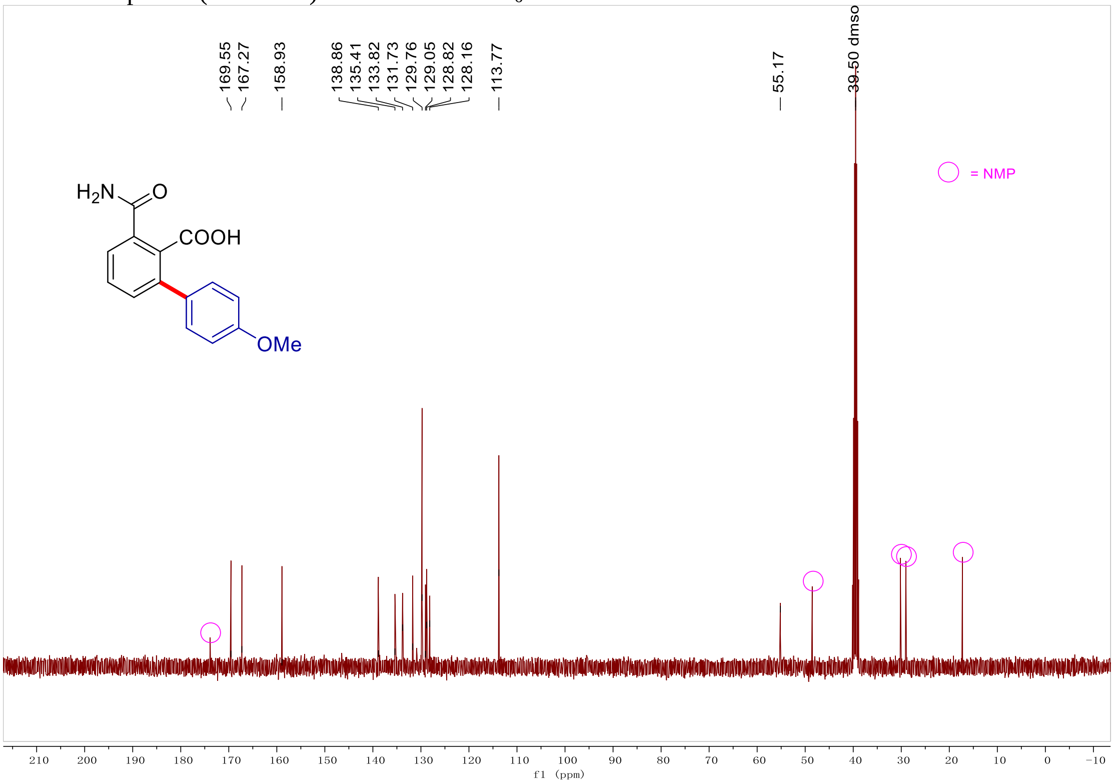

^{1}H NMR spectra (400 MHz) of **64** in $CDCl_3$.

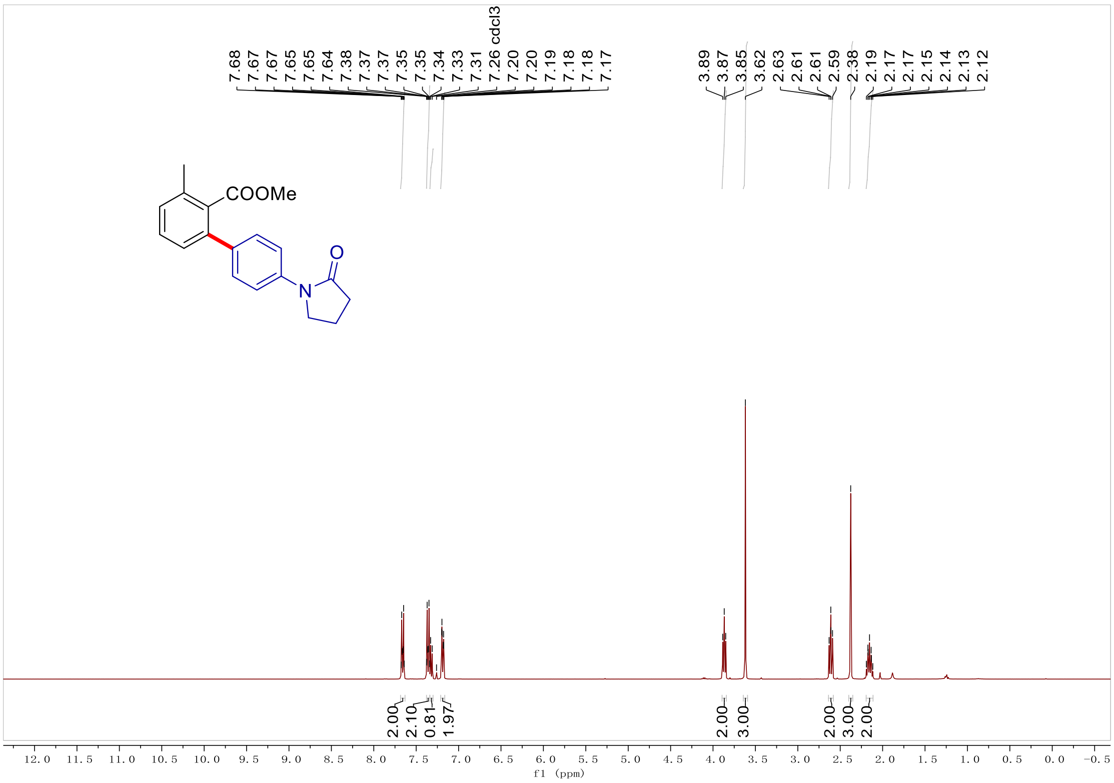

^{13}C NMR spectra (101 MHz) of **64** in $CDCl_3$.

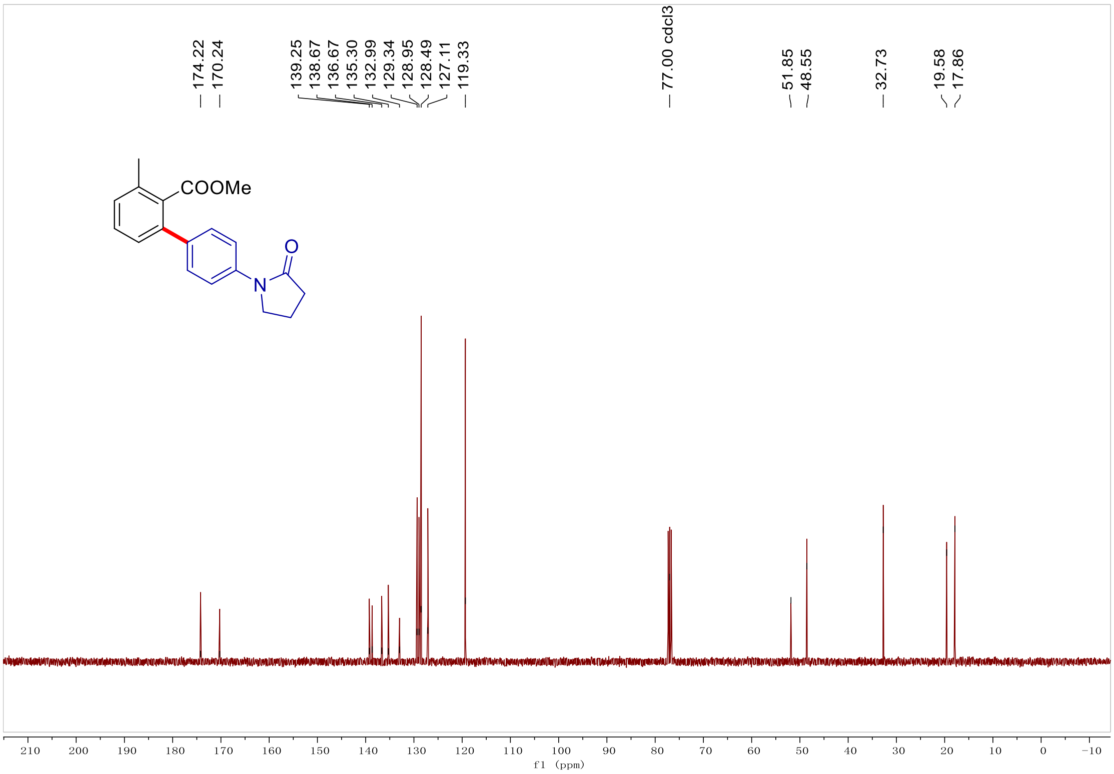

^{1}**H NMR** spectra (400 MHz) of **65** in $CDCl_3$.

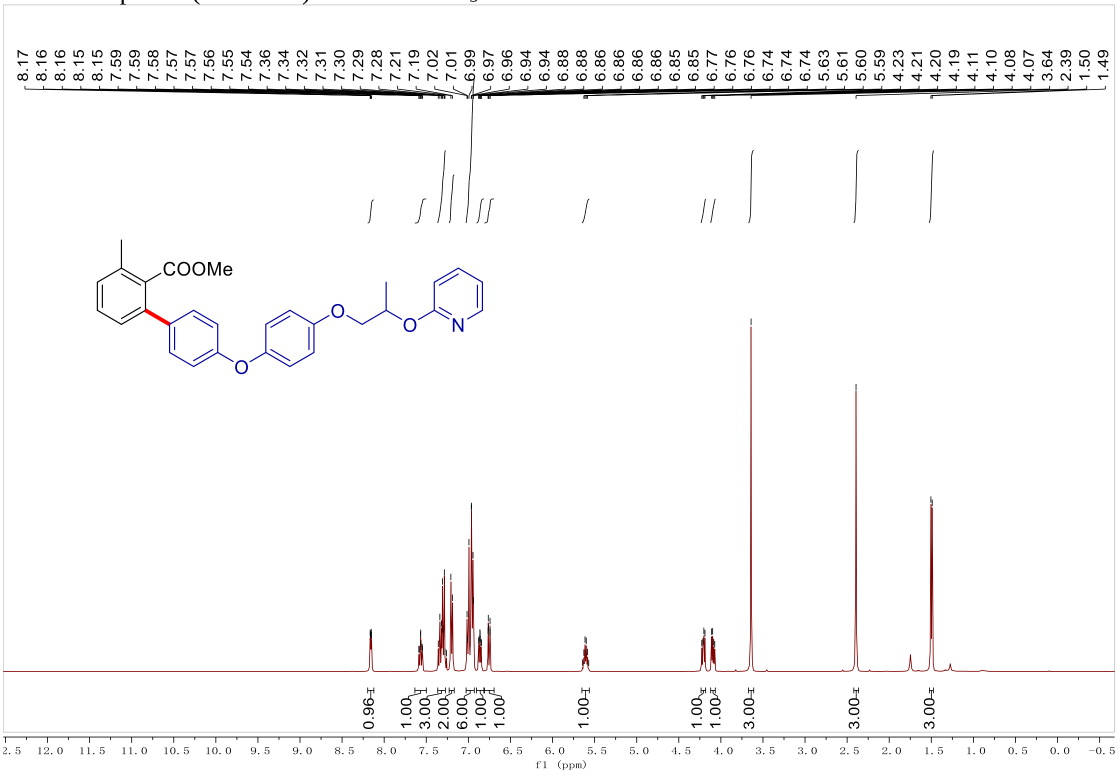

^{13}**C NMR** spectra (101 MHz) of **65** in $CDCl_3$.

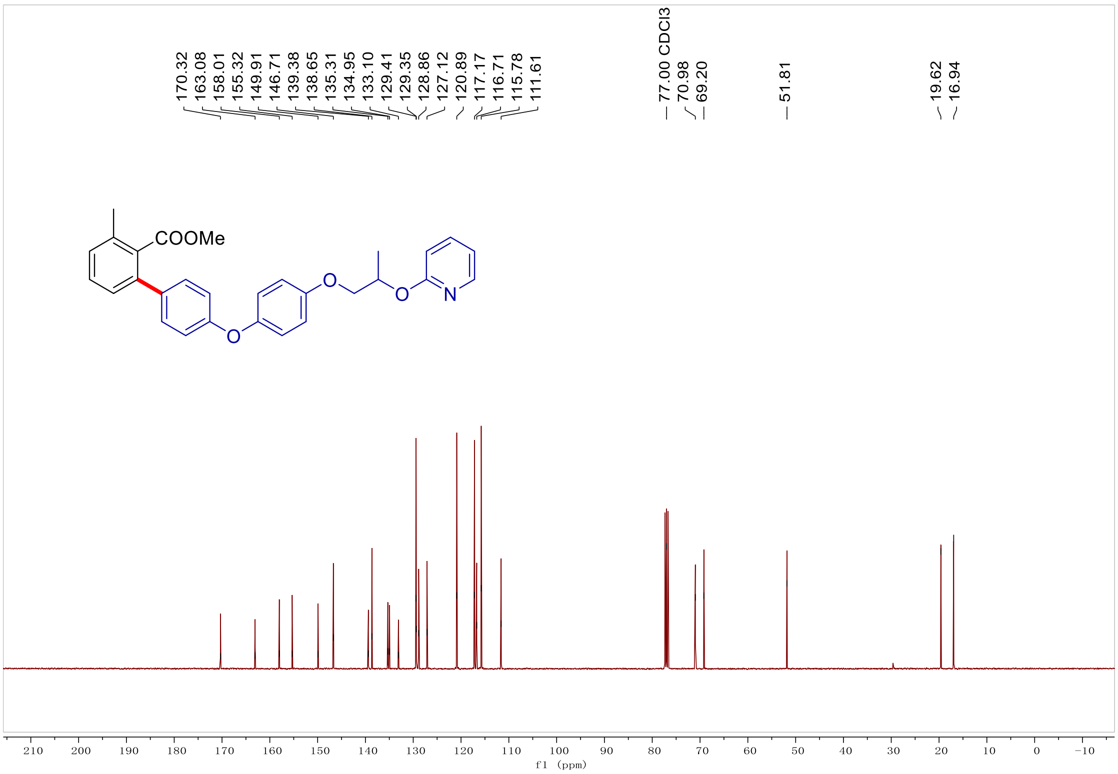

[1]**H NMR** spectra (400 MHz) of **66** in $CDCl_3$.

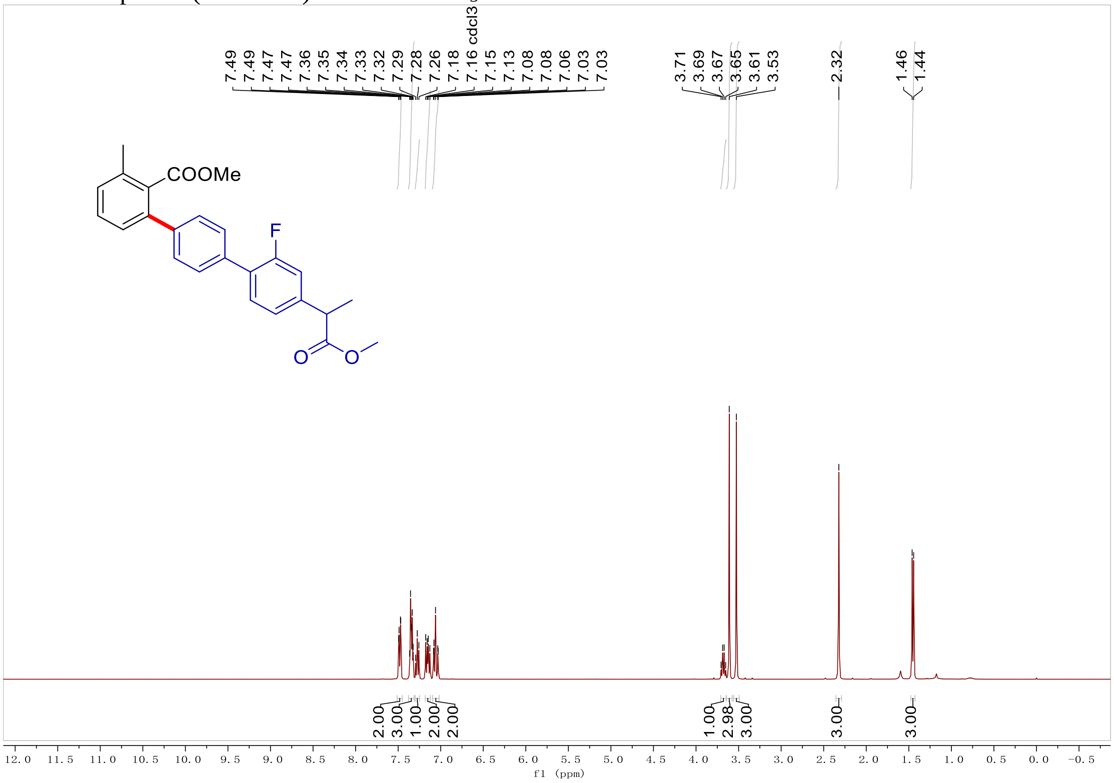

[13]**C NMR** spectra (101 MHz) of **66** in $CDCl_3$.

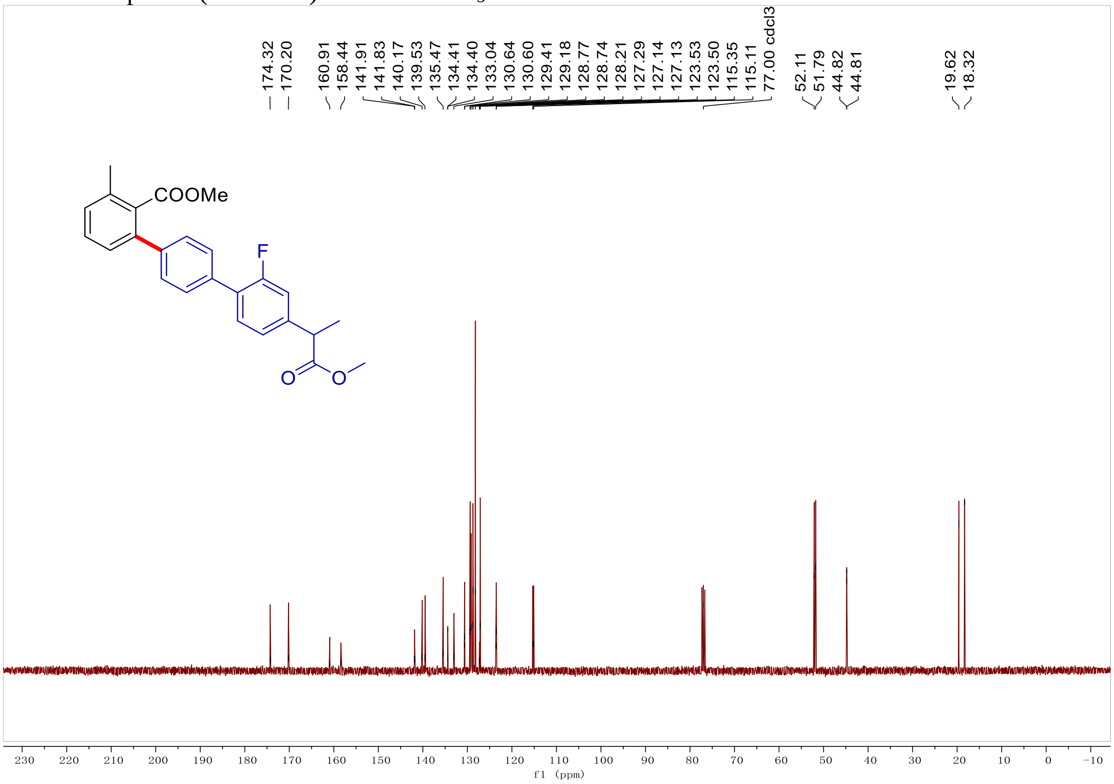

[1]**H NMR** spectra (400 MHz) of **67** in $CDCl_3$.

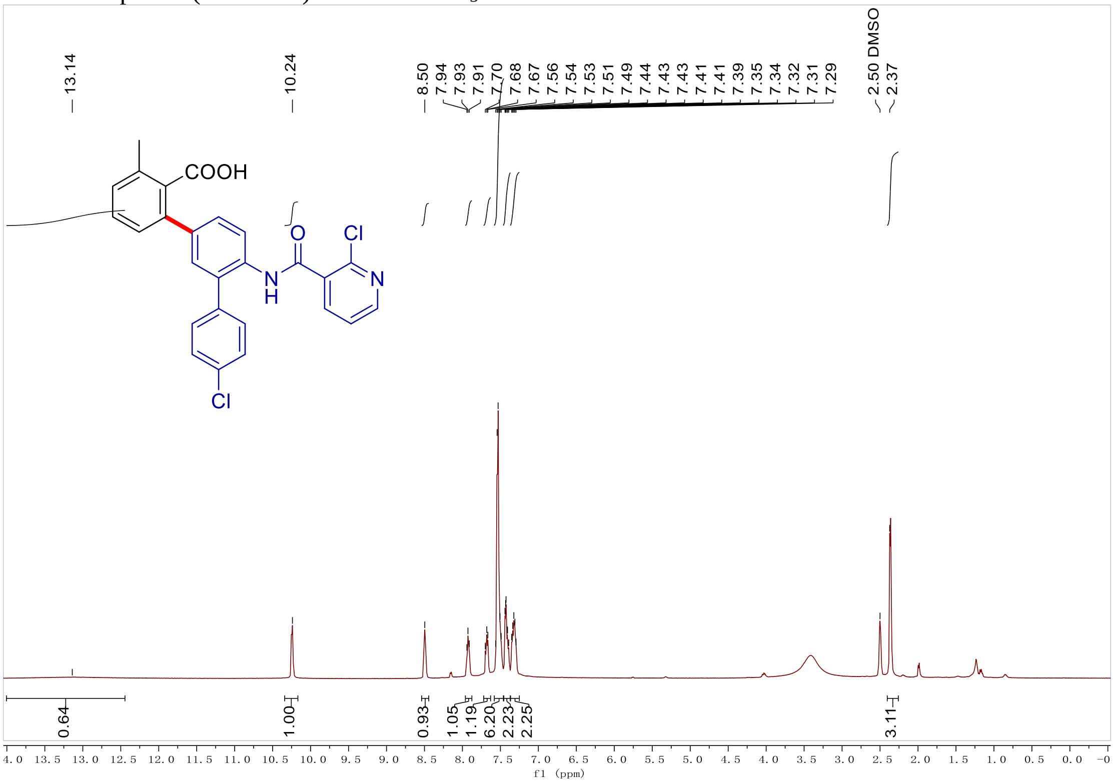

[13]**C NMR** spectra (101 MHz) of **67** in $CDCl_3$.

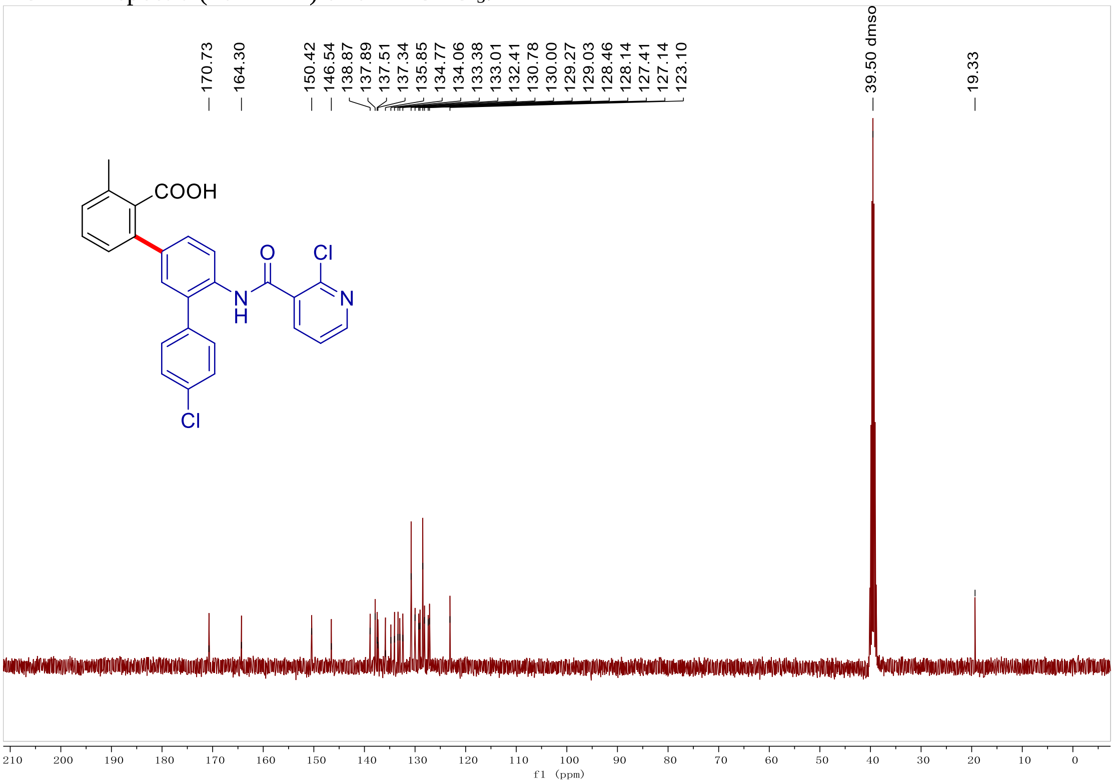

^{1}H NMR spectra (400 MHz) of **68** in $CDCl_3$.

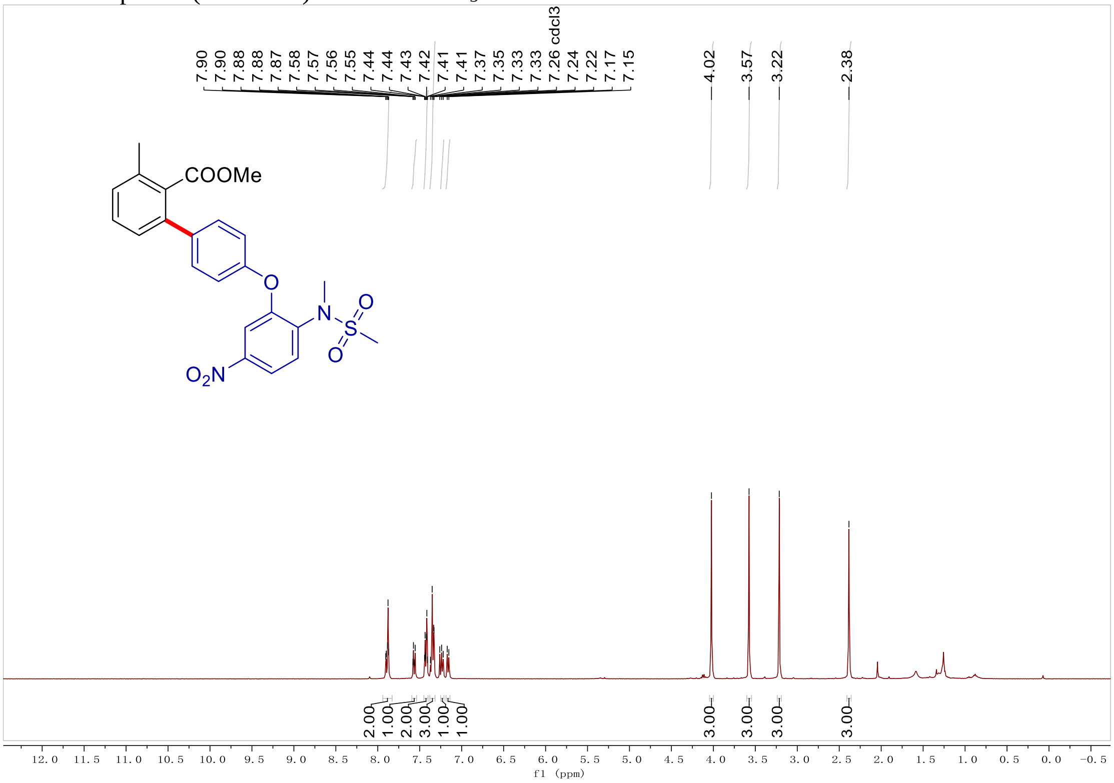

^{13}C NMR spectra (101 MHz) of **68** in $CDCl_3$.

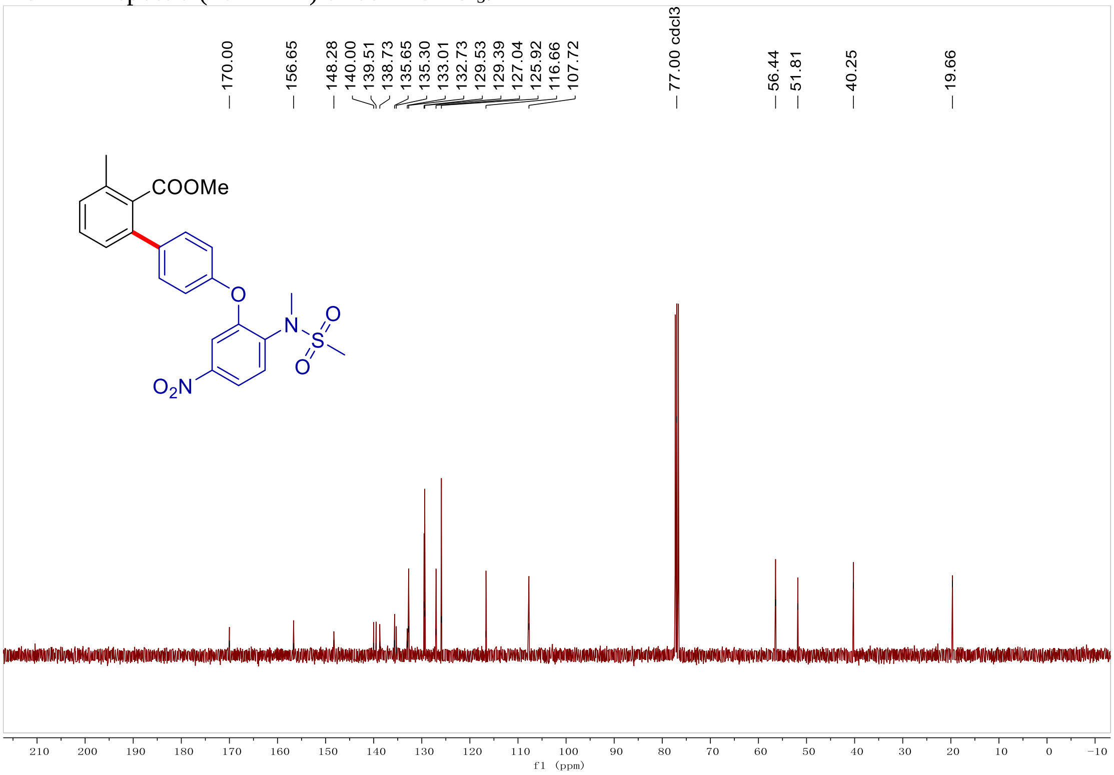

^{1}H NMR spectra (400 MHz) of **69** in $CDCl_3$.

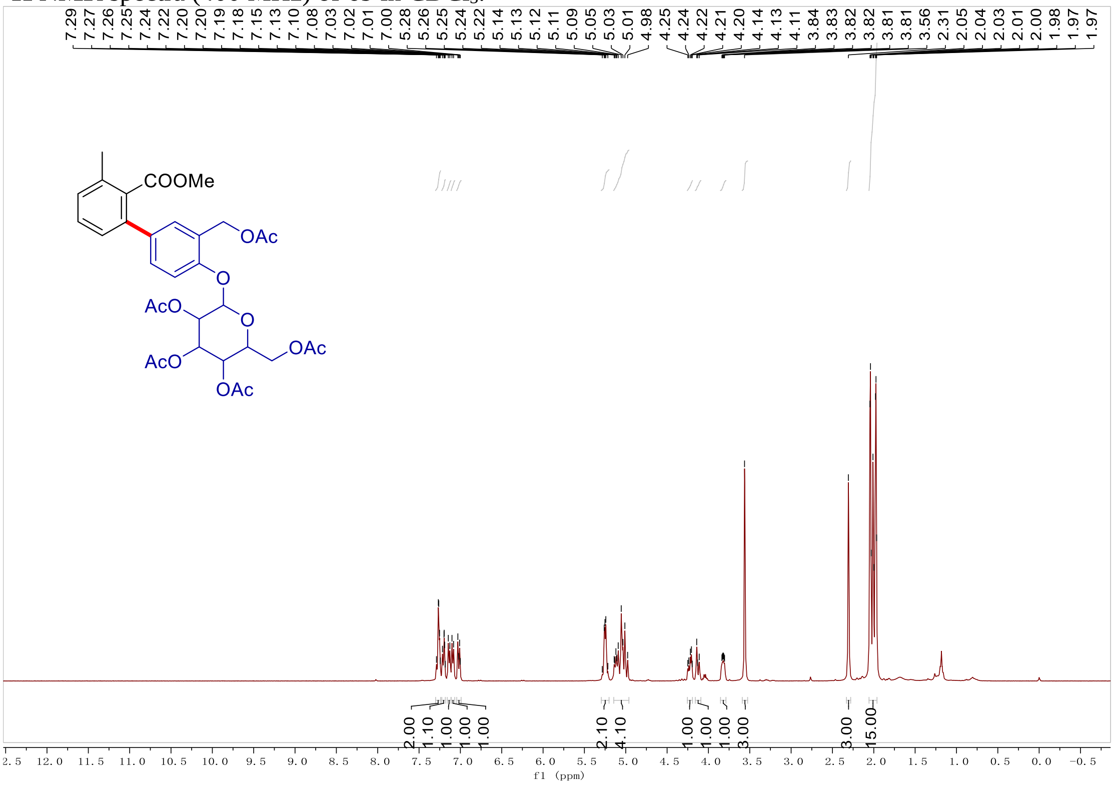

^{13}C NMR spectra (101 MHz) of **69** in $CDCl_3$.

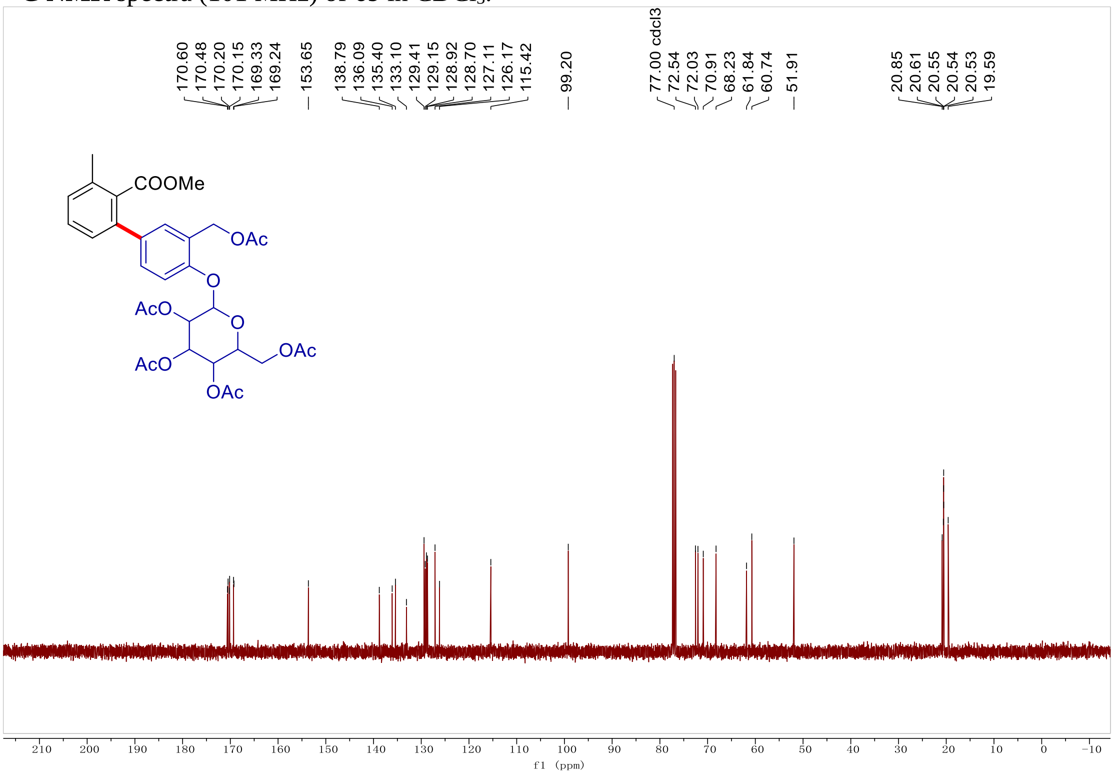

[1]**H NMR** spectra (400 MHz) of **70** in $CDCl_3$.

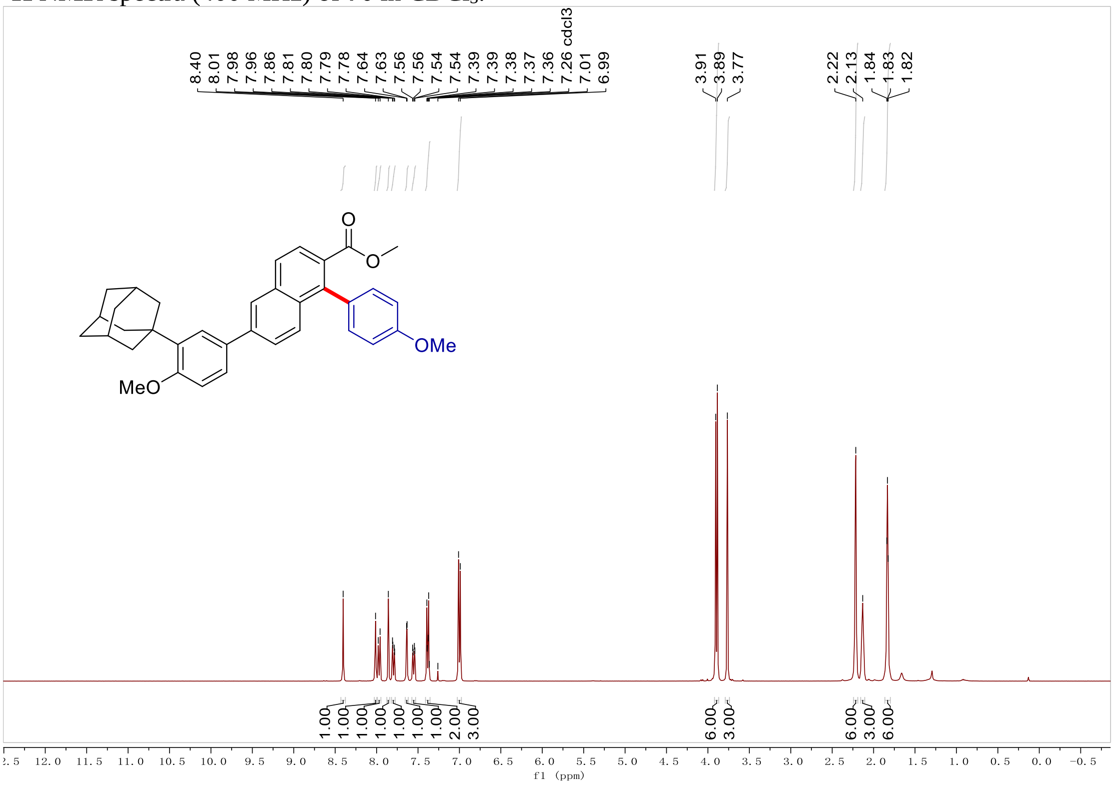

[13]**C NMR** spectra (101 MHz) of **70** in $CDCl_3$.

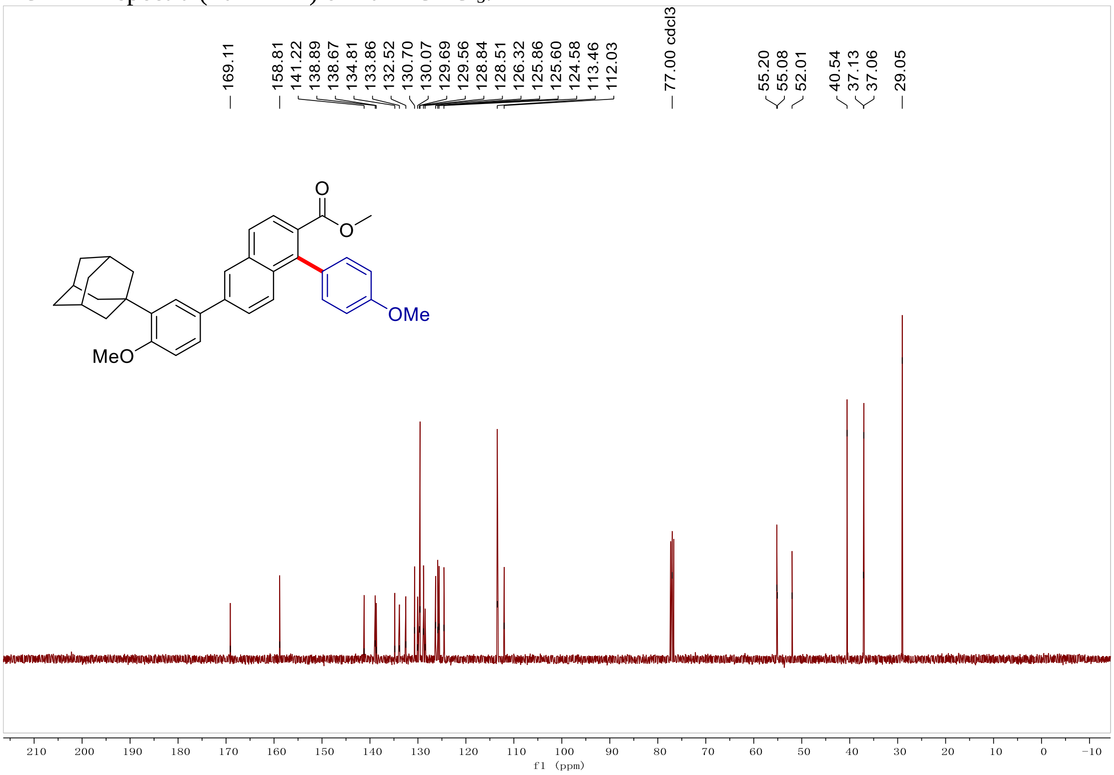

[1]**H NMR** spectra (400 MHz) of **71** in $CDCl_3$.

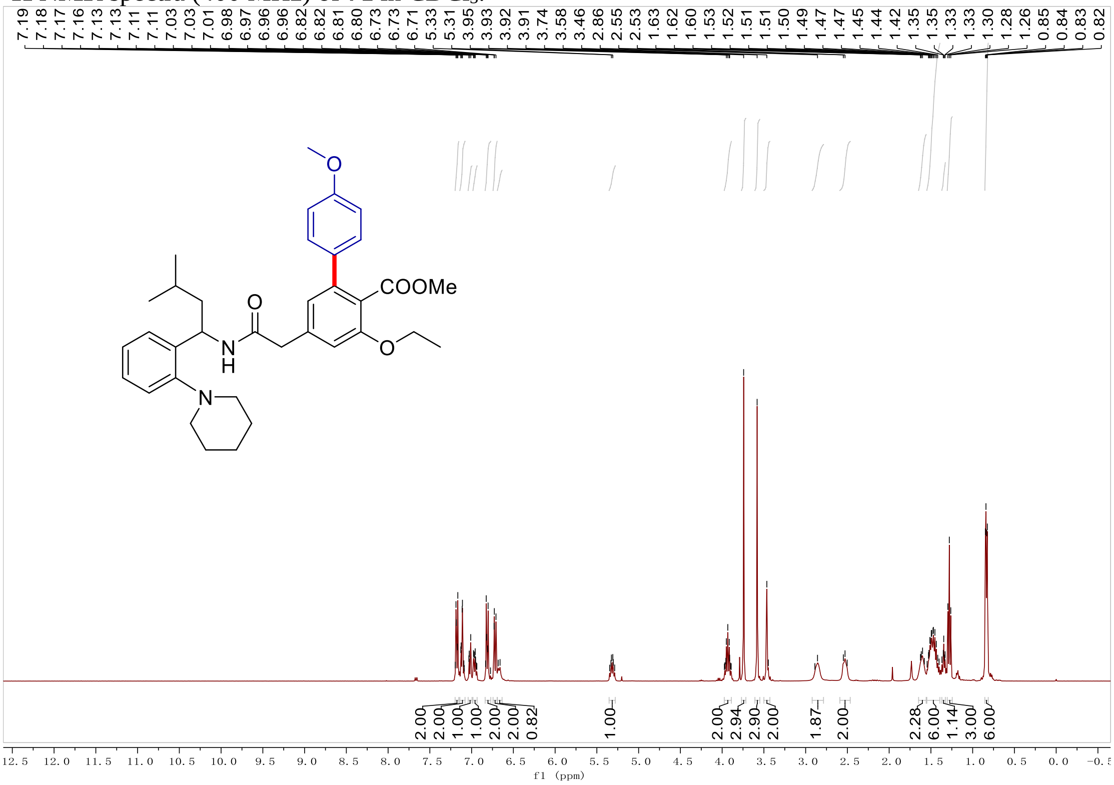

[13]**C NMR** spectra (101 MHz) of **71** in $CDCl_3$.

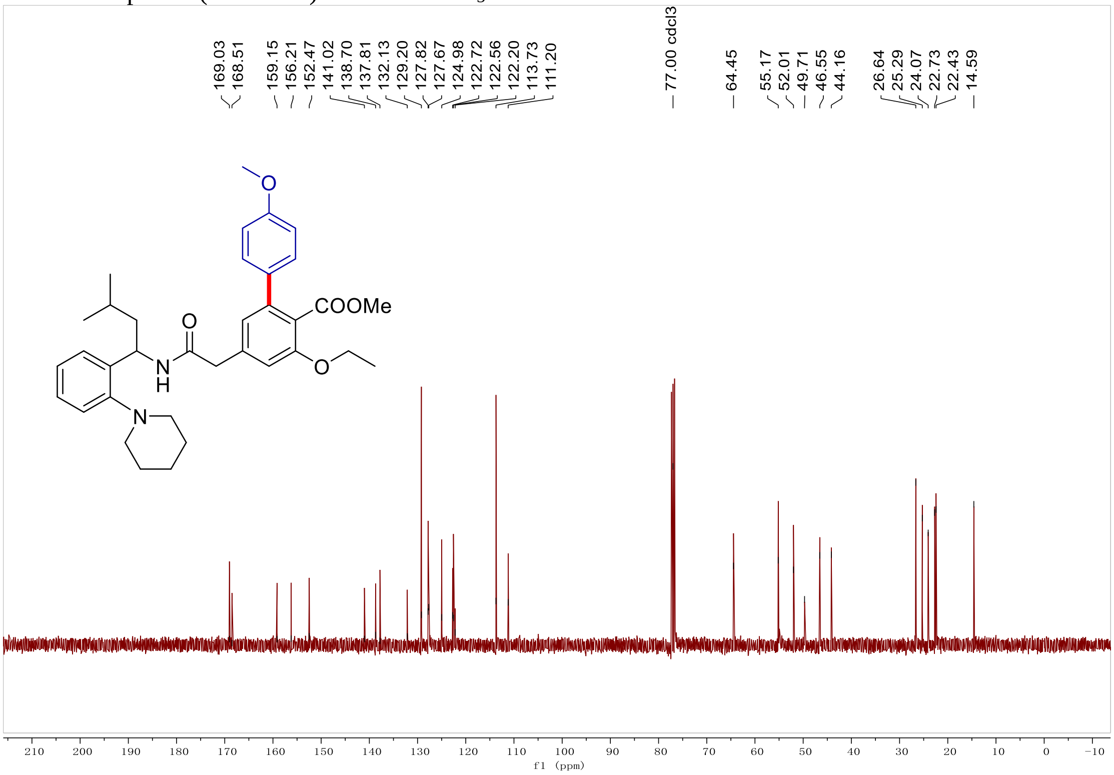

^{1}H NMR spectra (400 MHz) of **72** in $CDCl_3$.

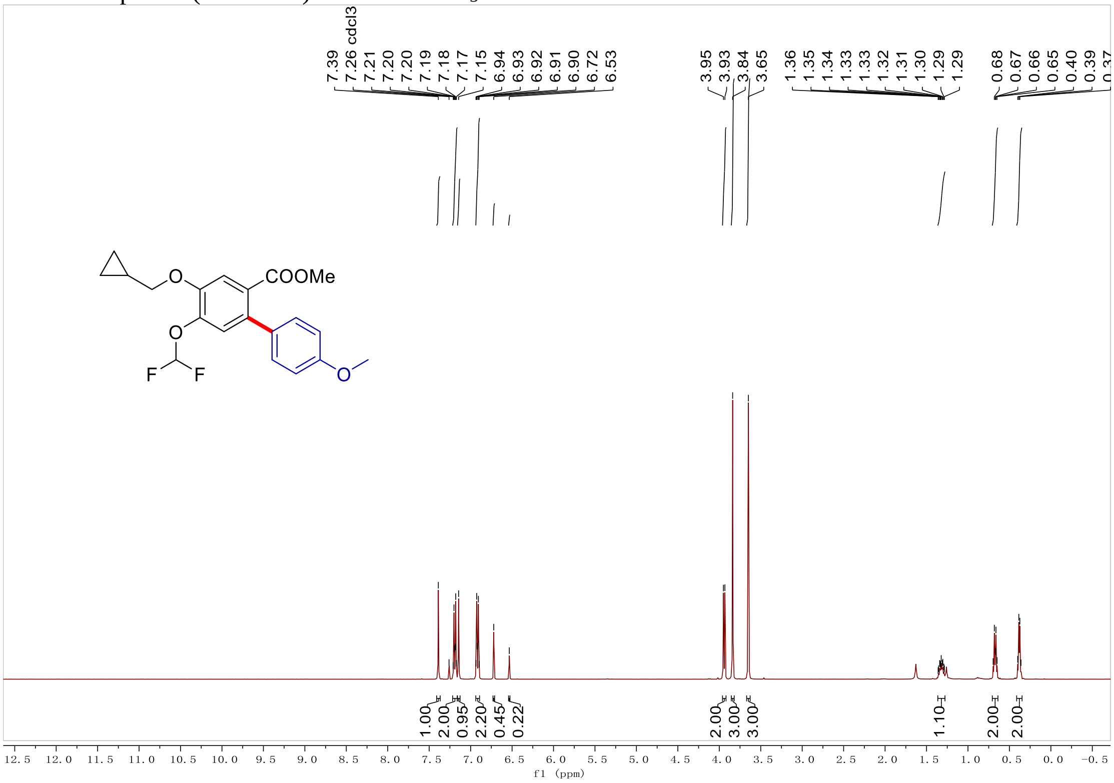

^{13}C NMR spectra (101 MHz) of **72** in $CDCl_3$.

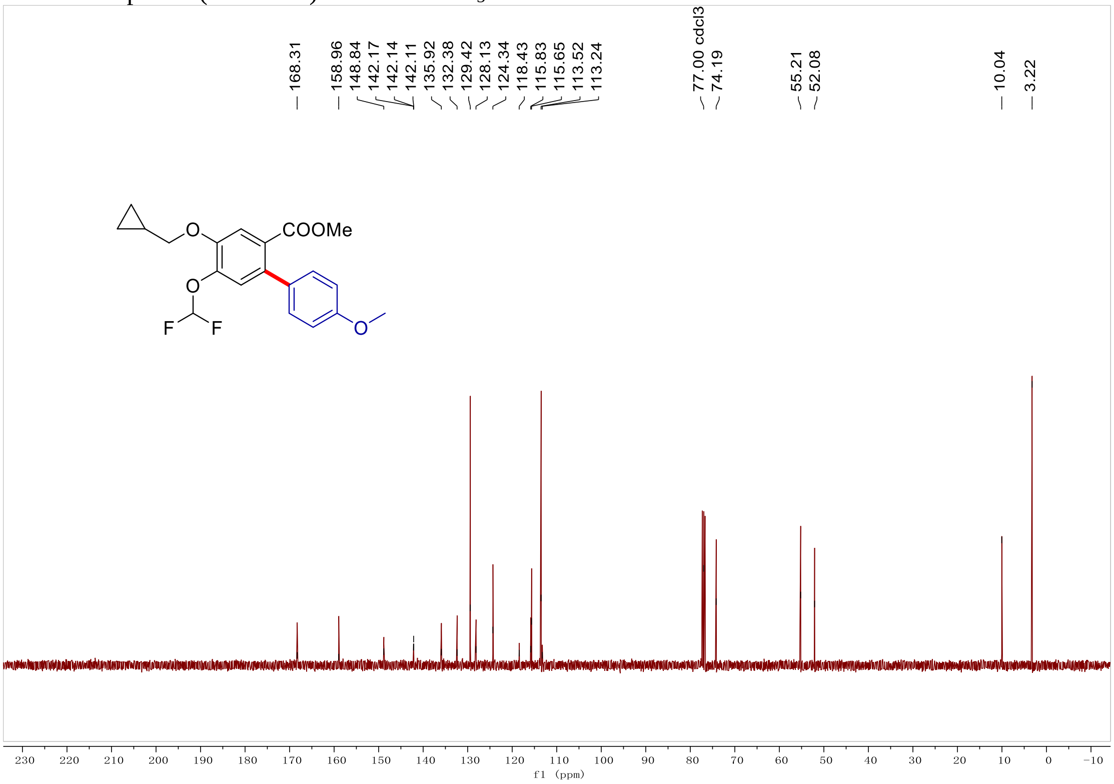

^{1}H NMR spectra (400 MHz) of **73** in $CDCl_3$.

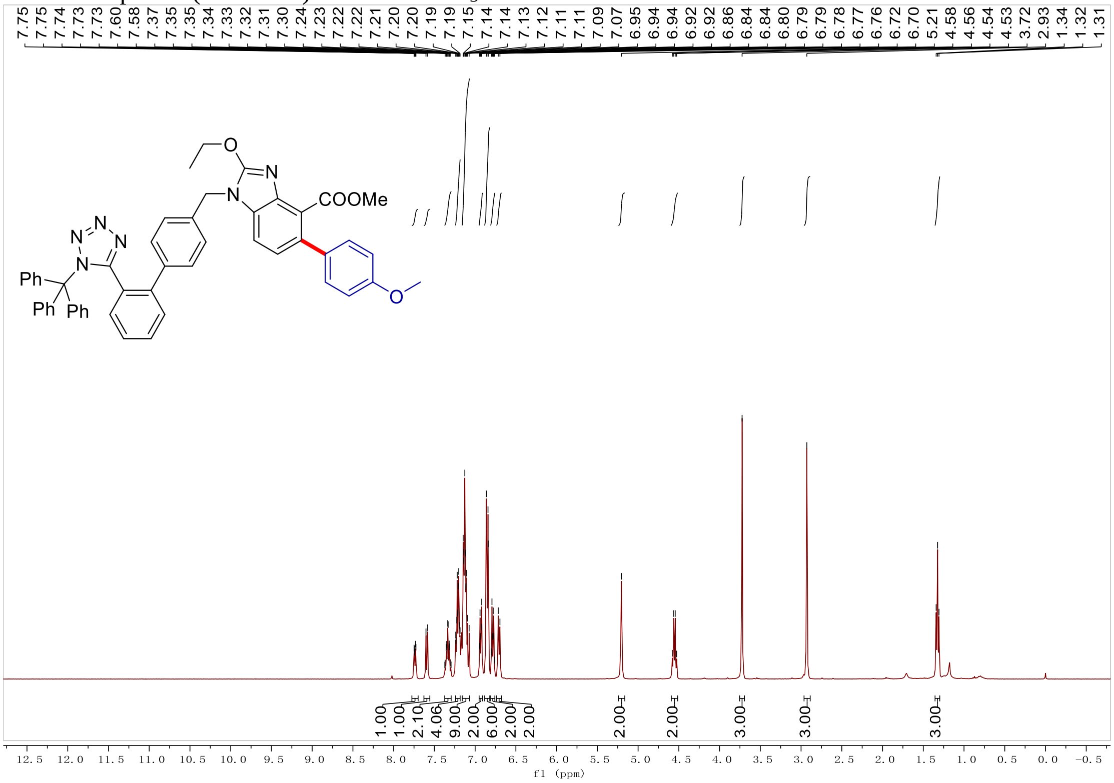

^{13}C NMR spectra (101 MHz) of **73** in $CDCl_3$.

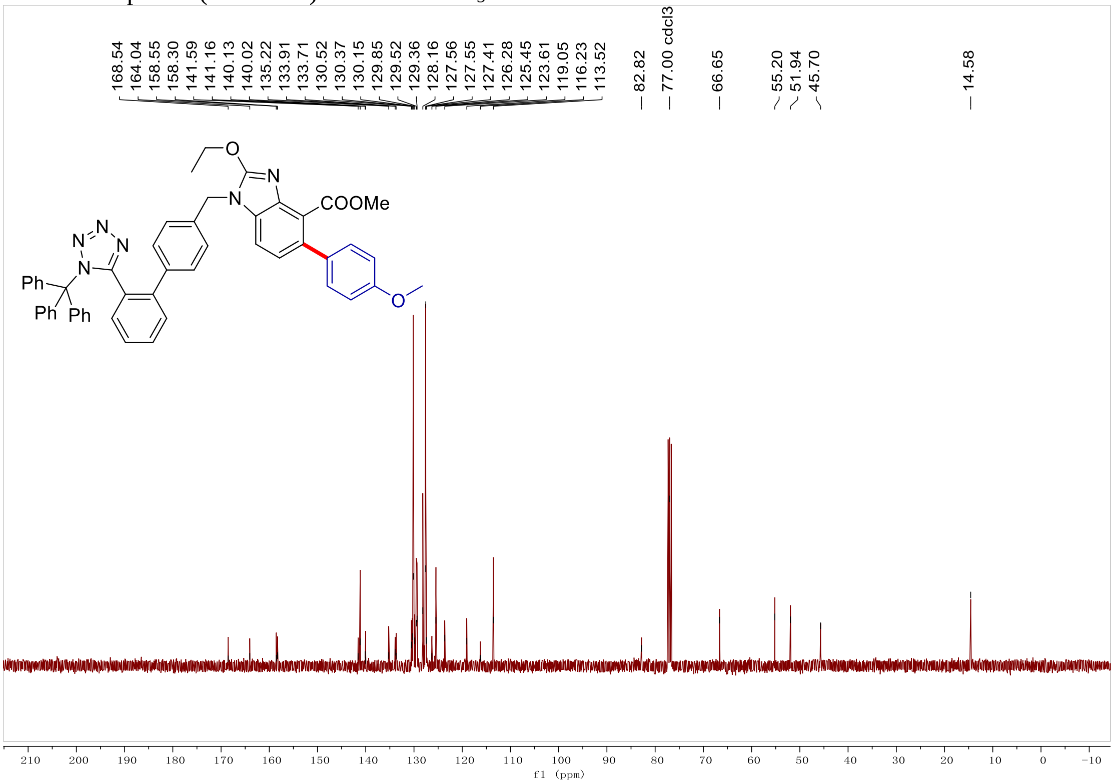

[1]**H NMR** spectra (400 MHz) of **74** in $CDCl_3$.

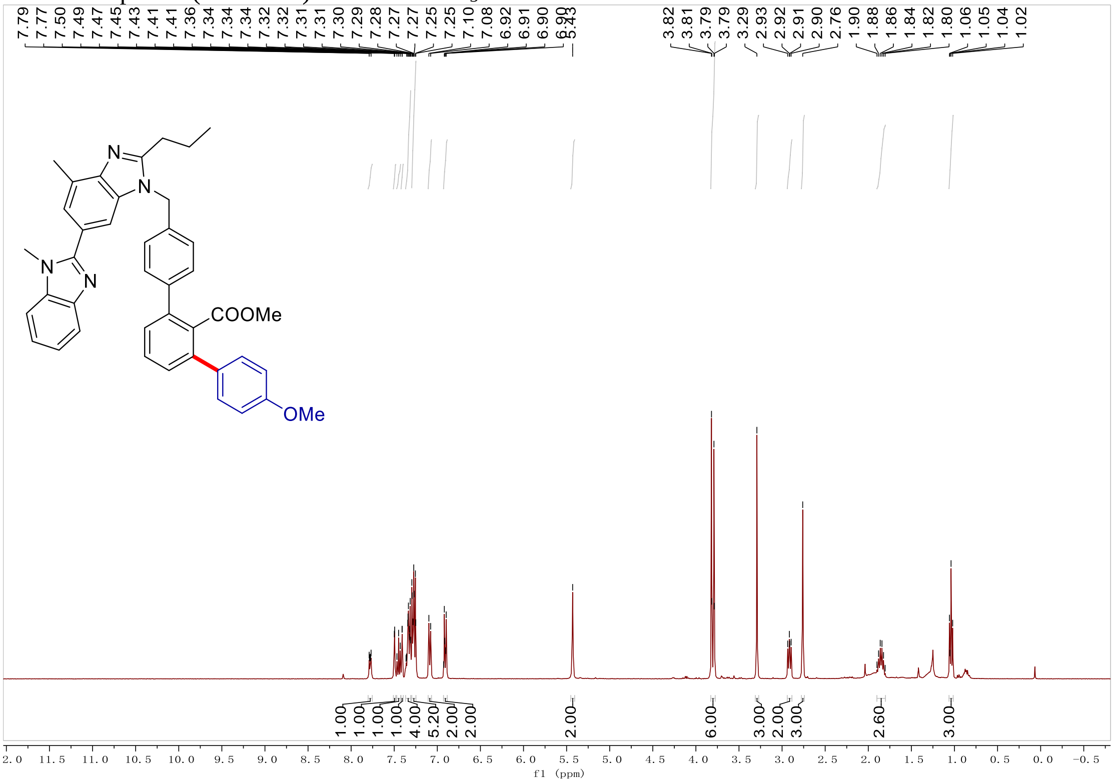

[13]**C NMR** spectra (101 MHz) of **74** in $CDCl_3$.

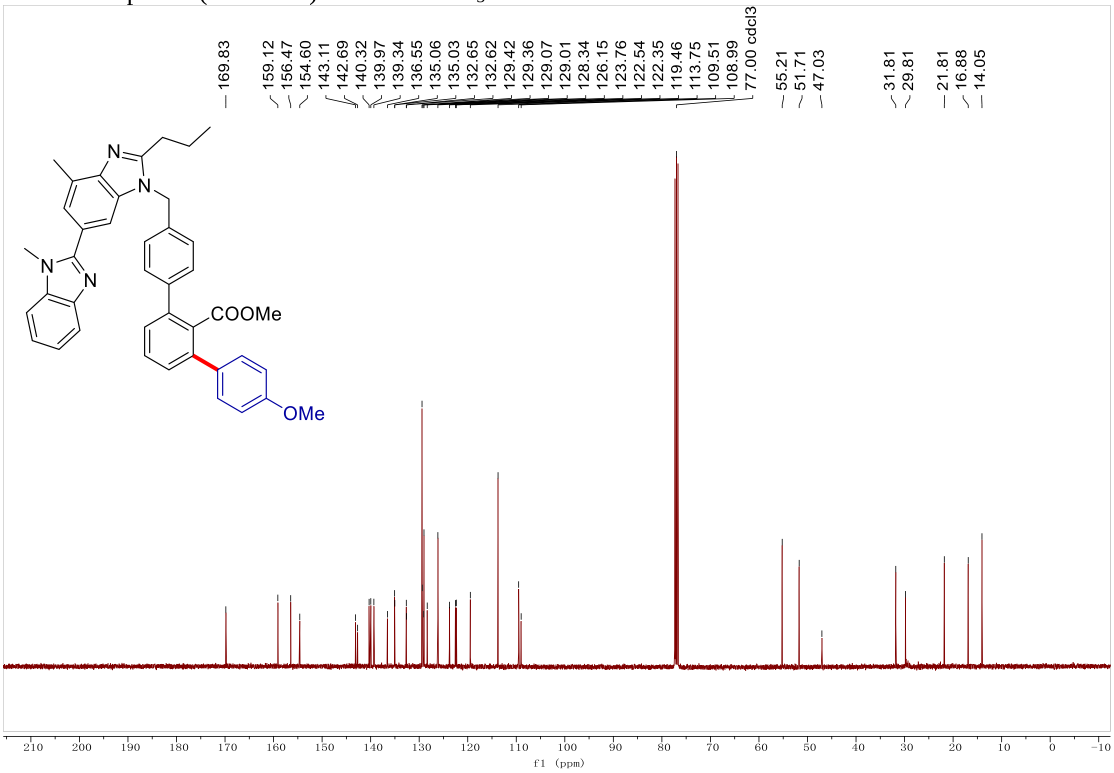

[1]**H NMR** spectra (400 MHz) of **75** in $CDCl_3$.

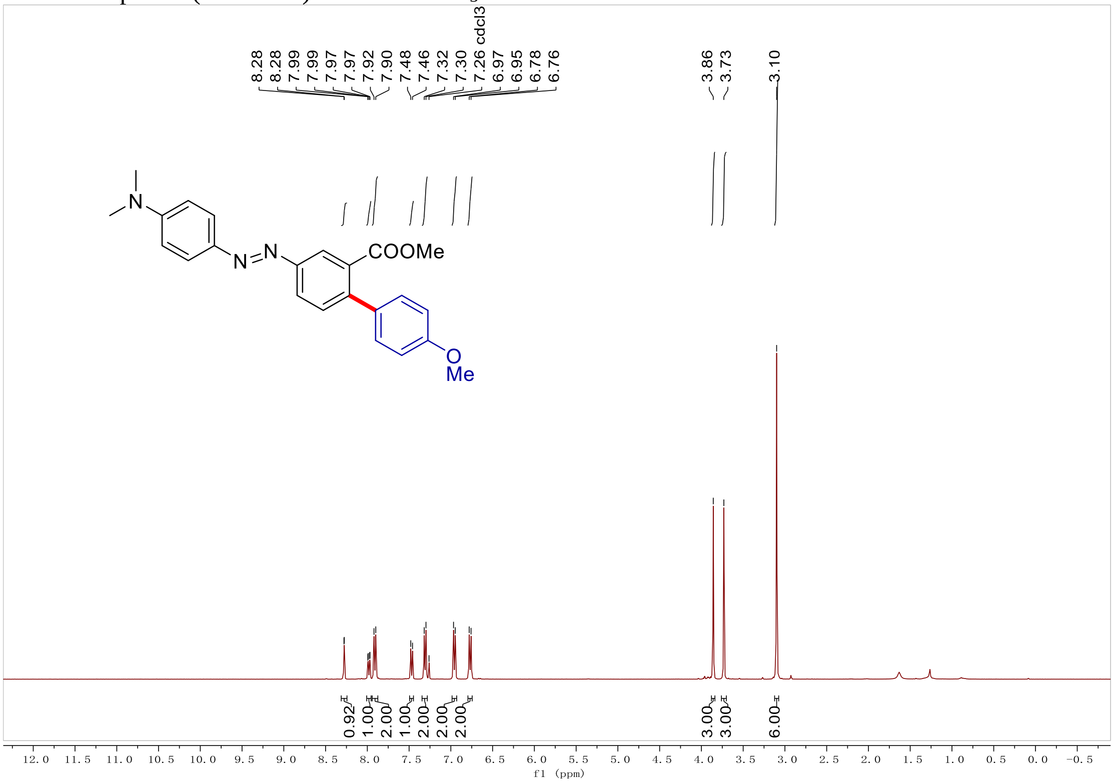

[13]**C NMR** spectra (101 MHz) of **75** in $CDCl_3$.

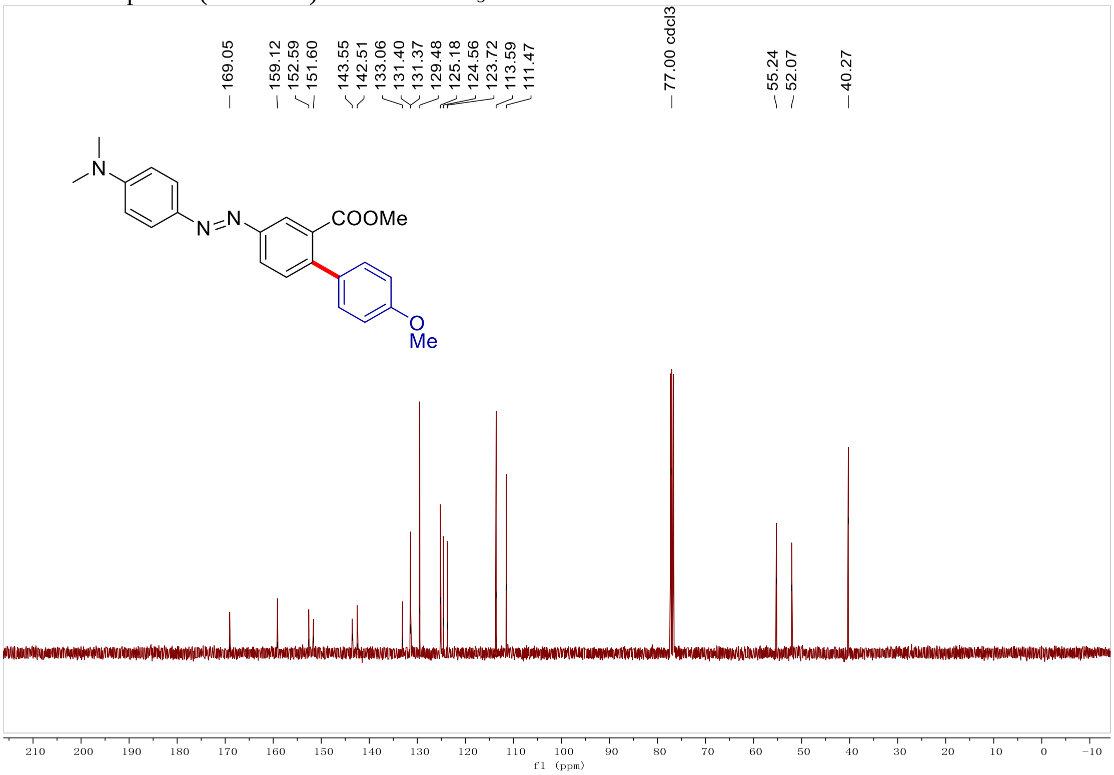

[1]**H NMR** spectra (400 MHz) of **76** in $CDCl_3$.

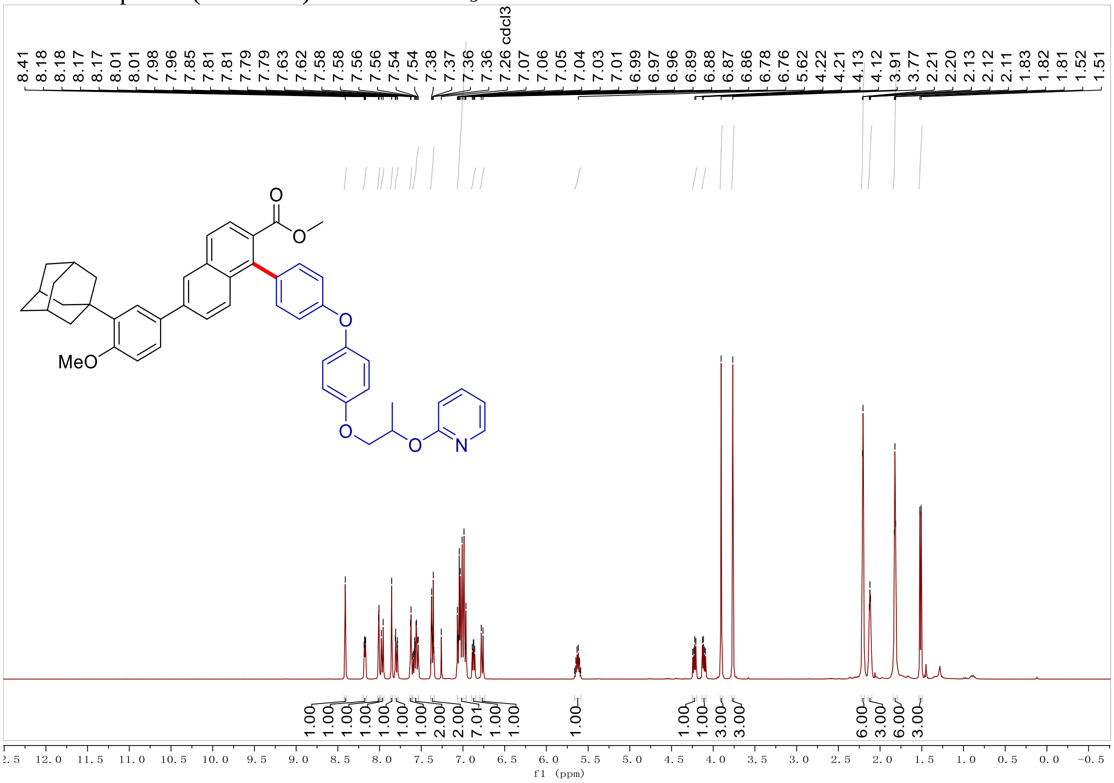

[13]**C NMR** spectra (101 MHz) of **76** in $CDCl_3$.

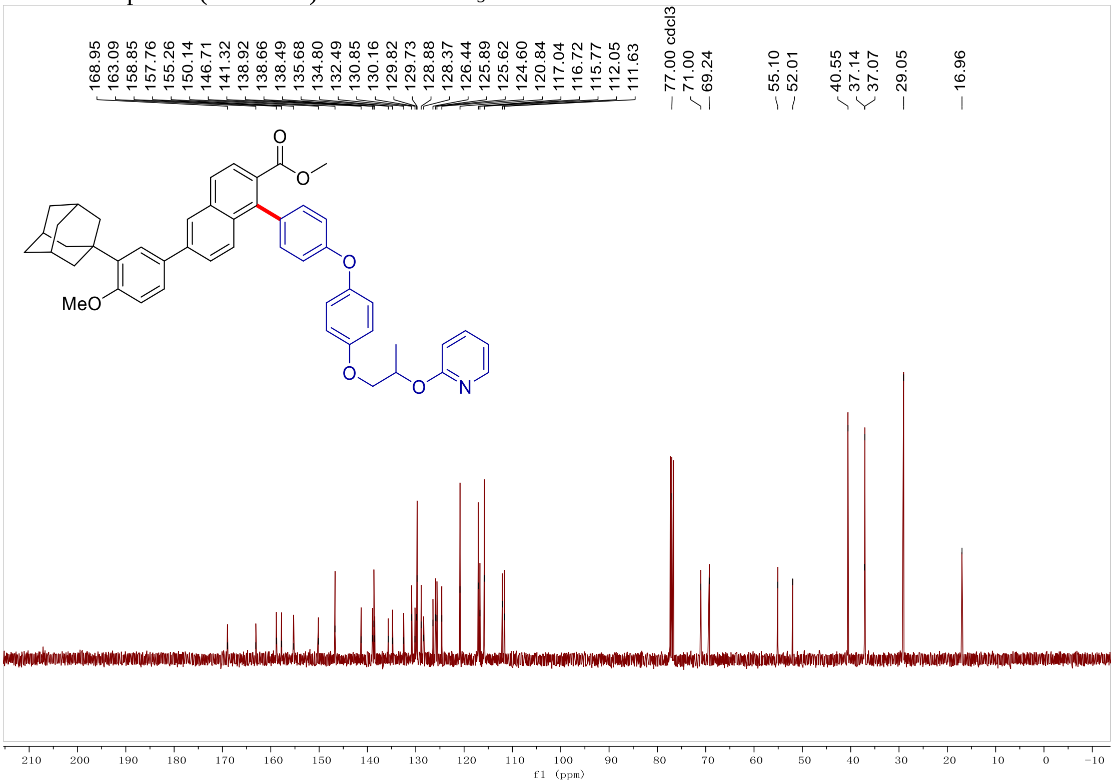

^{1}H NMR spectra (400 MHz) of **77** in $CDCl_3$.

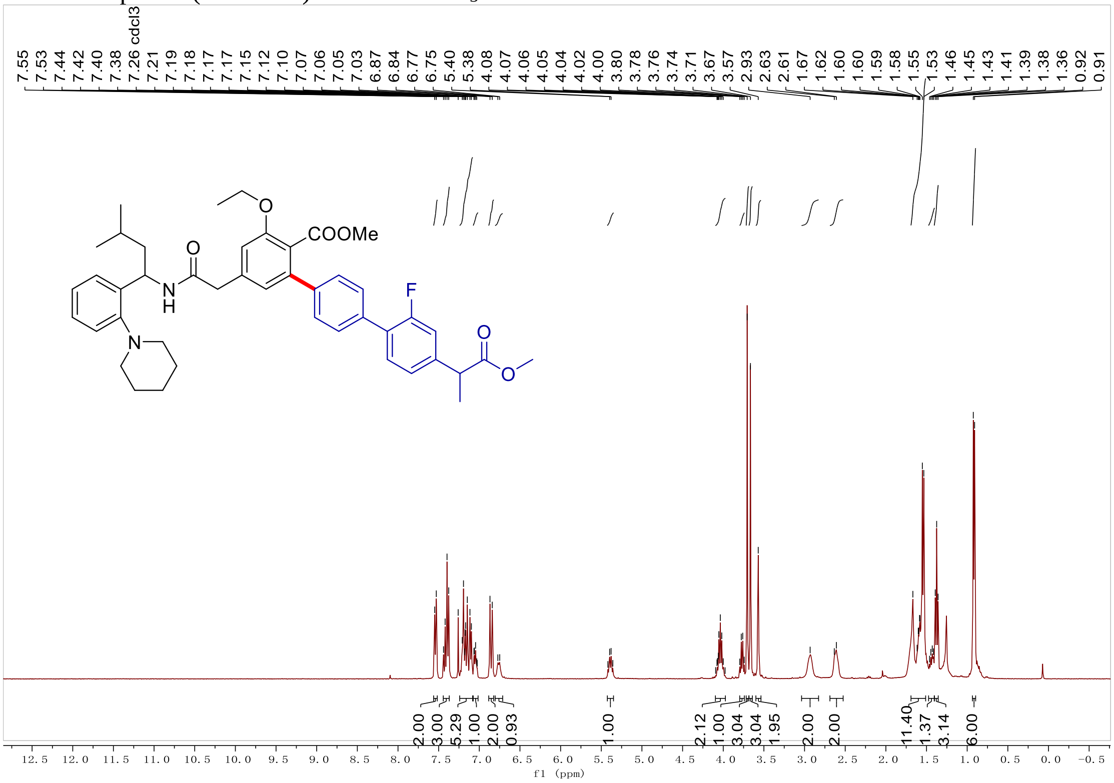

^{13}C NMR spectra (101 MHz) of **77** in $CDCl_3$.

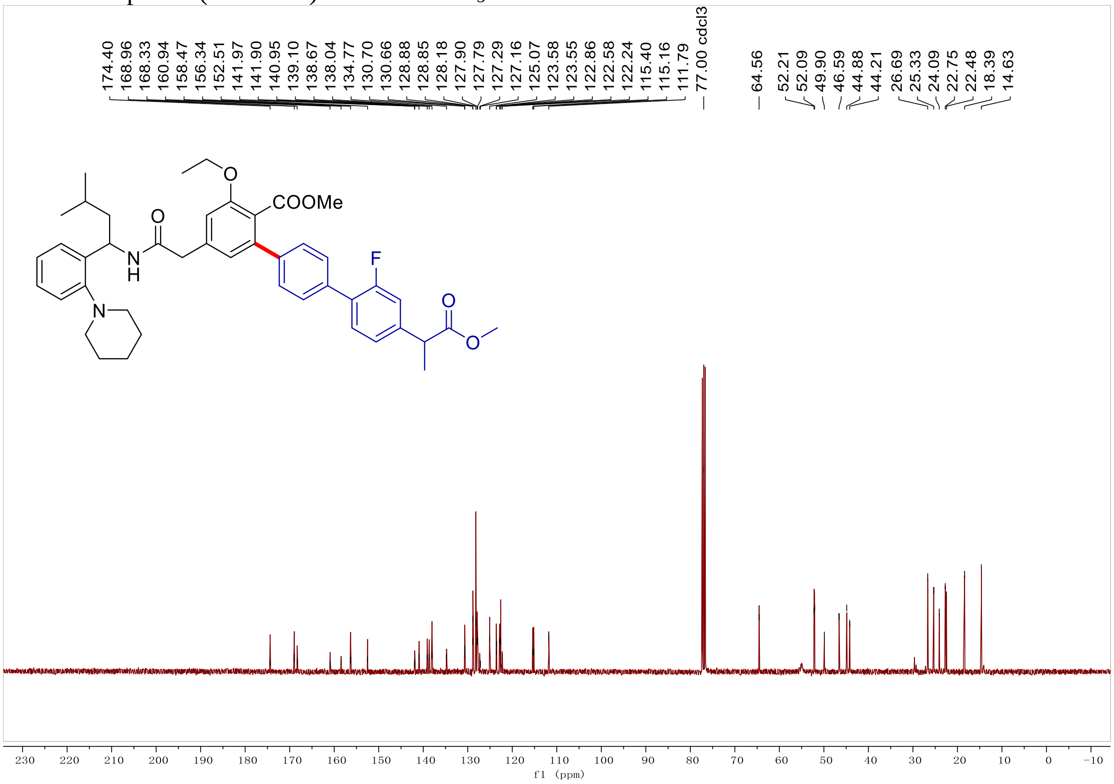

^{1}H NMR spectra (400 MHz) of **78** in $CDCl_3$.

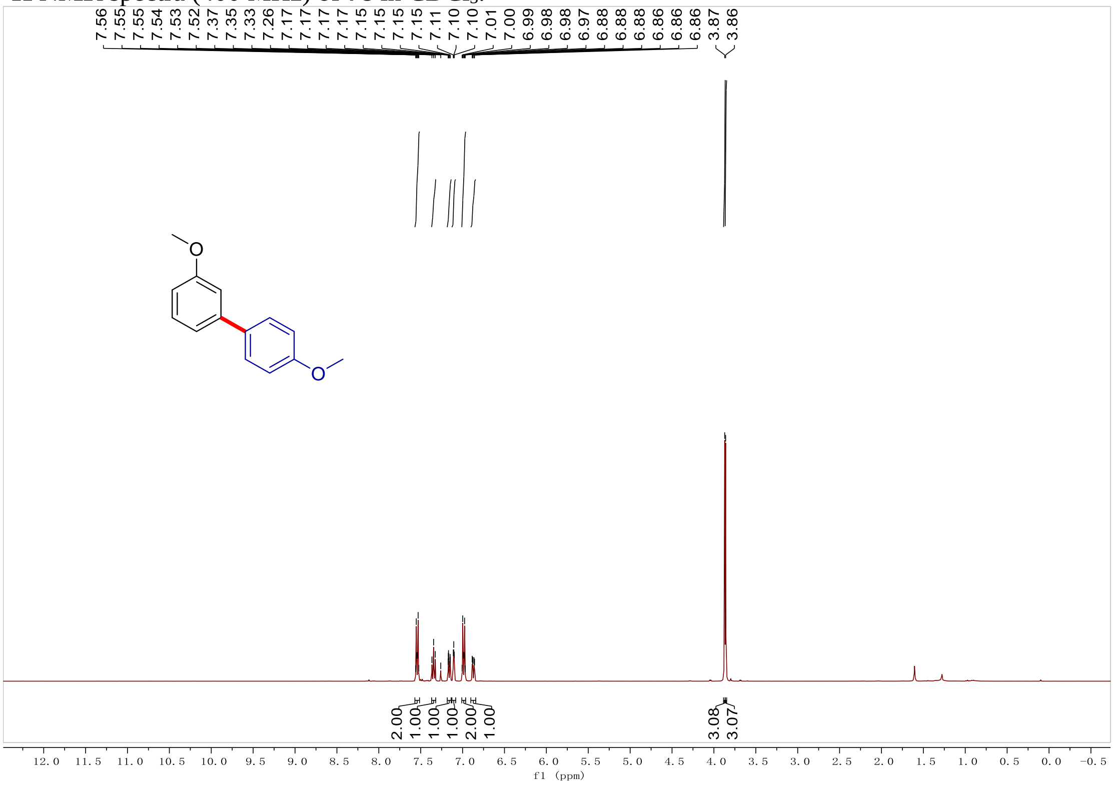

^{13}C NMR spectra (101 MHz) of **78** in $CDCl_3$.

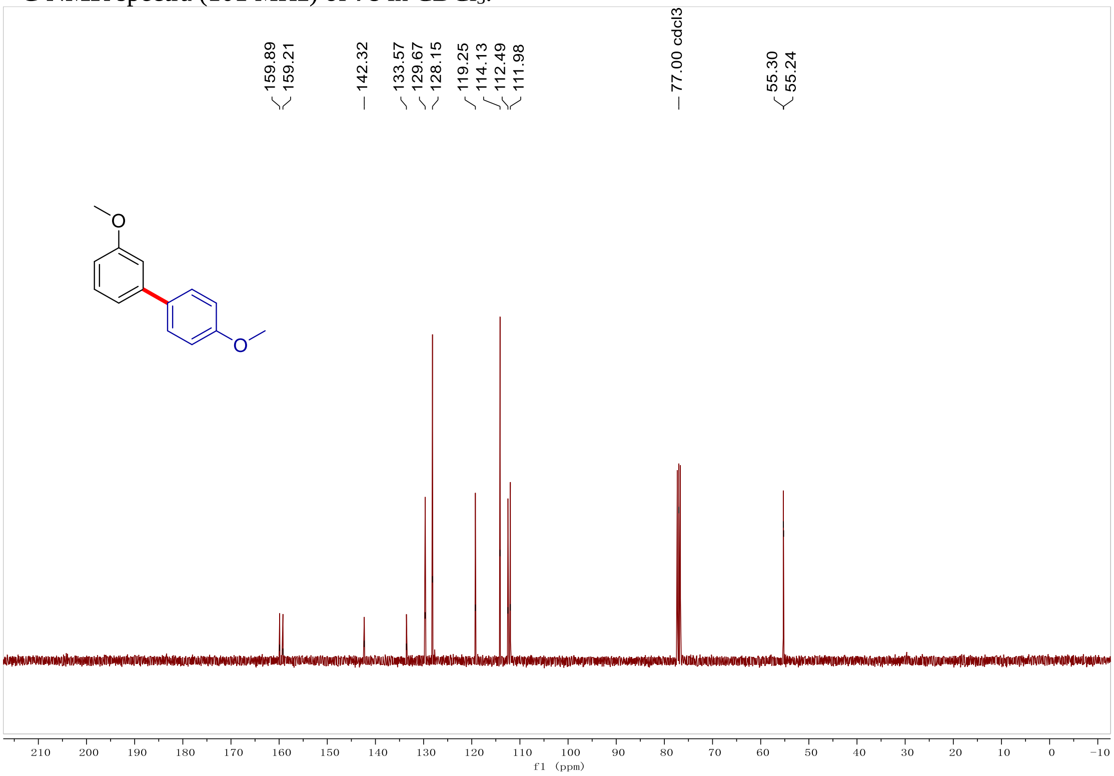

^{1}H NMR spectra (400 MHz) of **79** in CDCl3.

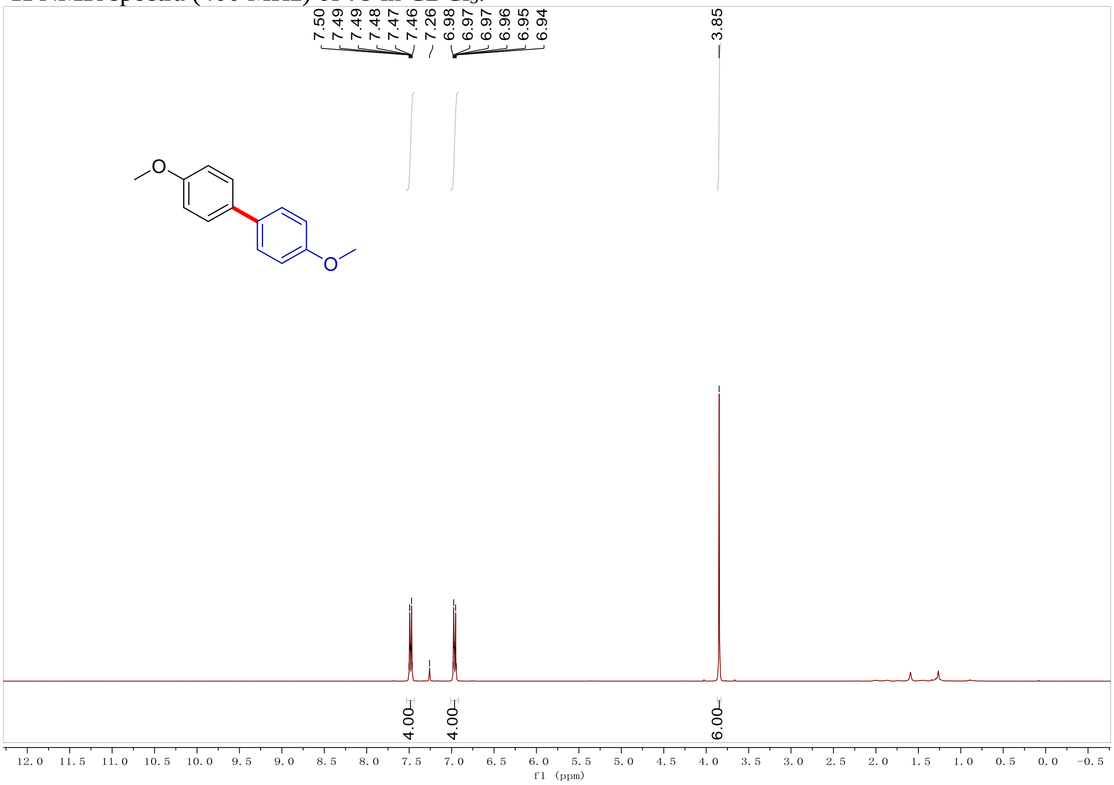

^{13}C NMR spectra (101 MHz) of **79** in CDCl3.

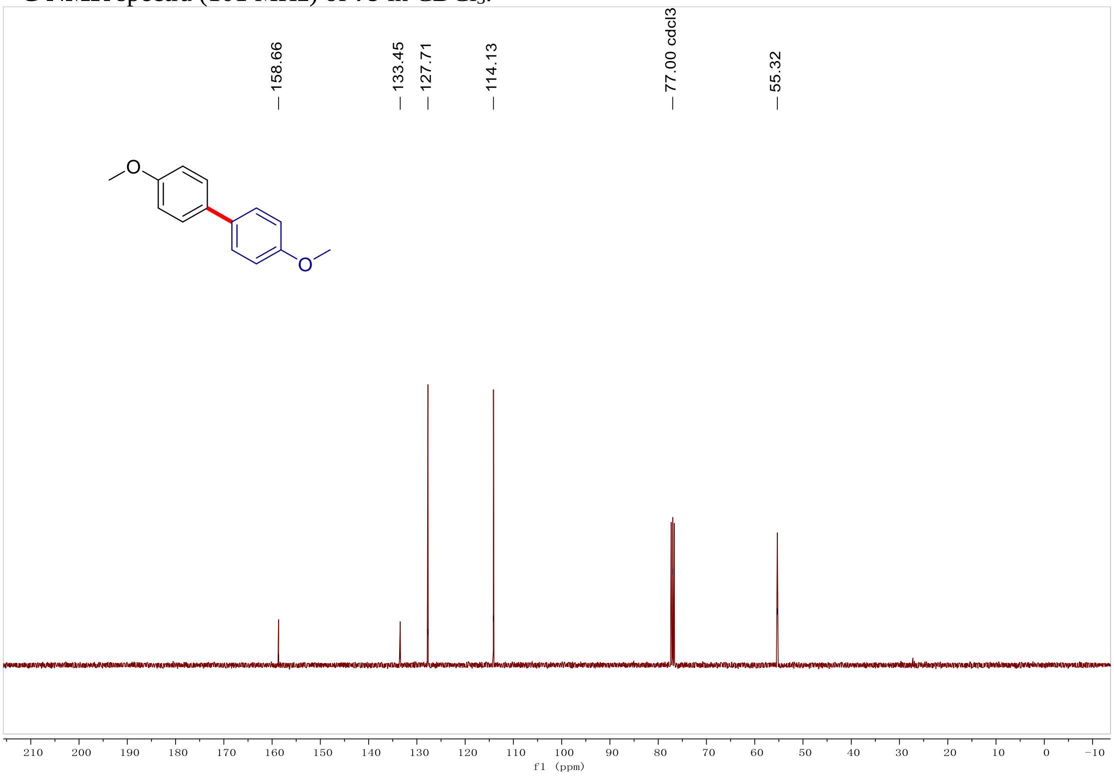

^{1}H NMR spectra (400 MHz) of **80** in $CDCl_3$.

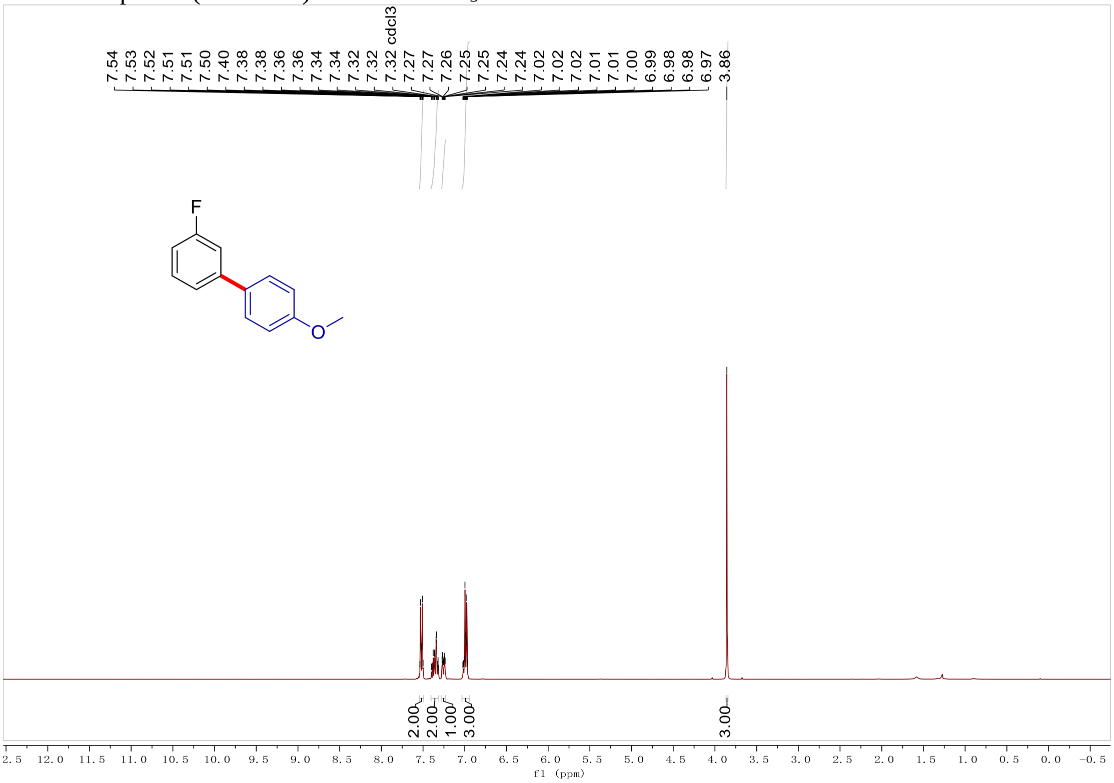

^{13}C NMR spectra (101 MHz) of **80** in $CDCl_3$.

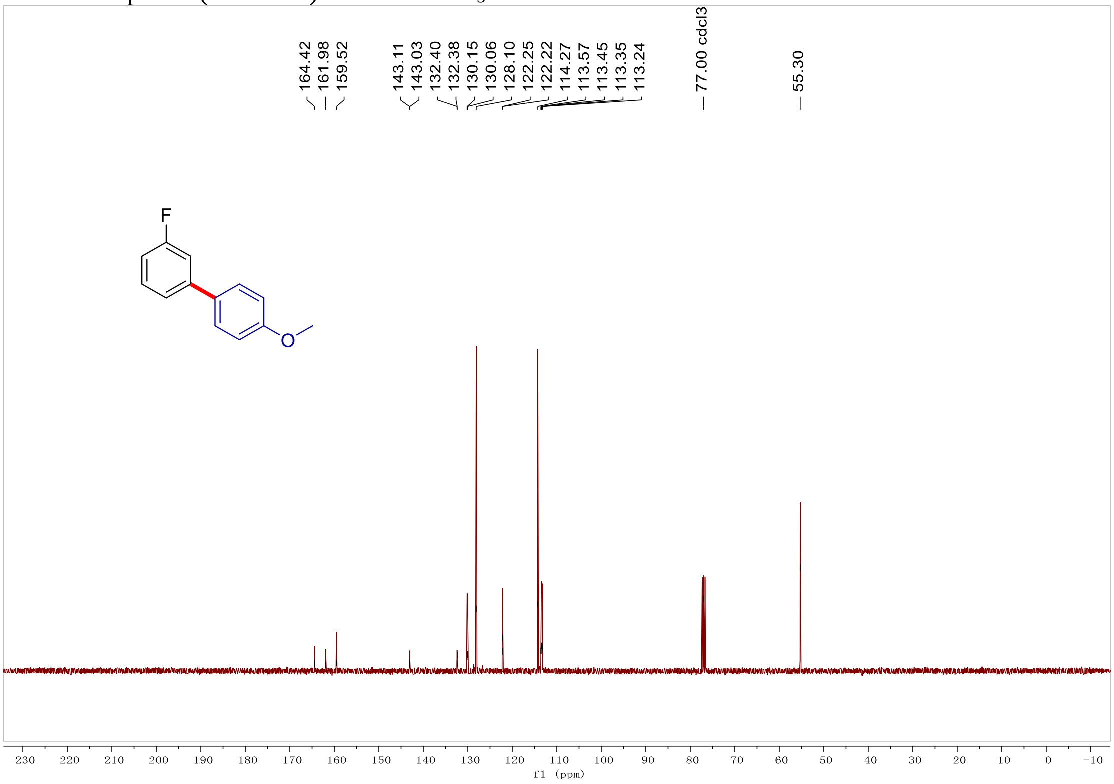

^{1}H NMR spectra (400 MHz) of **81** in $CDCl_3$.

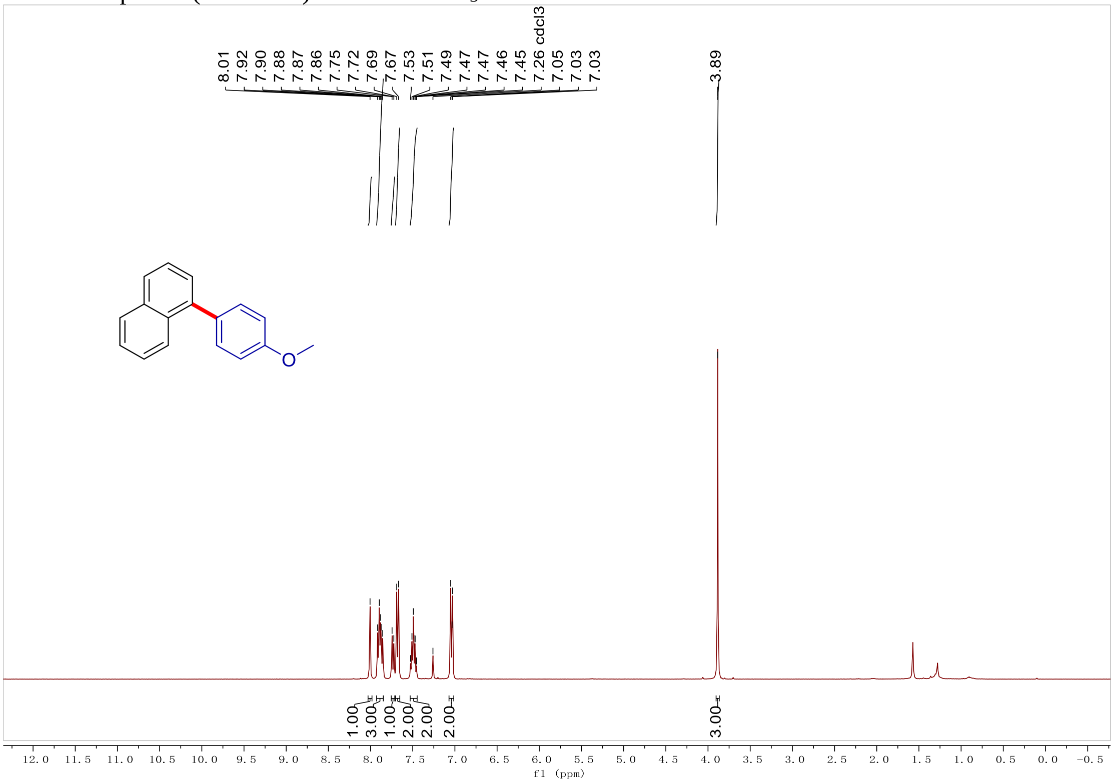

^{13}C NMR spectra (101 MHz) of **81** in $CDCl_3$.

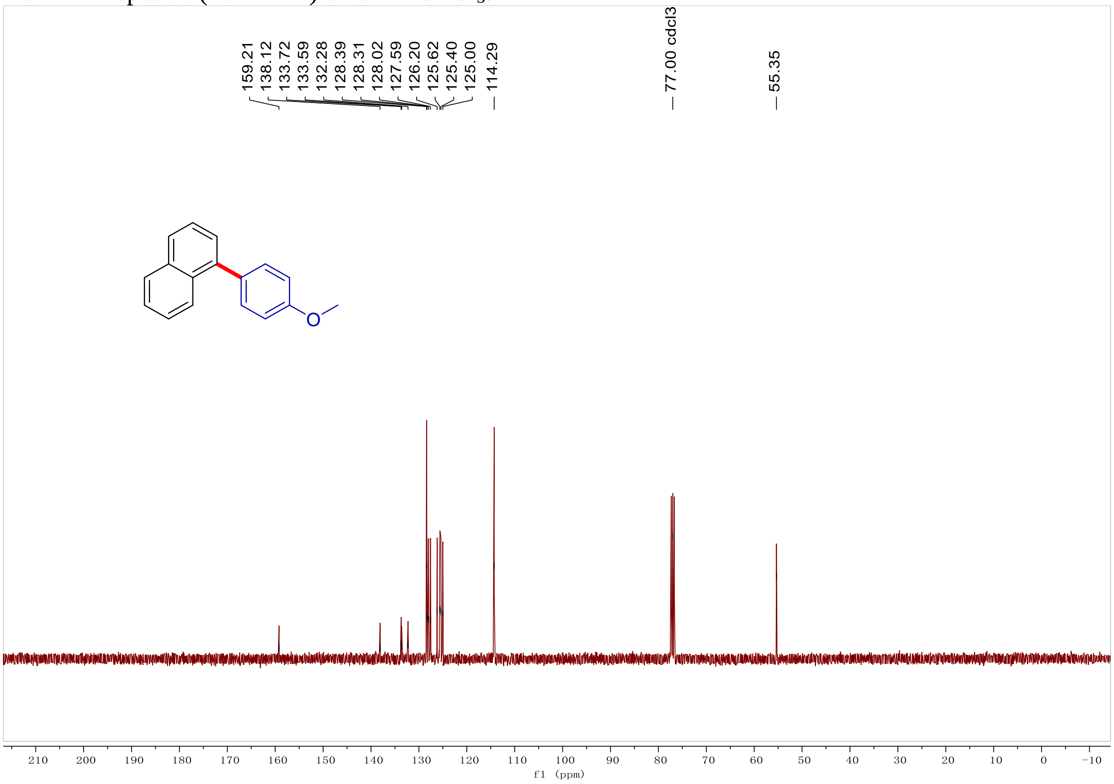

[1]**H NMR** spectra (400 MHz) of **82** in $CDCl_3$.

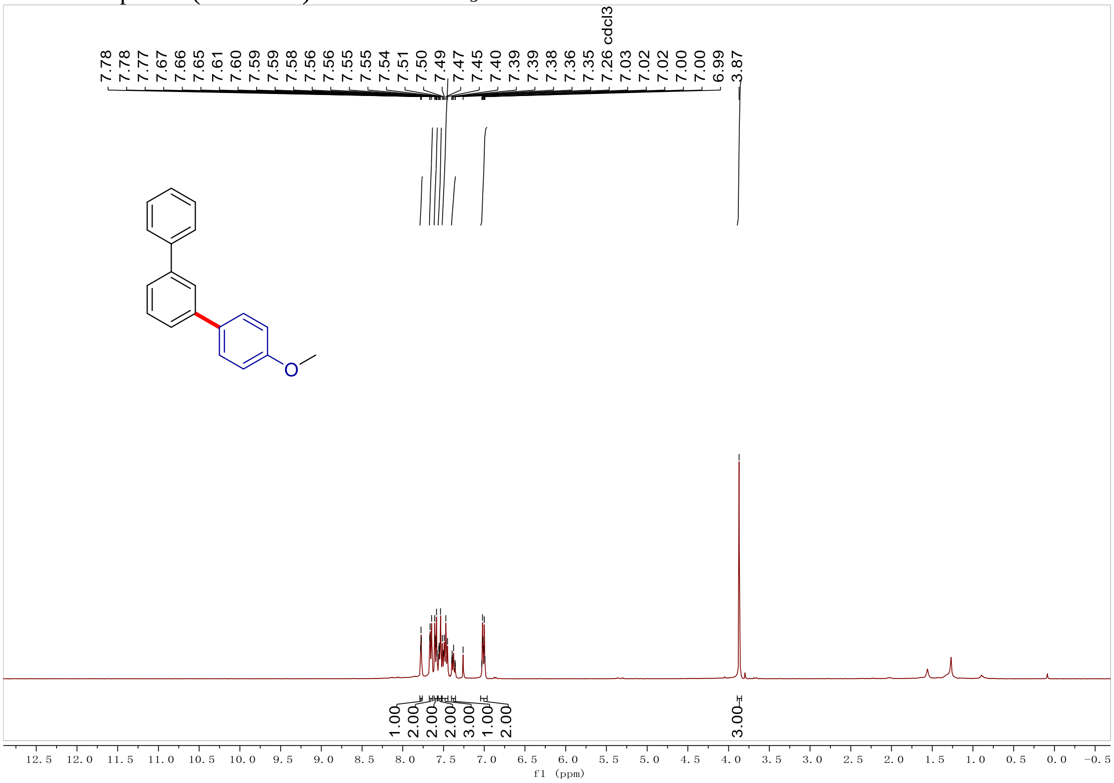

[13]**C NMR** spectra (101 MHz) of **82** in $CDCl_3$.

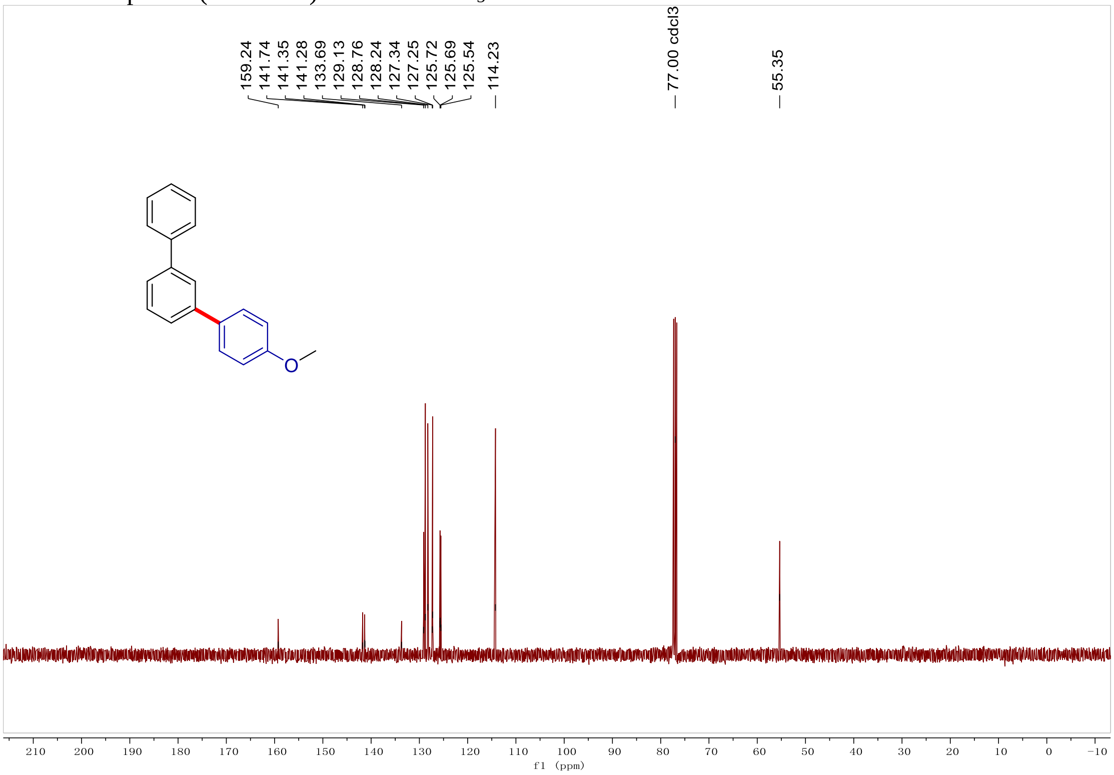

^{1}H NMR spectra (400 MHz) of **83** in $CDCl_3$.

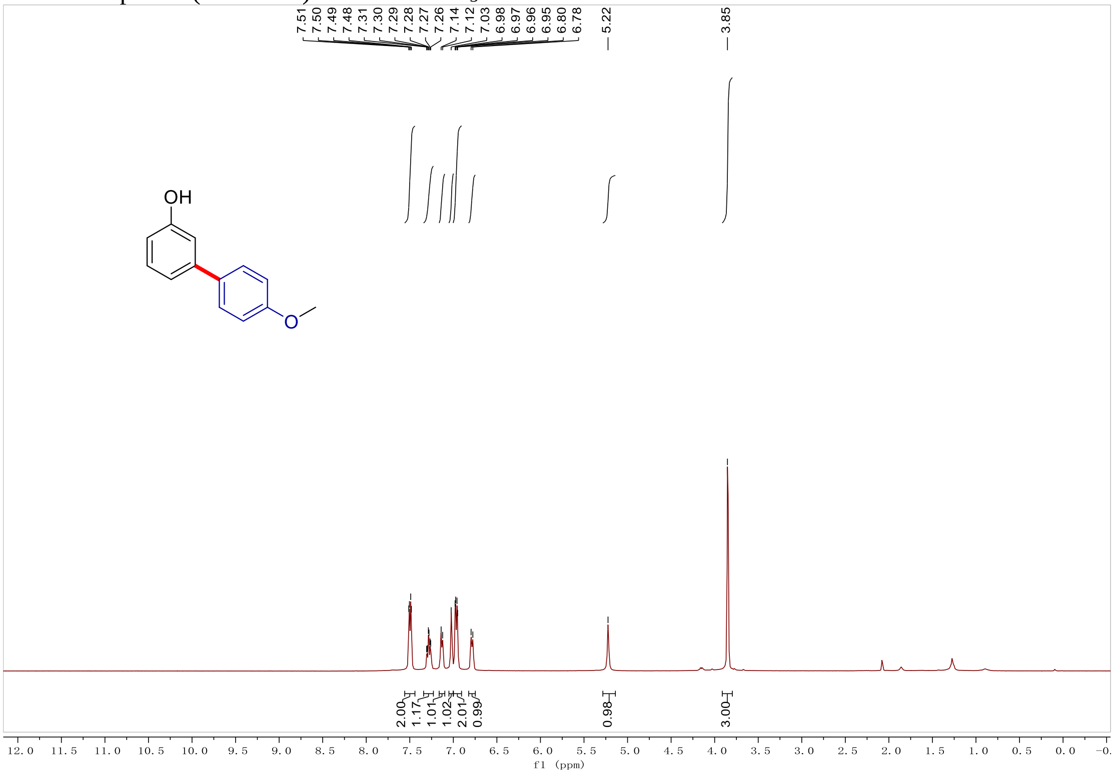

^{13}C NMR spectra (101 MHz) of **83** in $CDCl_3$.

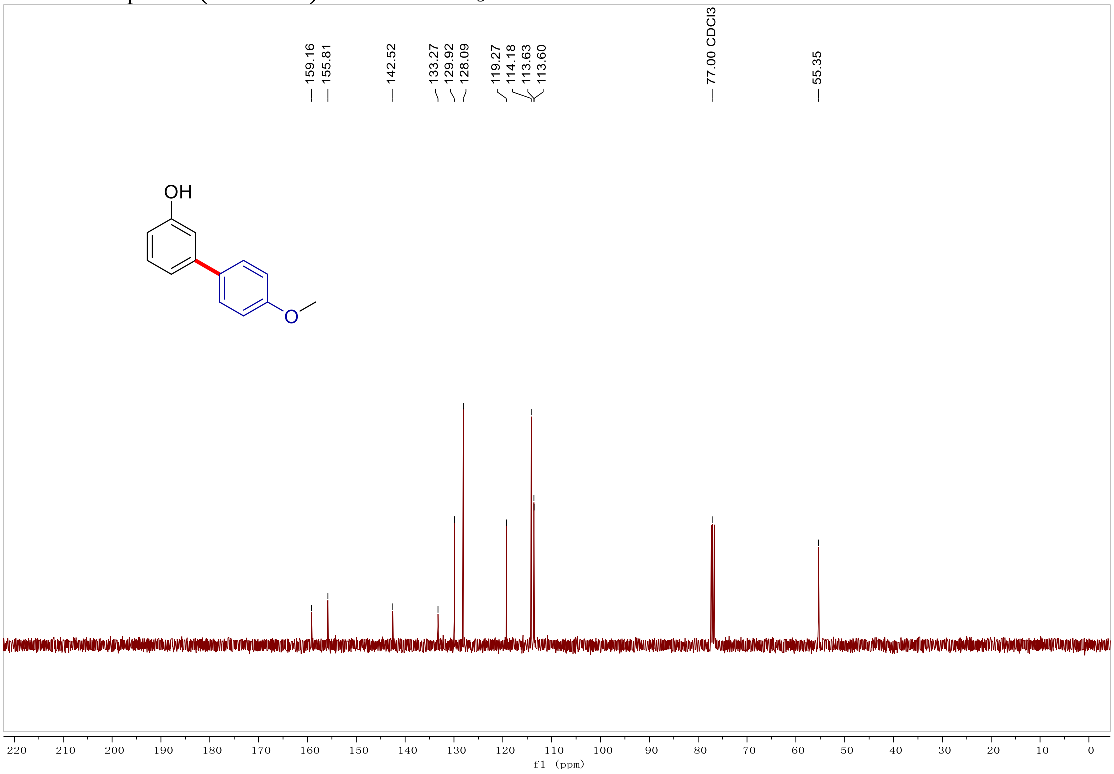

^{1}**H NMR** spectra (400 MHz) of **84** in $CDCl_3$.

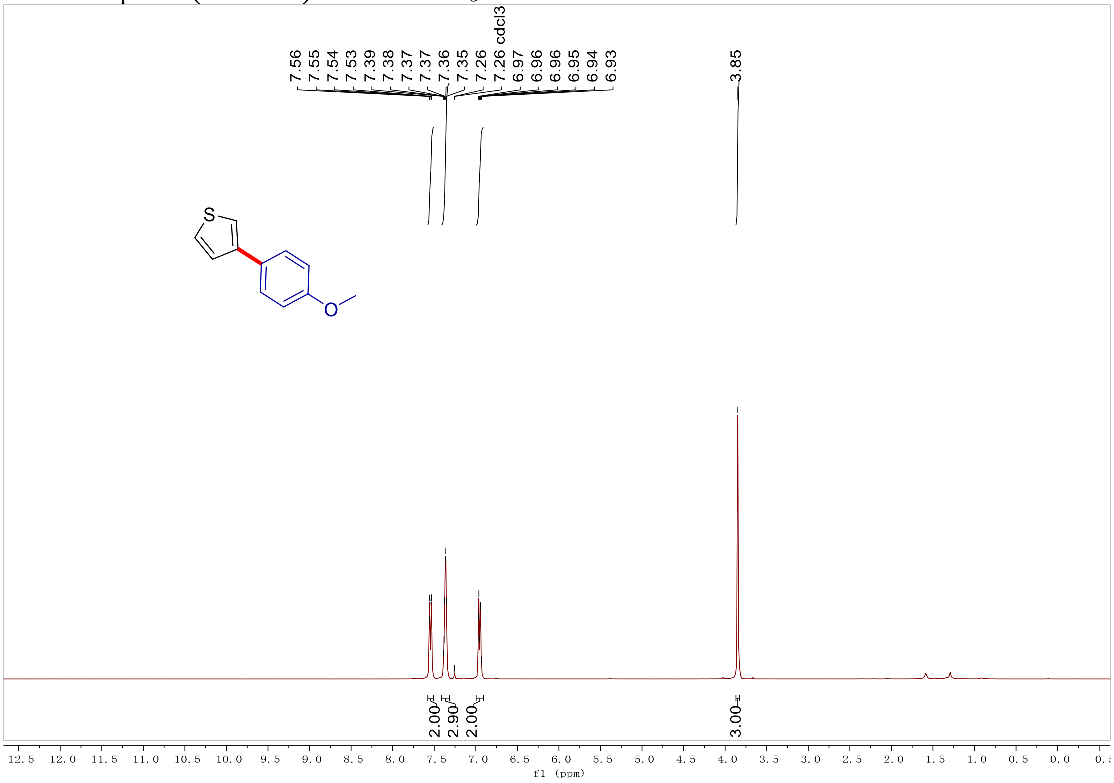

^{13}**C NMR** spectra (101 MHz) of **84** in $CDCl_3$.

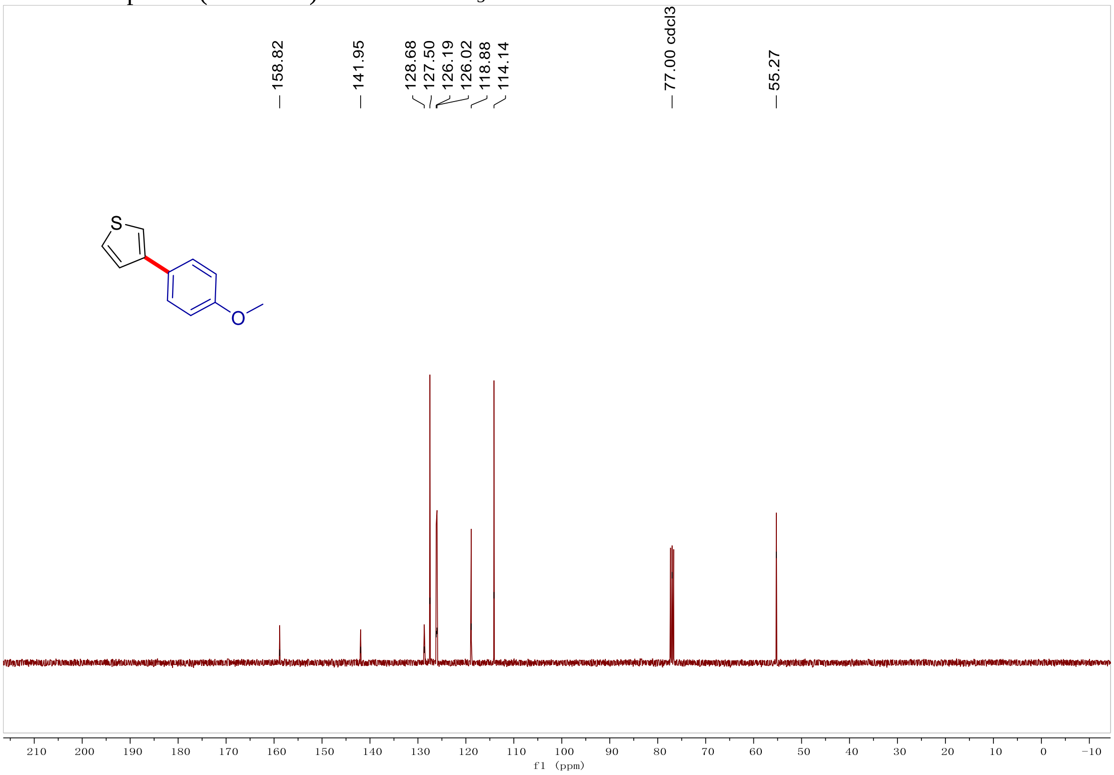

[1]**H NMR** spectra (400 MHz) of **85** in $CDCl_3$.

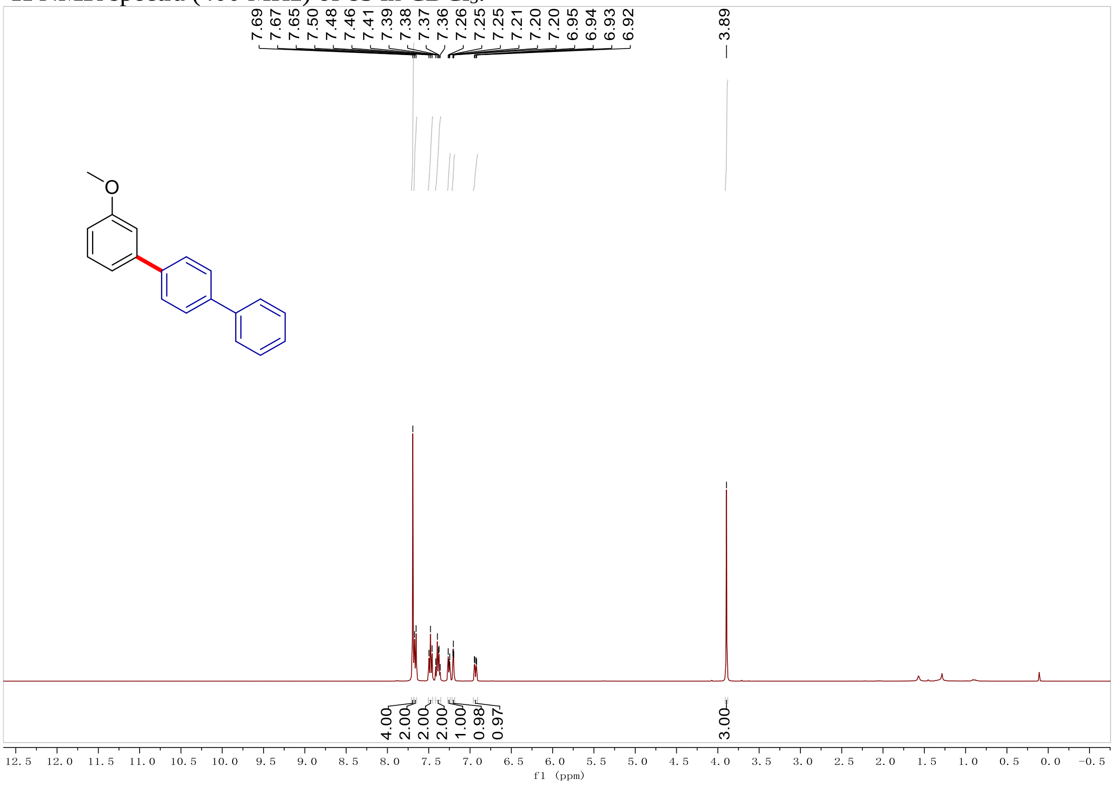

[13]**C NMR** spectra (101 MHz) of **85** in $CDCl_3$.

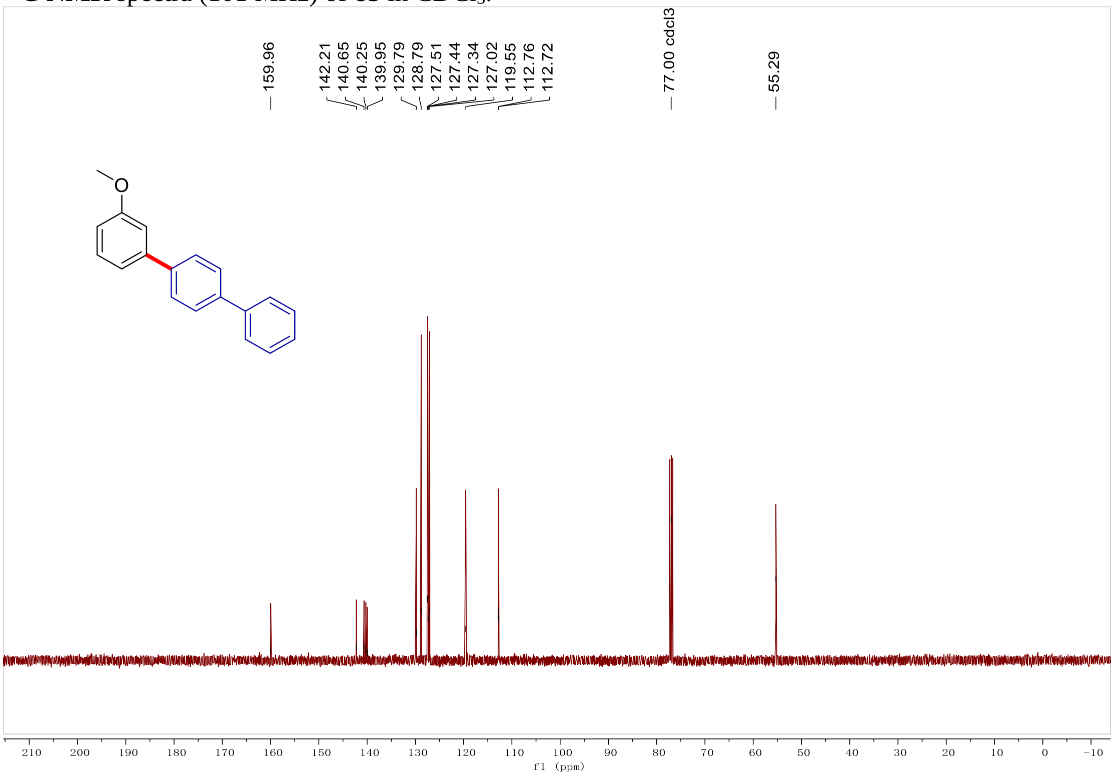

^{1}H NMR spectra (400 MHz) of **86** in $CDCl_3$.

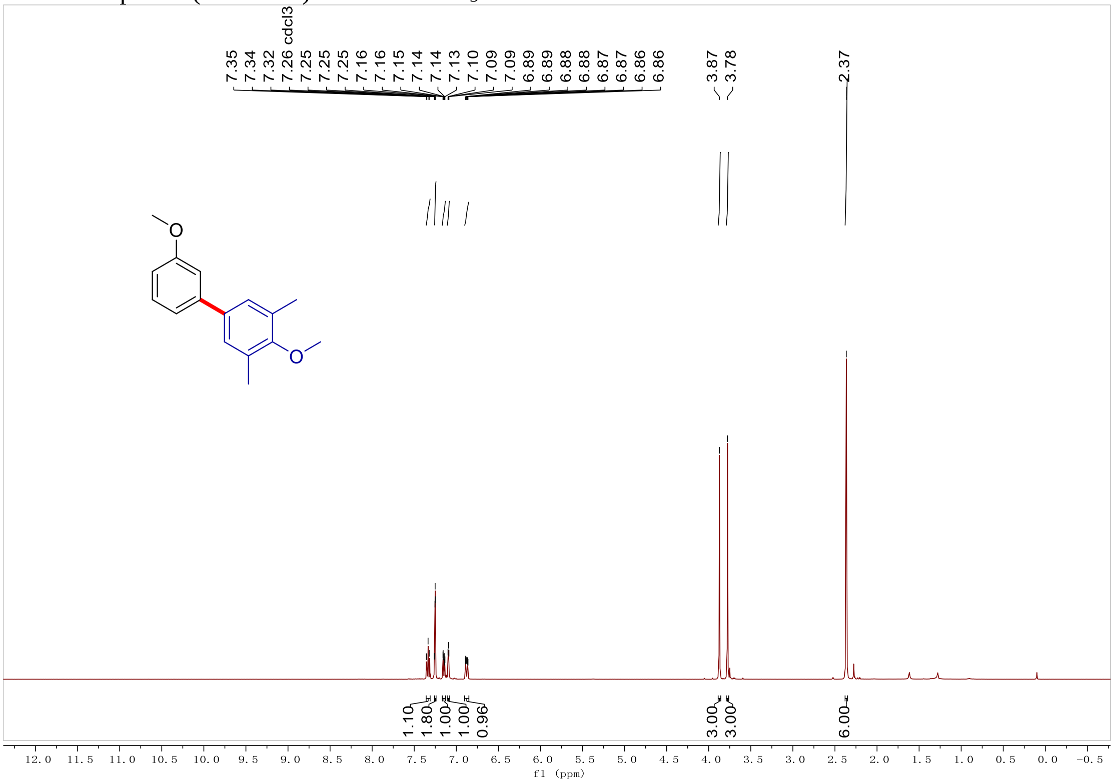

^{13}C NMR spectra (101 MHz) of **86** in $CDCl_3$.

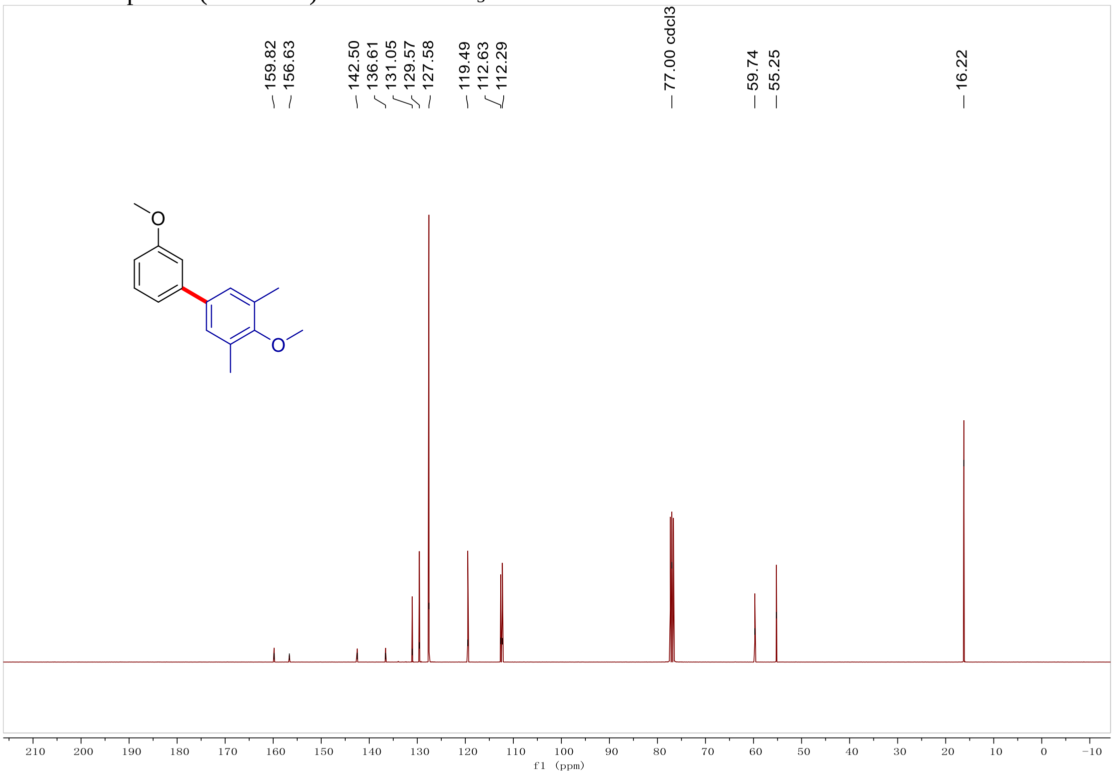

[1]**H NMR** spectra (400 MHz) of **87** in $CDCl_3$.

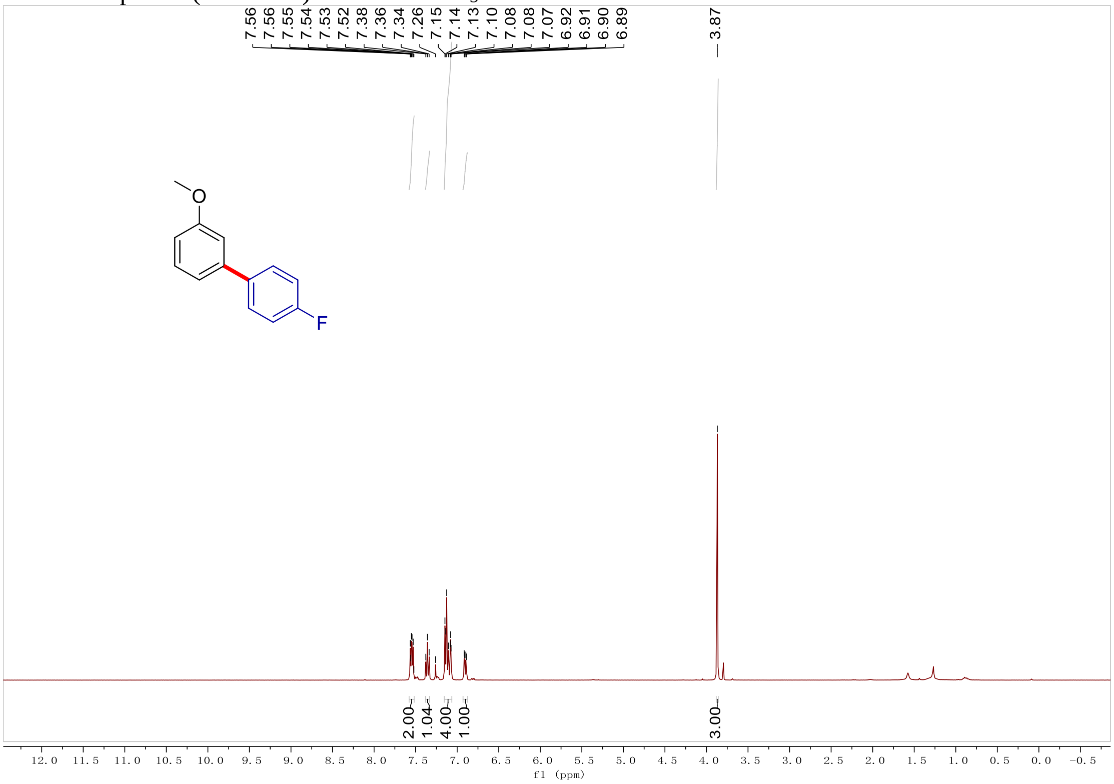

[13]**C NMR** spectra (101 MHz) of **87** in $CDCl_3$.

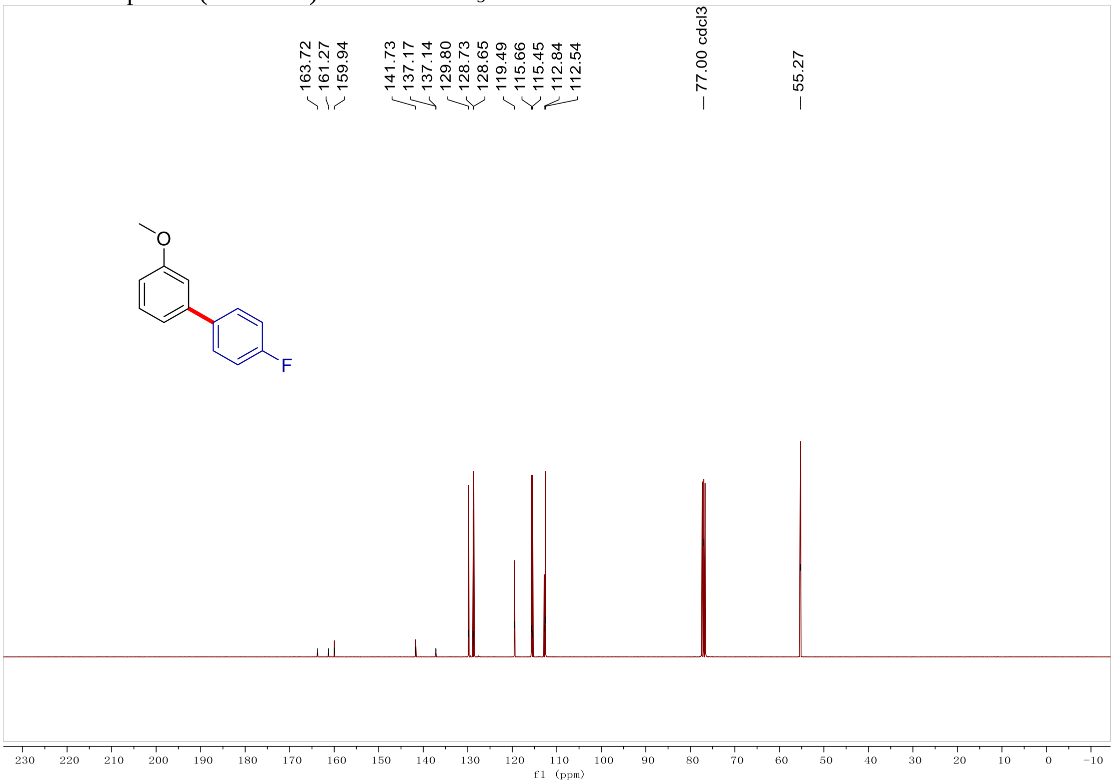

^{1}**H NMR** spectra (400 MHz) of **88** in $CDCl_3$.

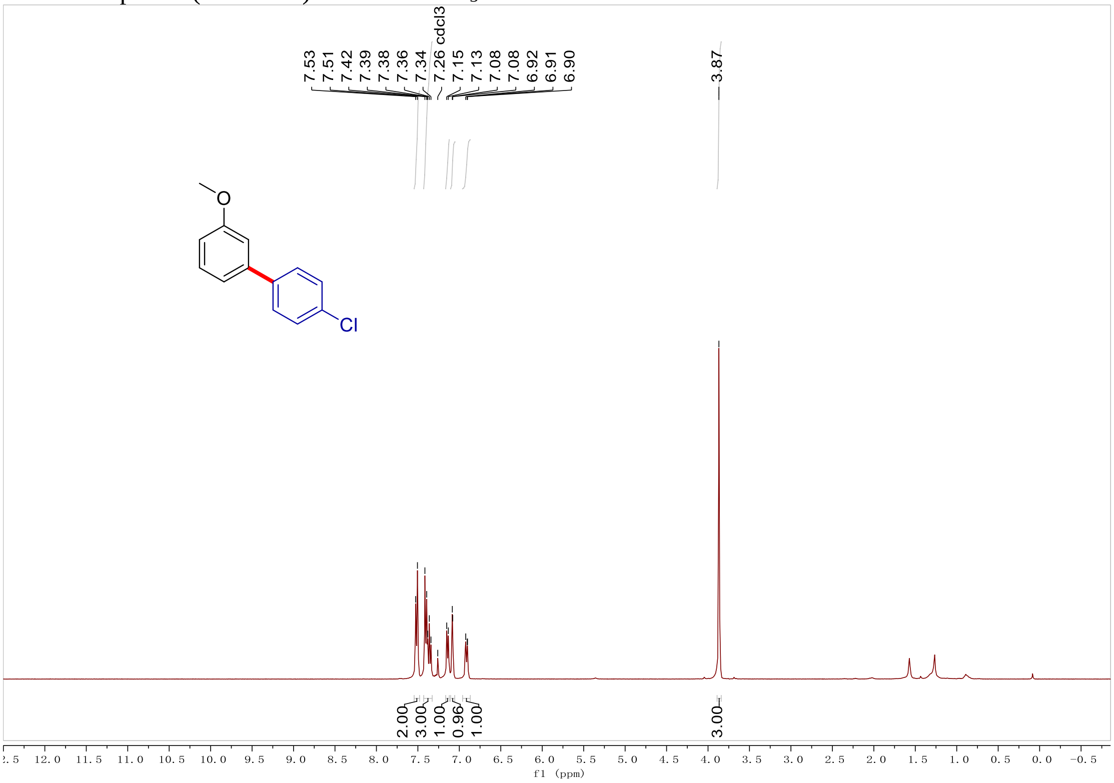

^{13}**C NMR** spectra (101 MHz) of **88** in $CDCl_3$.

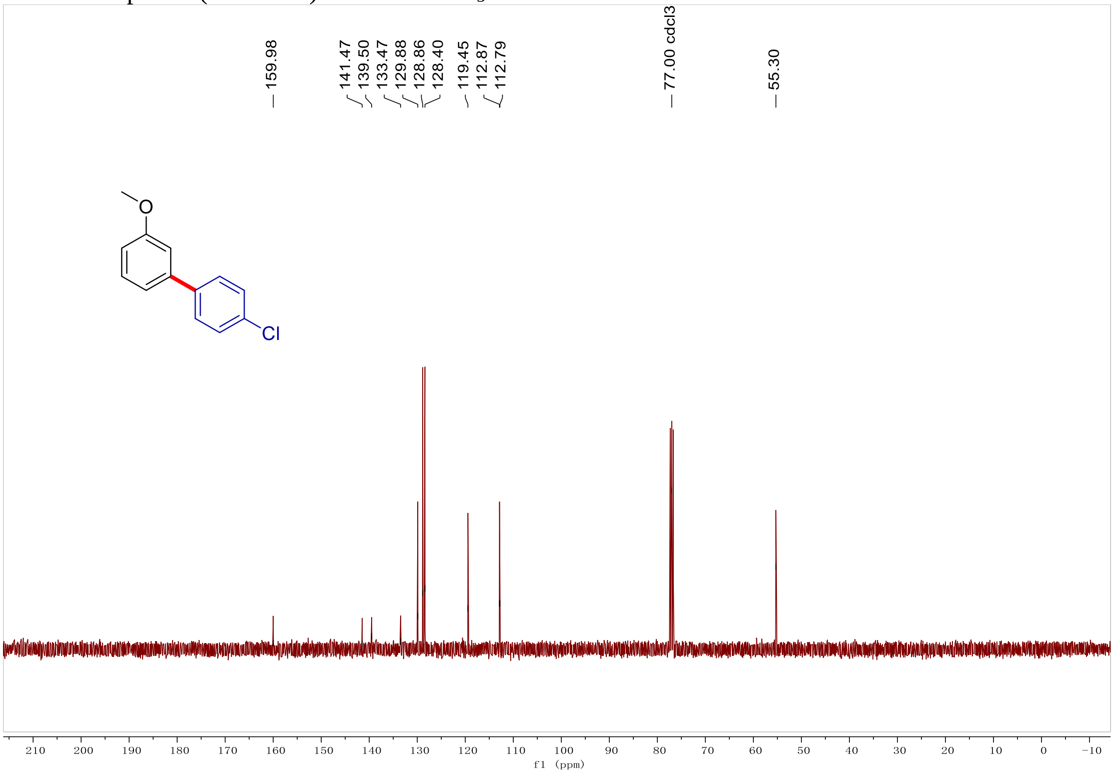

^{1}H NMR spectra (400 MHz) of **89** in $CDCl_3$.

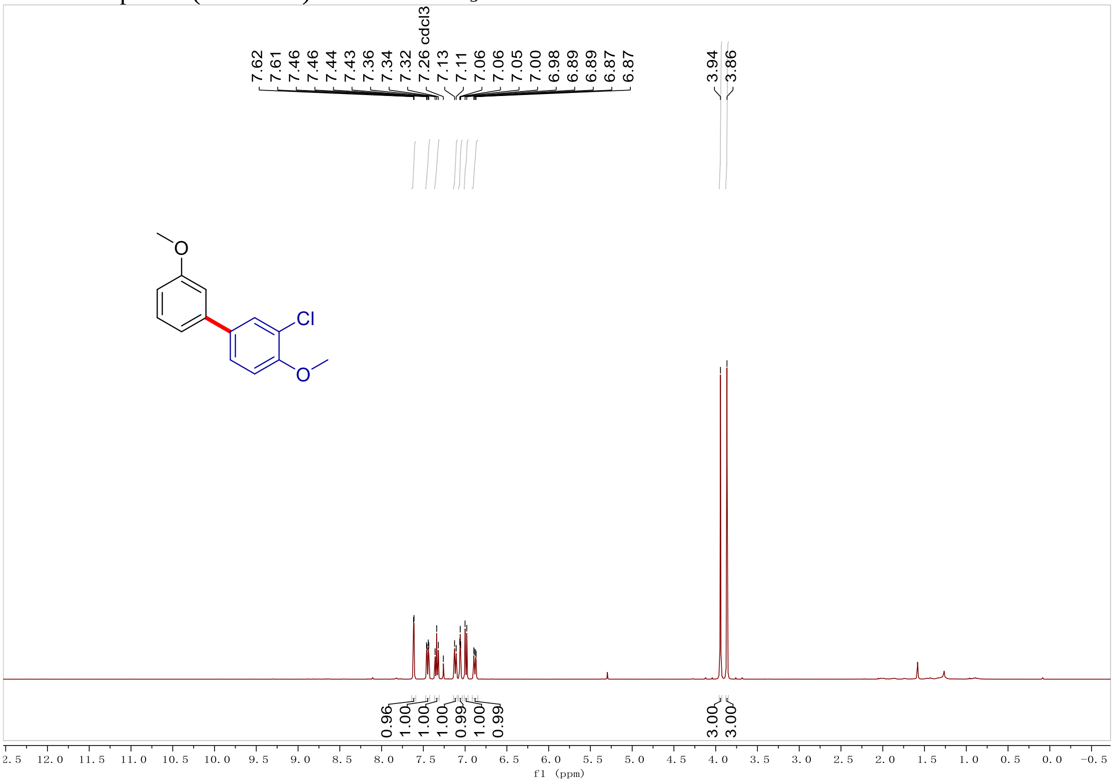

^{13}C NMR spectra (101 MHz) of **89** in $CDCl_3$.

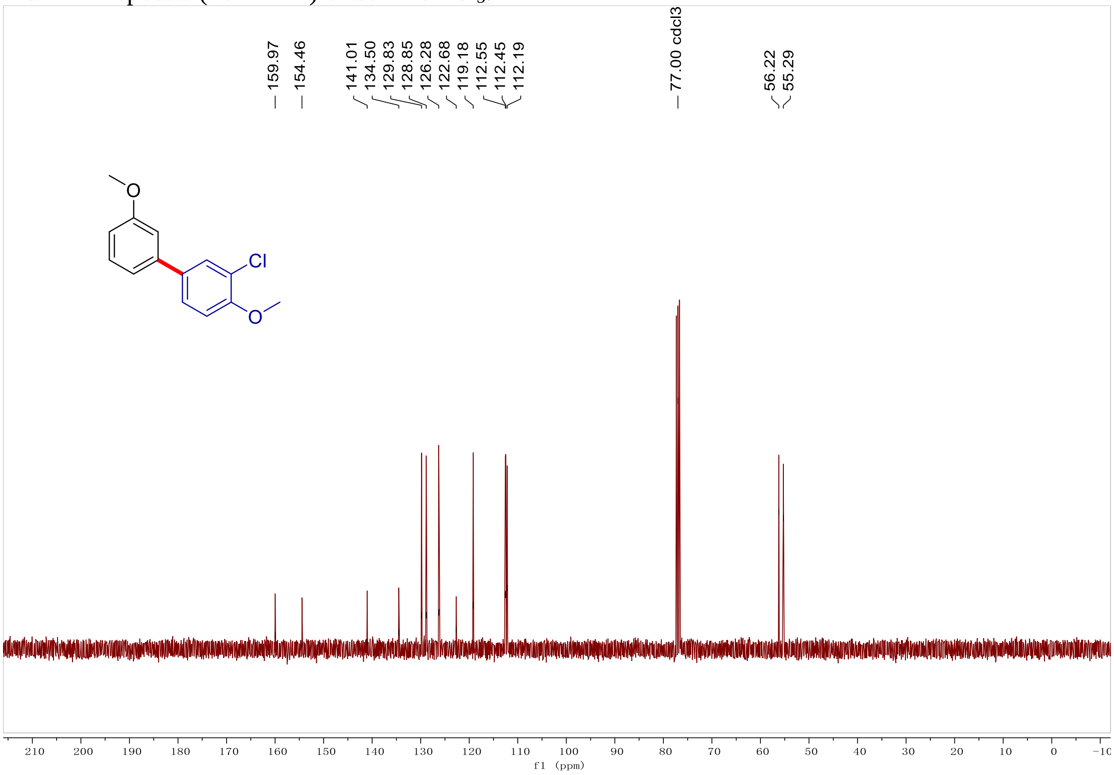

^{1}H NMR spectra (400 MHz) of **90** in $CDCl_3$.

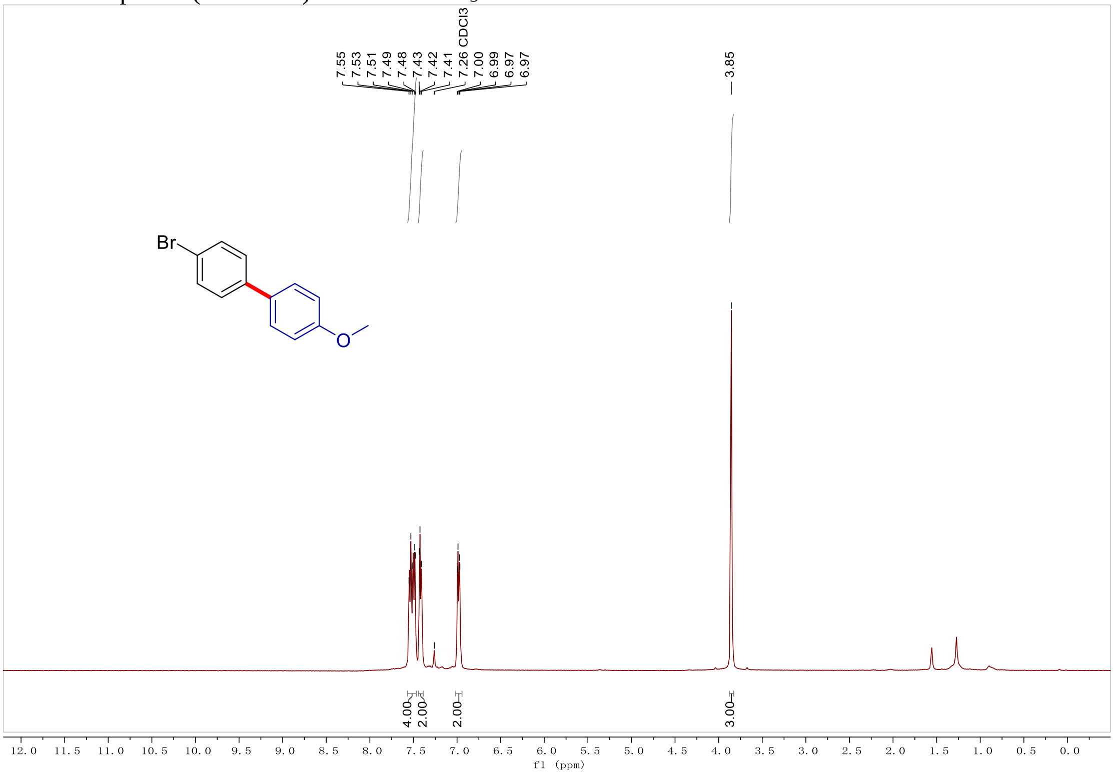

^{13}C NMR spectra (101 MHz) of **90** in $CDCl_3$.

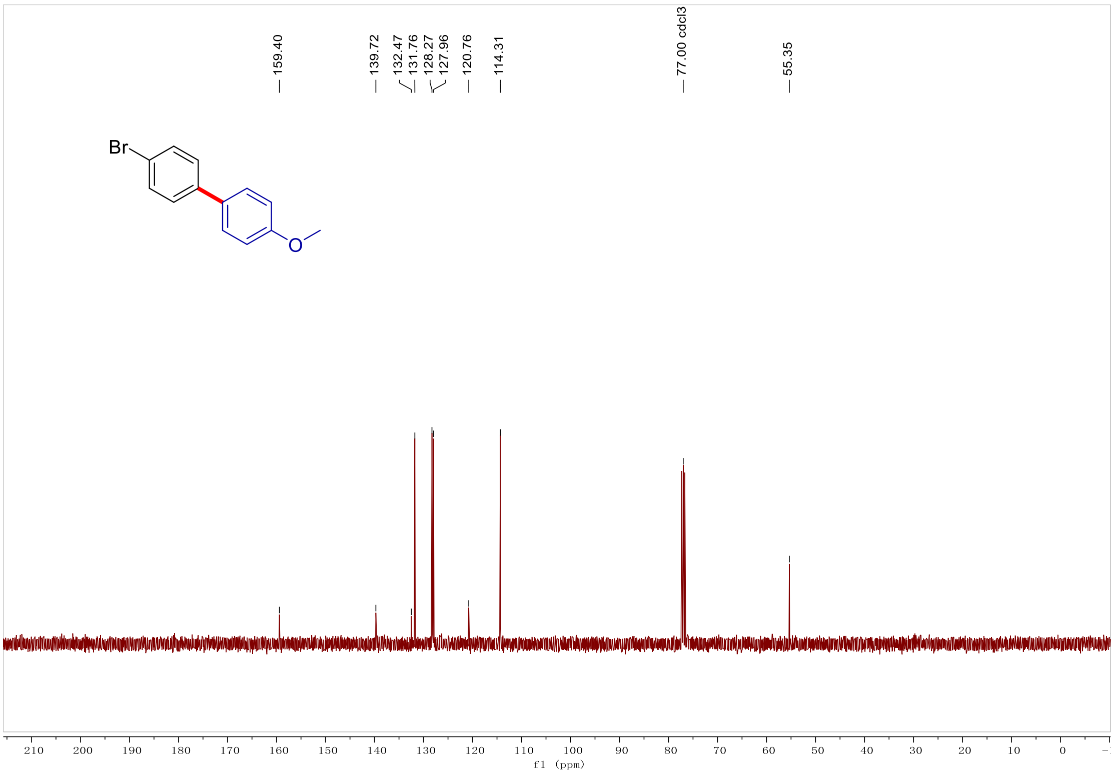

[1]**H NMR** spectra (400 MHz) of **91** in $CDCl_3$.

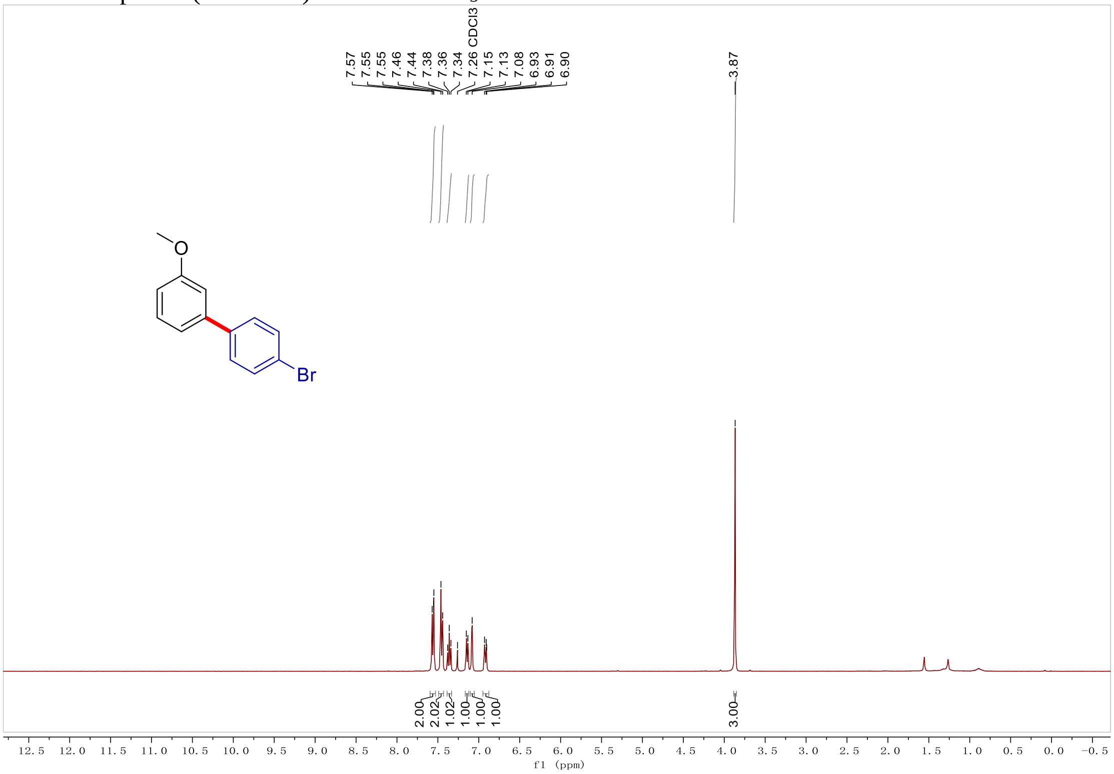

[13]**C NMR** spectra (101 MHz) of **91** in $CDCl_3$.

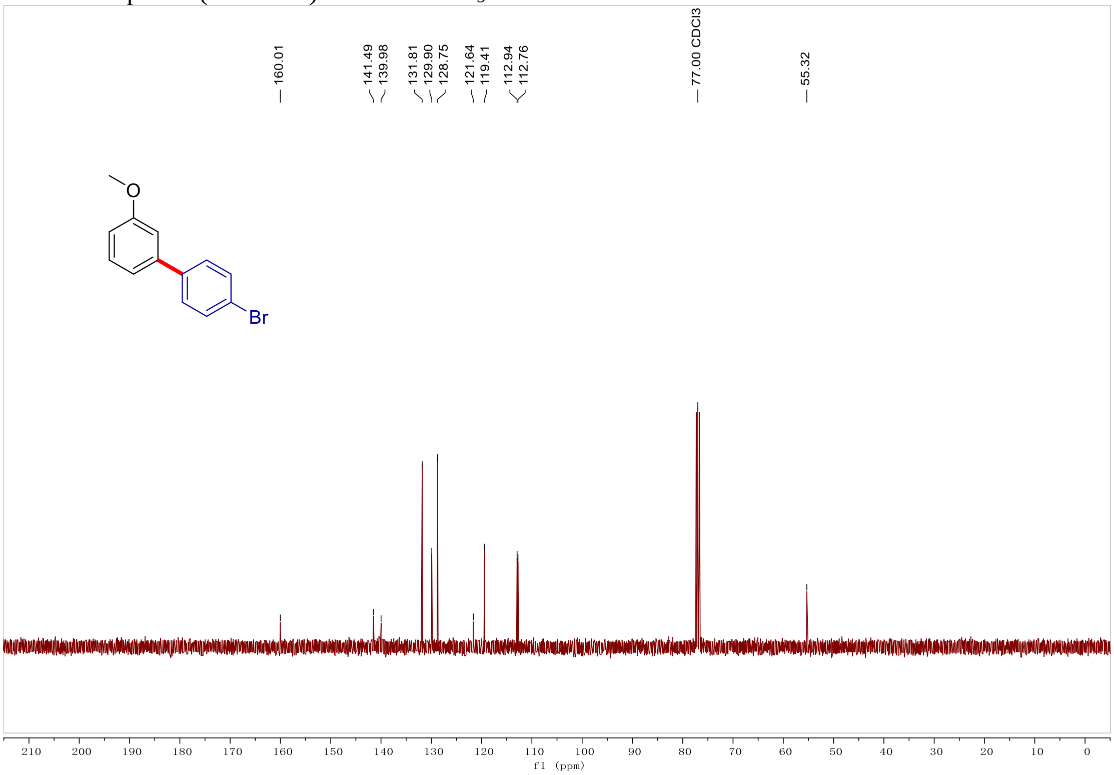

^{1}H NMR spectra (400 MHz) of **92** in $CDCl_3$.

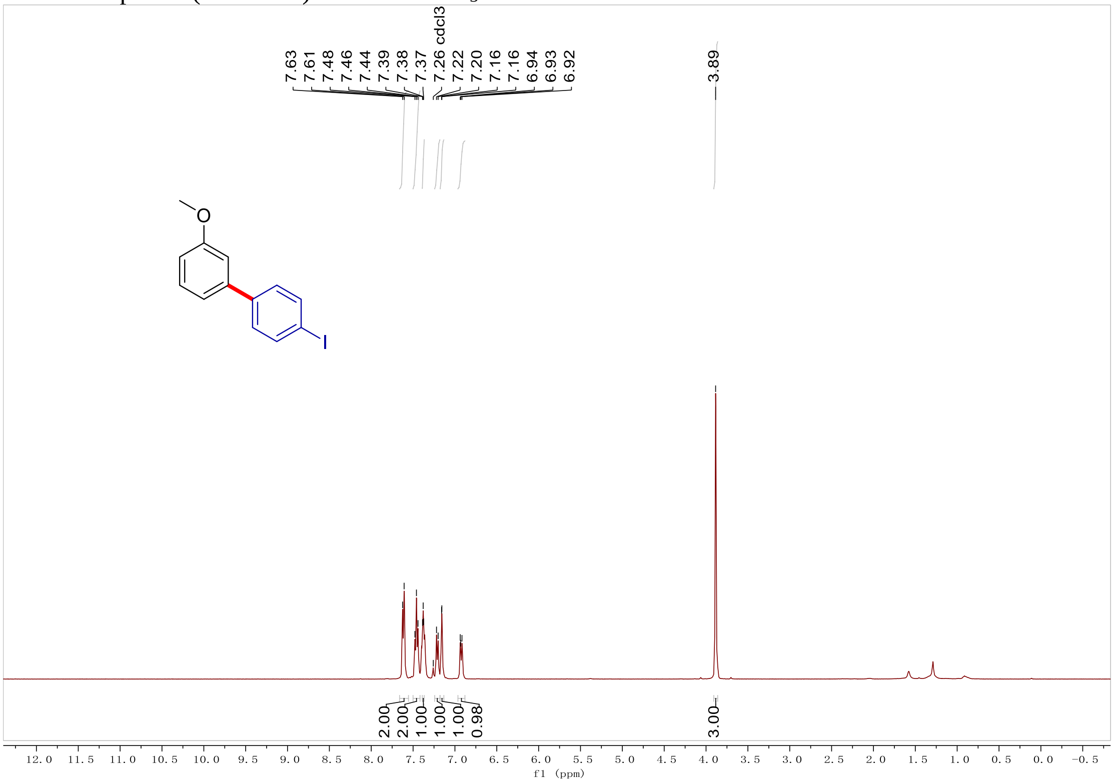

^{13}C NMR spectra (101 MHz) of **92** in $CDCl_3$.

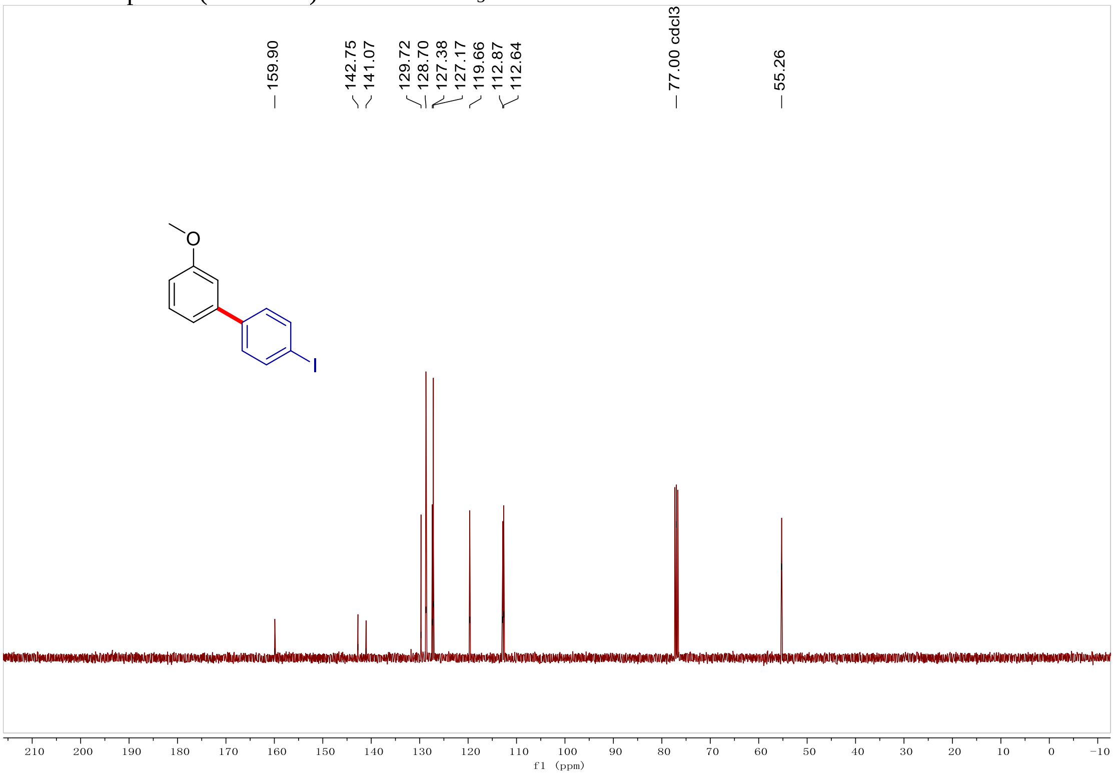

^{1}H NMR spectra (400 MHz) of **93** in $CDCl_3$.

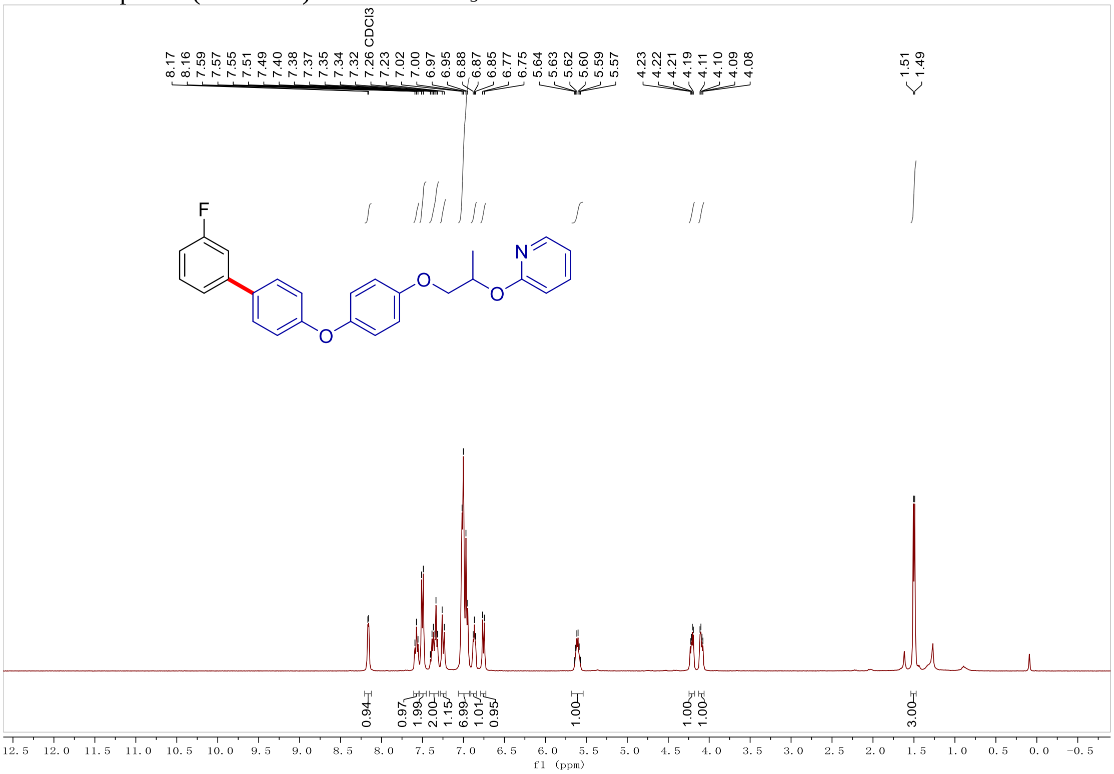

^{13}C NMR spectra (101 MHz) of **93** in $CDCl_3$.

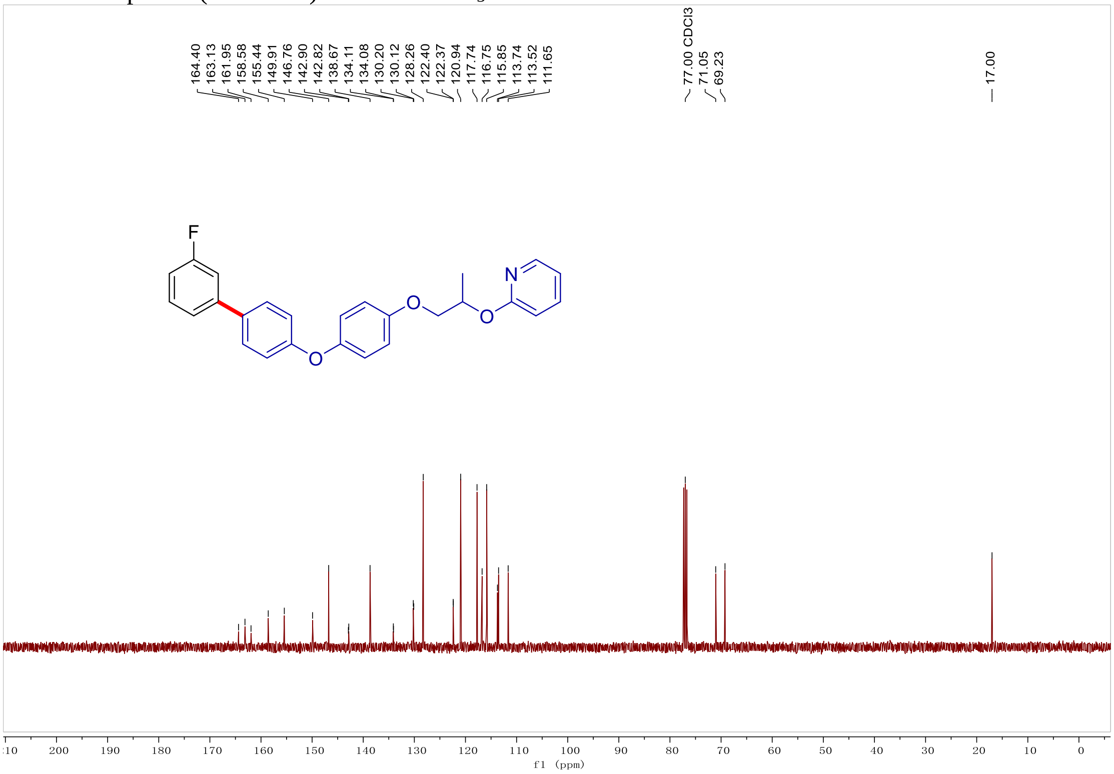

¹H NMR spectra (400 MHz) of **94** in $CDCl_3$.

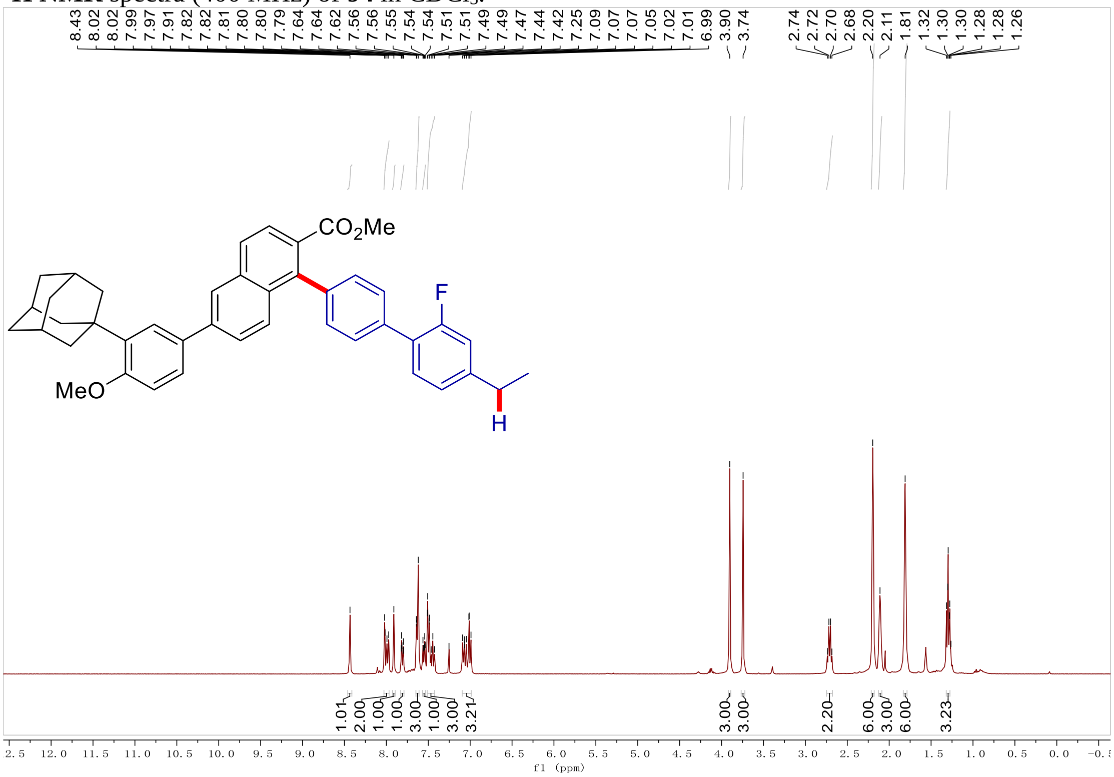

¹³C NMR spectra (101 MHz) of **94** in $CDCl_3$.

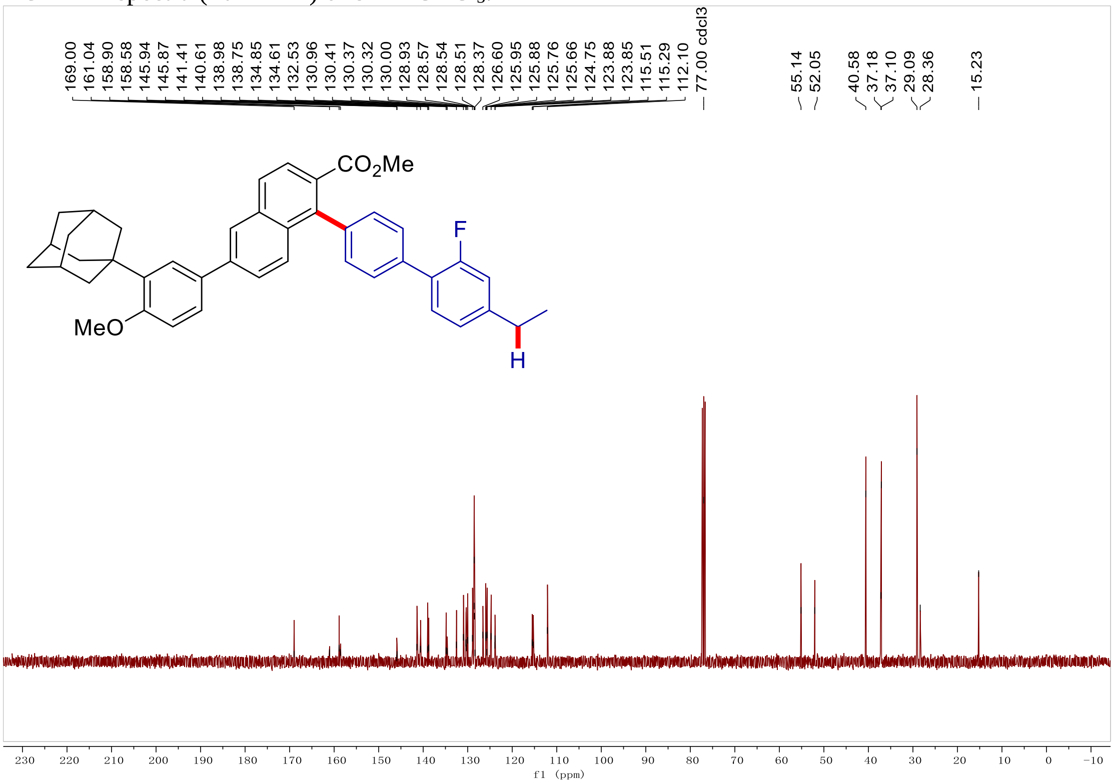

[1]**H NMR** spectra (400 MHz) of **12** in $CDCl_3$.

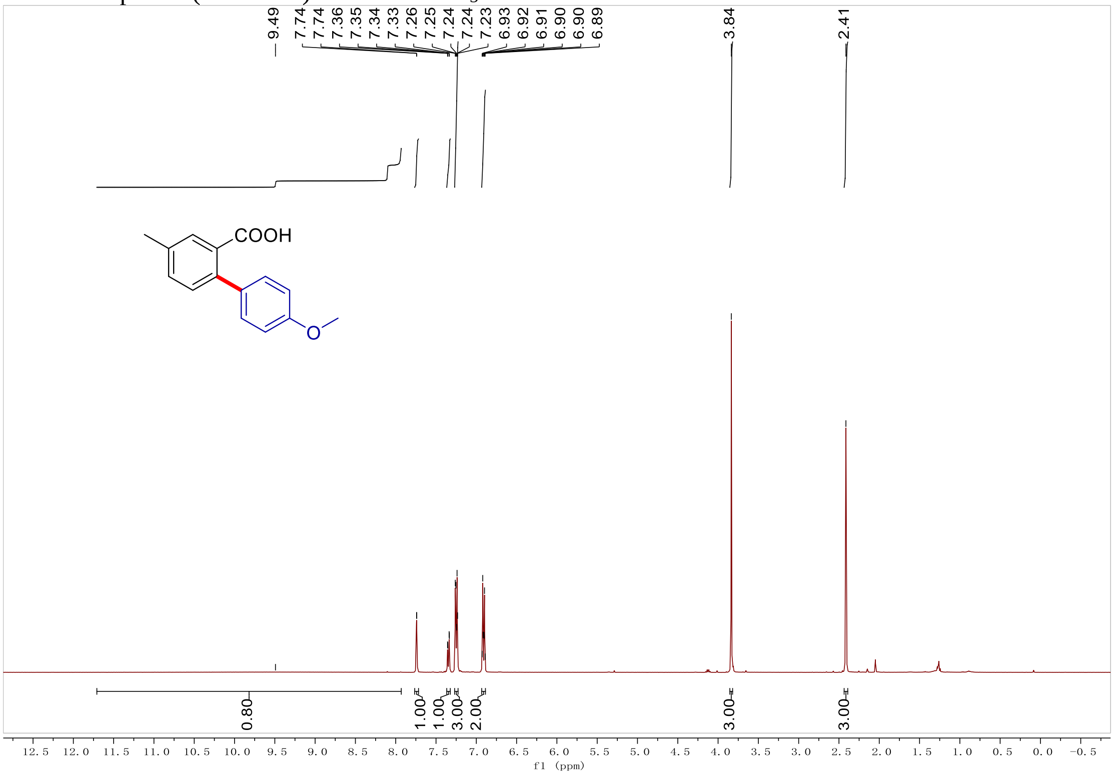

[13]**C NMR** spectra (101 MHz) of **12** in $CDCl_3$.

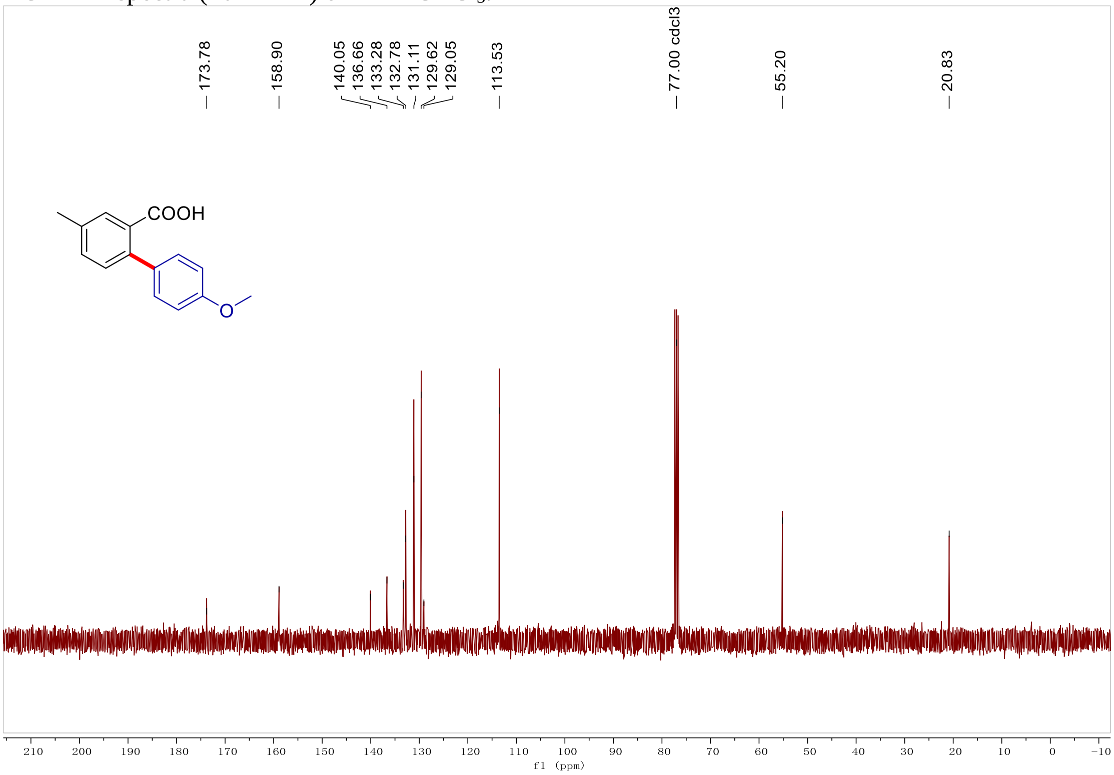

^{1}H NMR spectra (400 MHz) of **95** in $CDCl_3$.

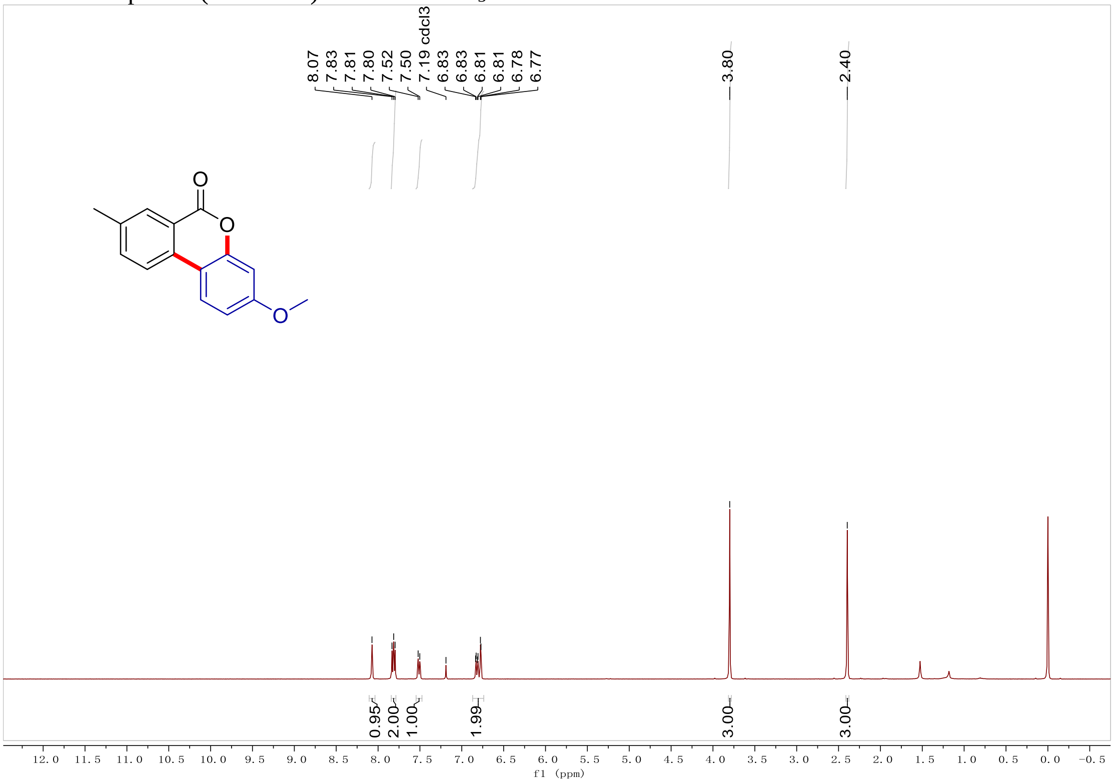

^{13}C NMR spectra (101 MHz) of **95** in $CDCl_3$.

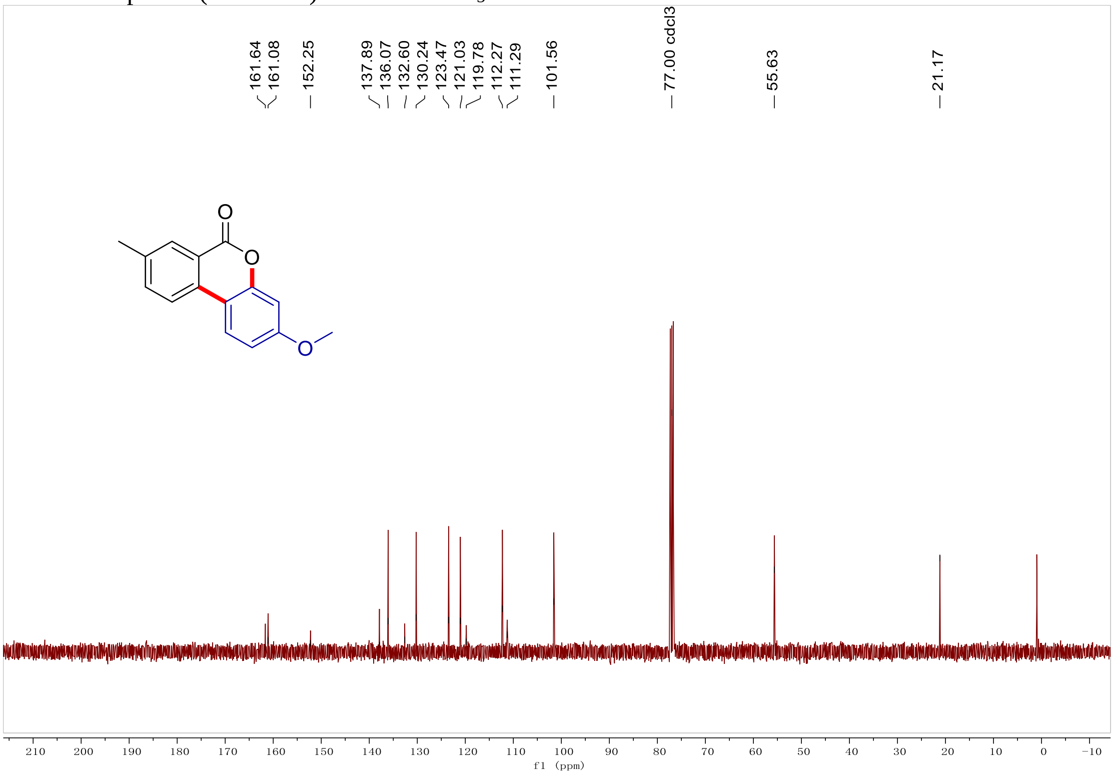

^{1}H NMR spectra (400 MHz) of **96** in $CDCl_3$.

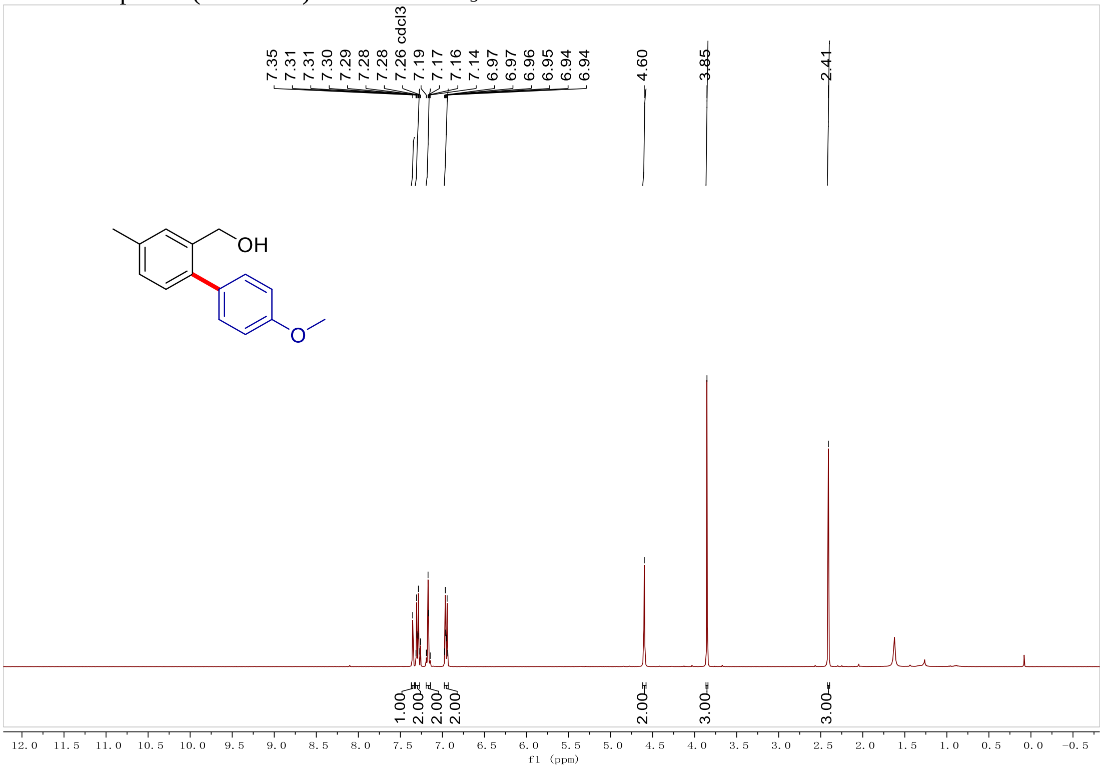

^{13}C NMR spectra (101 MHz) of **96** in $CDCl_3$.

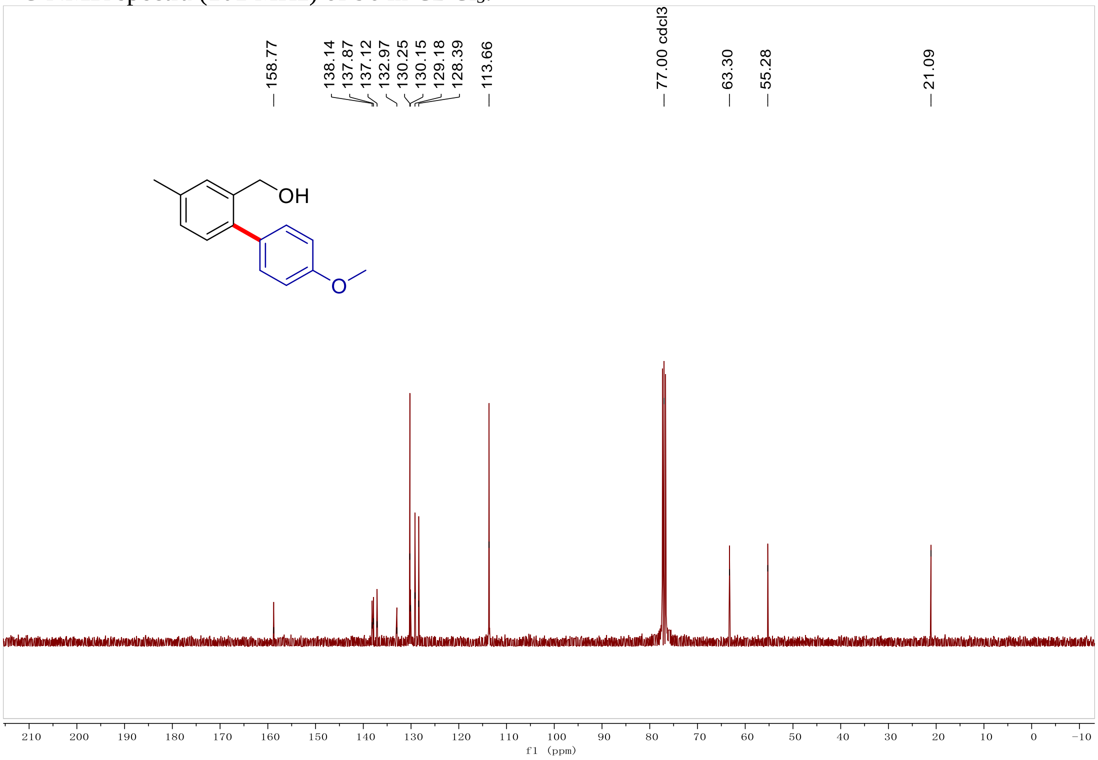

^{1}H NMR spectra (400 MHz) of **97** in $CDCl_3$.

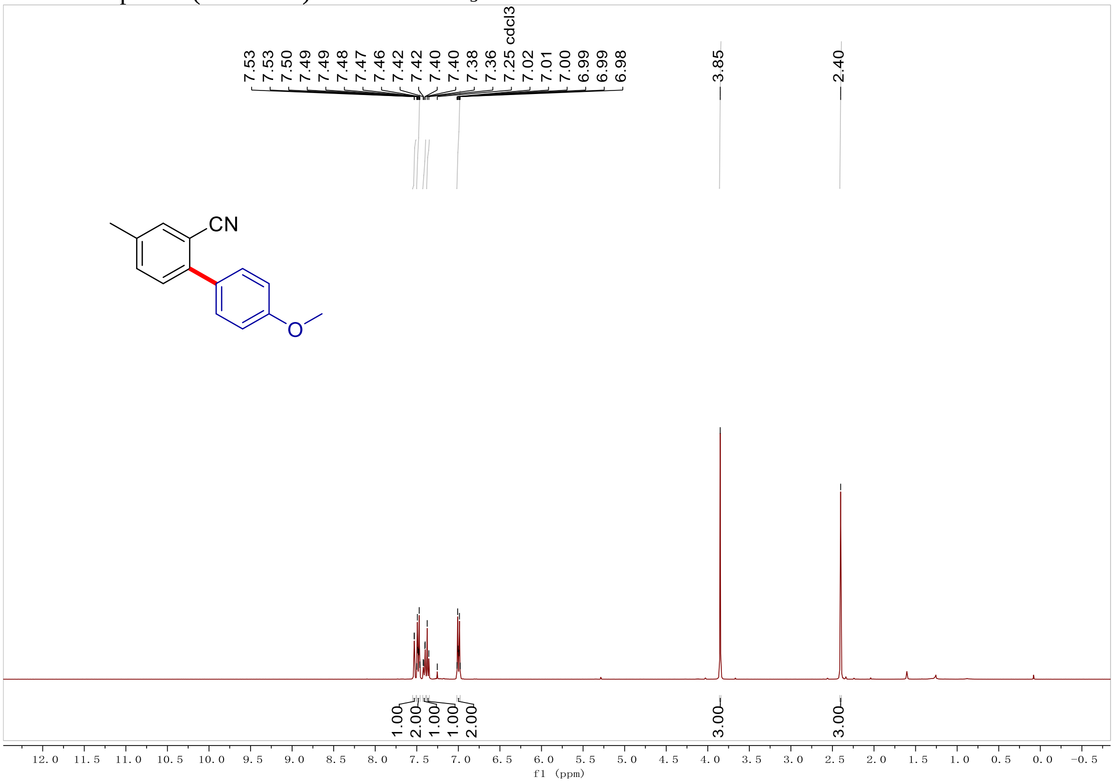

^{13}C NMR spectra (101 MHz) of **97** in $CDCl_3$.

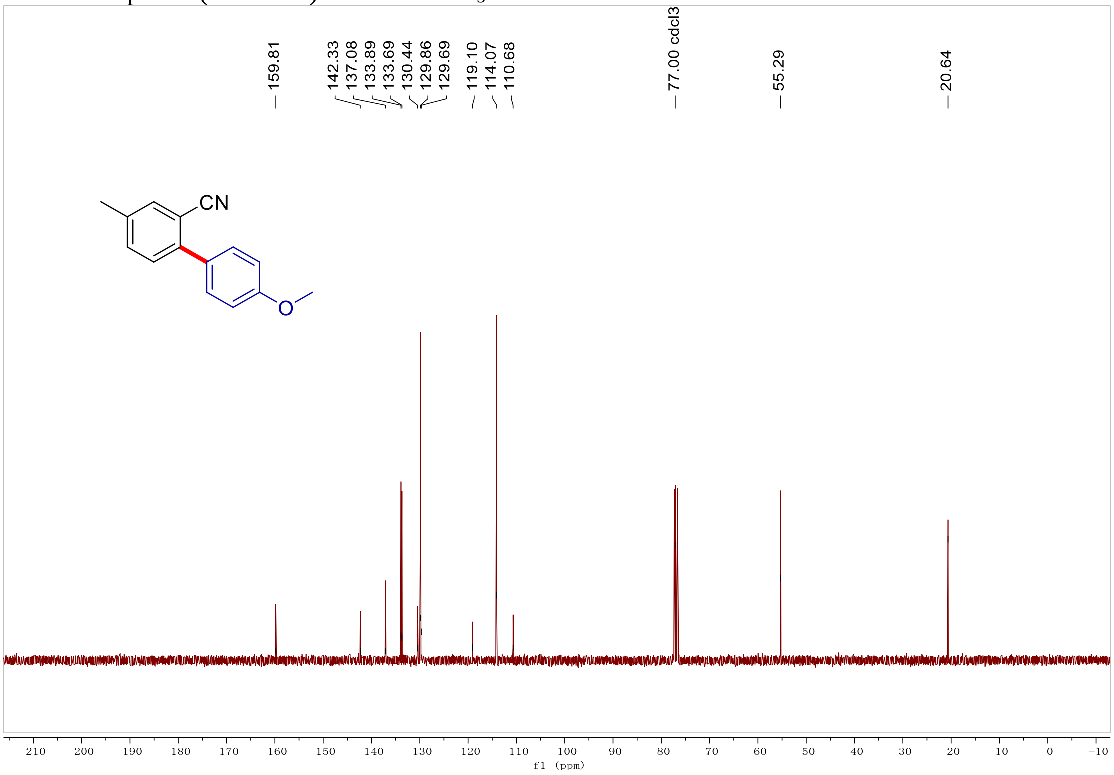

[1]**H NMR** spectra (400 MHz) of **98** in CDCl3.

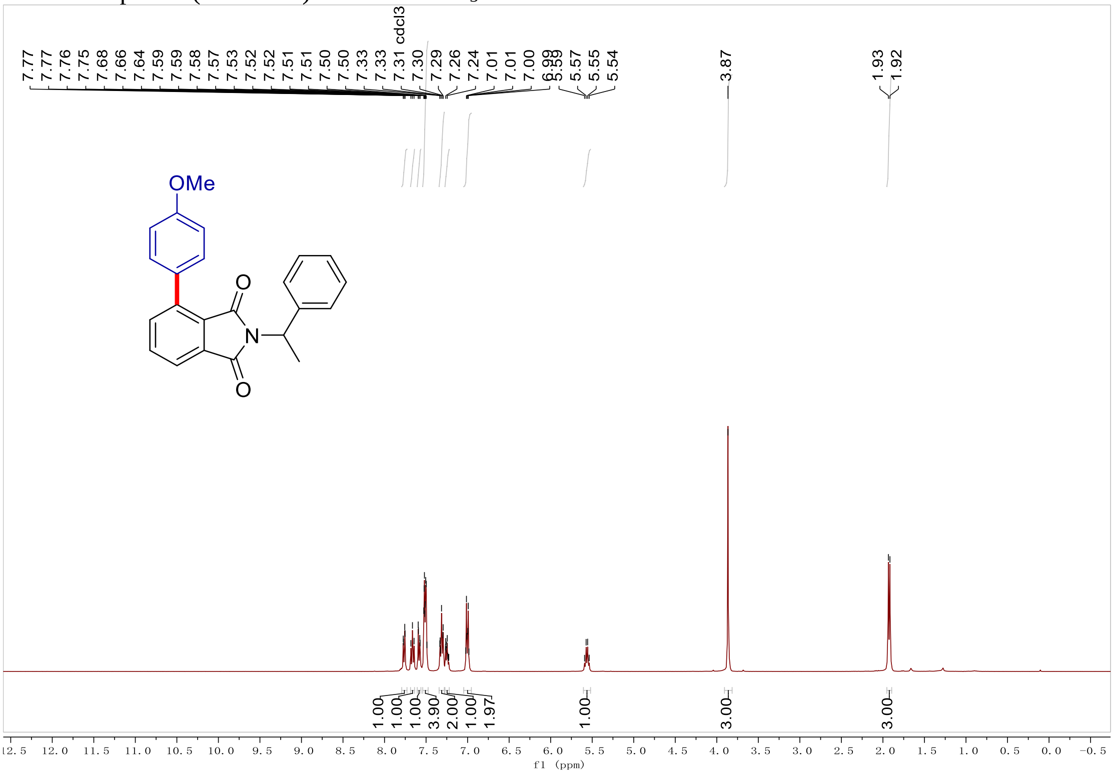

[13]**C NMR** spectra (101 MHz) of **98** in CDCl3.

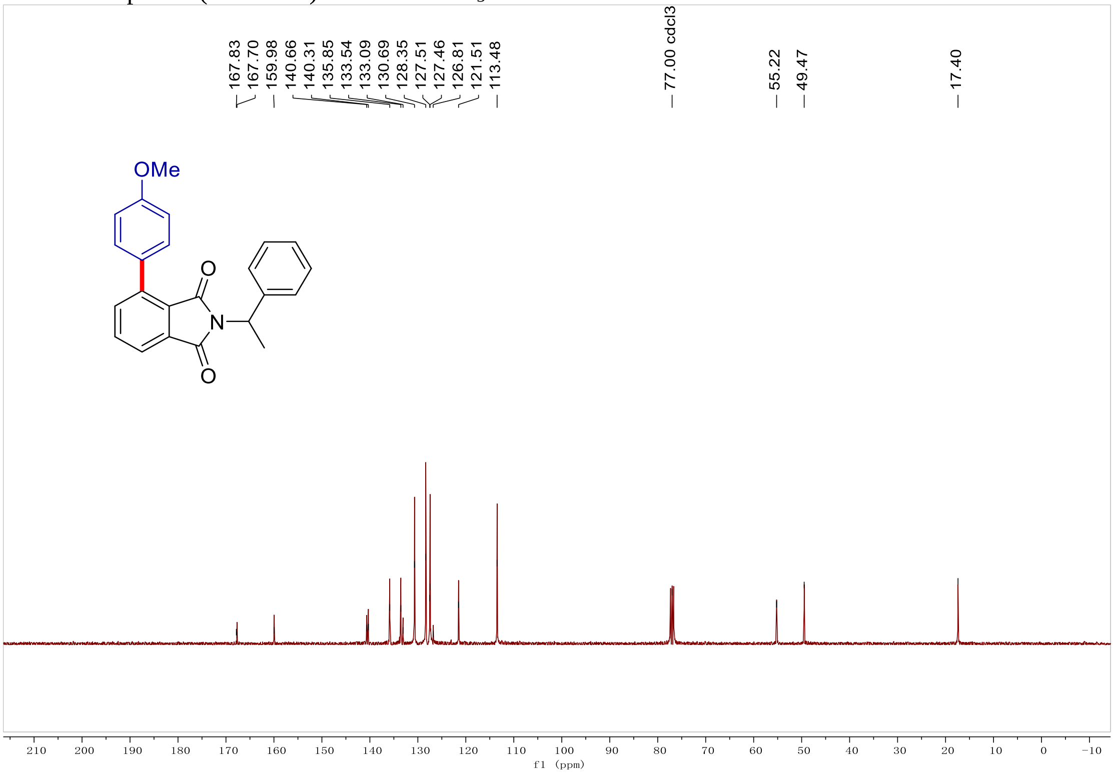

^{1}H NMR spectra (400 MHz) of **99** in $CDCl_3$.

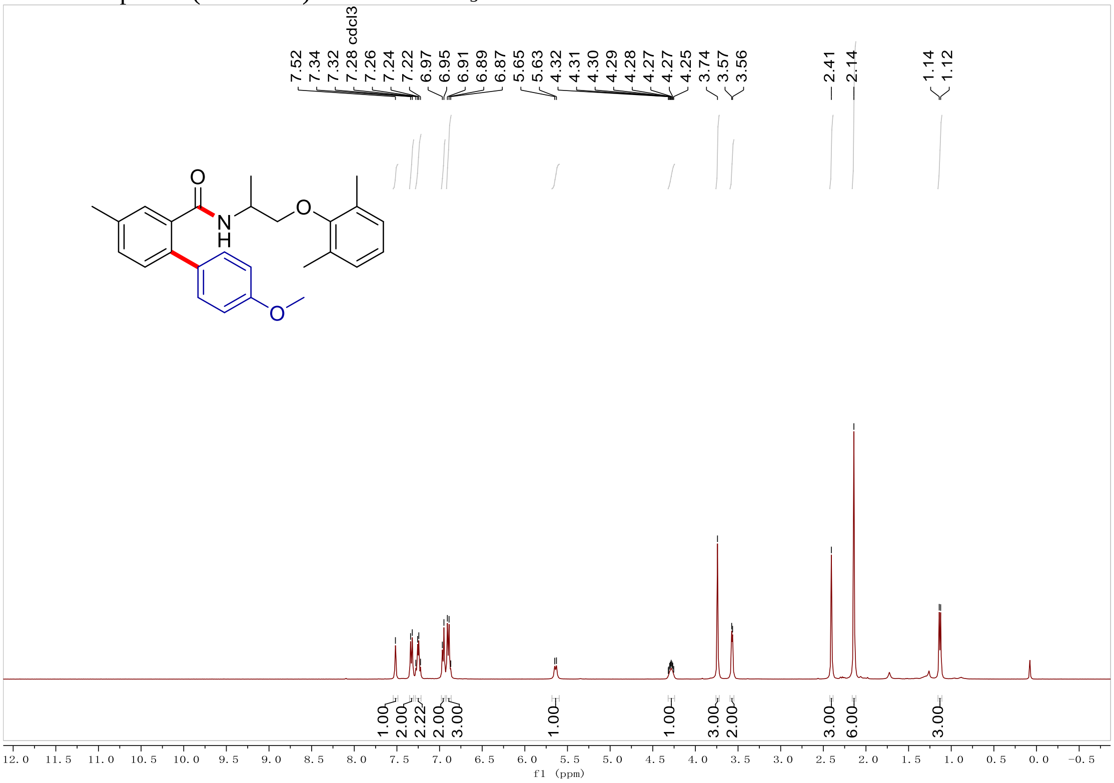

^{13}C NMR spectra (101 MHz) of **99** in $CDCl_3$.

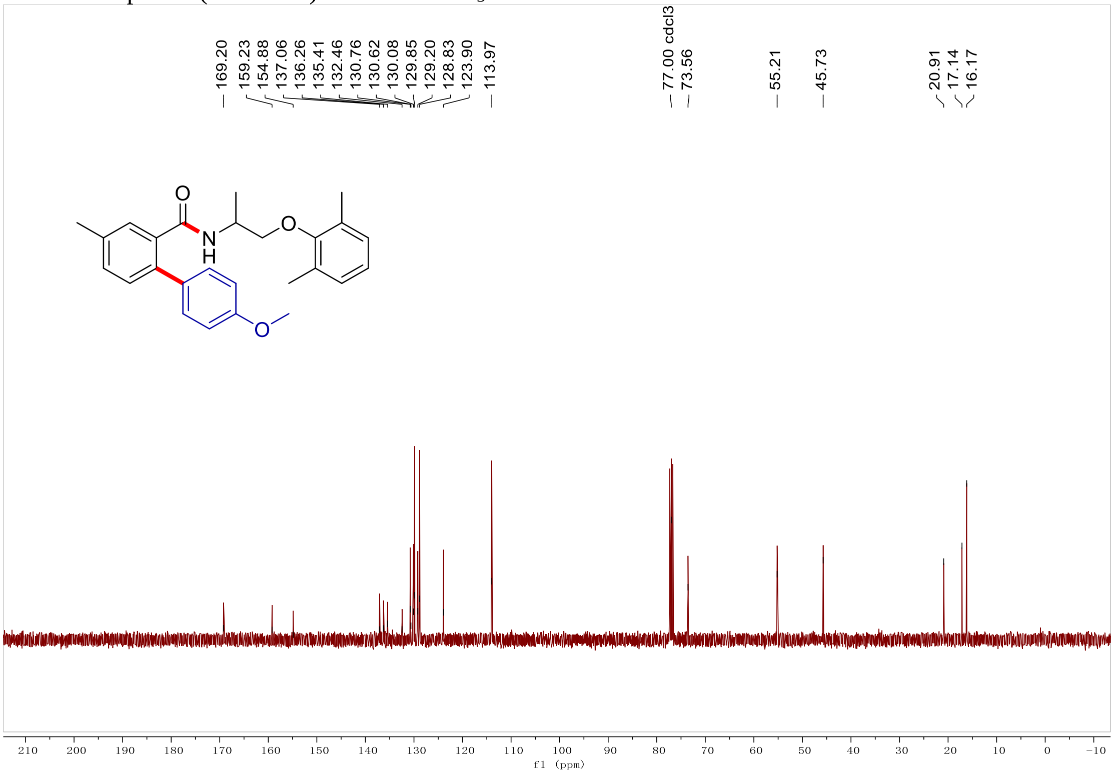

^{1}H NMR spectra (400 MHz) of **100** in $CDCl_3$.

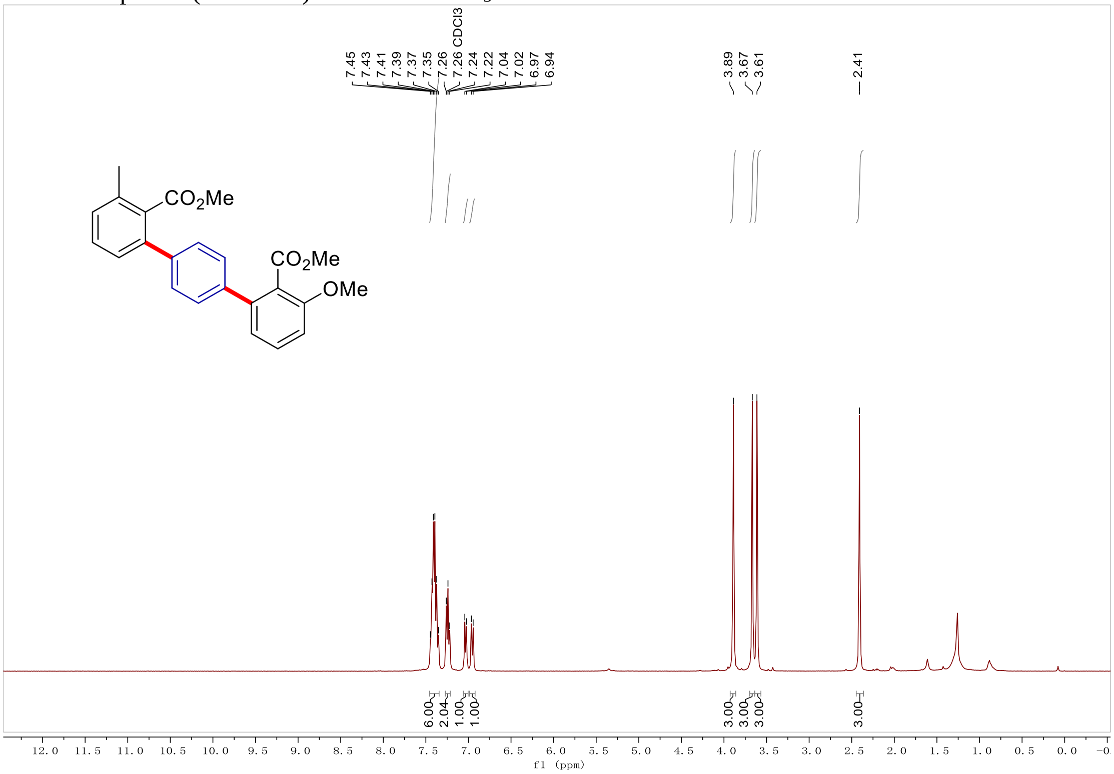

^{13}C NMR spectra (101 MHz) of **100** in $CDCl_3$.

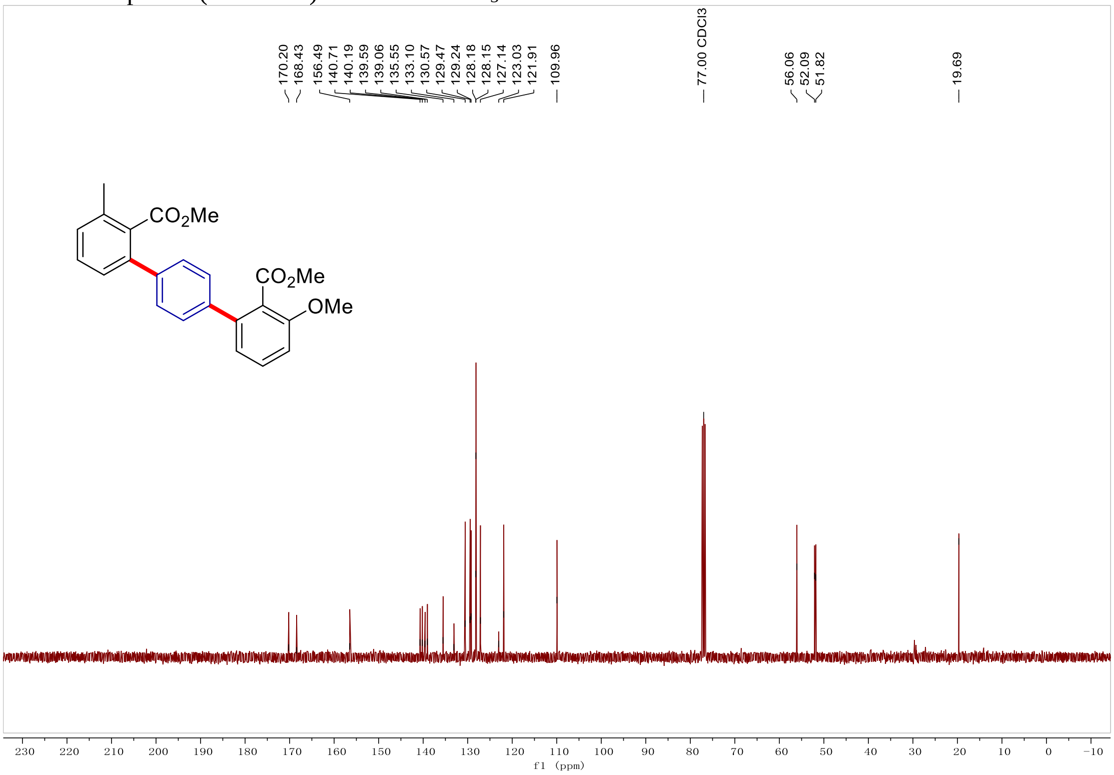

[1]**H NMR** spectra (400 MHz) of **101** in $CDCl_3$.

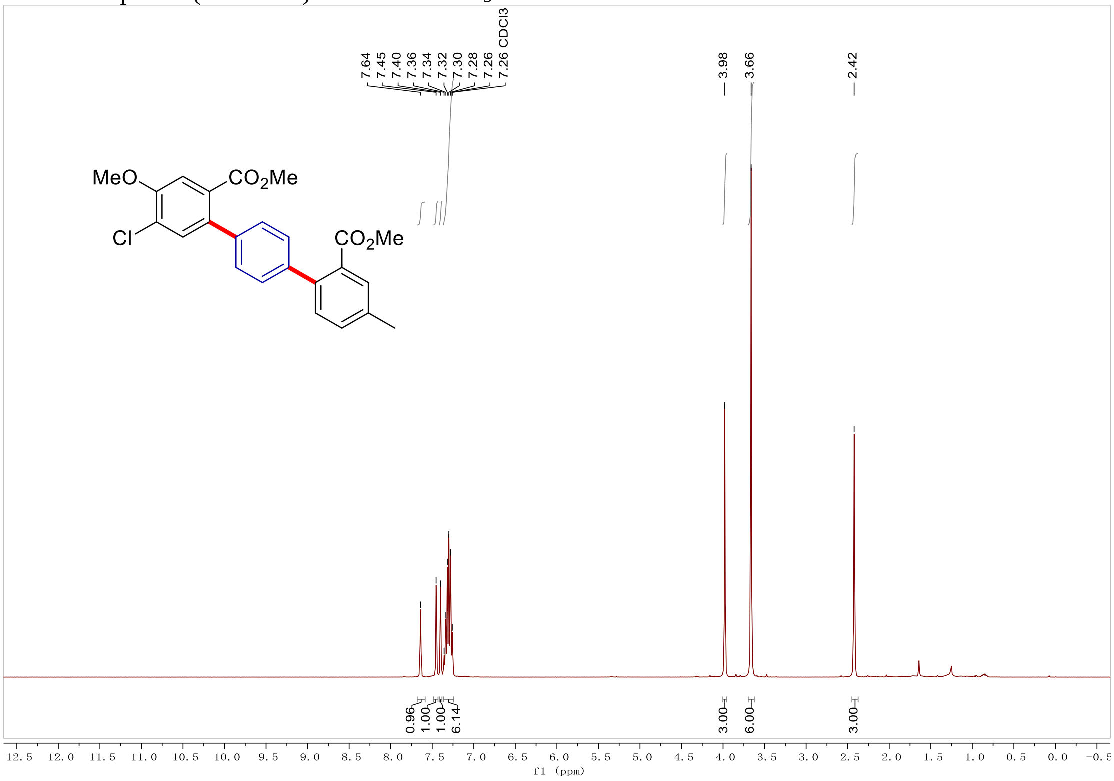

[13]**C NMR** spectra (101 MHz) of **101** in $CDCl_3$.

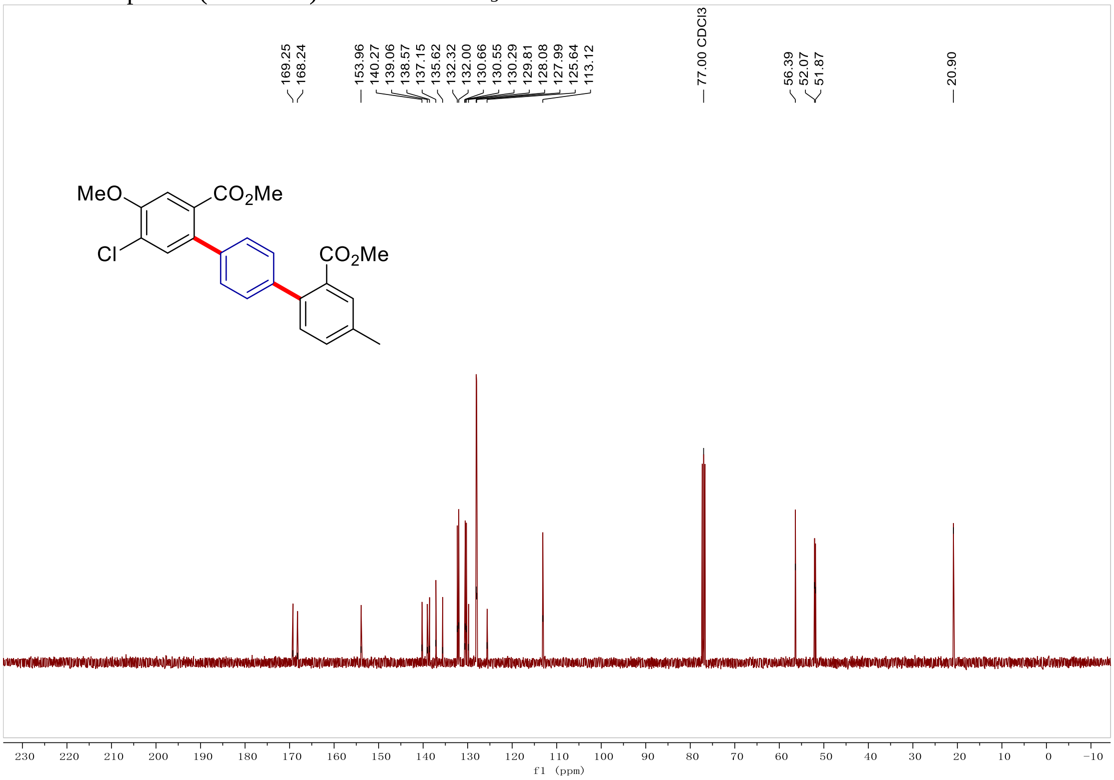

References

[1] a) Y. Wu, Y.-H. Huang, X.-Y. Chen, P. Wang, *Org. Lett.* **2020**, *22*, 6657–6661; b) Z.-W. Cao, J.-X. Zhang, J.-T. Wang, L. Li, X.-Y. Chen, S. Jin, Z.-Y. Cao, P. Wang, *Org. Lett.* **2024**, *26*, 6681–6686; c) Z.-H. Lin, Y.-F. Yao, C.-P. Zhang, *Org. Lett.* **2022**, *24*, 8417–8422; d) X.-Y. Chen, Y.-N. Li, Y. Wu, J. Bai, Y. Guo, P. Wang, *J. Am. Chem. Soc*. **2023**, *145*, 10431–10440.

[2] A. Biafora, T. Krause, D. Hackenberger, F. Belitz, L. J. Gooßen, *Angew. Chem. Int. Ed.* **2016**, *55*, 14752–14755.

[3] L. Huang, D. Hackenberger, L. J. Gooßen, *Angew. Chem. Int. Ed.* **2015**, *54*, 12607–12611.

[4] C.-A. Wang, N. Chatani, *Chem. Lett.* **2021**, *50*, 589–592.

[5] R.-H. Mei, C.-J. Zhu, L. Ackermann, *Chem. Commun.* **2016**, *52*, 13171-13174.

[6] M. Tobisu, K. Yasui, Y. Aihara, N. Chatani, *Angew. Chem. Int. Ed.* **2017**, *56*, 1877–1880.

[7] L.-B. Huang, D. J. Weix, *Org. Lett.* **2016**, *18*, 5432–5435.

[8] M. R. Yadav, M. Nagaoka, M. Kashihara, R.-L. Zhong, T. Miyazaki, S. Sakaki, Y. Nakao, *J. Am. Chem. Soc.* **2017**,*139*, 9423-9426.

[9] Prasanna, S. K. Bhat, K. M. Usha, *Catal Lett.* **2021***, 151*, 3313–3322.

[10] J.-A. Kan, S.-J. Huang, J. Lin, M. Zhang, W. Su, *Angew. Chem. Int. Ed.* **2015**, *54*, 2199–2203.

[11] X.-L. Luo, P.-C. Hou, J.-Y. Shen, Y.-F. Kuang, F.-C. Sun, H.-F. Jiang, L. J. Gooßen, L.-B. Huang, *Nat Commun.* **2024**, *15*, 5552.

[12] Y. Li, Y.-J. Ding, J.-Y. Wang, Y.-M. Su, X.-S. Wang, *Org. Lett.* **2013**, *15*, 2574–2577.

[13] R.-H. Mei, L. Ackermann, *Adv. Synth. Catal.* **2016**, *358*, 2443-2448.

[14] G.-D. Zhang, C.-Y. Zhang, Y. Tian, F. Chen, *Org. Lett.* **2023**, *25*, 917–922.

[15] X.-Q. Zhang, F.-K. Zhang, X.-L. Li, M.-Z. Lu, X. Meng, L. Huang, H.-Q. Luo, *Org. Lett.* **2022**, *24*, 5029–5033.
